# Genomic sequence analysis and characterization of *Sneathia amnii *sp. nov

**DOI:** 10.1186/1471-2164-13-S8-S4

**Published:** 2012-12-17

**Authors:** Michael D Harwich, Myrna G Serrano, Jennifer M Fettweis, João MP Alves, Mark A Reimers, Vaginal Microbiome Consortium (additional members), Gregory A Buck, Kimberly K Jefferson

**Affiliations:** 1 Department of Microbiology and Immunology, Virginia Commonwealth University School of Medicine, 1101 E. Marshall Street - PO Box 980678, Richmond, VA 23298-0678, USA; 2 Center for the Study of Biological Complexity, Virginia Commonwealth University, 1015 Floyd Avenue, PO Box 842030, Richmond, VA 23284-2030, USA; 3 Department of Biostatistics, Virginia Commonwealth University School of Medicine, 830 East Main Street - PO Box 980032, Richmond, VA 23298-0032, USA

## Abstract

**Background:**

Bacteria of the genus *Sneathia *are emerging as potential pathogens of the female reproductive tract. Species of *Sneathia*, which were formerly grouped with *Leptotrichia*, can be part of the normal microbiota of the genitourinary tracts of men and women, but they are also associated with a variety of clinical conditions including bacterial vaginosis, preeclampsia, preterm labor, spontaneous abortion, post-partum bacteremia and other invasive infections. *Sneathia *species also exhibit a significant correlation with sexually transmitted diseases and cervical cancer. Because *Sneathia *species are fastidious and rarely cultured successfully *in vitro*; and the genomes of members of the genus had until now not been characterized, very little is known about the physiology or the virulence of these organisms.

**Results:**

Here, we describe a novel species, *Sneathia amnii *sp. nov, which closely resembles bacteria previously designated "*Leptotrichia amnionii*". As part of the Vaginal Human Microbiome Project at VCU, a vaginal isolate of *S. amnii *sp. nov. was identified, successfully cultured and bacteriologically cloned. The biochemical characteristics and virulence properties of the organism were examined *in vitro*, and the genome of the organism was sequenced, annotated and analyzed. The analysis revealed a reduced circular genome of ~1.34 Mbp, containing ~1,282 protein-coding genes. Metabolic reconstruction of the bacterium reflected its biochemical phenotype, and several genes potentially associated with pathogenicity were identified.

**Conclusions:**

Bacteria with complex growth requirements frequently remain poorly characterized and, as a consequence, their roles in health and disease are unclear. Elucidation of the physiology and identification of genes putatively involved in the metabolism and virulence of *S. amnii *may lead to a better understanding of the role of this potential pathogen in bacterial vaginosis, preterm birth, and other issues associated with vaginal and reproductive health.

## Background

A recent study, based on phylogenetic and phenotypic analyses, showed that the organism previously named "*Leptotrichia sanguinegens" *should be reassigned to a separate genus. Thus, the genus *Sneathia *was described, and the species was formally named *Sneathia sanguinegens *[1]. Species of this genus are long, gram-negative, non-motile rods that sometimes exhibit bulbous protrusions [2]. A novel bacterium that is closely related to *S. sanguinegens *was isolated from amniotic fluid and published as "*Leptotrichia amnionii" *[3]. The species was not validly named and no type strain was designated. Subsequently, 16S rDNA phylogenetic analysis showed that "*L. amnionii" *is better assigned to the genus *Sneathia *[4]. Herein, we describe a vaginal isolate that phenotypically and phylogenetically resembles this bacterium. Our genomic and phenotypic data clearly support the reclassification of this species to the genus *Sneathia*, and we propose the designation *Sneathia amnii *sp. nov. The type strain of *S. amnii *is Sn35.

*Sneathia *species have been associated with serious obstetric complications including spontaneous abortions and preterm labor [3,5,6]. While the uterine cavity and amniotic fluid are usually sterile, bacterial infection can occur and is frequently associated with preterm labor and preterm premature rupture of fetal membranes [7]. *Sneathia *is one of the most common genera detected in amniotic fluid, and its presence can lead to inflammation, histological chorioamnionitis, and/or amnionitis [8]. Two recent studies investigated the rates of bacterial invasion of the amniotic cavity. Amniotic fluid samples were analyzed for the presence of bacteria, and of samples from women with preeclampsia that were positive for bacteria, 50% contained *Sneathia *while in samples from women presenting with preterm labor that were positive for bacteria, 25% contained *Sneathia *[9,10]. These findings suggest that *Sneathia *has the pathogenic capacity to invade the uterine cavity and the amniotic sac and thereby cause pregnancy complications. *Sneathia *has also been implicated in both infant and maternal post-partum bacteremia [11-13]. More recently, a case of septic arthritis in a healthy, non-pregnant woman was reported, demonstrating that this opportunistic pathogen has the potential to cause infections outside of the reproductive tract as well [11-13].

*Sneathia *has also been associated with bacterial vaginosis (BV), the most common vaginal disorder in women of reproductive age worldwide [14]. BV is thought to be a clinically significant disorder because it is associated with increased risk for preterm labor and increased rates of transmission of HIV and other sexually transmitted infections (STIs). BV is characterized by a decline in the number of healthy vaginal lactobacilli (long rods), and an increase in number of short rod-shaped and coccoid anaerobic bacteria. The overall bacterial load and species diversity are both drastically elevated in BV. The etiology of the disorder is poorly defined, complicating therapy, and BV tends to be recurrent [15]. Recent culture-independent techniques that comprehensively identify bacterial taxa associated with BV have been employed in an effort to better understand its etiology. Although these studies have revealed an association between BV and the presence of *Sneathia *sp., the role of these bacteria in the etiology and pathology of the disease remains undefined [16-18].

A recent study of the microbiome of the male urogenital tract found that men can also be colonized with *Sneathia*, and that there is a significant correlation between colonization with *Sneathia *and other sexually transmitted pathogens, suggesting that these bacteria can be sexually transmitted [19]. Another study investigated the rate of *Sneathia *colonization in female subjects with human papillomavirus (HPV), and while there was not a positive correlation between HPV and *Sneathia*, there was a significant correlation between colonization with *S. amnii *("*L. amnionii"*) and cervical cancer in HPV-positive subjects [20].

In summary, *Sneathia *appears to be a significant, emerging opportunistic pathogen that may play a significant role in vaginal and reproductive health. However, due to its fastidious nature, very little is known about the genus. Our analysis of mid-vaginal microbiome profiles from over 700 women who were recruited as part of the Vaginal Human Microbiome Project [21], revealed that *Sneathia *species, and especially *S. amnii *sp. nov., commonly inhabit the human vagina. In an effort to better define this microorganism, we cultured a vaginal isolate of *Sneathia*, analyzed its genetic virulence potential, and characterized its virulence properties *in vitro*. Herein, we describe *S. amnii *sp. nov. as a novel species that closely resembles isolates formerly designated as "*L. amnionii*," and designate the type strain as *S. amnii *Sn35. Furthermore, we sequenced and analyzed the genome of this strain, which represents the first genome for the *Sneathia *genus.

## Results and discussion

### *Sneathia *species are a common component of the vaginal microbiota

**Figure 1:**
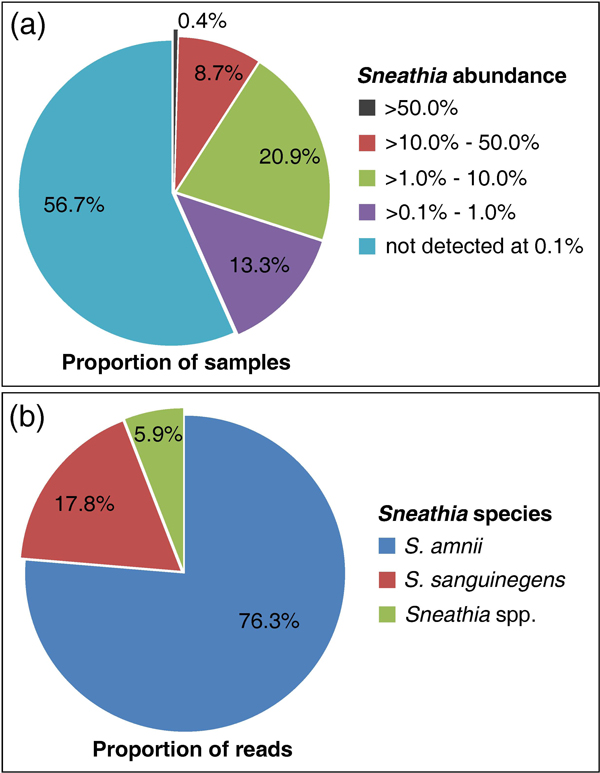
**Prevalence of *Sneathia *species in mid-vaginal samples**. (A) Relative abundance of *Sneathia *species in mid-vaginal samples. *Sneathia *was identified by 16S rDNA analysis in 319 of 736 mid-vaginal samples collected from volunteers recruited from outpatient clinics using a 0.1% abundance threshold; i.e., *Sneathia *was considered present if at least 0.1% of total 16S rRNA reads classified to *Sneathia*. (B) Abundance of different species of *Sneathia *(*S. sanguinegens*, *S. amnii*, or non-speciated *Sneathia*) in mid-vaginal samples. The majority of *Sneathia *reads (76.3%) identified in the mid-vaginal microbiome profiles classify to *Sneathia amnii*.

We collected mid-vaginal samples from 736 women visiting urban outpatient clinics in Virginia [21] for a variety of reasons (e.g., annual examination, vaginal discharge, STI screening, pregnancy, etc.). To identify the diversity of species present in these samples, we targeted the V1-V3 region of the 16S ribosomal-RNA-encoding gene. We applied a deep-sequencing approach that generated an average of ~30,000 reads per sample (mean = 30,165, median = 28,223), and therefore were able to detect taxa present in low abundance. *Sneathia *species were found in 43.3% (319 of 736) of these samples using an abundance threshold of 0.1% of total reads (Figure 1a). Using a more stringent abundance threshold of 1% of total reads (i.e., approximately 300 reads for an average sample), we still detected *Sneathia *in 30.0% of samples. Notably, in three mid-vaginal samples, more than half of the total reads classified to *Sneathia*. Thus, organisms in the genus *Sneathia *are common members of the vaginal microbiome, and they are occasionally identified as the predominant taxa.

### *S. amnii *in mid-vaginal microbiome profiles

The reference sequences of the V1-V3 regions of the 16S rRNA genes of *S. sanguinegens *and *S. amnii *sp. nov. are only 91% identical, permitting clear separation of the 16S rDNA metagenomic reads from these two species. Although we detected both *S. sanguinegens *and *S. amnii *in the mid-vaginal samples, the majority of *Sneathia *reads (76.3%) classified to *S. amnii *(Figure 1b). Moreover, both *S. amnii and S. sanguinegens *often co-occur in mid-vaginal samples. Using a 0.1% abundance threshold for presence, both *S. amnii *and *S. sanguinegens *were detected in 70.1% of mid-vaginal samples containing at least one of the two *Sneathia *species. However, approximately 6% of all reads classified as *Sneathia *do not clearly classify to one of the two known *Sneathia *species. Our preliminary results indicate that there is likely a third, less-abundant vaginal *Sneathia *that has yet to be described. Reads classified to the novel putative *Sneathia *taxon were detected in 1.4% of samples using a 0.1% abundance threshold, representing 1.3% of all reads classifying to *Sneathia *at the genus level. The V1-V3 rDNA sequences from these bacteria are ~91% and ~93% identical to corresponding rDNA sequences from *S. amnii *and *S. sanguinegens*, respectively, and these differences do not appear to be due to sequence errors or chimeras (data not shown). Thus, our results suggest that at least three related *Sneathia *types are found in vaginal samples. Herein, we focus on characterizing the *S. amnii *isolate, which we have successfully cultured and cloned, although we are continuing to attempt to culture and clone vaginal isolates of S. *sanguinegens *and the third, as yet unnamed, putative *Sneathia*.

### Phylogenetic analysis of the 16S rDNA of *S. amnii*

**Figure 2:**
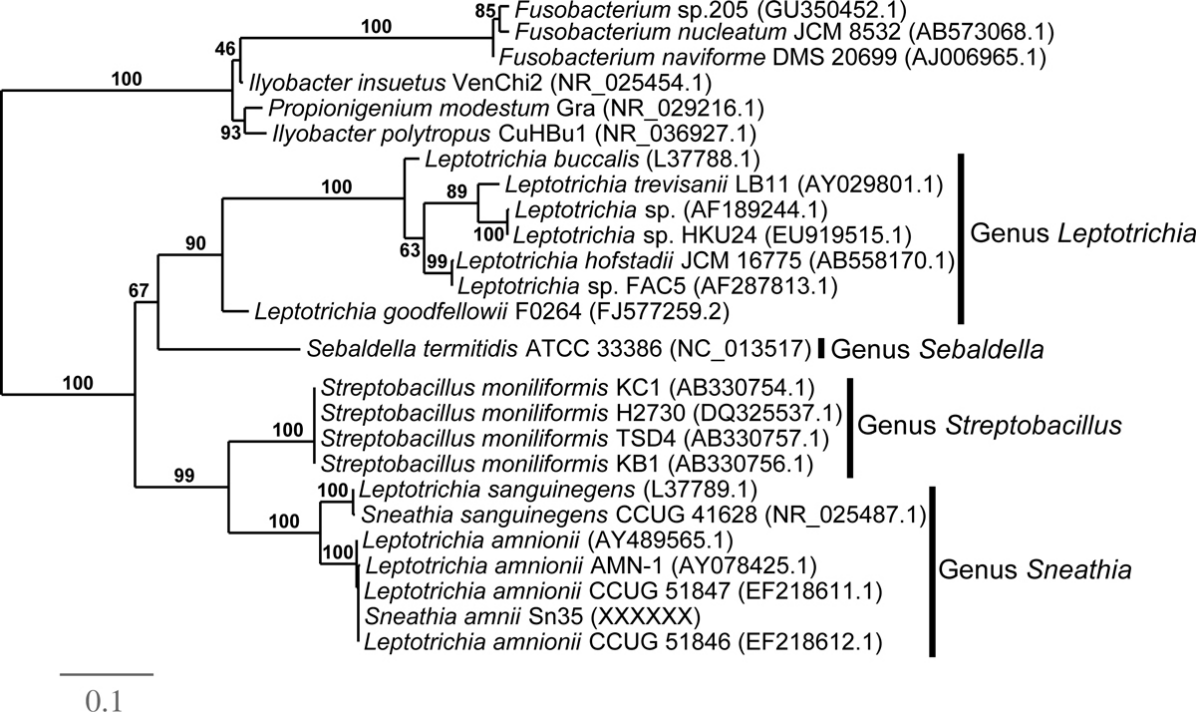
**Maximum likelihood phylogenetic tree of *S. amnii *and related organisms within the family Fusobacteriaceae**. The tree was inferred from 1,271 aligned characters of the 16S rRNA gene sequence (Additional file 1) and rooted in accordance with the current taxonomy. Numbers at nodes correspond to the support values from 1,000 bootstrap replicates.

To better define the role of *S. amnii *sp. nov. in vaginal health and disease, we isolated a clone from a mid-vaginal sample taken from an African American woman in her early 20's presenting with symptoms of preterm labor at 26 weeks of gestation. The 16S rRNA gene of this isolate was sequenced in its entirety and aligned with the 16S rDNAs from other members of Fusobacteriaceae family (Additional file 1) to assess their phylogenetic relationships (Figure 2). This alignment showed that the 16S rDNA of our isolate is 99.8% identical to the 16S rDNA of the isolate first described as "*L. amnionii" *(accession number AY078425.1). This result, along with phenotypic characteristics, suggests that the *Sneathia *isolate described herein is very similar to the bacterium previously described by Shukla et al. (2002) [3]. Furthermore, the 16S rDNA of our *Sneathia *isolate exhibited 94.7% overall sequence identity to *S. sanguinegens *(accession numbers L37789.1 and NR_025487.1), but only 84.4% overall sequence similarity to *Leptotrichia buccalis *(accession numbers L37788.1), the type species of the genus *Leptotrichia*. Phylogenetic analysis of the 16S rDNA gene, are consistent with the classification of this bacterium to the genus *Sneathia*, as previously proposed by Eribe et al. [4] for the bacterium termed *L. amnionii*. Therefore, we propose *S. amnii *sp. nov. as the name for the species. Our analyses also confirm, as previously shown [1], the close relationship (~88% across the rDNA, and similar relationships among other genes) between *Sneathia *and *Streptobacillus moniliformis*, the causative agent of Rat bite fever [22].

### The genome of *S. amnii *sp. nov

**Figure 3:**
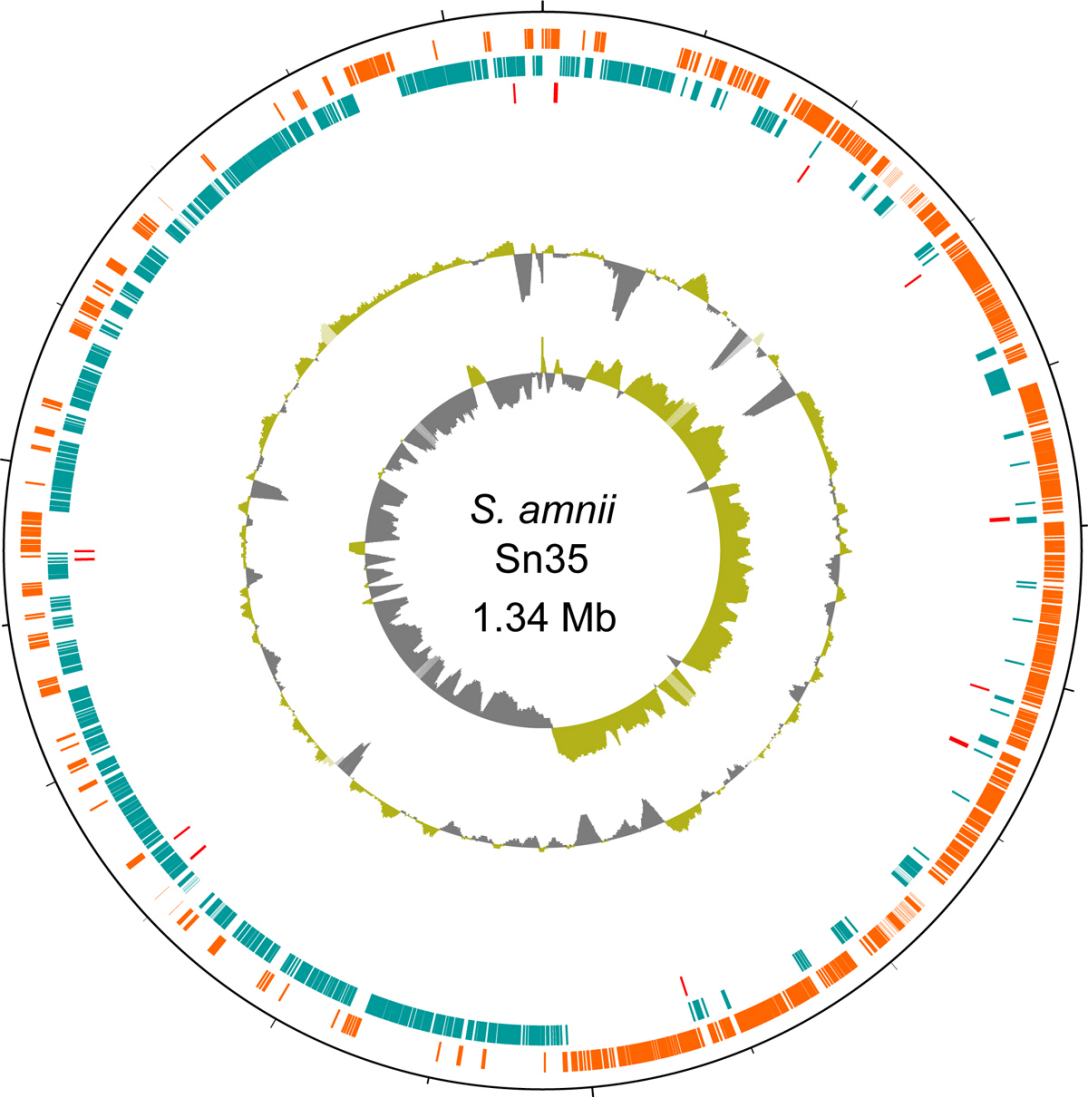
**Genomic atlas of *S. amnii *Sn35**. Coordinates are indicated starting from the predicted origin of replication at base 1. Circles represent the following (from the outer circle inward): 1) Coding regions are marked on the first two rings: orange color if encoded on the positive strand and teal color if encoded on the negative strand. 2) location of the tRNA. 3) GC content using a 10-kbp sliding window, green, positive GC skew; gray, negative GC skew. The innermost graph shows the GC skew, with sharp changes in skew occurring at the putative origin and terminus of replication. The atlas was constructed using DNAPlotter [58].

General features of the *S. amnii *genome are shown and compared to the genomes of the related bacteria *S. moniliformis, L. buccalis, and Sebaldella termiditis *in Table 1. The genome was sequenced to ~245 fold coverage using Roche 454 FLX Titanium technology, and this sequence assembled into one scaffold of 1,339,284 bases (Figure 3). This initial assembly was interrupted by 27 sequence gaps ranging from ~225 to 4200 nucleotides. Gaps within the scaffold were filled by PCR ampliflication and capillary sequencing of the amplicons. Although mate pair sequencing permitted the assembly of the genome into a single circular scaffold, the final (~11) sequence gaps represent a very small fraction of the genome (a few hundred bases) and therefore are unlikely to impact our analyses.

**Table 1:** General features of *S. amnii *genome and related organisms.

	*S. amnii*	*S. moniliformis*	*L. buccalis*	*S. termiditis*
**Approximate Genome Size (bp)**	**1,339,284**	**1,673,280**	**2,465,610**	**4,486,650**

Total DNA Coding region (b)	1,207,722	1,556,870	2,139,206	3,918,335

Average intergenic region (b)	80	77	129	128

Average gene density (genes/Mbp)	968	903	935	938

G+C content (%)	28.30	26.28	29.65	33.38

tRNA genes	34	39	46	40

Predicted ORFS^1^	1,282	1,511	2,306	4,210

Average ORF size in bp (aa)^2^	942 (314)	1014 (338)	924 (308)	927 (309)

Genes assigned to COGs^3^	1,042	1,111	1,731	2,229

Genes associated with a putative function^4^	852	907	1306	2164

Genes with signal peptides	137	262	432	801

Genes with transmenbrane helices	257	343	530	901

^1^Open reading frames predicted as described in the Materials and Methods;

^2^Average ORFs sizes shown herein were calculated using protein-coding sequences available at GeneBank: *S. moniliformis *(1434 protein sequences), *L. buccalis *(2220 protein sequences) and *S. termiditis *(4083 protein sequences);

^3^Clusters of orthologous groups as described in the Materials and Methods;

^4^Genes assigned to general functional prediction only (Category R) and function unknown (Category S) were not included.

The genome of *Sneathia amnii *is approximately 1.34 Mbp and has a GC content of ~28%. Thus, this organism exhibits the smallest of the Fusobacteriaceae family genomes sequenced to date. A representative from each of the most closely related genera; i.e., *S. moniliformis *(1.67 Mbp), *L. buccalis *(2.47 Mbp), and *S. termiditi*s (4.49 Mbp), was selected for genomic comparisons. The *S. amnii *genome exhibits 1,282 putative protein-coding genes, with an average gene length of 969 bp (323 amino acids), constituting 92% of the genome. Thus, the coding density of the genome is quite high. *S. amnii *intergenic regions have an average length of 80 bp, very similar to the 77 bp average intergenic size of *S. moniliformis*. The genomes of *L. buccalis *and *S. termiditis *have slightly larger intergenic regions, averaging 129 and 128 bp, respectively (Table 1). However, several studies have found no significant correlation between bacterial genome size and average intergenic region lengths [23]. We found that the differences in the gene composition account for the difference in the genome sizes of these related organisms. Although the genome of *S. amnii *is relatively small, its gene density (968 genes/Mbp) is higher than the average gene density of related members of the Fusobacteriaceae family, including *S. moniliformis*, *L. buccalis *and *S. termiditis*. This high gene density of *S. amnii *seems to be attributable to numerous overlapping genes. Our evidence also suggests that the *S. amnii *genome contains at least 110 overlapping genes. The compact genome of this organism supports a hypothesis that it is subject to selective pressures that attribute high cost to genome size and replication.

**Figure 4:**
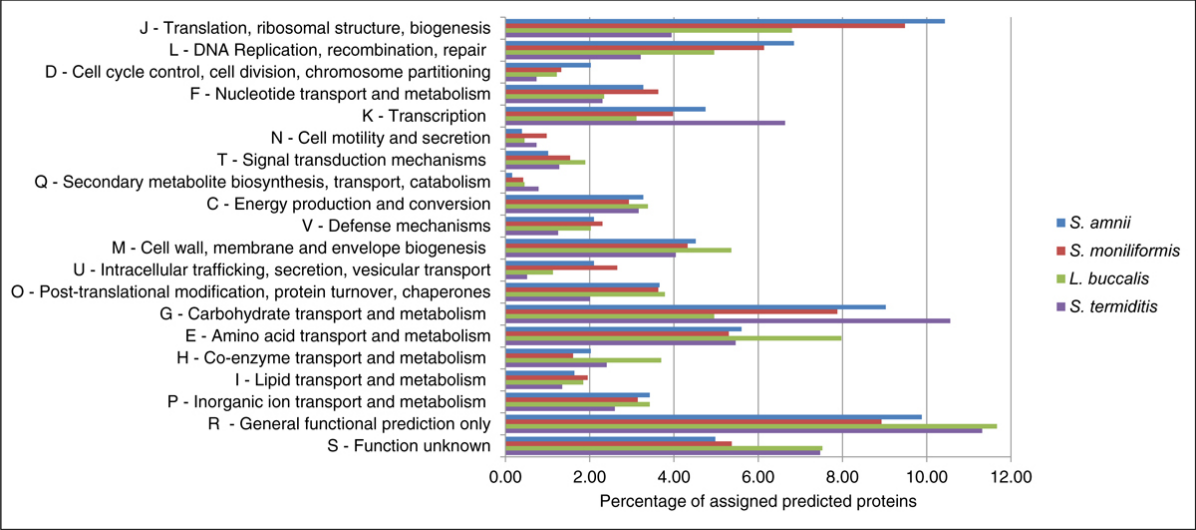
**Comparison of the distribution of predicted proteins of *S. amnii *and related organisms**. Predicted ORFs from *S. amnii*, *S. moniliformis*, *L. buccalis *and *S. termiditis *were classified into COG functional categories. The percent of genes belonging to each COG is represented for each species.

Of the 1,282 predicted genes of *S. amnii*, 852 (~66%) clustered into COG orthologous groups [24] and could be associated with a putative function. A comparison of COG functional groups for the selected Fusobacteriaceae genomes revealed a correlation of gene content with genome size (Figure 4). As observed previously [25], among the four taxa that we examined, gene contents for COG classes J, L, D and F are generally inversely correlated to genome size, whereas classes K, N, T, and Q are generally positively correlated. COG groups J, L, D and F generally deal with DNA replication, cell cycle regulation and protein translation, whereas COG groups K, N, T and Q are associated with transcription, signal transduction, cell motility and the biochemistry of secondary metabolites (Figure 4, and Additional file 2). There is a rationale for this in that as the genome size becomes compressed, functions of cell motility and signaling, which are dispensable or can be 'outsourced' to the host, are lost, while essential functions like DNA replication are maintained. As the total number of genes is reduced, the less important functions become a lower percentage of the remaining genes, and the more important functions become a higher percentage of the total. This trend was also apparent among genes not characterized in COG, which is probably not surprisingly since these genes are likely to be involved in more specialized processes and may not be essential for growth and survival. Interestingly, whereas a positive correlation between genome size and COG class C (energy production and conversion) has previously been reported, we did not observe this correlation in our analysis of these four genomes (Figure 4). Thus, the general characteristics of this genome are fairly typical of a pathogen under pressure to tailor its genetic capabilities to the bare essentials while taking maximum advantage of a close relationship with its mammalian host.

### Central metabolism

Analysis of the metabolic potential of *S. amnii *provided insight into the biochemical reactions underlying the complex growth requirements of this fastidious organism. Our genome-wide metabolic reconstruction analyses suggested that *S. amnii *is able to metabolize a limited variety of carbohydrates, including glucose, maltose, glycogen and glucosamine. In contrast, *Sneathia *would be unable to ferment starch, mucin and mannose. The key enzymes; i.e. hexokinase, fructokinase, galactokinase, mannokinase and rhamnulokinase, needed for these conversions of the latter carbohydrates are missing in the genome of *S. amnii*. Fermentation assays confirmed that, as predicted, *S. amnii *ferments glycogen, maltose, and glucose, but not starch, galactose, mucin, mannose, sucrose or fructose. In addition, lactic acid was produced during fermentative metabolism as predicted by metabolic analyses. Since *S. termiditis*, *L. buccalis *and *S. moniliformis*, which clearly arose from a progenitor common to *S. amnii*, maintain these capabilities, these results support the hypothesis that *S. amnii *sp. nov. has lost many of the capabilities of its progenitors (Additional file 2).

Glycogen is produced by vaginal epithelial cells in women of reproductive age, and many known vaginal colonizers (e.g., the Lactobacillaceae) utilize this carbohydrate source. Therefore, it was not surprising that *S. amnii*, with its "reduced" metabolic capabilities, still effectively utilizes glycogen but can ferment only a few alternative carbon sources. Glycogen and glucose are the most abundant carbohydrate sources in the vagina, but fructose, mannose, glucosamine and starch are also present in lower amounts [26]. Maltose is an intermediate of glycogen metabolism and it is possible that *S. amnii *acquires this nutrient source from other bacteria that share the niche.

*In silico *analysis of the *S. amnii *genome revealed complete phosphotransferase systems (PTSs) for mannose, galactitol and cellobiose. Since we have empirically shown that *S. amnii *cannot metabolize mannose or galactose, the role(s) of these systems is unclear. In contrast, the majority of the sugar transporter PTS systems, including those for beta-glucosides, D-glucosamine, fructose, glucose, lactose, mannitol and sucrose, are incomplete. For these systems, genes for an EIIA component (phosphocarrier protein HPr) are present, but no genes encoding putative permeases were identified. Thus, the functions of these incomplete PTS systems are currently unknown. Some studies suggest that, instead of carbohydrate transport, some of the enzymes in these systems may be involved in regulation of other biochemical pathways [27].

### Gene function

#### Energy metabolism

Genes encoding enzymes of the non-oxidative branch of the pentose phosphate pathway (PPP), including the genes for transaldolase and transketolase, were identified in the genome. These enzymes link the PPP with glycolysis by catalyzing the conversion of dietary 5 carbon sugars into both 6 (fructose-6-phosphate) and 3 (glyceraldehyde-3-phosphate) carbon sugars, which can then be utilized by the pathways of glycolysis. It has been suggested that some human pathogens turn to gluconeogenesis to sustain growth when faced with limited sugar substrates [28]. However, our analyses suggest that *S. amnii *cannot use this strategy, since fructose bisphosphatase, an essential gene for gluconeogenesis, is apparently not present in its genome. Since the organism is anaerobic (see below), although moderately aerotolerant, it was not unexpected that enzymes for oxidative phosphorylation (e.g., fumarate reductase, succinate dehydrogenase) were absent from the genome. Additionally, the tricarboxylic acid (TCA) cycle of *S. amnii *appears to be absent, since only one of its required enzymes, dihydrolipoamide S-succinyltransferase, was detected in the 1.34 Mbp genome of the bacterium.

#### Amino acid and nucleotide biosynthesis

Loss of genes required for biosynthesis of amino acids is common among opportunistic pathogenic bacteria [29], and we found that *S. amnii *also lacks the enzymes required to synthesize most amino acids. In contrast, however, the enzymes needed to convert L-aspartate to fumarate, L-asparagine, and oxaloacetate appear to be present in the genome. In addition, many of the required enzymes that convert L-amino acids to D-amino acids were identified. As for genes required for amino acid biosynthesis, the genes required for *de novo *synthesis of purines and pyrimidines were not identified in the genome of *S. amnii*. However, several components of the salvage pathways for purine and pyrimidine biosynthesis were present. Thus, *S. amnii *apparently relies on the purine nucleotide salvage via adenosine and hypoxanthine. Adenosine is likely to be imported from host cells and converted into inosine by purine nucleoside phosphorylase. Hypoxanthine also appears to be derived from the host cell and then converted to inosine-5'-monophosphate (IMP) by hypoxanthine-guanine phosphoribosyltransferase. IMP serves as the precursor for both AMP and GMP, which are further converted to triphosphates. As part of the purine salvage process, the enzymes purine-nucleoside phosphorylase and xanthine-guanine phosphoribosyltransferase are also encoded in this genome, and they are responsible for the conversion of the xanthosine to either xanthine or guanine to XMP or GMP, respectively. In addition, there are two alternative salvage pathways by which bacteria convert uridine to UMP. One is catalyzed by uracil phosphoribosyltransferase, the other requires the sequential enzymatic reactions of uridine phosphorylase and uridine kinase. Uridine kinase also converts cytidine to CMP. These key components of the pyrimidine salvage pathway are encoded in the *S. amnii *genome. Thus, despite the lack of many enzymes required for amino acid and nucleotide synthesis, *S. amnii *seems to be able to scavenge what it needs from its hosts.

### DNA repair and exchange

A number of genes predicted to encode proteins involved in DNA modification and repair were also detected. Thus, we identified a putative DNA restriction-modification system. There are two genes encoding putative competence related proteins, but a complete system for genetic competence is apparently lacking, since the complex named RecBCD, composed of three different subunits called RecB, RecC, and RecD, was not found. Genes encoding apparent recombination proteins, including RecA, RecG, RecX, RecF, RecR, RuvABC, and viral RecT, were present. The genome contains a *uvrD *homolog, but, like other pathogenic bacterial species with minimal genomes [30], other genes involved in UV-induced DNA repair, including *uvrABC*, were not found and seem to be absent.

The *S. amnii *genome bears a complete temperate prophage genome in addition to several individual phage-related genes dispersed throughout the genome. The prophage consists of a 20,259 kbp sequence containing both a lysogeny module, including putative integrase and repressor genes, and a replicative module, including genes encoding terminase, portal and tail proteins. Other phage-related genes encoding integrases, a replisome organizer and a capsid are distributed throughout the genome. Two identical 1.2 kbp insertion sequences are also present in the *Sneathia *genome. This insertion sequence has 99% identity to IS605, which was previously reported in the BV-associated Clostridiales species BVAB3, suggesting a possible cross genus transmission.

In brief, *S*. *amnii *exhibits the capacity for general DNA recombination and repair and while it does not appear to have the capacity for high-level natural competence, it has clearly acquired exogenous DNA, possibly through phage transduction and/or other mechanisms.

### Transport

Few genes encoding known secretion systems were found; however, several genes predicted to encode homologs of the Type II protein secretion machinery (T2SS) were detected. Normally, components of T2SS are encoded within an operon located at a single genomic locus, although single genomes can contain multiple, discontiguous T2SS [31]. S. *amnii *contains genes homologous to both PulF/PilC and GspD proteins in a single locus. A second locus encodes a GspE homolog, hypothesized to be the ATPase that energizes Type II secretion, and a SecA homolog is located at a third locus. Taken together, these observations suggest that *S. amnii *has functional Sec and Type II secretion systems, although other protein secretion systems that were not identified by our analyses may also be present. In addition, there are many genes apparently related to small molecule transport, including ~40 ABC-type transporter genes, ~13 genes involved in ion transport, and ~7 multidrug/lipid/protein pumps. Apparent homologs of the F_1_F_0 _ATPase were also detected. In brief, *S. amnii *seems to be well equipped to obtain vitamins, cofactors and other nutrients from its environment.

### Growth requirements

*S. sanguinegens *reportedly requires blood for growth [11]. In contrast, *S. amnii *did not require blood for growth, but its growth rate was enhanced by the addition of human serum (not shown). *S. amnii *grew well on chocolate agar. Colonies appeared after 48 h, and by 72 h the colonies were flat, ~1 mm in diameter, and crystalline. *S. amnii *did not grow on Brucella Sheep's blood agar but colonies on BHI agar containing 10% fresh human blood were mucoid, raised, amorphous, ~2 mm in diameter, and displayed alpha hemolytic activity. Consistent with our predictions from the genome analysis, *S. amnii *was catalase-negative and grew only under anaerobic conditions. However, it was able to tolerate transient exposure to air and was positive for superoxide dismutase activity. Interestingly, the genome did not contain an obvious gene encoding superoxide dismutase, and maximum identity to superoxide dismutase proteins, as detected using blastx, was 30%.

### Morphology

**Figure 5:**
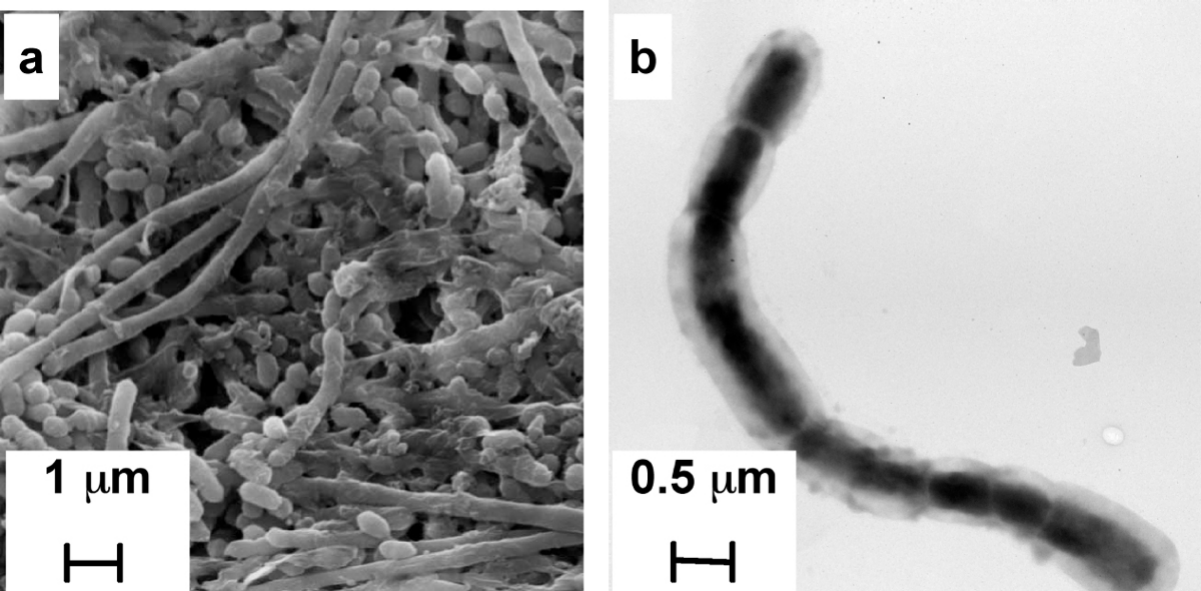
**Electron Micrographs of *S. amnii***. *S. amnii *were fixed to either glass cover slips, or copper grids, and visualized by SEM (a) or TEM (b), respectively.

Scanning electron microscopy of *S. amnii *(Figure 5a) revealed variable morphology including long (10+ μm) gram-negative rods as well as short, amorphous rods and cocci. Similar short morphotypes have been reported for *S*. *moniliformis *[32], and other bacteria, and are referred to as "L forms". The L forms of *S. moniliformis *are reportedly deficient in cell walls and thought to be non-pathogenic. *S. amnii *L-forms were more common in older cultures, although actively growing cultures were predominated by the bacillary form. Tandem electron microscopy revealed that the long rods were actually chains of 1-2 μm long bacilli with rounded ends (Figure 5b). *S. amnii *did not appear to be motile, the genome does not contain genes predicted to encode pilin or flagellar proteins, and neither SEM nor TEM revealed structures that resemble pili or flagella.

### Antibiotic sensitivity

We performed sensitivity assays for a number of antibiotics (Table 2). *S. amnii *displayed high resistance to nafcillin, as is common for other gram-negative organisms. The standard therapy for BV is metronidazole, but this therapy is suboptimal, and it is not known whether all BV-associated organisms are sensitive to this agent. Interestingly, *S. amnii *was more sensitive than *Gardnerella vaginalis*, another common BV-associated organism, to metronidazole. In contrast, *S. amnii *was more resistant than *G. vaginalis *to every other drug tested [33]. Gram-negative bacteria are generally resistant to the glycopeptide, vancomycin, because the large compound is unable to pass through the gram-negative outer membrane. In fact, vancomycin sensitivity is often used as an alternative test to gram staining and more than 99% of gram-negative clinical bacterial isolates, including *Leptrotrichia *species, are vancomycin resistant [34,35]. Thus, it was surprising that *S. amnii *was vancomycin-sensitive. This finding may suggest that the cell envelope of *S. amnii *differs from that of typical gram-negative bacteria, or it may be related to the large number of permeases and transporters predicted to be within the *S. amnii *envelope. Finally, *S. amnii *exhibited relatively high levels of resistance to antibiotics such as tetracycline (> 50 μg/ml) and ciprofloxacin (> 25 μg/ml). The genome encodes two apparent MATE efflux family homologs. Members of this family are frequently involved in drug export and single or multi-drug resistance and could be involved in resistance to these antimicrobial agents.

**Table 2:** **Sensitivity of *S. amnii *to antibiotics**.

Antibiotic	MIC (μg/ml)
Ampicillin	9.8

Nafcillin	1.25

Ciprofloxacin	25

Nalidixic acid	312

Chloramphenicol	1.25

Minocycline	0.97

Tetracycline	> 50

Erythromycin	> 10

Clindamycin	5

Kanamycin	6

Rifampin	0.4

Metronidazole	0.5

Vancomycin	1.0

Although BV is associated with a greater than 2-fold increased risk for preterm birth, treatment with metronidazole during pregnancy does not reduce the risk for preterm birth [36]. BV involves the formation of a bacterial biofilm on the vaginal epithelium and the bacteria within these biofilms appear to be recalcitrant to metronidazole therapy, leading to a high rate of relapse [37]. Thus, even though *S. amnii *was sensitive to metronidazole, it may exhibit antibiotic tolerance if it occurs within the BV-associated biofilm. Therapeutic intervention strategies that target both the biofilm and the causative species may need to be developed in order to reduce the rate of BV-associated preterm birth.

### Hemolytic activity and cytotoxicity

**Figure 6:**
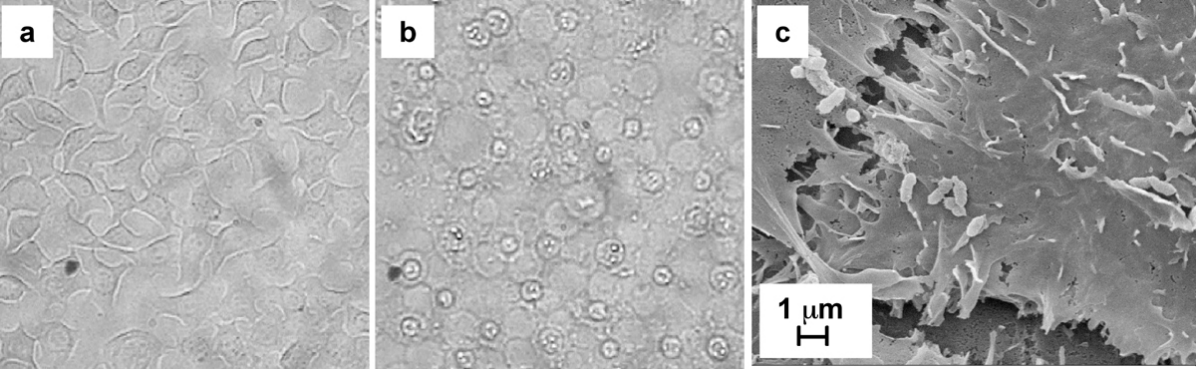
***S. amnii *was cytotoxic and adhered to Me180 cells**. To assess cytotoxicity, bacteria were added to Me180 monolayers and monitored by light microscopy during the incubation for phenotypic alterations. In stark contrast to monolayers treated with PBS alone as a control (a), monolayers exposed to *S. amnii *rapidly induced cell rounding and loss of adherence (b). To determine whether or not *S. amnii *adhered, bacteria were added to Me180 monolayers grown on glass coverslips. Following incubation, the glass coverslips were washed extensively in PBS, stained, and analyzed by SEM (c).

Consistent with the observation of alpha hemolysis on blood agar, *S. amnii *exhibited weak hemolytic activity. *S. amnii *induced the release of ~9% of the hemoglobin from human red blood cells while an *E. coli *strain without hemolytic activity lead to the release of less than 0.5%. The genome encodes a putative hemolysin of approximately 28 kDa that could be responsible for the hemolytic activity. It also encodes a large protein (161 kDa) with homology to both hemagglutinins and hemolysins. However, hemagglutination of human red blood cells was not observed. *S. amnii *was cytotoxic to ME-180 human cervical cancer cells. Thus, after 2 hours of exposure, the cervical epithelial cells exhibited rounding and other cytopathic changes associated with cytotoxicity (Figure 6a, b), suggesting the existence of a toxin that causes damage to these cells. Although a cytotoxin was not annotated in the genome, one of the hemolysins discussed above could be responsible for this activity.

### Adherence to cervical epithelial cancer cells

*S. amnii *adhered to ME-180 human cervical cancer cells (Figure 6c). The image also reveals apparent perforations in the epithelial cells near the site of bacterial adherence that were not present in untreated cells (not shown), suggesting that the bacteria may be causing local membrane damage. The duration of the adherence was minimized to prevent major cytopathogenic changes; however, it is likely that cytotoxic effects were starting to manifest. The *S. amnii *genome contains only one apparent adhesin homolog, but three apparent homologs of the Hia family cell surface protein and a number of YadA domain-containing proteins are present [38,39]. The Hia and YadA families are autotransporters often implicated in adherence to host tissues and cells. There is also a gene encoding a putative fibronectin-binding protein, which could play a role in adherence to host tissues.

### Other genetic components with potential roles in virulence

We did not detect any putative quorum-sensing systems. However, only a few systems including the LuxS system are highly conserved and it is possible if not likely that a quorum-sensing system would be difficult to detect. A putative toxin-antitoxin cassette was found. These cassettes can play a role in the stabilization of foreign DNA, such as plasmids [40]. They have also been shown to contribute to stress resistance and the persister phenotype [41]. Sneathia has been isolated from amniotic fluid and therefore presumably has the capacity to invade the uterine cavity and traverse the fetal membranes.

Very little is known about virulence factors that promote uterine and intra-amniotic invasion although it has been speculated that enzymes including sialidase that hydrolyze sialic acid residues or enzymes that degrade sialylated proteins plays a role in traversal of the cervical mucus [42]. *Sneathia *encoded a protein with 63% identity and 77% similarity to a O-sialoglycoprotein endopeptidase that could be involved in the degradation of sialylated proteins. Evidence suggests that *Listeria *crosses the gestational membranes through villous syncytiotrophoblasts, and that the invasin, internalin is required for this process [43]. *Sneathia *encoded a number of potential invasins. The YadA-like surface protein mentioned as a putative adhesin has also been implicated in invasion. It also encodes a protein with 22% identity and 37% similarity to internalin F from *Clostridium perfringens*.

Clustered regularly interspaced short palindromic repeats (CRISPR) are hypervariable sequences that are widely distributed in bacteria and archaea and play critical roles in the adaptation and persistence of a microbial host in a particular ecosystem by providing acquired resistance against viruses [44]. Using CRISPFinder [45] we identified one CRISPR locus with nine 36 bp direct repeats (DR) located adjacent to the putative *cas *gene. These finding suggest the presence of a functional CRISPR system in *S. amnii*.

## Conclusions

*Sneathia *is one of the most frequently detected organisms from amniotic fluid in cases of preterm labor, suggesting an important role in obstetric health. We detect *Sneathia *species in over 40% of mid-vaginal samples in our ongoing study. The fastidious nature of *Sneathia *makes cultivation somewhat difficult, and consequently, very little is known about its biology or its pathogenic potential. The first genomic sequence of an organism in the genus *Sneathia *provides a starting point for the detailed study of these organisms and their role in obstetric and gynecologic health. The genome is small, approximately 1.34 Mbp, and highly reduced, which may be the basis of its fastidious nature. Metabolic reconstructions of the genome revealed capacities that largely reflect the known metabolic, energy, and anaerobic phenotypes of the bacterium. We did observe an increased growth phenotype in the presence of human serum, which was not reproducible using fetal bovine serum (data not shown), reaffirming the highly specialized nature of this organism for the human niche. Our results indicate that *S. amnii *is able to adhere to, and has high cytotoxic potential for, cervical epithelial cells. Analysis of the genome sequence revealed a hemolysin, which could exert this cytotoxic activity. While *S. amnii *was sensitive to metronidazole in our assay, BV-associated biofilms may confer antibiotic tolerance to species that appear sensitive in vitro and have been shown to be involved in the high rate of relapse. In summary, the genome of this emerging pathogen reflects its phenotype and begins to shed light on the reveal its pathogenic potential and likely role in the gynecologic and obstetric complications associated with *S. amnii*.

### Description of *Sneathia amnii *sp.nov

*Sneathia amnii *(am'ni.i. Gr. n. *amnion*, inner membrane surrounding the fetus; N.L. gen. n. *amnii*, of the amnion, pertaining to the amniotic fluid from which the organism was first isolated) consists of long gram-negative rods as well as short, amorphous rods and cocci. Closer examination suggests that the long rods are chains of short rod-like bacteria. Colonies on blood agar plates after 72 hours were flat, ~1 mm in diameter, and crystalline. Colonies on BHI agar containing 10% fresh human blood were ~2 mm in diameter and displayed alpha hemolytic activity. Cells were catalase-negative, aerotolerant, and optimal growth occurred under anaerobic conditions. Fermentation of glycogen, maltose, and glucose were demonstrated. Lactic acid was produced during fermentative metabolism. Strains have been isolated from human clinical specimens (mid-vaginal wall, amniotic fluid). The type strain of *S. amnii*, Sn35, has a %G+C of ~28% and a genome of ~1.34 Mbp.

## Methods

### Subject recruitment

We enrolled adult women from outpatient women's health clinics in Virginia as part of the Vaginal Human Microbiome Project [21]. Incarcerated women and women who were not scheduled for a vaginal examination were excluded from the study. Enrollment occurred between August 2009 and March 2011. Written consent was obtained from all study participants and the institutional review boards at Virginia Commonwealth University and the Virginia Department of Health approved the study. Clinicians obtained swab samples from the mid-vaginal wall during a speculum examination.

### Metagenomic 16S rRNA gene identification of *Sneathia *in mid-vaginal samples

DNA was extracted within four hours of collection using the MoBio PowerSoil DNA processing protocol. The V1-V3 region of the 16S rRNA gene was PCR-amplified with bar-coded primers and sequenced on a Roche 454 FLX Titanium Genome Sequencer as described in [21].

### Isolation and growth conditions

Bacteria from a swab sample taken from the mid-vaginal wall of a female volunteer were cultured on chocolate agar at 37°C for 72 h using the AnaeroPack system (Mitsubishi Gas Chemical Co, Tokyo, Japan). Single colonies were isolated and 'colony' PCR was performed using 16S rDNA-specific primers UnivFwd (AGAGTTTGATCCTGGCTCAG) and UnivRev (5'- GGACTACCAGGGTATCTAAT -3') [46] and PCR Supermix HiFi (Invitrogen). PCR conditions were 94°C 2 min followed by 35 cycles of 94°C 30 s, 51°C 30 s, and 72°C 30 s. PCR products were sequenced and species identification was based on identity with 16S rDNA sequences in the NCBI database. Following initial isolation, *S. amnii *was grown on chocolate agar or in Brain Heart Infusion (BHI) broth (EMD, Gibbstown, NJ) supplemented with 1% yeast extract, 2% gelatin, 0.1% starch, 0.1% glucose, and 5% human serum (sBHI) at 37°C under anaerobic conditions. To analyze carbohydrate fermentation, 5% human serum, 0.002% phenol red, and 1% sugar (glucose, starch, maltose, galactose, glycogen, mucin, mannose, sucrose, or fructose) were added to chemically defined vaginal fluid medium (described in [47]), 200 μL of the medium was aliquoted into 96 well plates, and inoculated with *S. amnii*. The plates were incubated anaerobically and inspected visually at 24 h and 48 h for signs of growth (turbidity) and acid production (change in media color from red to yellow). Lactic acid was measured using the Lactate Assay Kit (Biovision, Mountain View, Ca) according to manufacturer's instructions.

### Superoxide dismutase activity

Approximately 2 × 10^9 ^bacteria were resuspended in 500 μL Tris EDTA buffer (TE) and lysed with 500 μL of 1 μm glass beads twice at power 6.5 for 30 s, in a Fastprep cell disrupter (Thermo). Protein concentration was determined by measuring A_260 nm _on a Nanodrop™ spectrophotmeter. Superoxide Dismutase activity was measured using the Superoxide Dismutase Assay Kit (Cayman Chemical Company, Ann Arbor, MI). Briefly, 10 μL of lysate were added to 200 μL of the diluted radical detector, the reaction was initiated by addition of 20 μL diluted xanthine oxidase and incubated at room temperature for 20 min with gentle mixing. Units of activity were calculated by comparing the A_450 nm _to the standard provided by the manufacturer.

### DNA isolation and sequencing

*S. amnii *was grown in 20 mL sBHI overnight. The cells were collected by centrifugation, and DNA was isolated using the Genomic-tip 500/G (Qiagen) according to manufacturer's instructions. Genome sequencing of *S. amnii *was performed with a combined strategy using whole genome shotgun and 8-kilobase pair (kbp) paired-end reads. For the shotgun library, fifty nanograms of DNA were used in a tagmentation reaction with a Nextera™ DNA Sample Prep Kit (Roche Titanium-compatible, Epicentre Biotechnologies) following the manufacturer's protocol. For the paired end library, genomic DNA was fragmented into 8-kbp fragments using our HydroShear™ DNA Shearing Device (GeneMachines, Inc.). Further paired-end library preparation was performed according to the manufacturer's protocols (Roche). The genomic libraries of *S. amnii *were sequenced on the Roche 454 FLX Titanium system in the Nucleic Acids Research Facilities at VCU. A total of 583,691 shotgun reads and 287,309 paired-end reads yielded a ~247-fold coverage of the genome. The reads were assembled using Newbler™ version 2.0.00.20 software (Roche) using default parameters. The final assembly generated a single circular scaffold containing the entire genome. Closure of physical gaps was performed by PCR amplification using primers targeted to contigs flanking the gaps, followed by fluorescent chain termination sequence analysis on AB3730 or AB3130 capillary sequencers (Applied Biosystems).

### Gene calling and analysis

Genes were called using Glimmer 3 [48] using default parameters. Transfer RNA genes were predicted using tRNAscan-SE 1.23 [49] and ribosomal RNA genes were found by similarity search. Sequences were initially annotated by comparison with currently annotated bacterial sequences present in NCBI's NR protein database. Metabolic reconstruction and Gene Ontology classification assignments were performed using ASGARD software [50] in conjunction with the UniRef100 database [51]. Other annotation features were predicted as follows: transmembrane domains by TMHMM 2.0c [52]; signal peptides by SignalP 3.0b [53]; COG similarities and Pfam domain composition by rpsblast [24]

### Phylogenetic analysis

The 16S rDNA sequences of *S. amnii *and 31 related organisms from Fusobacteriaceae family were aligned using the ClustalW program [54] and the alignments were corrected by visual inspection. Phylogenetic analysis was performed from 1,271 aligned characters of the 16S rRNA sequences under the maximum likelihood criterion [55]. Maximum likelihood trees were inferred using PhyML 3.0 program [56,57] using the HKY85 model, gamma shape parameter and proportion of invariable sites. Model parameters were estimated in PhyML over the duration of the tree search. The numbers at the nodes are the result of a PhyML bootstrap analysis.

### Antibiotic sensitivity assay

Antibiotics were serially diluted 2-fold in 200 μL sBHI in mictotiter wells and 5 μL of a 48 h bacterial culture was added to each well. The microtiter plates were incubated anaerobically for 48 h and the lowest concentrations of antibiotics that prevented visible bacterial growth were recorded.

### Electron microscopy

For tandem electron microscopy, bacteria grown in sBHI were collected by centrifugation, washed in sterile deionized water, spotted onto formvar-coated 200-mesh copper grids (Electron Microscopy Sciences, Hatfield, PA), stained with 2% phosphotungstic acid, and analyzed using a Jeol JEM-1230 transmission electron microscope equipped with a Gatan UltraScan 4000SP 4K × 4K CCD camera. For scanning electron microscopy, washed bacteria were fixed in 2% gluteraldehyde. To visualize adherence to cervical epithelial cells, washed bacteria were added to ME-180 human cervical cancer cell monolayers grown on poly-lysine-coated glass coverslips, incubated for 5 min, and the monolayers were washed 3 times with 1× PBS to remove non-adherent bacteria. The monolayers were fixed in 2% gluteraldehyde. All samples were then rinsed, fixed in 1% osmium tetraoxide, rinsed, and then dehydrated with washes of increasing concentrations of ethanol followed by hexamethadisilizane (HMDS), mounted, and coated with gold using an EMS 550 Sputter Coater (Electron Microscopy Sciences, Hatfield, PA) just prior to viewing. Samples were analyzed using a Zeiss EVO50XVP Scanning Electron Microscope (Carl Zeiss SMT, Inc., Peabody, MA).

### Hemolysis and hemagglutination

Fresh human blood collected with EDTA as an anticoagulant was centrifuged at 500 × g for 10 min. The serum was removed, the red blood cells (RBC) were washed once in 10 volumes of phosphate buffered saline (PBS), and resuspended in 10 volumes of fresh PBS. 100 μL of the RBC were added to 0.5 mL microfuge tubes. Approximately 1 × 10^6 ^bacteria from 24 h liquid cultures of *S. amnii *or *E. coli *(negative control) were washed with PBS and added to the RBC-containing microfuge tubes. PBS was used as a negative control. The tubes were incubated, stationary, for 30 min at 37°C and observed visually for lattice formation (hemagglutination). The tubes were centrifuged at 500 × g for 10 min, the supernatant was collected, and the A_450 _was determined as a measure of hemoglobin release. RBCs lysed in pure water were used as a positive control for 100% hemolysis. Bacteria-induced hemolysis was calculated as the percentage relative to the positive control as 100% [(A_450_Bacteria - A_450_PBS)/A_450 _water].

### Cytotoxicity

*S. amnii *was cultured in sBHI, collected by centrifugation, and resuspended in PBS to an OD_600 _= 1.0. Two-fold serial dilutions were made. ME-180 human cervical cancer cells were cultured at 37°C in 5% CO_2 _in McCoy's 5A medium (Quality Biologic, Gaithersburg, MD) supplemented with 10% fetal bovine serum and 1 IU mL^-1 ^penicillin/streptomycin (MediaTech, Manassass, VA) in 96 well plates. Once the cells reached ~90% confluence, the media was replaced with 100 μL PBS, and 100 μL of each bacterial dilution was added. The monolayers were monitored every hour by light microscopy for cytopathogenic changes, such as cell rounding, loss of adhesion, and disruption of the monolayer. Photos were taken using an Olympus CK2 light microscope at magnifications of 100× and 400×.

## Competing interests

The authors declare that they have no competing interests.

## Authors' contributions

MDH isolated *S. amnii*, performed biochemical, cytotoxicity, adhesion, and antibiotic sensitivity assays, performed electron microscopic analysis and wrote the manuscript.

MGS performed sequence analysis, assembly, annotation, metabolic reconstruction, phylogenetic analysis, and wrote the manuscript.

JMF coordinates the Vaginal Human Microbiome Project, speciated *Sneathia *in the microbiome dataset, analyzed *Sneathia *carriage rates, contributed to phylogenetic analysis and interpretation of metabolic reconstruction, and wrote the manuscript.

JMA contributed to sequence analysis, assisted with the metabolic reconstructions, and edited the manuscript.

MAR assisted in identifying the importance of *Sneathia *in the vaginal microbiome.

Vaginal Microbiome Consortium members contributed to the design and execution of the study, provided creative feedback, and added to the collective interpretation of the relevance of the results to vaginal health.

GAB conceived the research plan, oversaw the project, directed the analysis pipelines, set research priorities, provided creative interpretation of the results, and assisted in the content and writing of the manuscript.

KKJ designed the research, supervised the microbiological and biochemical analyses, interpreted the data, and assisted in the content and writing of the manuscript.

All authors have read and approved the final version of the manuscript.

## Authors' details

All authors are members of the Vaginal Microbiome Consortium. Additional members of the Vaginal Microbiome Consortium who contributed to this study are listed in alphabetical order: James P. Brooks, Christopher J. Friedline, Philippe H. Girerd, Stephanie L. Hendricks, Vladimir Lee, Melissa A. Prestosa, Federico A. Puma, Maria C. Rivera, Nihar U. Sheth, Jerome F. Strauss III, and Logan J. Voegtly.

## Supplementary Material

Additional file 1 **Supplementary figure S1 - Multiple sequence alignment of 16S rDNA from *S. amnii *and related organisms**. Alignment of 16S rDNA sequences of *S. amnii *and representative strains belonging to seven representants of the Fusobacteriaceae family: *Sneathia*, *Streptobacillus*, *Leptotrichia*, *Sebaldella*, *Propionigenium*, *Ilyobacter*, and *Fusobacterium*. Click here for file

Additional file 2 **Supplementary table 1 - Fraction of genes associated with specific COG functional groups in each species**. Click here for file

Additional file 3 **Supplementary table 2 - In silico reconstruction of the metabolic pathways of *S. amnii*, *S. moniliformis*, *L. buccalis *and *S. termiditis***. Click here for file

## References

[B1] Collins MD Hoyles L Tornqvist E von Essen R Falsen E Characterization of some strains from human clinical sources which resemble "*Leptotrichia sanguinegens*": description of *Sneathia sanguinegens *sp. nov., gen. nov Syst Appl Microbiol 2001 24 3 358 361 10.1078/0723-2020-00047 11822670

[B2] Goto M Hitomi S Ishii T Bacterial arthritis caused by *Leptotrichia amnionii* J Clin Microbiol 2007 45 6 2082 2083 10.1128/JCM.02441-06 17392444 PMC1933078

[B3] Shukla SK Meier PR Mitchell PD Frank DN Reed KD *Leptotrichia amnionii *sp. nov., a novel bacterium isolated from the amniotic fluid of a woman after intrauterine fetal demise J Clin Microbiol 2002 40 9 3346 3349 10.1128/JCM.40.9.3346-3349.2002 12202577 PMC130742

[B4] Eribe ER Paster BJ Caugant DA Dewhirst FE Stromberg VK Lacy GH Olsen I Genetic diversity of *Leptotrichia *and description of *Leptotrichia goodfellowii *sp. nov., *Leptotrichia hofstadii *sp. nov., *Leptotrichia shahii *sp. nov. and *Leptotrichia wadei *sp. nov Int J Syst Evol Microbiol 2004 54 Pt 2 583 592 15023979 10.1099/ijs.0.02819-0

[B5] Boennelycke M Christensen JJ Arpi M Krause S *Leptotrichia amnionii *found in septic abortion in Denmark Scand J Infect Dis 2007 39 4 382 383 10.1080/00365540601053022 17454911

[B6] Thilesen CM Nicolaidis M Lokebo JE Falsen E Jorde AT Muller F *Leptotrichia amnionii*, an emerging pathogen of the female urogenital tract J Clin Microbiol 2007 45 7 2344 2347 10.1128/JCM.00167-07 17522272 PMC1933011

[B7] Goldenberg RL Culhane JF Infection as a cause of preterm birth Clin Perinatol 2003 30 4 677 700 10.1016/S0095-5108(03)00110-6 14714919

[B8] Han YW Shen T Chung P Buhimschi IA Buhimschi CS Uncultivated bacteria as etiologic agents of intra-amniotic inflammation leading to preterm birth J Clin Microbiol 2009 47 1 38 47 10.1128/JCM.01206-08 18971361 PMC2620857

[B9] DiGiulio DB Romero R Amogan HP Kusanovic JP Bik EM Gotsch F Kim CJ Erez O Edwin S Relman DA Microbial prevalence, diversity and abundance in amniotic fluid during preterm labor: a molecular and culture-based investigation PLoS One 2008 3 8 e3056 10.1371/journal.pone.0003056 18725970 PMC2516597

[B10] DiGiulio DB Gervasi M Romero R Mazaki-Tovi S Vaisbuch E Kusanovic JP Seok KS Gomez R Mittal P Gotsch F Microbial invasion of the amniotic cavity in preeclampsia as assessed by cultivation and sequence-based methods J Perinat Med 2010 38 5 503 513 20482470 10.1515/JPM.2010.078 PMC3325506

[B11] Hanff PA Rosol-Donoghue JA Spiegel CA Wilson KH Moore LH *Leptotrichia sanguinegens *sp. nov., a new agent of postpartum and neonatal bacteremia Clin Infect Dis 1995 20 Suppl 2 S237 239 7548563 10.1093/clinids/20.supplement_2.s237

[B12] De Martino SJ Mahoudeau I Brettes JP Piemont Y Monteil H Jaulhac B Peripartum bacteremias due to *Leptotrichia amnionii *and *Sneathia sanguinegens*, rare causes of fever during and after delivery J Clin Microbiol 2004 42 12 5940 5943 10.1128/JCM.42.12.5940-5943.2004 15583348 PMC535221

[B13] Bachy B Bemer P Tortellier L Giraudeau C Reynaud A Corvec S First case of septic arthritis due to *Sneathia *species most closely related to *S. sanguinegens* J Med Microbiol 2011 10.1099/jmm.0.027458-0 21737545

[B14] Koumans EH Sternberg M Bruce C McQuillan G Kendrick J Sutton M Markowitz LE The prevalence of bacterial vaginosis in the United States, 2001-2004; associations with symptoms, sexual behaviors, and reproductive health Sex Transm Dis 2007 34 11 864 869 10.1097/OLQ.0b013e318074e565 17621244

[B15] Srinivasan S Fredricks DN The human vaginal bacterial biota and bacterial vaginosis Interdiscip Perspect Infect Dis 2008 2008 750479 19282975 10.1155/2008/750479 PMC2648628

[B16] Fredricks DN Fiedler TL Thomas KK Oakley BB Marrazzo JM Targeted PCR for detection of vaginal bacteria associated with bacterial vaginosis J Clin Microbiol 2007 45 10 3270 3276 10.1128/JCM.01272-07 17687006 PMC2045326

[B17] Haggerty CL Totten PA Ferris M Martin DH Hoferka S Astete SG Ondondo R Norori J Ness RB Clinical characteristics of bacterial vaginosis among women testing positive for fastidious bacteria Sex Transm Infect 2009 85 4 242 248 10.1136/sti.2008.032821 19004865 PMC2708344

[B18] Ling Z Kong J Liu F Zhu H Chen X Wang Y Li L Nelson KE Xia Y Xiang C Molecular analysis of the diversity of vaginal microbiota associated with bacterial vaginosis BMC Genomics 2010 11 488 10.1186/1471-2164-11-488 20819230 PMC2996984

[B19] Nelson DE Van Der Pol B Dong Q Revanna KV Fan B Easwaran S Sodergren E Weinstock GM Diao L Fortenberry JD Characteristic male urine microbiomes associate with asymptomatic sexually transmitted infection PLoS One 2010 5 11 e14116 10.1371/journal.pone.0014116 21124791 PMC2991352

[B20] Nawrot R Kamieniarz K Malinowska M Jozefiak A Kedzia W Kwasniewska A Kuzma D Gozdzicka-Jozefiak A The prevalence of *Leptotrichia amnionii *in cervical swabs of HPV positive and negative women Eur J Gynaecol Oncol 2010 31 4 425 428 20882886

[B21] Fettweis J Alves J Borzelleca J Brooks J Friedline C Gao Y Gao X Girerd P Harwich M Hendricks S The Vaginal Microbiome: Disease, Genetics and the Environment Nature Preceedings 2011 http://dx.doi.org/10.1038/npre.2011.5150.2

[B22] Gaastra W Boot R Ho HT Lipman LJ Rat bite fever Vet Microbiol 2009 133 3 211 228 10.1016/j.vetmic.2008.09.079 19008054

[B23] Molina N van Nimwegen E Universal patterns of purifying selection at noncoding positions in bacteria Genome Res 2008 18 1 148 160 18032729 10.1101/gr.6759507 PMC2134783

[B24] Altschul SF Madden TL Schaffer AA Zhang J Zhang Z Miller W Lipman DJ Gapped BLAST and PSI-BLAST: a new generation of protein database search programs Nucleic Acids Res 1997 25 17 3389 3402 10.1093/nar/25.17.3389 9254694 PMC146917

[B25] Bentley SD Parkhill J Comparative genomic structure of prokaryotes Annu Rev Genet 2004 38 771 792 10.1146/annurev.genet.38.072902.094318 15568993

[B26] Rajan N Cao Q Anderson BE Pruden DL Sensibar J Duncan JL Schaeffer AJ Roles of glycoproteins and oligosaccharides found in human vaginal fluid in bacterial adherence Infect Immun 1999 67 10 5027 5032 10496874 10.1128/iai.67.10.5027-5032.1999 PMC96849

[B27] Barabote RD Saier MH Jr Comparative genomic analyses of the bacterial phosphotransferase system Microbiol Mol Biol Rev 2005 69 4 608 634 10.1128/MMBR.69.4.608-634.2005 16339738 PMC1306802

[B28] Alteri CJ Smith SN Mobley HL Fitness of *Escherichia coli *during urinary tract infection requires gluconeogenesis and the TCA cycle PLoS Pathog 2009 5 5 e1000448 10.1371/journal.ppat.1000448 19478872 PMC2680622

[B29] Yu XJ Walker DH Liu Y Zhang L Amino acid biosynthesis deficiency in bacteria associated with human and animal hosts Infect Genet Evol 2009 9 4 514 517 10.1016/j.meegid.2009.02.002 19460317 PMC2723833

[B30] Gil R Silva FJ Pereto J Moya A Determination of the core of a minimal bacterial gene set Microbiol Mol Biol Rev 2004 68 3 518 537 10.1128/MMBR.68.3.518-537.2004 15353568 PMC515251

[B31] Cao TB Saier MH Jr The general protein secretory pathway: phylogenetic analyses leading to evolutionary conclusions Biochim Biophys Acta 2003 1609 1 115 125 10.1016/S0005-2736(02)00662-4 12507766

[B32] Dienes L The morphology of the L1 of Klieneberger and its relationship to *Streptobacillus moniliformis* J Bacteriol 1947 54 2 231 237 16561353 10.1128/jb.54.2.231-237.1947 PMC526541

[B33] Harwich M Alves J Buck G Strauss J Patterson J Oki A Girerd P Jefferson K Drawing the line between commensal and pathogenic *Gardnerella vaginalis *through genome analysis and virulence studies BMC Genomics 2010 in press 10.1186/1471-2164-11-375 PMC2890570 20540756

[B34] Arthi K Appalaraju B Parvathi S Vancomycin sensitivity and KOH string test as an alternative to gram staining of bacteria Indian J Med Microbiol 2003 21 2 121 123 17642996

[B35] Halebian S Harris B Finegold SM Rolfe RD Rapid method that aids in distinguishing Gram-positive from Gram-negative anaerobic bacteria J Clin Microbiol 1981 13 3 444 448 6165736 10.1128/jcm.13.3.444-448.1981 PMC273811

[B36] Carey JC Klebanoff MA Hauth JC Hillier SL Thom EA Ernest JM Heine RP Nugent RP Fischer ML Leveno KJ Metronidazole to prevent preterm delivery in pregnant women with asymptomatic bacterial vaginosis. National Institute of Child Health and Human Development Network of Maternal-Fetal Medicine Units N Engl J Med 2000 342 8 534 540 10.1056/NEJM200002243420802 10684911

[B37] Swidsinski A Mendling W Loening-Baucke V Swidsinski S Dorffel Y Scholze J Lochs H Verstraelen H An adherent *Gardnerella vaginalis *biofilm persists on the vaginal epithelium after standard therapy with oral metronidazole Am J Obstet Gynecol 2008 198 1 97 e91-96 18005928 10.1016/j.ajog.2007.06.039

[B38] St Geme JW Cutter D The *Haemophilus influenzae *Hia adhesin is an autotransporter protein that remains uncleaved at the C terminus and fully cell associated J Bacteriol 2000 182 21 6005 6013 10.1128/JB.182.21.6005-6013.2000 11029419 PMC94733

[B39] Tertti R Skurnik M Vartio T Kuusela P Adhesion protein YadA of *Yersinia *species mediates binding of bacteria to fibronectin Infect Immun 1992 60 7 3021 3024 1612772 10.1128/iai.60.7.3021-3024.1992 PMC257272

[B40] Bukowski M Rojowska A Wladyka B Prokaryotic toxin-antitoxin systems--the role in bacterial physiology and application in molecular biology Acta Biochim Pol 2011 58 1 1 9 21394325

[B41] Wang X Wood TK Toxin-antitoxin systems influence biofilm and persister cell formation and the general stress response Appl Environ Microbiol 2011 77 16 5577 5583 10.1128/AEM.05068-11 21685157 PMC3165247

[B42] Wiggins R Hicks SJ Soothill PW Millar MR Corfield AP Mucinases and sialidases: their role in the pathogenesis of sexually transmitted infections in the female genital tract Sex Transm Infect 2001 77 6 402 408 10.1136/sti.77.6.402 11714935 PMC1744407

[B43] Lecuit M Nelson DM Smith SD Khun H Huerre M Vacher-Lavenu MC Gordon JI Cossart P Targeting and crossing of the human maternofetal barrier by Listeria monocytogenes: role of internalin interaction with trophoblast E-cadherin Proc Natl Acad Sci USA 2004 101 16 6152 6157 10.1073/pnas.0401434101 15073336 PMC395938

[B44] Karginov FV Hannon GJ The CRISPR system: small RNA-guided defense in bacteria and archaea Mol Cell 2010 37 1 7 19 10.1016/j.molcel.2009.12.033 20129051 PMC2819186

[B45] Grissa I Vergnaud G Pourcel C CRISPRFinder: a web tool to identify clustered regularly interspaced short palindromic repeats Nucleic Acids Res 2007 35 Web Server W52 57 17537822 10.1093/nar/gkm360 PMC1933234

[B46] Wilson KH Blitchington RB Greene RC Amplification of bacterial 16S ribosomal DNA with polymerase chain reaction J Clin Microbiol 1990 28 9 1942 1946 2095137 10.1128/jcm.28.9.1942-1946.1990 PMC268083

[B47] Geshnizgani AM Onderdonk AB Defined medium simulating genital tract secretions for growth of vaginal microflora J Clin Microbiol 1992 30 5 1323 1326 1583140 10.1128/jcm.30.5.1323-1326.1992 PMC265277

[B48] Salzberg SL Pertea M Delcher AL Gardner MJ Tettelin H Interpolated Markov models for eukaryotic gene finding Genomics 1999 59 1 24 31 10.1006/geno.1999.5854 10395796

[B49] Lowe TM Eddy SR tRNAscan-SE: a program for improved detection of transfer RNA genes in genomic sequence Nucleic Acids Res 1997 25 5 955 964 9023104 10.1093/nar/25.5.955 PMC146525

[B50] Alves JM Buck GA Automated system for gene annotation and metabolic pathway reconstruction using general sequence databases Chem Biodivers 2007 4 11 2593 2602 10.1002/cbdv.200790212 18027373

[B51] Suzek BE Huang H McGarvey P Mazumder R Wu CH UniRef: comprehensive and non-redundant UniProt reference clusters Bioinformatics 2007 23 10 1282 1288 10.1093/bioinformatics/btm098 17379688

[B52] Krogh A Larsson B von Heijne G Sonnhammer EL Predicting transmembrane protein topology with a hidden Markov model: application to complete genomes J Mol Biol 2001 305 3 567 580 10.1006/jmbi.2000.4315 11152613

[B53] Bendtsen JD Nielsen H von Heijne G Brunak S Improved prediction of signal peptides: SignalP 3.0 J Mol Biol 2004 340 4 783 795 10.1016/j.jmb.2004.05.028 15223320

[B54] Thompson JD Higgins DG Gibson TJ CLUSTAL W: improving the sensitivity of progressive multiple sequence alignment through sequence weighting, position-specific gap penalties and weight matrix choice Nucleic Acids Res 1994 22 22 4673 4680 10.1093/nar/22.22.4673 7984417 PMC308517

[B55] Stamatakis A Ott M Efficient computation of the phylogenetic likelihood function on multi-gene alignments and multi-core architectures Philos Trans R Soc Lond B Biol Sci 2008 363 1512 3977 3984 10.1098/rstb.2008.0163 18852107 PMC2607410

[B56] Guindon S Gascuel O A simple, fast, and accurate algorithm to estimate large phylogenies by maximum likelihood Syst Biol 2003 52 5 696 704 10.1080/10635150390235520 14530136

[B57] Guindon S Dufayard JF Lefort V Anisimova M Hordijk W Gascuel O New algorithms and methods to estimate maximum-likelihood phylogenies: assessing the performance of PhyML 3.0 Syst Biol 2010 59 3 307 321 10.1093/sysbio/syq010 20525638

[B58] Carver T Thomson N Bleasby A Berriman M Parkhill J DNAPlotter: circular and linear interactive genome visualization Bioinformatics 2009 25 1 119 120 10.1093/bioinformatics/btn578 18990721 PMC2612626

