# Supplementary material for: Genomic sequence analysis and characterization of Sneathia amnii sp. nov
Source: BMC Genomics. 2012 Dec 17;13(Suppl 8):S4. doi: 10.1186/1471-2164-13-S8-S4 (PMC3535699; doi:10.1186/1471-2164-13-S8-S4)
Supplement: Additional file 2 — Supplementary table 1 - Fraction of genes associated with specific COG functional groups in each species. [file 1471-2164-13-S8-S4-S2.pdf]

Supplementary Table 1. Comparison of the distribution of genes by COG functional category. COG counts were normalized per genome equivalent and the percentage of total counts in COG categories is presented here.

	Code	Description	*S. amnii* (1.34 Mbp)[1]	*S. moniliformis* (1.68 Mbp)[1]	*L. buccalis* (2.47 Mbp)[1]	*S. termiditis* (4.49 Mbp)[1]
Information Storage and Processing	J	Translation, ribosomal structure, and biogenesis	10.43	9.48	6.80	3.94
	K	Transcription	4.75	3.97	3.11	6.64
	L	DNA Replication, recombination, and repair	6.85	6.14	4.95	3.21
Cellular Processing and Signaling	D	Cell cycle control, cell division and chromosome partitioning	2.02	1.32	1.22	0.73
	V	Defense mechanisms	2.10	2.30	2.03	1.25
	T	Signal transduction mechanisms	1.01	1.53	1.89	1.27
	M	Cell wall, membrane and envelope biogenesis	4.51	4.32	5.36	4.04
	N	Cell motility and secretion	0.39	0.98	0.45	0.73
	U	Intracellular trafficking, secretion, and transport	2.10	2.65	1.13	0.51
	O	Post-translational modification, protein turnover, chaperones	3.66	3.63	3.78	2.01
Metabolism	C	Energy production and conversion	3.27	2.93	3.38	3.16
	G	Carbohydrate transport and metabolism	9.03	7.88	4.95	10.56
	E	Amino acid transport and metabolism	5.60	5.30	7.97	5.46
	F	Nucleotide transport and metabolism	3.27	3.63	2.34	2.30
	H	Co-enzyme transport and metabolism	2.02	1.60	3.69	2.40
	I	Lipid transport and metabolism	1.63	1.95	1.85	1.35
	P	Inorganic ion transport and metabolism	3.42	3.14	3.42	2.60
	Q	Secondary metabolite biosynthesis, transport, and catabolism	0.16	0.42	0.45	0.78
Poorly Characterized	R	General functional prediction only	9.88	8.93	11.67	11.32
	S	Function unknown	4.98	5.37	7.52	7.47
	X	Not in COGs	18.91	22.52	22.03	28.26

[1]The number in parenthesis corresponds to the genome size in Megabases.
