# Supplementary material for: Genomic sequence analysis and characterization of Sneathia amnii sp. nov
Source: BMC Genomics. 2012 Dec 17;13(Suppl 8):S4. doi: 10.1186/1471-2164-13-S8-S4 (PMC3535699; doi:10.1186/1471-2164-13-S8-S4)
Supplement: Additional file 3 — Supplementary table 2 - In silico reconstruction of the metabolic pathways of S. amnii, S. moniliformis, L. buccalis and S. termiditis. [file 1471-2164-13-S8-S4-S3.pdf]

Supplementary Table 2 - *In silico* reconstruction of the metabolic pathways of *S. amnii* , *S. moniliformis* , *L. buccalis* and *S. termiditis*

Metabolic reconstruction assignments,
FOUND or NF (Not Found) status (columns D,
E, F and G), were performed using ASGARD,

	EC number	Enzyme/pathway name (KEGG)	*S. amnii*	*S. moniliformis*	*L. buccalis*	*S. termiditis*
1	>Glycolysis / Gluconeogenesis 00010					
2	1.1.1.1	Alcohol dehydrogenase.	FOUND	FOUND	FOUND	FOUND
3	1.1.1.2	Alcohol dehydrogenase (NADP(+)).	NF	NF	FOUND	NF
4	1.1.1.27	L-lactate dehydrogenase.	FOUND	FOUND	NF	FOUND
5	1.1.2.7	Methanol dehydrogenase (cytochrome c).	NF	NF	NF	NF
6	1.1.2.8	Alcohol dehydrogenase (cytochrome c).	NF	NF	NF	NF
7	1.2.1.12	Glyceraldehyde-3-phosphate dehydrogenase (phos	FOUND	FOUND	FOUND	FOUND
8	1.2.1.3	Aldehyde dehydrogenase (NAD(+)).	NF	NF	FOUND	FOUND
9	1.2.1.5	Aldehyde dehydrogenase (NAD(P)(+)).	NF	NF	NF	NF
10	1.2.1.59	Glyceraldehyde-3-phosphate dehydrogenase (NAD(	NF	NF	NF	NF
11	1.2.1.9	Glyceraldehyde-3-phosphate dehydrogenase (NADP	FOUND	FOUND	FOUND	FOUND
12	1.2.4.1	Pyruvate dehydrogenase (acetyl-transferring).	FOUND	FOUND	FOUND	FOUND
13	1.2.7.1	Pyruvate synthase.	NF	NF	NF	NF
14	1.2.7.5	Aldehyde ferredoxin oxidoreductase.	NF	NF	NF	NF
15	1.2.7.6	Glyceraldehyde-3-phosphate dehydrogenase (ferre	NF	NF	NF	NF
16	1.8.1.4	Dihydrolipoyl dehydrogenase.	FOUND	FOUND	FOUND	FOUND
17	2.3.1.12	Dihydrolipoyllysine-residue acetyltransferase.	FOUND	FOUND	FOUND	FOUND
18	2.7.1.1	Hexokinase.	NF	NF	NF	NF
19	2.7.1.11	6-phosphofructokinase.	FOUND	FOUND	FOUND	FOUND
20	2.7.1.146	ADP-specific phosphofructokinase.	NF	NF	NF	NF
21	2.7.1.147	ADP-specific glucokinase.	NF	NF	NF	NF
22	2.7.1.2	Glucokinase.	FOUND	FOUND	FOUND	FOUND
23	2.7.1.40	Pyruvate kinase.	FOUND	FOUND	FOUND	FOUND
24	2.7.1.41	Glucose-1-phosphate phosphodismutase.	NF	NF	NF	NF
25	2.7.1.63	Polyphosphate--glucose phosphotransferase.	NF	NF	NF	NF
26	2.7.1.69	Protein-N(pi)-phosphohistidine--sugar phosphotran	FOUND	FOUND	FOUND	FOUND
27	2.7.2.3	Phosphoglycerate kinase.	FOUND	FOUND	FOUND	FOUND
28	3.1.3.10	Glucose-1-phosphatase.	NF	NF	NF	NF
29	3.1.3.11	Fructose-bisphosphatase.	NF	NF	NF	FOUND
30	3.1.3.13	Bisphosphoglycerate phosphatase.	NF	NF	NF	NF
31	3.1.3.9	Glucose-6-phosphatase.	NF	NF	NF	NF
32	3.2.1.86	6-phospho-beta-glucosidase.	NF	NF	NF	NF
33	4.1.1.1	Pyruvate decarboxylase.	NF	NF	NF	NF
34	4.1.1.32	Phosphoenolpyruvate carboxykinase (GTP).	NF	NF	NF	NF
35	4.1.1.49	Phosphoenolpyruvate carboxykinase (ATP).	NF	NF	FOUND	FOUND
36	4.1.2.13	Fructose-bisphosphate aldolase.	FOUND	FOUND	FOUND	FOUND
37	4.2.1.11	Phosphopyruvate hydratase.	FOUND	FOUND	FOUND	FOUND
38	5.1.3.15	Glucose-6-phosphate 1-epimerase.	NF	NF	NF	NF
39	5.1.3.3	Aldose 1-epimerase.	NF	FOUND	NF	FOUND
40	5.3.1.1	Triose-phosphate isomerase.	FOUND	FOUND	FOUND	FOUND
41	5.3.1.9	Glucose-6-phosphate isomerase.	FOUND	FOUND	FOUND	FOUND
42	5.4.2.1	Phosphoglycerate mutase.	FOUND	FOUND	FOUND	FOUND
43	5.4.2.2	Phosphoglucomutase.	FOUND	FOUND	FOUND	FOUND
44	5.4.2.4	Bisphosphoglycerate mutase.	NF	NF	NF	NF
45	6.2.1.1	Acetate--CoA ligase.	NF	NF	NF	NF
46	6.2.1.13	Acetate--CoA ligase (ADP-forming).	NF	NF	NF	NF
47	>Citrate cycle (TCA cycle) 00020					
48	1.1.1.37	Malate dehydrogenase.	NF	NF	NF	NF
49	1.1.1.41	Isocitrate dehydrogenase (NAD(+)).	NF	NF	FOUND	FOUND

50	1.1.1.42	Isocitrate dehydrogenase (NADP(+)).	NF	NF	FOUND	FOUND
51	1.2.4.1	Pyruvate dehydrogenase (acetyl-transferring).	FOUND	FOUND	FOUND	FOUND
52	1.2.4.2	Oxoglutarate dehydrogenase (succinyl-transferring)	NF	NF	NF	NF
53	1.2.7.1	Pyruvate synthase.	NF	NF	NF	NF
54	1.2.7.3	2-oxoglutarate synthase.	NF	NF	NF	NF
55	1.3.5.1	Succinate dehydrogenase (ubiquinone).	NF	NF	NF	NF
56	1.3.99.1	Succinate dehydrogenase.	NF	NF	NF	NF
57	1.8.1.4	Dihydrolipoyl dehydrogenase.	FOUND	FOUND	FOUND	FOUND
58	2.3.1.12	Dihydrolipoyllysine-residue acetyltransferase.	FOUND	FOUND	FOUND	FOUND
59	2.3.1.61	Dihydrolipoyllysine-residue succinyltransferase.	FOUND	NF	FOUND	NF
60	2.3.3.1	Citrate (Si)-synthase.	NF	NF	FOUND	FOUND
61	2.3.3.8	ATP citrate synthase.	NF	NF	NF	NF
62	4.1.1.32	Phosphoenolpyruvate carboxykinase (GTP).	NF	NF	NF	NF
63	4.1.1.49	Phosphoenolpyruvate carboxykinase (ATP).	NF	NF	FOUND	FOUND
64	4.1.3.6	Citrate (pro-3S)-lyase.	NF	NF	NF	NF
65	4.2.1.2	Fumarate hydratase.	NF	NF	NF	FOUND
66	4.2.1.3	Aconitate hydratase.	NF	NF	FOUND	NF
67	6.2.1.4	Succinate--CoA ligase (GDP-forming).	NF	NF	NF	NF
68	6.2.1.5	Succinate--CoA ligase (ADP-forming).	NF	NF	NF	NF
69	6.4.1.1	Pyruvate carboxylase.	NF	NF	NF	NF
70	>Pentose phosphate pathway 00030					
71	1.1.1.215	Gluconate 2-dehydrogenase.	NF	NF	NF	NF
72	1.1.1.43	Phosphogluconate 2-dehydrogenase.	NF	NF	NF	NF
73	1.1.1.44	Phosphogluconate dehydrogenase (decarboxylating	NF	NF	NF	NF
74	1.1.1.47	Glucose 1-dehydrogenase.	NF	NF	NF	NF
75	1.1.1.49	Glucose-6-phosphate dehydrogenase.	NF	NF	NF	NF
76	1.1.3.4	Glucose oxidase.	NF	NF	NF	NF
77	1.1.3.5	Hexose oxidase.	NF	NF	NF	NF
78	1.1.5.2	Quinoprotein glucose dehydrogenase.	NF	NF	NF	NF
79	1.1.99.10	Glucose dehydrogenase (acceptor).	NF	NF	NF	NF
80	1.1.99.3	Gluconate 2-dehydrogenase (acceptor).	NF	NF	NF	NF
81	1.2.7.5	Aldehyde ferredoxin oxidoreductase.	NF	NF	NF	NF
82	2.2.1.1	Transketolase.	FOUND	FOUND	FOUND	FOUND
83	2.2.1.2	Transaldolase.	FOUND	NF	FOUND	FOUND
84	2.7.1.-	Phosphotransferases with an alcohol group as accep	FOUND	FOUND	FOUND	FOUND
85	2.7.1.11	6-phosphofructokinase.	FOUND	FOUND	FOUND	FOUND
86	2.7.1.12	Gluconokinase.	NF	NF	NF	NF
87	2.7.1.13	Dehydrogluconokinase.	NF	NF	NF	NF
88	2.7.1.15	Ribokinase.	FOUND	FOUND	FOUND	FOUND
89	2.7.1.45	2-dehydro-3-deoxygluconokinase.	FOUND	FOUND	NF	FOUND
90	2.7.4.23	Ribose 1,5-bisphosphate phosphokinase.	NF	NF	NF	NF
91	2.7.6.1	Ribose-phosphate diphosphokinase.	FOUND	FOUND	FOUND	FOUND
92	3.1.1.17	Gluconolactonase.	NF	NF	NF	NF
93	3.1.1.31	6-phosphogluconolactonase.	NF	NF	NF	NF
94	3.1.3.11	Fructose-bisphosphatase.	NF	NF	NF	FOUND
95	4.1.2.-	Aldehyde-lyases.	FOUND	FOUND	FOUND	FOUND
96	4.1.2.13	Fructose-bisphosphate aldolase.	FOUND	FOUND	FOUND	FOUND
97	4.1.2.14	2-dehydro-3-deoxy-phosphogluconate aldolase.	FOUND	FOUND	NF	FOUND
98	4.1.2.4	Deoxyribose-phosphate aldolase.	FOUND	FOUND	FOUND	FOUND
99	4.1.2.9	Phosphoketolase.	NF	NF	NF	NF
100	4.2.1.12	Phosphogluconate dehydratase.	NF	NF	NF	NF
101	4.2.1.39	Gluconate dehydratase.	NF	NF	NF	NF
102	4.3.1.9	Glucosaminate ammonia-lyase.	NF	NF	NF	NF
103	5.1.3.1	Ribulose-phosphate 3-epimerase.	FOUND	FOUND	FOUND	FOUND
104	5.3.1.6	Ribose-5-phosphate isomerase.	FOUND	FOUND	FOUND	FOUND

105	5.3.1.9	Glucose-6-phosphate isomerase.	FOUND	FOUND	FOUND	FOUND
106	5.4.2.2	Phosphoglucomutase.	FOUND	FOUND	FOUND	FOUND
107	5.4.2.7	Phosphopentomutase.	FOUND	NF	FOUND	FOUND
108	>Pentose and glucuronate interconversions 00040					
109	1.1.1.10	L-xylulose reductase.	NF	NF	NF	NF
110	1.1.1.11	D-arabinitol 4-dehydrogenase.	NF	NF	NF	NF
111	1.1.1.12	L-arabinitol 4-dehydrogenase.	NF	NF	NF	NF
112	1.1.1.125	2-deoxy-D-gluconate 3-dehydrogenase.	NF	FOUND	NF	FOUND
113	1.1.1.127	2-dehydro-3-deoxy-D-gluconate 5-dehydrogenase.	NF	NF	NF	NF
114	1.1.1.13	L-arabinitol 2-dehydrogenase.	NF	NF	NF	NF
115	1.1.1.130	3-dehydro-L-gulonate 2-dehydrogenase.	NF	NF	NF	NF
116	1.1.1.137	Ribitol-5-phosphate 2-dehydrogenase.	NF	NF	NF	NF
117	1.1.1.15	D-iditol 2-dehydrogenase.	NF	NF	NF	NF
118	1.1.1.175	D-xylose 1-dehydrogenase.	NF	NF	NF	NF
119	1.1.1.179	D-xylose 1-dehydrogenase (NADP(+)).	NF	NF	NF	NF
120	1.1.1.19	Glucuronate reductase.	NF	NF	NF	NF
121	1.1.1.21	Aldehyde reductase.	NF	NF	FOUND	FOUND
122	1.1.1.22	UDP-glucose 6-dehydrogenase.	NF	NF	NF	FOUND
123	1.1.1.45	L-gulonate 3-dehydrogenase.	NF	NF	NF	NF
124	1.1.1.56	Ribitol 2-dehydrogenase.	NF	NF	NF	NF
125	1.1.1.57	Fructuronate reductase.	FOUND	NF	NF	NF
126	1.1.1.58	Tagaturonate reductase.	NF	NF	NF	FOUND
127	1.1.1.9	D-xylulose reductase.	NF	NF	NF	NF
128	1.1.2.2	Mannitol dehydrogenase (cytochrome).	NF	NF	NF	NF
129	1.2.1.3	Aldehyde dehydrogenase (NAD(+)).	NF	NF	FOUND	FOUND
130	2.4.1.17	Glucuronosyltransferase.	NF	NF	NF	NF
131	2.7.1.16	Ribulokinase.	NF	NF	NF	NF
132	2.7.1.17	Xylulokinase.	FOUND	NF	NF	FOUND
133	2.7.1.43	Glucuronokinase.	NF	NF	NF	NF
134	2.7.1.45	2-dehydro-3-deoxygluconokinase.	FOUND	FOUND	NF	FOUND
135	2.7.1.47	D-ribulokinase.	NF	NF	NF	NF
136	2.7.1.5	Rhamnulokinase.	NF	NF	NF	FOUND
137	2.7.1.53	L-xylulokinase.	NF	NF	NF	NF
138	2.7.7.40	D-ribitol-5-phosphate cytidylyltransferase.	NF	NF	NF	NF
139	2.7.7.44	Glucuronate-1-phosphate uridylyltransferase.	NF	NF	NF	NF
140	2.7.7.9	UTP--glucose-1-phosphate uridylyltransferase.	FOUND	FOUND	FOUND	FOUND
141	3.1.1.11	Pectinesterase.	NF	NF	NF	NF
142	3.1.1.68	Xylono-1,4-lactonase.	NF	NF	NF	NF
143	3.2.1.15	Polygalacturonase.	NF	NF	NF	FOUND
144	3.2.1.31	Beta-glucuronidase.	NF	NF	NF	NF
145	3.2.1.67	Galacturan 1,4-alpha-galacturonidase.	NF	NF	NF	NF
146	4.1.1.34	Dehydro-L-gulonate decarboxylase.	NF	NF	NF	NF
147	4.1.1.85	3-dehydro-L-gulonate-6-phosphate decarboxylase.	NF	NF	NF	NF
148	4.1.2.14	2-dehydro-3-deoxy-phosphogluconate aldolase.	FOUND	FOUND	NF	FOUND
149	4.1.2.19	Rhamnulose-1-phosphate aldolase.	NF	NF	NF	FOUND
150	4.1.2.28	2-dehydro-3-deoxy-D-pentonate aldolase.	NF	NF	NF	NF
151	4.1.2.43	3-hexulose-6-phosphate synthase.	NF	NF	NF	NF
152	4.2.1.-	Hydro-lyases.	FOUND	FOUND	FOUND	FOUND
153	4.2.1.7	Altronate dehydratase.	NF	NF	NF	FOUND
154	4.2.1.8	Mannonate dehydratase.	FOUND	FOUND	NF	FOUND
155	4.2.1.82	Xylonate dehydratase.	NF	NF	NF	NF
156	4.2.2.2	Pectate lyase.	NF	NF	NF	NF
157	4.2.2.6	Oligogalacturonide lyase.	NF	NF	NF	NF
158	4.2.2.9	Pectate disaccharide-lyase.	NF	NF	NF	NF
159	5.-.-.-	Isomerases.	FOUND	FOUND	FOUND	FOUND

160	5.1.3.1	Ribulose-phosphate 3-epimerase.	FOUND	FOUND	FOUND	FOUND
161	5.1.3.4	L-ribulose-5-phosphate 4-epimerase.	FOUND	FOUND	NF	FOUND
162	5.3.1.-	Interconverting aldoses and ketoses, and related co	FOUND	FOUND	FOUND	FOUND
163	5.3.1.12	Glucuronate isomerase.	FOUND	FOUND	NF	FOUND
164	5.3.1.15	D-lyxose ketol-isomerase.	NF	NF	NF	NF
165	5.3.1.17	4-deoxy-L-threo-5-hexosulose-uronate ketol-isomer	NF	FOUND	NF	FOUND
166	5.3.1.27	6-phospho-3-hexuloisomerase.	NF	NF	NF	NF
167	5.3.1.4	L-arabinose isomerase.	NF	NF	NF	NF
168	5.3.1.5	Xylose isomerase.	FOUND	NF	NF	FOUND
169	>Fructose and mannose metabolism 00051					
170	1.1.1.-	With NAD(+) or NADP(+) as acceptor.	FOUND	FOUND	FOUND	FOUND
171	1.1.1.11	D-arabinitol 4-dehydrogenase.	NF	NF	NF	NF
172	1.1.1.132	GDP-mannose 6-dehydrogenase.	NF	NF	NF	NF
173	1.1.1.135	GDP-6-deoxy-D-talose 4-dehydrogenase.	NF	NF	NF	NF
174	1.1.1.138	Mannitol 2-dehydrogenase (NADP(+)).	NF	NF	NF	NF
175	1.1.1.14	L-iditol 2-dehydrogenase.	NF	NF	NF	FOUND
176	1.1.1.140	Sorbitol-6-phosphate 2-dehydrogenase.	NF	NF	NF	FOUND
177	1.1.1.15	D-iditol 2-dehydrogenase.	NF	NF	NF	NF
178	1.1.1.17	Mannitol-1-phosphate 5-dehydrogenase.	NF	NF	NF	FOUND
179	1.1.1.173	L-rhamnose 1-dehydrogenase.	NF	NF	NF	NF
180	1.1.1.187	GDP-4-dehydro-D-rhamnose reductase.	NF	NF	NF	NF
181	1.1.1.21	Aldehyde reductase.	NF	NF	FOUND	FOUND
182	1.1.1.271	GDP-L-fucose synthase.	NF	NF	NF	NF
183	1.1.1.281	GDP-4-dehydro-6-deoxy-D-mannose reductase.	NF	NF	NF	NF
184	1.1.1.67	Mannitol 2-dehydrogenase.	NF	NF	NF	NF
185	1.1.2.2	Mannitol dehydrogenase (cytochrome).	NF	NF	NF	NF
186	1.1.99.21	D-sorbitol dehydrogenase (acceptor).	NF	NF	NF	NF
187	2.4.1.-	Hexosyltransferases.	FOUND	FOUND	FOUND	FOUND
188	2.4.1.217	Mannosyl-3-phosphoglycerate synthase.	NF	NF	NF	NF
189	2.4.1.33	Alginate synthase.	NF	NF	NF	NF
190	2.7.1.1	Hexokinase.	NF	NF	NF	NF
191	2.7.1.105	6-phosphofructo-2-kinase.	NF	NF	NF	NF
192	2.7.1.11	6-phosphofructokinase.	FOUND	FOUND	FOUND	FOUND
193	2.7.1.28	Triokinase.	NF	NF	NF	NF
194	2.7.1.3	Ketohexokinase.	NF	NF	NF	NF
195	2.7.1.4	Fructokinase.	NF	NF	FOUND	FOUND
196	2.7.1.5	Rhamnulokinase.	NF	NF	NF	FOUND
197	2.7.1.51	L-fuculokinase.	NF	NF	NF	NF
198	2.7.1.52	Fucokinase.	NF	NF	NF	NF
199	2.7.1.56	1-phosphofructokinase.	FOUND	FOUND	FOUND	FOUND
200	2.7.1.69	Protein-N(pi)-phosphohistidine--sugar phosphotran	FOUND	FOUND	FOUND	FOUND
201	2.7.1.7	Mannokinase.	NF	NF	NF	NF
202	2.7.1.90	Diphosphate--fructose-6-phosphate 1-phosphotran	NF	NF	NF	NF
203	2.7.7.13	Mannose-1-phosphate guanylyltransferase.	FOUND	NF	FOUND	FOUND
204	2.7.7.22	Mannose-1-phosphate guanylyltransferase (GDP).	FOUND	NF	FOUND	FOUND
205	2.7.7.30	Fucose-1-phosphate guanylyltransferase.	NF	NF	NF	NF
206	3.1.1.65	L-rhamnono-1,4-lactonase.	NF	NF	NF	NF
207	3.1.3.-	Phosphoric monoester hydrolases.	FOUND	FOUND	FOUND	FOUND
208	3.1.3.11	Fructose-bisphosphatase.	NF	NF	NF	FOUND
209	3.1.3.22	Mannitol-1-phosphatase.	NF	NF	NF	NF
210	3.1.3.46	Fructose-2,6-bisphosphate 2-phosphatase.	NF	NF	NF	NF
211	3.1.3.54	Fructose-2,6-bisphosphate 6-phosphatase.	NF	NF	NF	NF
212	3.1.3.70	Mannosyl-3-phosphoglycerate phosphatase.	NF	NF	NF	NF
213	3.2.1.137	Mannan exo-1,2-1,6-alpha-mannosidase.	NF	NF	NF	NF
214	3.2.1.77	Mannan 1,2-(1,3)-alpha-mannosidase.	NF	NF	NF	NF

215	3.2.1.78	Mannan endo-1,4-beta-mannosidase.	NF	NF	NF	NF
216	3.2.1.80	Fructan beta-fructosidase.	NF	NF	NF	NF
217	3.6.1.21	ADP-sugar diphosphatase.	NF	NF	NF	NF
218	4.1.2.-	Aldehyde-lyases.	FOUND	FOUND	FOUND	FOUND
219	4.1.2.13	Fructose-bisphosphate aldolase.	FOUND	FOUND	FOUND	FOUND
220	4.1.2.17	L-fuculose-phosphate aldolase.	FOUND	NF	NF	NF
221	4.1.2.18	2-dehydro-3-deoxy-L-pentonate aldolase.	NF	NF	NF	NF
222	4.1.2.19	Rhamnulose-1-phosphate aldolase.	NF	NF	NF	FOUND
223	4.2.1.47	GDP-mannose 4,6-dehydratase.	NF	NF	NF	NF
224	4.2.1.67	D-fuconate dehydratase.	NF	NF	NF	NF
225	4.2.1.90	L-rhamnonate dehydratase.	NF	NF	NF	NF
226	4.2.2.11	Poly(alpha-L-guluronate) lyase.	NF	NF	NF	NF
227	4.2.2.3	Poly(beta-D-mannuronate) lyase.	NF	FOUND	NF	NF
228	5.3.1.1	Triose-phosphate isomerase.	FOUND	FOUND	FOUND	FOUND
229	5.3.1.14	L-rhamnose isomerase.	NF	NF	NF	FOUND
230	5.3.1.25	L-fucose isomerase.	NF	NF	NF	NF
231	5.3.1.5	Xylose isomerase.	FOUND	NF	NF	FOUND
232	5.3.1.7	Mannose isomerase.	NF	NF	NF	NF
233	5.3.1.8	Mannose-6-phosphate isomerase.	FOUND	FOUND	FOUND	FOUND
234	5.4.2.8	Phosphomannomutase.	NF	NF	NF	FOUND
235	>Galactose metabolism 00052					
236	1.1.1.120	Galactose 1-dehydrogenase (NADP(+)).	NF	NF	NF	NF
237	1.1.1.16	Galactitol 2-dehydrogenase.	NF	NF	NF	NF
238	1.1.1.21	Aldehyde reductase.	NF	NF	FOUND	FOUND
239	1.1.1.251	Galactitol-1-phosphate 5-dehydrogenase.	NF	NF	NF	NF
240	1.1.1.48	Galactose 1-dehydrogenase.	NF	NF	NF	NF
241	1.1.3.9	Galactose oxidase.	NF	NF	NF	NF
242	2.4.1.123	Inositol 3-alpha-galactosyltransferase.	NF	NF	NF	NF
243	2.4.1.22	Lactose synthase.	NF	NF	NF	NF
244	2.4.1.67	Galactinol--raffinose galactosyltransferase.	NF	NF	NF	NF
245	2.4.1.82	Galactinol--sucrose galactosyltransferase.	NF	NF	NF	NF
246	2.7.1.1	Hexokinase.	NF	NF	NF	NF
247	2.7.1.101	Tagatose kinase.	NF	NF	NF	NF
248	2.7.1.11	6-phosphofructokinase.	FOUND	FOUND	FOUND	FOUND
249	2.7.1.144	Tagatose-6-phosphate kinase.	NF	FOUND	FOUND	FOUND
250	2.7.1.2	Glucokinase.	FOUND	FOUND	FOUND	FOUND
251	2.7.1.58	2-dehydro-3-deoxygalactonokinase.	NF	NF	NF	NF
252	2.7.1.6	Galactokinase.	NF	FOUND	FOUND	FOUND
253	2.7.1.69	Protein-N(pi)-phosphohistidine--sugar phosphotran	FOUND	FOUND	FOUND	FOUND
254	2.7.7.10	UTP--hexose-1-phosphate uridylyltransferase.	NF	NF	NF	NF
255	2.7.7.12	UDP-glucose--hexose-1-phosphate uridylyltransfera	FOUND	FOUND	FOUND	FOUND
256	2.7.7.9	UTP--glucose-1-phosphate uridylyltransferase.	FOUND	FOUND	FOUND	FOUND
257	3.1.1.25	1,4-lactonase.	NF	NF	NF	NF
258	3.1.3.9	Glucose-6-phosphatase.	NF	NF	NF	NF
259	3.2.1.108	Lactase.	NF	NF	NF	NF
260	3.2.1.20	Alpha-glucosidase.	NF	NF	NF	NF
261	3.2.1.22	Alpha-galactosidase.	NF	NF	NF	NF
262	3.2.1.23	Beta-galactosidase.	NF	NF	NF	NF
263	3.2.1.26	Beta-fructofuranosidase.	NF	NF	NF	NF
264	3.2.1.85	6-phospho-beta-galactosidase.	NF	NF	FOUND	FOUND
265	3.5.1.25	N-acetylglucosamine-6-phosphate deacetylase.	FOUND	FOUND	FOUND	FOUND
266	4.1.2.21	2-dehydro-3-deoxy-6-phosphogalactonate aldolase.	NF	NF	NF	NF
267	4.1.2.40	Tagatose-bisphosphate aldolase.	NF	FOUND	FOUND	FOUND
268	4.2.1.6	Galactonate dehydratase.	NF	NF	NF	FOUND
269	5.1.3.2	UDP-glucose 4-epimerase.	NF	FOUND	FOUND	FOUND

270	5.3.1.-	Interconverting aldoses and ketoses, and related co	FOUND	FOUND	FOUND	FOUND
271	5.3.1.26	Galactose-6-phosphate isomerase.	NF	NF	FOUND	FOUND
272	5.4.2.2	Phosphoglucomutase.	FOUND	FOUND	FOUND	FOUND
273	>Ascorbate and aldarate metabolism 00053					
274	1.1.1.-	With NAD(+) or NADP(+) as acceptor.	FOUND	FOUND	FOUND	FOUND
275	1.1.1.122	D-threo-aldose 1-dehydrogenase.	NF	NF	NF	NF
276	1.1.1.129	L-threonate 3-dehydrogenase.	NF	NF	NF	NF
277	1.1.1.130	3-dehydro-L-gulonate 2-dehydrogenase.	NF	NF	NF	NF
278	1.1.1.19	Glucuronate reductase.	NF	NF	NF	NF
279	1.1.1.20	Glucuronolactone reductase.	NF	NF	NF	NF
280	1.1.1.22	UDP-glucose 6-dehydrogenase.	NF	NF	NF	FOUND
281	1.1.1.46	L-arabinose 1-dehydrogenase.	NF	NF	NF	NF
282	1.1.3.8	L-gulonolactone oxidase.	NF	NF	NF	NF
283	1.10.3.3	L-ascorbate oxidase.	NF	NF	NF	NF
284	1.11.1.11	L-ascorbate peroxidase.	NF	NF	NF	NF
285	1.13.11.13	Ascorbate 2,3-dioxygenase.	NF	NF	NF	NF
286	1.13.99.1	Inositol oxygenase.	NF	NF	NF	NF
287	1.14.-.-	Acting on paired donors, with incorporation or redu	NF	NF	NF	NF
288	1.2.1.26	2,5-dioxovalerate dehydrogenase.	NF	NF	NF	NF
289	1.2.1.3	Aldehyde dehydrogenase (NAD(+)).	NF	NF	FOUND	FOUND
290	1.3.2.3	L-galactonolactone dehydrogenase.	NF	NF	NF	NF
291	1.3.3.12	L-galactonolactone oxidase.	NF	NF	NF	NF
292	1.6.5.4	Monodehydroascorbate reductase (NADH).	NF	NF	NF	NF
293	1.8.5.1	Glutathione dehydrogenase (ascorbate).	NF	NF	NF	NF
294	2.4.1.17	Glucuronosyltransferase.	NF	NF	NF	NF
295	2.7.1.43	Glucuronokinase.	NF	NF	NF	NF
296	2.7.1.53	L-xylulokinase.	NF	NF	NF	NF
297	2.7.1.69	Protein-N(pi)-phosphohistidine--sugar phosphotran	FOUND	FOUND	FOUND	FOUND
298	2.7.7.44	Glucuronate-1-phosphate uridylyltransferase.	NF	NF	NF	NF
299	2.7.7.69	GDP-L-galactose phosphorylase.	NF	NF	NF	NF
300	3.1.1.-	Carboxylic ester hydrolases.	FOUND	FOUND	FOUND	FOUND
301	3.1.1.15	L-arabinonolactonase.	NF	NF	NF	NF
302	3.1.1.17	Gluconolactonase.	NF	NF	NF	NF
303	3.1.1.19	Uronolactonase.	NF	NF	NF	NF
304	3.1.1.25	1,4-lactonase.	NF	NF	NF	NF
305	3.1.3.-	Phosphoric monoester hydrolases.	FOUND	FOUND	FOUND	FOUND
306	3.2.1.-	Glycosidases, i.e. enzymes hydrolyzing O- and S-glyc	FOUND	FOUND	FOUND	FOUND
307	3.7.1.-	In ketonic substances.	NF	NF	NF	FOUND
308	4.1.1.-	Carboxy-lyases.	FOUND	FOUND	FOUND	FOUND
309	4.1.1.85	3-dehydro-L-gulonate-6-phosphate decarboxylase.	NF	NF	NF	NF
310	4.1.2.18	2-dehydro-3-deoxy-L-pentonate aldolase.	NF	NF	NF	NF
311	4.1.2.20	2-dehydro-3-deoxyglucarate aldolase.	NF	NF	NF	NF
312	4.2.1.25	L-arabinonate dehydratase.	NF	NF	NF	NF
313	4.2.1.40	Glucarate dehydratase.	NF	NF	NF	NF
314	4.2.1.41	5-dehydro-4-deoxyglucarate dehydratase.	NF	NF	NF	NF
315	4.2.1.42	Galactarate dehydratase.	NF	NF	NF	NF
316	4.2.1.43	2-dehydro-3-deoxy-L-arabinonate dehydratase.	NF	NF	NF	NF
317	5.1.3.18	GDP-mannose 3,5-epimerase.	NF	NF	NF	NF
318	5.1.3.22	L-ribulose-5-phosphate 3-epimerase.	NF	NF	NF	NF
319	5.1.3.4	L-ribulose-5-phosphate 4-epimerase.	FOUND	FOUND	NF	FOUND
320	>Fatty acid biosynthesis 00061					
321	1.1.1.100	3-oxoacyl-[acyl-carrier-protein] reductase.	NF	NF	FOUND	FOUND
322	1.14.19.2	Acyl-[acyl-carrier-protein] desaturase.	NF	NF	NF	NF
323	1.3.1.-	With NAD(+) or NADP(+) as acceptor.	FOUND	FOUND	FOUND	FOUND
324	1.3.1.9	Enoyl-[acyl-carrier-protein] reductase (NADH).	NF	NF	NF	NF

325	2.3.1.-	Transferring groups other than amino-acyl groups.	FOUND	FOUND	FOUND	FOUND
326	2.3.1.179	Beta-ketoacyl-acyl-carrier-protein synthase II.	NF	NF	NF	NF
327	2.3.1.180	Beta-ketoacyl-acyl-carrier-protein synthase III.	NF	NF	NF	NF
328	2.3.1.39	[Acyl-carrier-protein] S-malonyltransferase.	NF	NF	FOUND	FOUND
329	2.3.1.41	Beta-ketoacyl-acyl-carrier-protein synthase I.	NF	NF	FOUND	FOUND
330	2.3.1.85	Fatty-acid synthase.	NF	NF	NF	NF
331	2.3.1.86	Fatty-acyl-CoA synthase.	NF	NF	NF	NF
332	3.1.2.-	Thiolester hydrolases.	NF	FOUND	NF	NF
333	3.1.2.14	Oleoyl-[acyl-carrier-protein] hydrolase.	NF	NF	NF	NF
334	3.1.2.21	Dodecanoyl-[acyl-carrier-protein] hydrolase.	NF	NF	NF	NF
335	4.2.1.-	Hydro-lyases.	FOUND	FOUND	FOUND	FOUND
336	4.2.1.58	Crotonoyl-[acyl-carrier-protein] hydratase.	NF	NF	NF	NF
337	4.2.1.60	3-hydroxydecanoyl-[acyl-carrier-protein] dehydrata	NF	NF	NF	NF
338	4.2.1.61	3-hydroxypalmitoyl-[acyl-carrier-protein] dehydrata	NF	NF	NF	NF
339	6.3.4.14	Biotin carboxylase.	NF	NF	FOUND	FOUND
340	6.4.1.2	Acetyl-CoA carboxylase.	NF	NF	FOUND	FOUND
341	>Fatty acid elongation in mitochondria 00062					
342	1.1.1.211	Long-chain-3-hydroxyacyl-CoA dehydrogenase.	NF	NF	NF	NF
343	1.1.1.35	3-hydroxyacyl-CoA dehydrogenase.	NF	NF	NF	NF
344	1.3.1.38	Trans-2-enoyl-CoA reductase (NADPH).	NF	NF	NF	NF
345	1.3.1.8	Acyl-CoA dehydrogenase (NADP(+)).	NF	NF	NF	NF
346	2.3.1.16	Acetyl-CoA C-acyltransferase.	NF	NF	NF	NF
347	3.1.2.22	Palmitoyl-protein hydrolase.	NF	NF	NF	NF
348	4.2.1.17	Enoyl-CoA hydratase.	NF	NF	NF	NF
349	4.2.1.74	Long-chain-enoyl-CoA hydratase.	NF	NF	NF	NF
350	>Fatty acid metabolism 00071					
351	1.1.1.1	Alcohol dehydrogenase.	FOUND	FOUND	FOUND	FOUND
352	1.1.1.192	Long-chain-alcohol dehydrogenase.	NF	NF	NF	NF
353	1.1.1.211	Long-chain-3-hydroxyacyl-CoA dehydrogenase.	NF	NF	NF	NF
354	1.1.1.35	3-hydroxyacyl-CoA dehydrogenase.	NF	NF	NF	NF
355	1.1.99.20	Alkan-1-ol dehydrogenase (acceptor).	NF	NF	NF	NF
356	1.14.14.1	Unspecific monooxygenase.	NF	NF	NF	NF
357	1.14.15.3	Alkane 1-monooxygenase.	NF	NF	NF	NF
358	1.18.1.1	Rubredoxin--NAD(+) reductase.	NF	NF	NF	NF
359	1.18.1.3	Ferredoxin--NAD(+) reductase.	NF	NF	NF	NF
360	1.18.1.4	Rubredoxin--NAD(P)(+) reductase.	NF	NF	NF	NF
361	1.2.1.3	Aldehyde dehydrogenase (NAD(+)).	NF	NF	FOUND	FOUND
362	1.2.1.48	Long-chain-aldehyde dehydrogenase.	NF	NF	NF	NF
363	1.2.99.3	Aldehyde dehydrogenase (pyrroloquinoline-quinon	NF	NF	NF	NF
364	1.3.3.6	Acyl-CoA oxidase.	NF	NF	NF	NF
365	1.3.99.-	With other acceptors.	NF	NF	FOUND	FOUND
366	1.3.99.13	Long-chain-acyl-CoA dehydrogenase.	NF	NF	NF	NF
367	1.3.99.2	Butyryl-CoA dehydrogenase.	NF	NF	NF	NF
368	1.3.99.3	Acyl-CoA dehydrogenase.	NF	NF	FOUND	FOUND
369	1.3.99.7	Glutaryl-CoA dehydrogenase.	NF	NF	NF	NF
370	2.3.1.16	Acetyl-CoA C-acyltransferase.	NF	NF	NF	NF
371	2.3.1.21	Carnitine O-palmitoyltransferase.	NF	NF	NF	NF
372	2.3.1.9	Acetyl-CoA C-acetyltransferase.	FOUND	FOUND	NF	NF
373	4.2.1.17	Enoyl-CoA hydratase.	NF	NF	NF	NF
374	4.2.1.74	Long-chain-enoyl-CoA hydratase.	NF	NF	NF	NF
375	5.1.2.3	3-hydroxybutyryl-CoA epimerase.	NF	NF	NF	NF
376	5.3.3.8	Dodecenoyl-CoA isomerase.	NF	NF	NF	NF
377	6.2.1.20	Long-chain-fatty-acid--[acyl-carrier-protein] ligase.	NF	NF	NF	NF
378	6.2.1.3	Long-chain-fatty-acid--CoA ligase.	NF	NF	NF	NF
379	6.2.1.6	Glutarate--CoA ligase.	NF	NF	NF	NF

380	>Synthesis and degradation of ketone bodies 00072					
381	1.1.1.30	3-hydroxybutyrate dehydrogenase.	NF	NF	NF	FOUND
382	2.3.1.9	Acetyl-CoA C-acetyltransferase.	FOUND	FOUND	NF	NF
383	2.3.3.10	Hydroxymethylglutaryl-CoA synthase.	FOUND	FOUND	FOUND	NF
384	2.8.3.5	3-oxoacid CoA-transferase.	NF	NF	NF	NF
385	4.1.1.4	Acetoacetate decarboxylase.	NF	NF	NF	NF
386	4.1.3.4	Hydroxymethylglutaryl-CoA lyase.	NF	NF	NF	NF
387	>Steroid biosynthesis 00100					
388	1.1.1.170	Sterol-4-alpha-carboxylate 3-dehydrogenase (decar	NF	NF	NF	NF
389	1.1.1.270	3-keto-steroid reductase.	NF	NF	NF	NF
390	1.1.3.6	Cholesterol oxidase.	NF	NF	NF	NF
391	1.14.-.-	Acting on paired donors, with incorporation or redu	NF	NF	NF	NF
392	1.14.13.13	Calcidiol 1-monooxygenase.	NF	NF	NF	NF
393	1.14.13.70	Sterol 14-demethylase.	NF	NF	NF	NF
394	1.14.13.72	Methylsterol monooxygenase.	NF	NF	NF	NF
395	1.14.21.6	Lathosterol oxidase.	NF	NF	NF	NF
396	1.14.99.7	Squalene monooxygenase.	NF	NF	NF	NF
397	1.3.1.21	7-dehydrocholesterol reductase.	NF	NF	NF	NF
398	1.3.1.70	Delta(14)-sterol reductase.	NF	NF	NF	NF
399	1.3.1.71	Delta(24(24(1)))-sterol reductase.	NF	NF	NF	NF
400	1.3.1.72	Delta(24)-sterol reductase.	NF	NF	NF	NF
401	1.3.3.-	With oxygen as acceptor.	FOUND	FOUND	FOUND	FOUND
402	2.1.1.143	24-methylenesterol C-methyltransferase.	NF	NF	NF	NF
403	2.1.1.41	Sterol 24-C-methyltransferase.	NF	NF	NF	NF
404	2.3.1.26	Sterol O-acyltransferase.	NF	NF	NF	NF
405	2.3.1.73	Diacylglycerol--sterol O-acyltransferase.	NF	NF	NF	NF
406	2.5.1.21	Squalene synthase.	NF	NF	NF	NF
407	3.1.1.13	Sterol esterase.	NF	NF	NF	NF
408	5.-.-.-	Isomerases.	FOUND	FOUND	FOUND	FOUND
409	5.3.3.5	Cholestenol Delta-isomerase.	NF	NF	NF	NF
410	5.4.99.17	Squalene--hopene cyclase.	NF	NF	NF	NF
411	5.4.99.7	Lanosterol synthase.	NF	NF	NF	NF
412	5.4.99.8	Cycloartenol synthase.	NF	NF	NF	NF
413	5.5.1.9	Cycloeucalenol cycloisomerase.	NF	NF	NF	NF
414	>Primary bile acid biosynthesis 00120					
415	1.1.1.181	Cholest-5-ene-3-beta,7-alpha-diol 3-beta-dehydrog	NF	NF	NF	NF
416	1.1.1.35	3-hydroxyacyl-CoA dehydrogenase.	NF	NF	NF	NF
417	1.1.1.50	3-alpha-hydroxysteroid dehydrogenase (B-specific).	NF	NF	NF	NF
418	1.14.13.1	Salicylate 1-monooxygenase.	NF	NF	NF	NF
419	1.14.13.100	25-hydroxycholesterol 7-alpha-hydroxylase.	NF	NF	NF	NF
420	1.14.13.15	Cholestanetriol 26-monooxygenase.	NF	NF	NF	NF
421	1.14.13.17	Cholesterol 7-alpha-monooxygenase.	NF	NF	NF	NF
422	1.14.13.96	5-beta-cholestane-3-alpha,7-alpha-diol 12-alpha-hy	NF	NF	NF	NF
423	1.14.13.98	Cholesterol 24-hydroxylase.	NF	NF	NF	NF
424	1.14.13.99	24-hydroxycholesterol 7-alpha-hydroxylase.	NF	NF	NF	NF
425	1.14.99.38	Cholesterol 25-hydroxylase.	NF	NF	NF	NF
426	1.17.99.3	3-alpha,7-alpha,12-alpha-trihydroxy-5-beta-cholest	NF	NF	NF	NF
427	1.3.1.3	Delta(4)-3-oxosteroid 5-beta-reductase.	NF	NF	NF	NF
428	2.3.1.176	Propanoyl-CoA C-acyltransferase.	NF	NF	NF	NF
429	2.3.1.65	Bile acid-CoA:amino acid N-acyltransferase.	NF	NF	NF	NF
430	3.5.1.24	Choloylglycine hydrolase.	NF	NF	NF	NF
431	4.2.1.107	3-alpha,7-alpha,12-alpha-trihydroxy-5-beta-cholest	NF	NF	NF	NF
432	5.1.99.4	Alpha-methylacyl-CoA racemase.	NF	NF	NF	NF
433	6.2.1.7	Cholate--CoA ligase.	NF	NF	NF	NF
434	>Secondary bile acid biosynthesis 00121					

435	1.-.-.-	Oxidoreductases.	FOUND	FOUND	FOUND	FOUND
436	1.17.99.5	Bile-acid 7-alpha-dehydroxylase.	NF	NF	NF	NF
437	3.1.2.26	Bile-acid-CoA hydrolase.	NF	NF	NF	NF
438	3.5.1.24	Choloylglycine hydrolase.	NF	NF	NF	NF
439	3.5.1.74	Chenodeoxycholoyltaurine hydrolase.	NF	NF	NF	NF
440	4.2.1.-	Hydro-lyases.	FOUND	FOUND	FOUND	FOUND
441	4.2.1.106	Bile-acid 7-alpha-dehydratase.	NF	NF	NF	NF
442	6.-.-.-	Ligases.	FOUND	FOUND	FOUND	FOUND
443	>Ubiquinone and other terpenoid-quinone biosynthesis 00130					
444	1.1.1.237	Hydroxyphenylpyruvate reductase.	NF	NF	NF	NF
445	1.13.11.27	4-hydroxyphenylpyruvate dioxygenase.	NF	NF	NF	NF
446	1.14.-.-	Acting on paired donors, with incorporation or redu	NF	NF	NF	NF
447	1.14.13.-	With NADH or NADPH as one donor, and incorporat	NF	NF	NF	NF
448	1.21.-.-	Acting on x-H and y-H to form an x-y bond.	NF	NF	NF	NF
449	2.1.1.-	Methyltransferases.	FOUND	FOUND	FOUND	FOUND
450	2.1.1.114	Hexaprenyldihydroxybenzoate methyltransferase.	NF	NF	NF	NF
451	2.1.1.64	3-demethylubiquinone-9 3-O-methyltransferase.	NF	NF	NF	NF
452	2.1.1.95	Tocopherol O-methyltransferase.	NF	NF	NF	NF
453	2.2.1.9	2-succinyl-5-enolpyruvyl-6-hydroxy-3-cyclohexene-1	NF	NF	NF	NF
454	2.4.1.194	4-hydroxybenzoate 4-O-beta-D-glucosyltransferase.	NF	NF	NF	NF
455	2.5.1.-	Transferring alkyl or aryl groups, other than methyl	FOUND	FOUND	FOUND	FOUND
456	2.5.1.39	4-hydroxybenzoate polyprenyltransferase.	NF	NF	NF	NF
457	2.5.1.74	1,4-dihydroxy-2-naphthoate polyprenyltransferase.	NF	NF	NF	NF
458	2.6.1.5	Tyrosine transaminase.	NF	NF	NF	NF
459	3.1.2.-	Thiolester hydrolases.	NF	FOUND	NF	NF
460	3.1.2.23	4-hydroxybenzoyl-CoA thioesterase.	NF	NF	NF	NF
461	3.2.1.-	Glycosidases, i.e. enzymes hydrolyzing O- and S-glyc	FOUND	FOUND	FOUND	FOUND
462	3.2.2.26	Futalosine hydrolase.	NF	NF	NF	NF
463	4.1.1.-	Carboxy-lyases.	FOUND	FOUND	FOUND	FOUND
464	4.1.3.36	1,4-dihydroxy-2-naphthoyl-CoA synthase.	NF	NF	NF	NF
465	4.1.3.40	Chorismate lyase.	NF	NF	NF	NF
466	4.1.99.-	Other carbon-carbon lyases.	NF	NF	NF	FOUND
467	4.2.1.113	o-succinylbenzoate synthase.	NF	NF	NF	NF
468	4.2.99.20	2-succinyl-6-hydroxy-2,4-cyclohexadiene-1-carboxyl	NF	NF	NF	NF
469	5.4.4.2	Isochorismate synthase.	NF	NF	NF	NF
470	6.2.1.12	4-coumarate--CoA ligase.	NF	NF	NF	NF
471	6.2.1.26	o-succinylbenzoate--CoA ligase.	NF	NF	NF	NF
472	>Steroid hormone biosynthesis 00140					
473	1.1.1.-	With NAD(+) or NADP(+) as acceptor.	FOUND	FOUND	FOUND	FOUND
474	1.1.1.145	3-beta-hydroxy-Delta(5)-steroid dehydrogenase.	NF	NF	NF	NF
475	1.1.1.146	11-beta-hydroxysteroid dehydrogenase.	NF	NF	NF	NF
476	1.1.1.148	Estradiol 17-alpha-dehydrogenase.	NF	NF	NF	NF
477	1.1.1.149	20-alpha-hydroxysteroid dehydrogenase.	NF	NF	NF	NF
478	1.1.1.152	3-alpha-hydroxy-5-beta-androstane-17-one 3-alpha	NF	NF	NF	NF
479	1.1.1.213	3-alpha-hydroxysteroid dehydrogenase (A-specific).	NF	NF	NF	NF
480	1.1.1.239	3-alpha-(17-beta)-hydroxysteroid dehydrogenase (N	NF	NF	NF	NF
481	1.1.1.50	3-alpha-hydroxysteroid dehydrogenase (B-specific).	NF	NF	NF	NF
482	1.1.1.51	3(or 17)-beta-hydroxysteroid dehydrogenase.	NF	NF	NF	NF
483	1.1.1.53	3-alpha-(or 20-beta)-hydroxysteroid dehydrogenase	NF	NF	NF	NF
484	1.1.1.62	Estradiol 17-beta-dehydrogenase.	NF	NF	NF	NF
485	1.1.1.63	Testosterone 17-beta-dehydrogenase.	NF	NF	NF	NF
486	1.1.1.64	Testosterone 17-beta-dehydrogenase (NADP(+)).	NF	NF	NF	NF
487	1.14.13.100	25-hydroxycholesterol 7-alpha-hydroxylase.	NF	NF	NF	NF
488	1.14.13.17	Cholesterol 7-alpha-monooxygenase.	NF	NF	NF	NF
489	1.14.14.1	Unspecific monooxygenase.	NF	NF	NF	NF

490	1.14.15.4	Steroid 11-beta-monooxygenase.	NF	NF	NF	NF
491	1.14.15.5	Corticosterone 18-monooxygenase.	NF	NF	NF	NF
492	1.14.15.6	Cholesterol monooxygenase (side-chain-cleaving).	NF	NF	NF	NF
493	1.14.99.10	Steroid 21-monooxygenase.	NF	NF	NF	NF
494	1.14.99.11	Estradiol 6-beta-monooxygenase.	NF	NF	NF	NF
495	1.14.99.12	Androst-4-ene-3,17-dione monooxygenase.	NF	NF	NF	NF
496	1.14.99.14	Progesterone 11-alpha-monooxygenase.	NF	NF	NF	NF
497	1.14.99.9	Steroid 17-alpha-monooxygenase.	NF	NF	NF	NF
498	1.3.1.3	Delta(4)-3-oxosteroid 5-beta-reductase.	NF	NF	NF	NF
499	1.3.1.30	Progesterone 5-alpha-reductase.	NF	NF	NF	NF
500	1.3.99.5	3-oxo-5-alpha-steroid 4-dehydrogenase.	NF	NF	NF	NF
501	1.3.99.6	3-oxo-5-beta-steroid 4-dehydrogenase.	NF	NF	NF	NF
502	2.1.1.6	Catechol O-methyltransferase.	NF	NF	NF	NF
503	2.4.1.17	Glucuronosyltransferase.	NF	NF	NF	NF
504	2.8.2.15	Steroid sulfotransferase.	NF	NF	NF	NF
505	2.8.2.2	Alcohol sulfotransferase.	NF	NF	NF	NF
506	2.8.2.4	Estrone sulfotransferase.	NF	NF	NF	NF
507	3.1.6.1	Arylsulfatase.	NF	NF	NF	FOUND
508	3.1.6.2	Steryl-sulfatase.	NF	NF	NF	NF
509	4.1.2.30	17-alpha-hydroxyprogesterone aldolase.	NF	NF	NF	NF
510	5.3.3.1	Steroid Delta-isomerase.	NF	NF	NF	NF
511	>Oxidative phosphorylation 00190					
512	1.10.2.2	Ubiquinol--cytochrome-c reductase.	NF	NF	NF	NF
513	1.3.5.1	Succinate dehydrogenase (ubiquinone).	NF	NF	NF	NF
514	1.3.99.1	Succinate dehydrogenase.	NF	NF	NF	NF
515	1.6.5.3	NADH dehydrogenase (ubiquinone).	NF	NF	NF	FOUND
516	1.6.99.3	NADH dehydrogenase.	FOUND	NF	FOUND	FOUND
517	1.6.99.5	NADH dehydrogenase (quinone).	NF	NF	NF	NF
518	1.9.3.1	Cytochrome-c oxidase.	NF	NF	NF	NF
519	2.7.4.1	Polyphosphate kinase.	NF	NF	NF	FOUND
520	3.6.1.1	Inorganic diphosphatase.	FOUND	FOUND	FOUND	FOUND
521	3.6.3.10	Hydrogen/potassium-exchanging ATPase.	NF	NF	NF	NF
522	3.6.3.14	H(+)-transporting two-sector ATPase.	FOUND	NF	FOUND	NF
523	3.6.3.6	Proton-exporting ATPase.	FOUND	FOUND	FOUND	FOUND
524	>Photosynthesis 00195					
525	1.10.99.1	Plastoquinol--plastocyanin reductase.	NF	NF	NF	NF
526	1.18.1.2	Ferredoxin--NADP(+) reductase.	NF	NF	NF	NF
527	3.6.3.14	H(+)-transporting two-sector ATPase.	FOUND	NF	FOUND	NF
528	>Photosynthesis - antenna proteins 00196					
529	4.-.-.-	Lyases.	FOUND	FOUND	FOUND	FOUND
530	>Purine metabolism 00230					
531	1.1.1.154	Ureidoglycolate dehydrogenase.	NF	NF	NF	NF
532	1.1.1.205	IMP dehydrogenase.	FOUND	FOUND	FOUND	FOUND
533	1.17.1.4	Xanthine dehydrogenase.	NF	NF	NF	FOUND
534	1.17.3.2	Xanthine oxidase.	NF	NF	NF	NF
535	1.17.4.1	Ribonucleoside-diphosphate reductase.	FOUND	FOUND	FOUND	FOUND
536	1.17.4.2	Ribonucleoside-triphosphate reductase.	FOUND	FOUND	FOUND	FOUND
537	1.7.1.7	GMP reductase.	NF	NF	NF	NF
538	1.7.3.3	Factor independent urate hydroxylase.	NF	NF	NF	NF
539	2.1.2.2	Phosphoribosylglycinamide formyltransferase.	NF	FOUND	FOUND	FOUND
540	2.1.2.3	Phosphoribosylaminoimidazolecarboxamide formyl	NF	NF	NF	NF
541	2.1.2.4	Glycine formimidoyltransferase.	NF	NF	NF	NF
542	2.1.3.5	Oxamate carbamoyltransferase.	NF	NF	NF	NF
543	2.4.2.-	Pentosyltransferases.	FOUND	FOUND	FOUND	FOUND
544	2.4.2.1	Purine-nucleoside phosphorylase.	FOUND	FOUND	FOUND	FOUND

545	2.4.2.14	Amidophosphoribosyltransferase.	NF	FOUND	FOUND	FOUND
546	2.4.2.15	Guanosine phosphorylase.	NF	NF	NF	NF
547	2.4.2.16	Urate-ribonucleotide phosphorylase.	NF	NF	NF	NF
548	2.4.2.22	Xanthine phosphoribosyltransferase.	NF	NF	NF	FOUND
549	2.4.2.7	Adenine phosphoribosyltransferase.	FOUND	FOUND	FOUND	FOUND
550	2.4.2.8	Hypoxanthine phosphoribosyltransferase.	FOUND	FOUND	FOUND	FOUND
551	2.7.1.113	Deoxyguanosine kinase.	NF	NF	NF	NF
552	2.7.1.20	Adenosine kinase.	NF	NF	NF	NF
553	2.7.1.25	Adenylyl-sulfate kinase.	NF	NF	NF	NF
554	2.7.1.40	Pyruvate kinase.	FOUND	FOUND	FOUND	FOUND
555	2.7.1.73	Inosine kinase.	NF	NF	NF	NF
556	2.7.1.74	Deoxycytidine kinase.	NF	NF	NF	NF
557	2.7.1.76	Deoxyadenosine kinase.	FOUND	FOUND	FOUND	FOUND
558	2.7.2.2	Carbamate kinase.	FOUND	FOUND	NF	FOUND
559	2.7.4.11	(Deoxy)adenylate kinase.	NF	NF	NF	NF
560	2.7.4.3	Adenylate kinase.	FOUND	FOUND	FOUND	FOUND
561	2.7.4.6	Nucleoside-diphosphate kinase.	NF	NF	NF	FOUND
562	2.7.4.8	Guanylate kinase.	FOUND	FOUND	FOUND	FOUND
563	2.7.6.1	Ribose-phosphate diphosphokinase.	FOUND	FOUND	FOUND	FOUND
564	2.7.6.5	GTP diphosphokinase.	FOUND	FOUND	FOUND	FOUND
565	2.7.7.4	Sulfate adenylyltransferase.	NF	NF	FOUND	NF
566	2.7.7.48	RNA-directed RNA polymerase.	NF	NF	NF	NF
567	2.7.7.53	ATP adenylyltransferase.	NF	NF	NF	NF
568	2.7.7.6	DNA-directed RNA polymerase.	FOUND	FOUND	FOUND	FOUND
569	2.7.7.7	DNA-directed DNA polymerase.	FOUND	FOUND	FOUND	FOUND
570	2.7.7.8	Polyribonucleotide nucleotidyltransferase.	FOUND	FOUND	FOUND	FOUND
571	3.1.3.5	5'-nucleotidase.	FOUND	FOUND	FOUND	FOUND
572	3.1.3.6	3'-nucleotidase.	NF	NF	NF	NF
573	3.1.4.16	2',3'-cyclic-nucleotide 2'-phosphodiesterase.	FOUND	FOUND	FOUND	FOUND
574	3.1.4.17	3',5'-cyclic-nucleotide phosphodiesterase.	NF	NF	NF	FOUND
575	3.1.4.35	3',5'-cyclic-GMP phosphodiesterase.	NF	NF	NF	NF
576	3.1.4.53	3',5'-cyclic-AMP phosphodiesterase.	NF	NF	NF	NF
577	3.1.5.1	dGTPase.	NF	FOUND	NF	FOUND
578	3.1.7.2	Guanosine-3',5'-bis(diphosphate) 3'-diphosphatase.	NF	NF	NF	NF
579	3.2.2.1	Purine nucleosidase.	NF	FOUND	FOUND	FOUND
580	3.2.2.12	Inosinate nucleosidase.	NF	NF	NF	NF
581	3.2.2.2	Inosine nucleosidase.	NF	NF	NF	NF
582	3.2.2.4	AMP nucleosidase.	NF	NF	NF	NF
583	3.2.2.7	Adenosine nucleosidase.	NF	NF	NF	NF
584	3.2.2.8	Ribosylpyrimidine nucleosidase.	NF	NF	NF	NF
585	3.5.1.5	Urease.	NF	NF	NF	NF
586	3.5.2.-	In cyclic amides.	NF	NF	FOUND	FOUND
587	3.5.2.17	Hydroxyisourate hydrolase.	NF	NF	NF	NF
588	3.5.2.5	Allantoinase.	NF	NF	NF	NF
589	3.5.3.-	In linear amidines.	NF	NF	FOUND	FOUND
590	3.5.3.19	Ureidoglycolate hydrolase.	NF	NF	NF	NF
591	3.5.3.4	Allantoicase.	NF	NF	NF	NF
592	3.5.3.9	Allantoate deiminase.	NF	NF	NF	NF
593	3.5.4.10	IMP cyclohydrolase.	NF	FOUND	FOUND	FOUND
594	3.5.4.2	Adenine deaminase.	NF	NF	NF	FOUND
595	3.5.4.3	Guanine deaminase.	NF	NF	NF	FOUND
596	3.5.4.4	Adenosine deaminase.	NF	FOUND	NF	NF
597	3.5.4.6	AMP deaminase.	NF	NF	NF	NF
598	3.5.4.8	Aminoimidazolase.	NF	NF	NF	NF
599	3.6.1.-	In phosphorous-containing anhydrides.	FOUND	FOUND	FOUND	FOUND

600	3.6.1.11	Exopolyphosphatase.	FOUND	FOUND	NF	FOUND
601	3.6.1.13	ADP-ribose diphosphatase.	NF	NF	NF	NF
602	3.6.1.14	Adenosine-tetraphosphatase.	NF	NF	NF	NF
603	3.6.1.15	Nucleoside-triphosphatase.	FOUND	FOUND	FOUND	FOUND
604	3.6.1.17	Bis(5'-nucleosyl)-tetraphosphatase (asymmetrical).	FOUND	NF	NF	FOUND
605	3.6.1.19	Nucleoside-triphosphate diphosphatase.	NF	NF	NF	NF
606	3.6.1.20	5'-acylphosphoadenosine hydrolase.	NF	NF	NF	NF
607	3.6.1.21	ADP-sugar diphosphatase.	NF	NF	NF	NF
608	3.6.1.29	Bis(5'-adenosyl)-triphosphatase.	NF	NF	NF	NF
609	3.6.1.3	Adenosinetriphosphatase.	FOUND	FOUND	FOUND	FOUND
610	3.6.1.40	Guanosine-5'-triphosphate,3'-diphosphate diphospl	NF	NF	NF	NF
611	3.6.1.41	Bis(5'-nucleosyl)-tetraphosphatase (symmetrical).	NF	NF	NF	NF
612	3.6.1.5	Apyrase.	NF	NF	NF	NF
613	3.6.1.6	Nucleoside-diphosphatase.	NF	NF	NF	NF
614	3.6.1.8	ATP diphosphatase.	NF	NF	NF	NF
615	3.6.1.9	Nucleotide diphosphatase.	NF	NF	NF	NF
616	3.6.4.1	Myosin ATPase.	NF	NF	NF	NF
617	4.1.1.-	Carboxy-lyases.	FOUND	FOUND	FOUND	FOUND
618	4.1.1.21	Phosphoribosylaminoimidazole carboxylase.	NF	FOUND	FOUND	FOUND
619	4.3.2.2	Adenylosuccinate lyase.	FOUND	FOUND	FOUND	FOUND
620	4.3.2.3	Ureidoglycolate lyase.	NF	NF	NF	NF
621	4.6.1.1	Adenylate cyclase.	NF	NF	NF	NF
622	4.6.1.2	Guanylate cyclase.	NF	NF	NF	NF
623	5.1.99.3	Allantoin racemase.	NF	NF	NF	NF
624	5.4.2.2	Phosphoglucomutase.	FOUND	FOUND	FOUND	FOUND
625	5.4.2.7	Phosphopentomutase.	FOUND	NF	FOUND	FOUND
626	5.4.99.18	5-(carboxyamino)imidazole ribonucleotide mutase.	NF	NF	NF	NF
627	6.3.2.6	Phosphoribosylaminoimidazolesuccinocarboxamide	NF	FOUND	FOUND	FOUND
628	6.3.3.1	Phosphoribosylformylglycinamidine cyclo-ligase.	NF	FOUND	FOUND	FOUND
629	6.3.4.-	Other carbon--nitrogen ligases.	FOUND	FOUND	FOUND	FOUND
630	6.3.4.1	GMP synthase.	NF	NF	NF	NF
631	6.3.4.13	Phosphoribosylamine--glycine ligase.	NF	FOUND	FOUND	FOUND
632	6.3.4.18	5-(carboxyamino)imidazole ribonucleotide synthase	NF	NF	NF	NF
633	6.3.4.4	Adenylosuccinate synthase.	FOUND	FOUND	FOUND	FOUND
634	6.3.4.7	Ribose-5-phosphate--ammonia ligase.	NF	NF	NF	NF
635	6.3.5.2	GMP synthase (glutamine-hydrolyzing).	FOUND	FOUND	FOUND	FOUND
636	6.3.5.3	Phosphoribosylformylglycinamidine synthase.	NF	FOUND	FOUND	FOUND
637	>Puromycin biosynthesis 00231					
638	1.-.-.-	Oxidoreductases.	FOUND	FOUND	FOUND	FOUND
639	2.-.-.-	Transferases.	FOUND	FOUND	FOUND	FOUND
640	2.1.1.38	O-demethylpuromycin O-methyltransferase.	NF	NF	NF	NF
641	2.3.-.-	Acyltransferases.	FOUND	FOUND	FOUND	FOUND
642	3.-.-.-	Hydrolases.	FOUND	FOUND	FOUND	FOUND
643	>Caffeine metabolism 00232					
644	1.13.12.-	With incorporation of one atom of oxygen.	NF	NF	FOUND	NF
645	1.14.14.1	Unspecific monooxygenase.	NF	NF	NF	NF
646	1.17.3.2	Xanthine oxidase.	NF	NF	NF	NF
647	1.17.5.-	With a quinone or similar compound as acceptor.	NF	NF	NF	NF
648	1.7.3.3	Factor independent urate hydroxylase.	NF	NF	NF	NF
649	2.1.1.158	7-methylxanthosine synthase.	NF	NF	NF	NF
650	2.1.1.159	Theobromine synthase.	NF	NF	NF	NF
651	2.1.1.160	Caffeine synthase.	NF	NF	NF	NF
652	2.3.1.5	Arylamine N-acetyltransferase.	NF	NF	NF	NF
653	3.2.2.25	N-methyl nucleosidase.	NF	NF	NF	NF
654	>Pyrimidine metabolism 00240					

655	1.17.4.1	Ribonucleoside-diphosphate reductase.	FOUND	FOUND	FOUND	FOUND
656	1.17.4.2	Ribonucleoside-triphosphate reductase.	FOUND	FOUND	FOUND	FOUND
657	1.17.99.4	Uracil/thymine dehydrogenase.	NF	NF	NF	NF
658	1.3.1.1	Dihydrouracil dehydrogenase (NAD(+)).	NF	NF	NF	NF
659	1.3.1.14	Orotate reductase (NADH).	NF	NF	NF	NF
660	1.3.1.2	Dihydropyrimidine dehydrogenase (NADP(+)).	NF	NF	NF	NF
661	1.3.3.1	Dihydroorotate oxidase.	NF	NF	NF	FOUND
662	1.3.5.2	Dihydroorotate dehydrogenase.	NF	NF	FOUND	FOUND
663	1.8.1.9	Thioredoxin-disulfide reductase.	NF	NF	FOUND	NF
664	2.1.1.148	Thymidylate synthase (FAD).	NF	NF	NF	NF
665	2.1.1.45	Thymidylate synthase.	FOUND	NF	FOUND	FOUND
666	2.1.2.8	Deoxycytidylate 5-hydroxymethyltransferase.	NF	NF	NF	NF
667	2.1.3.2	Aspartate carbamoyltransferase.	NF	NF	FOUND	FOUND
668	2.4.2.1	Purine-nucleoside phosphorylase.	FOUND	FOUND	FOUND	FOUND
669	2.4.2.10	Orotate phosphoribosyltransferase.	NF	NF	FOUND	FOUND
670	2.4.2.2	Pyrimidine-nucleoside phosphorylase.	FOUND	FOUND	FOUND	FOUND
671	2.4.2.23	Deoxyuridine phosphorylase.	NF	NF	NF	NF
672	2.4.2.3	Uridine phosphorylase.	NF	NF	NF	FOUND
673	2.4.2.4	Thymidine phosphorylase.	FOUND	FOUND	FOUND	FOUND
674	2.4.2.6	Nucleoside deoxyribosyltransferase.	FOUND	FOUND	NF	NF
675	2.4.2.9	Uracil phosphoribosyltransferase.	FOUND	FOUND	NF	FOUND
676	2.7.1.21	Thymidine kinase.	FOUND	FOUND	FOUND	FOUND
677	2.7.1.48	Uridine kinase.	FOUND	FOUND	FOUND	FOUND
678	2.7.1.74	Deoxycytidine kinase.	NF	NF	NF	NF
679	2.7.1.83	Pseudouridine kinase.	NF	NF	NF	NF
680	2.7.4.10	Nucleoside-triphosphate--adenylate kinase.	NF	NF	NF	NF
681	2.7.4.14	Cytidylate kinase.	FOUND	FOUND	FOUND	FOUND
682	2.7.4.22	UMP kinase.	NF	NF	NF	NF
683	2.7.4.4	Nucleoside-phosphate kinase.	NF	NF	NF	NF
684	2.7.4.6	Nucleoside-diphosphate kinase.	NF	NF	NF	FOUND
685	2.7.4.9	dTMP kinase.	FOUND	FOUND	FOUND	FOUND
686	2.7.7.6	DNA-directed RNA polymerase.	FOUND	FOUND	FOUND	FOUND
687	2.7.7.7	DNA-directed DNA polymerase.	FOUND	FOUND	FOUND	FOUND
688	2.7.7.8	Polyribonucleotide nucleotidyltransferase.	FOUND	FOUND	FOUND	FOUND
689	3.1.3.5	5'-nucleotidase.	FOUND	FOUND	FOUND	FOUND
690	3.1.3.6	3'-nucleotidase.	NF	NF	NF	NF
691	3.1.4.16	2',3'-cyclic-nucleotide 2'-phosphodiesterase.	FOUND	FOUND	FOUND	FOUND
692	3.2.2.10	Pyrimidine-5'-nucleotide nucleosidase.	NF	NF	NF	NF
693	3.2.2.3	Uridine nucleosidase.	NF	NF	NF	NF
694	3.2.2.8	Ribosylpyrimidine nucleosidase.	NF	NF	NF	NF
695	3.5.1.6	Beta-ureidopropionase.	NF	NF	NF	NF
696	3.5.1.95	N-malonylurea hydrolase.	NF	NF	NF	NF
697	3.5.2.1	Barbiturase.	NF	NF	NF	NF
698	3.5.2.2	Dihydropyrimidinase.	NF	NF	NF	FOUND
699	3.5.2.3	Dihydroorotase.	NF	NF	FOUND	FOUND
700	3.5.4.1	Cytosine deaminase.	NF	NF	NF	NF
701	3.5.4.12	dCMP deaminase.	FOUND	NF	NF	NF
702	3.5.4.13	dCTP deaminase.	NF	NF	NF	NF
703	3.5.4.14	Deoxycytidine deaminase.	NF	NF	NF	NF
704	3.5.4.30	dCTP deaminase (dUMP-forming).	NF	NF	NF	NF
705	3.5.4.5	Cytidine deaminase.	FOUND	FOUND	FOUND	FOUND
706	3.6.1.12	dCTP diphosphatase.	NF	NF	NF	NF
707	3.6.1.17	Bis(5'-nucleosyl)-tetraphosphatase (asymmetrical).	FOUND	NF	NF	FOUND
708	3.6.1.19	Nucleoside-triphosphate diphosphatase.	NF	NF	NF	NF
709	3.6.1.23	dUTP diphosphatase.	FOUND	FOUND	NF	FOUND

710	3.6.1.39	Thymidine-triphosphatase.	NF	NF	NF	NF
711	3.6.1.5	Apyrase.	NF	NF	NF	NF
712	3.6.1.6	Nucleoside-diphosphatase.	NF	NF	NF	NF
713	3.6.1.8	ATP diphosphatase.	NF	NF	NF	NF
714	4.1.1.23	Orotidine-5'-phosphate decarboxylase.	NF	NF	FOUND	FOUND
715	4.2.1.70	Pseudouridylate synthase.	NF	NF	NF	NF
716	6.3.4.2	CTP synthase.	NF	FOUND	FOUND	FOUND
717	6.3.5.5	Carbamoyl-phosphate synthase (glutamine-hydroly:	NF	NF	FOUND	FOUND
718	>Alanine, aspartate and glutamate metabolism 00250					
719	1.2.1.16	Succinate-semialdehyde dehydrogenase (NAD(P)(+)	NF	NF	NF	NF
720	1.2.1.24	Succinate-semialdehyde dehydrogenase (NAD(+)).	NF	NF	NF	NF
721	1.4.1.1	Alanine dehydrogenase.	NF	NF	NF	NF
722	1.4.1.13	Glutamate synthase (NADPH).	NF	NF	NF	FOUND
723	1.4.1.14	Glutamate synthase (NADH).	NF	NF	NF	FOUND
724	1.4.1.2	Glutamate dehydrogenase.	NF	NF	FOUND	NF
725	1.4.1.3	Glutamate dehydrogenase (NAD(P)(+)).	NF	NF	NF	NF
726	1.4.1.4	Glutamate dehydrogenase (NADP(+)).	NF	FOUND	NF	FOUND
727	1.4.3.1	D-aspartate oxidase.	NF	NF	NF	NF
728	1.4.3.15	D-glutamate(D-aspartate) oxidase.	NF	NF	NF	NF
729	1.4.3.16	L-aspartate oxidase.	NF	NF	NF	NF
730	1.4.3.2	L-amino-acid oxidase.	NF	NF	NF	NF
731	1.5.1.12	1-pyrroline-5-carboxylate dehydrogenase.	NF	NF	NF	NF
732	2.1.3.2	Aspartate carbamoyltransferase.	NF	NF	FOUND	FOUND
733	2.3.1.17	Aspartate N-acetyltransferase.	NF	NF	NF	NF
734	2.4.2.14	Amidophosphoribosyltransferase.	NF	FOUND	FOUND	FOUND
735	2.6.1.1	Aspartate transaminase.	FOUND	FOUND	FOUND	FOUND
736	2.6.1.12	Alanine--oxo-acid transaminase.	NF	NF	NF	NF
737	2.6.1.14	Asparagine--oxo-acid transaminase.	NF	NF	NF	NF
738	2.6.1.15	Glutamine--pyruvate transaminase.	NF	NF	NF	NF
739	2.6.1.16	Glutamine--fructose-6-phosphate transaminase (iso	FOUND	NF	FOUND	FOUND
740	2.6.1.19	4-aminobutyrate transaminase.	NF	NF	NF	FOUND
741	2.6.1.2	Alanine transaminase.	NF	NF	NF	NF
742	2.6.1.44	Alanine--glyoxylate transaminase.	NF	NF	NF	NF
743	3.5.1.1	Asparaginase.	NF	NF	NF	NF
744	3.5.1.15	Aspartoacylase.	NF	NF	NF	NF
745	3.5.1.2	Glutaminase.	NF	NF	NF	NF
746	3.5.1.3	Omega-amidase.	NF	NF	NF	NF
747	3.5.1.38	Glutamin-(asparagin-)ase.	NF	NF	NF	NF
748	3.5.1.7	Ureidosuccinase.	NF	NF	NF	NF
749	4.1.1.12	Aspartate 4-decarboxylase.	NF	NF	NF	NF
750	4.1.1.15	Glutamate decarboxylase.	NF	NF	NF	NF
751	4.3.1.1	Aspartate ammonia-lyase.	NF	NF	NF	NF
752	4.3.2.1	Argininosuccinate lyase.	NF	NF	FOUND	FOUND
753	4.3.2.2	Adenylosuccinate lyase.	FOUND	FOUND	FOUND	FOUND
754	5.1.1.13	Aspartate racemase.	NF	NF	NF	NF
755	6.3.1.1	Aspartate--ammonia ligase.	FOUND	FOUND	FOUND	FOUND
756	6.3.1.2	Glutamate--ammonia ligase.	NF	FOUND	FOUND	FOUND
757	6.3.4.16	Carbamoyl-phosphate synthase (ammonia).	NF	NF	NF	NF
758	6.3.4.4	Adenylosuccinate synthase.	FOUND	FOUND	FOUND	FOUND
759	6.3.4.5	Argininosuccinate synthase.	NF	NF	FOUND	FOUND
760	6.3.5.4	Asparagine synthase (glutamine-hydrolyzing).	NF	NF	NF	FOUND
761	6.3.5.5	Carbamoyl-phosphate synthase (glutamine-hydroly:	NF	NF	FOUND	FOUND
762	>Tetracycline biosynthesis 00253					
763	1.1.1.-	With NAD(+) or NADP(+) as acceptor.	FOUND	FOUND	FOUND	FOUND
764	1.14.-.-	Acting on paired donors, with incorporation or redu	NF	NF	NF	NF

765	1.14.13.38	Anhydrotetracycline monooxygenase.	NF	NF	NF	NF
766	1.14.14.-	With reduced flavin or flavoprotein as one donor, a	NF	NF	NF	NF
767	2.1.1.-	Methyltransferases.	FOUND	FOUND	FOUND	FOUND
768	2.3.1.-	Transferring groups other than amino-acyl groups.	FOUND	FOUND	FOUND	FOUND
769	4.2.1.-	Hydro-lyases.	FOUND	FOUND	FOUND	FOUND
770	6.3.5.-	Carbon--nitrogen ligases with glutamine as amido-N	FOUND	FOUND	FOUND	FOUND
771	6.4.1.2	Acetyl-CoA carboxylase.	NF	NF	FOUND	FOUND
772	>Glycine, serine and threonine metabolism 00260					
773	1.1.1.-	With NAD(+) or NADP(+) as acceptor.	FOUND	FOUND	FOUND	FOUND
774	1.1.1.1	Alcohol dehydrogenase.	FOUND	FOUND	FOUND	FOUND
775	1.1.1.103	L-threonine 3-dehydrogenase.	NF	NF	NF	NF
776	1.1.1.29	Glycerate dehydrogenase.	NF	NF	NF	NF
777	1.1.1.3	Homoserine dehydrogenase.	NF	NF	FOUND	FOUND
778	1.1.1.75	(R)-aminopropanol dehydrogenase.	NF	NF	NF	NF
779	1.1.1.81	Hydroxypyruvate reductase.	NF	NF	NF	NF
780	1.1.1.95	Phosphoglycerate dehydrogenase.	FOUND	NF	FOUND	FOUND
781	1.1.3.17	Choline oxidase.	NF	NF	NF	NF
782	1.1.99.1	Choline dehydrogenase.	NF	NF	NF	NF
783	1.14.11.-	With 2-oxoglutarate as one donor, and incorporatio	NF	NF	NF	NF
784	1.14.15.7	Choline monooxygenase.	NF	NF	NF	NF
785	1.2.1.11	Aspartate-semialdehyde dehydrogenase.	FOUND	FOUND	FOUND	FOUND
786	1.2.1.8	Betaine-aldehyde dehydrogenase.	NF	NF	NF	NF
787	1.4.2.1	Glycine dehydrogenase (cytochrome).	NF	NF	NF	NF
788	1.4.3.21	Primary-amine oxidase.	NF	NF	FOUND	FOUND
789	1.4.3.3	D-amino-acid oxidase.	NF	NF	NF	NF
790	1.4.3.4	Monoamine oxidase.	NF	NF	NF	NF
791	1.4.4.2	Glycine dehydrogenase (decarboxylating).	NF	NF	NF	NF
792	1.5.3.1	Sarcosine oxidase.	NF	NF	NF	NF
793	1.5.99.1	Sarcosine dehydrogenase.	NF	NF	NF	NF
794	1.5.99.2	Dimethylglycine dehydrogenase.	NF	NF	NF	NF
795	1.8.1.4	Dihydrolipoyl dehydrogenase.	FOUND	FOUND	FOUND	FOUND
796	2.1.1.2	Guanidinoacetate N-methyltransferase.	NF	NF	NF	NF
797	2.1.1.20	Glycine N-methyltransferase.	NF	NF	NF	NF
798	2.1.1.5	Betaine--homocysteine S-methyltransferase.	NF	NF	NF	NF
799	2.1.2.1	Glycine hydroxymethyltransferase.	NF	NF	FOUND	FOUND
800	2.1.2.10	Aminomethyltransferase.	NF	NF	NF	NF
801	2.1.4.1	Glycine amidinotransferase.	NF	NF	NF	NF
802	2.3.1.178	Diaminobutyrate acetyltransferase.	NF	NF	NF	NF
803	2.3.1.29	Glycine C-acetyltransferase.	NF	NF	NF	NF
804	2.3.1.37	5-aminolevulinate synthase.	NF	NF	NF	NF
805	2.6.1.4	Glycine transaminase.	NF	NF	NF	NF
806	2.6.1.44	Alanine--glyoxylate transaminase.	NF	NF	NF	NF
807	2.6.1.45	Serine--glyoxylate transaminase.	NF	NF	NF	NF
808	2.6.1.51	Serine--pyruvate transaminase.	NF	NF	FOUND	FOUND
809	2.6.1.52	Phosphoserine transaminase.	NF	NF	NF	NF
810	2.6.1.76	Diaminobutyrate--2-oxoglutarate transaminase.	NF	NF	NF	NF
811	2.7.1.31	Glycerate kinase.	FOUND	NF	NF	FOUND
812	2.7.1.39	Homoserine kinase.	NF	NF	FOUND	FOUND
813	2.7.2.4	Aspartate kinase.	FOUND	FOUND	FOUND	FOUND
814	2.7.3.5	Lombricine kinase.	NF	NF	NF	NF
815	2.7.8.8	CDP-diacylglycerol--serine O-phosphatidyltransferas	NF	NF	NF	NF
816	3.1.3.3	Phosphoserine phosphatase.	NF	NF	NF	NF
817	3.1.3.38	3-phosphoglycerate phosphatase.	NF	NF	NF	NF
818	3.5.3.3	Creatinase.	NF	NF	NF	NF
819	4.1.1.86	Diaminobutyrate decarboxylase.	NF	NF	NF	NF

820	4.1.2.5	Threonine aldolase.	NF	NF	FOUND	NF
821	4.2.1.108	Ectoine synthase.	NF	NF	NF	NF
822	4.2.1.20	Tryptophan synthase.	NF	NF	FOUND	FOUND
823	4.2.1.22	Cystathionine beta-synthase.	NF	NF	NF	NF
824	4.2.3.1	Threonine synthase.	NF	NF	FOUND	FOUND
825	4.3.1.17	L-serine ammonia-lyase.	FOUND	NF	NF	FOUND
826	4.3.1.18	D-serine ammonia-lyase.	NF	NF	NF	NF
827	4.3.1.19	Threonine ammonia-lyase.	FOUND	FOUND	FOUND	FOUND
828	4.4.1.1	Cystathionine gamma-lyase.	NF	NF	FOUND	NF
829	5.1.1.10	Amino-acid racemase.	NF	NF	NF	NF
830	5.1.1.18	Serine racemase.	NF	NF	NF	NF
831	>Cysteine and methionine metabolism 00270					
832	1.1.1.27	L-lactate dehydrogenase.	FOUND	FOUND	NF	FOUND
833	1.1.1.272	(R)-2-hydroxyacid dehydrogenase.	NF	NF	NF	NF
834	1.1.1.3	Homoserine dehydrogenase.	NF	NF	FOUND	FOUND
835	1.13.11.20	Cysteine dioxygenase.	NF	NF	NF	NF
836	1.13.11.53	Acireductone dioxygenase (Ni(2+)-requiring).	NF	NF	NF	NF
837	1.13.11.54	Acireductone dioxygenase (Fe(2+)-requiring).	NF	NF	NF	NF
838	1.14.17.4	Aminocyclopropanecarboxylate oxidase.	NF	NF	NF	NF
839	1.2.1.11	Aspartate-semialdehyde dehydrogenase.	FOUND	FOUND	FOUND	FOUND
840	1.4.1.-	With NAD(+) or NADP(+) as acceptor.	NF	FOUND	FOUND	FOUND
841	1.4.3.2	L-amino-acid oxidase.	NF	NF	NF	NF
842	1.8.1.10	CoA-glutathione reductase.	NF	NF	NF	NF
843	1.8.1.6	Cystine reductase.	NF	NF	NF	NF
844	1.8.4.1	Glutathione--homocystine transhydrogenase.	NF	NF	NF	NF
845	1.8.4.13	L-methionine (S)-S-oxide reductase.	NF	NF	NF	NF
846	1.8.4.14	L-methionine (R)-S-oxide reductase.	NF	NF	NF	NF
847	1.8.4.3	Glutathione--CoA-glutathione transhydrogenase.	NF	NF	NF	NF
848	1.8.4.4	Glutathione--cystine transhydrogenase.	NF	NF	NF	NF
849	2.1.1.10	Homocysteine S-methyltransferase.	NF	NF	NF	NF
850	2.1.1.13	Methionine synthase.	NF	NF	FOUND	FOUND
851	2.1.1.14	5-methyltetrahydropteroyltriglutamate--homocyste	FOUND	FOUND	FOUND	NF
852	2.1.1.37	DNA (cytosine-5-)-methyltransferase.	NF	FOUND	FOUND	FOUND
853	2.1.1.5	Betaine--homocysteine S-methyltransferase.	NF	NF	NF	NF
854	2.3.1.30	Serine O-acetyltransferase.	NF	NF	FOUND	FOUND
855	2.3.1.31	Homoserine O-acetyltransferase.	NF	NF	NF	NF
856	2.3.1.46	Homoserine O-succinyltransferase.	NF	NF	FOUND	FOUND
857	2.4.2.28	S-methyl-5'-thioadenosine phosphorylase.	NF	NF	NF	NF
858	2.5.1.-	Transferring alkyl or aryl groups, other than methyl	FOUND	FOUND	FOUND	FOUND
859	2.5.1.16	Spermidine synthase.	NF	NF	FOUND	NF
860	2.5.1.22	Spermine synthase.	NF	NF	NF	NF
861	2.5.1.47	Cysteine synthase.	NF	NF	FOUND	FOUND
862	2.5.1.48	Cystathionine gamma-synthase.	NF	NF	FOUND	NF
863	2.5.1.49	O-acetylhomoserine aminocarboxypropyltransferas	NF	NF	NF	FOUND
864	2.5.1.6	Methionine adenosyltransferase.	FOUND	FOUND	FOUND	FOUND
865	2.5.1.65	O-phosphoserine sulfhydrylase.	NF	NF	NF	NF
866	2.6.1.-	Transaminases (aminotransferases).	FOUND	FOUND	FOUND	FOUND
867	2.6.1.1	Aspartate transaminase.	FOUND	FOUND	FOUND	FOUND
868	2.6.1.3	Cysteine transaminase.	NF	NF	NF	NF
869	2.6.1.5	Tyrosine transaminase.	NF	NF	NF	NF
870	2.6.1.57	Aromatic-amino-acid transaminase.	NF	NF	NF	NF
871	2.7.1.100	S-methyl-5-thioribose kinase.	NF	NF	NF	NF
872	2.7.2.4	Aspartate kinase.	FOUND	FOUND	FOUND	FOUND
873	2.8.1.2	3-mercaptopyruvate sulfurtransferase.	NF	NF	NF	NF
874	3.1.3.77	Acireductone synthase.	NF	NF	NF	NF

875	3.2.2.16	Methylthioadenosine nucleosidase.	NF	NF	NF	NF
876	3.2.2.9	Adenosylhomocysteine nucleosidase.	NF	FOUND	FOUND	FOUND
877	3.3.1.1	Adenosylhomocysteinase.	NF	NF	NF	NF
878	3.4.13.12	Met-Xaa dipeptidase.	NF	NF	NF	NF
879	3.5.1.31	Formylmethionine deformylase.	NF	NF	NF	NF
880	4.1.1.12	Aspartate 4-decarboxylase.	NF	NF	NF	NF
881	4.1.1.50	Adenosylmethionine decarboxylase.	NF	NF	FOUND	NF
882	4.2.1.109	Methylthioribulose 1-phosphate dehydratase.	NF	NF	NF	NF
883	4.2.1.22	Cystathionine beta-synthase.	NF	NF	NF	NF
884	4.3.1.17	L-serine ammonia-lyase.	FOUND	NF	NF	FOUND
885	4.4.1.1	Cystathionine gamma-lyase.	NF	NF	FOUND	NF
886	4.4.1.10	Cysteine lyase.	NF	NF	NF	NF
887	4.4.1.11	Methionine gamma-lyase.	NF	NF	NF	NF
888	4.4.1.14	1-aminocyclopropane-1-carboxylate synthase.	NF	NF	NF	NF
889	4.4.1.15	D-cysteine desulfhydrase.	NF	NF	NF	NF
890	4.4.1.21	S-ribosylhomocysteine lyase.	NF	NF	NF	NF
891	4.4.1.24	Sulfolactate sulfo-lyase.	NF	NF	NF	NF
892	4.4.1.25	L-cysteate sulfo-lyase.	NF	NF	NF	NF
893	4.4.1.8	Cystathionine beta-lyase.	FOUND	NF	FOUND	NF
894	5.1.1.10	Amino-acid racemase.	NF	NF	NF	NF
895	5.3.1.23	S-methyl-5-thioribose-1-phosphate isomerase.	NF	NF	NF	NF
896	>Valine, leucine and isoleucine degradation 00280					
897	1.1.1.178	3-hydroxy-2-methylbutyryl-CoA dehydrogenase.	NF	NF	NF	NF
898	1.1.1.31	3-hydroxyisobutyrate dehydrogenase.	NF	NF	NF	NF
899	1.1.1.35	3-hydroxyacyl-CoA dehydrogenase.	NF	NF	NF	NF
900	1.2.1.27	Methylmalonate-semialdehyde dehydrogenase (acy	NF	NF	NF	FOUND
901	1.2.1.3	Aldehyde dehydrogenase (NAD(+)).	NF	NF	FOUND	FOUND
902	1.2.3.1	Aldehyde oxidase.	NF	NF	NF	NF
903	1.2.4.4	3-methyl-2-oxobutanoate dehydrogenase (2-methy	NF	NF	NF	FOUND
904	1.2.7.7	3-methyl-2-oxobutanoate dehydrogenase (ferredox	NF	NF	NF	NF
905	1.3.99.-	With other acceptors.	NF	NF	FOUND	FOUND
906	1.3.99.10	Isovaleryl-CoA dehydrogenase.	NF	NF	NF	NF
907	1.3.99.12	2-methylacyl-CoA dehydrogenase.	NF	NF	NF	NF
908	1.3.99.2	Butyryl-CoA dehydrogenase.	NF	NF	NF	NF
909	1.3.99.3	Acyl-CoA dehydrogenase.	NF	NF	FOUND	FOUND
910	1.4.1.9	Leucine dehydrogenase.	NF	NF	NF	NF
911	1.4.3.2	L-amino-acid oxidase.	NF	NF	NF	NF
912	1.8.1.4	Dihydrolipoyl dehydrogenase.	FOUND	FOUND	FOUND	FOUND
913	2.3.1.16	Acetyl-CoA C-acyltransferase.	NF	NF	NF	NF
914	2.3.1.168	Dihydrolipoyllysine-residue (2-methylpropanoyl)tra	NF	NF	NF	NF
915	2.3.1.9	Acetyl-CoA C-acetyltransferase.	FOUND	FOUND	NF	NF
916	2.3.3.10	Hydroxymethylglutaryl-CoA synthase.	FOUND	FOUND	FOUND	NF
917	2.6.1.18	Beta-alanine--pyruvate transaminase.	NF	NF	NF	NF
918	2.6.1.22	(S)-3-amino-2-methylpropionate transaminase.	NF	NF	NF	NF
919	2.6.1.42	Branched-chain-amino-acid transaminase.	NF	NF	FOUND	FOUND
920	2.6.1.6	Leucine transaminase.	NF	NF	NF	NF
921	2.8.3.5	3-oxoacid CoA-transferase.	NF	NF	NF	NF
922	3.1.2.4	3-hydroxyisobutyryl-CoA hydrolase.	NF	NF	NF	NF
923	4.1.3.4	Hydroxymethylglutaryl-CoA lyase.	NF	NF	NF	NF
924	4.2.1.17	Enoyl-CoA hydratase.	NF	NF	NF	NF
925	4.2.1.18	Methylglutaconyl-CoA hydratase.	NF	NF	NF	NF
926	5.1.99.1	Methylmalonyl-CoA epimerase.	NF	NF	NF	NF
927	5.4.3.7	Leucine 2,3-aminomutase.	NF	NF	NF	NF
928	5.4.99.2	Methylmalonyl-CoA mutase.	NF	NF	NF	NF
929	6.4.1.3	Propionyl-CoA carboxylase.	NF	NF	NF	NF

930	6.4.1.4	Methylcrotonoyl-CoA carboxylase.	NF	NF	NF	NF
931	>Geraniol degradation 00281					
932	1.1.1.183	Geraniol dehydrogenase.	NF	NF	NF	NF
933	1.1.1.35	3-hydroxyacyl-CoA dehydrogenase.	NF	NF	NF	NF
934	1.2.1.-	With NAD(+) or NADP(+) as acceptor.	FOUND	FOUND	FOUND	FOUND
935	1.3.99.-	With other acceptors.	NF	NF	FOUND	FOUND
936	2.3.1.16	Acetyl-CoA C-acyltransferase.	NF	NF	NF	NF
937	2.8.3.-	CoA-transferases.	NF	NF	NF	NF
938	4.1.3.26	3-hydroxy-3-isohexenylglutaryl-CoA lyase.	NF	NF	NF	NF
939	4.1.3.4	Hydroxymethylglutaryl-CoA lyase.	NF	NF	NF	NF
940	4.2.1.17	Enoyl-CoA hydratase.	NF	NF	NF	NF
941	4.2.1.57	Isohexenylglutaconyl-CoA hydratase.	NF	NF	NF	NF
942	5.2.1.-	Cis-trans Isomerases.	FOUND	FOUND	FOUND	FOUND
943	6.2.1.-	Acid--thiol ligases.	NF	NF	NF	NF
944	6.4.1.5	Geranoyl-CoA carboxylase.	NF	NF	NF	NF
945	>Valine, leucine and isoleucine biosynthesis 00290					
946	1.1.1.85	3-isopropylmalate dehydrogenase.	NF	NF	FOUND	FOUND
947	1.1.1.86	Ketol-acid reductoisomerase.	NF	NF	FOUND	FOUND
948	1.2.4.1	Pyruvate dehydrogenase (acetyl-transferring).	FOUND	FOUND	FOUND	FOUND
949	1.4.1.9	Leucine dehydrogenase.	NF	NF	NF	NF
950	2.2.1.6	Acetolactate synthase.	NF	NF	FOUND	FOUND
951	2.3.1.182	(R)-citramalate synthase.	NF	NF	NF	NF
952	2.3.3.13	2-isopropylmalate synthase.	NF	NF	FOUND	FOUND
953	2.6.1.42	Branched-chain-amino-acid transaminase.	NF	NF	FOUND	FOUND
954	2.6.1.6	Leucine transaminase.	NF	NF	NF	NF
955	2.6.1.66	Valine--pyruvate transaminase.	NF	NF	NF	NF
956	4.2.1.33	3-isopropylmalate dehydratase.	NF	NF	FOUND	FOUND
957	4.2.1.35	(R)-2-methylmalate dehydratase.	NF	NF	NF	NF
958	4.2.1.9	Dihydroxy-acid dehydratase.	NF	NF	FOUND	FOUND
959	4.3.1.19	Threonine ammonia-lyase.	FOUND	FOUND	FOUND	FOUND
960	5.4.99.3	2-acetolactate mutase.	NF	NF	NF	NF
961	6.1.1.4	Leucine--tRNA ligase.	FOUND	FOUND	FOUND	FOUND
962	6.1.1.5	Isoleucine--tRNA ligase.	FOUND	FOUND	FOUND	FOUND
963	6.1.1.9	Valine--tRNA ligase.	FOUND	FOUND	FOUND	FOUND
964	>Lysine biosynthesis 00300					
965	1.1.1.3	Homoserine dehydrogenase.	NF	NF	FOUND	FOUND
966	1.1.1.87	Homoisocitrate dehydrogenase.	NF	NF	NF	NF
967	1.2.1.-	With NAD(+) or NADP(+) as acceptor.	FOUND	FOUND	FOUND	FOUND
968	1.2.1.11	Aspartate-semialdehyde dehydrogenase.	FOUND	FOUND	FOUND	FOUND
969	1.2.1.31	L-aminoadipate-semialdehyde dehydrogenase.	NF	NF	NF	NF
970	1.3.1.26	Dihydrodipicolinate reductase.	FOUND	FOUND	FOUND	FOUND
971	1.4.1.16	Diaminopimelate dehydrogenase.	NF	NF	NF	NF
972	1.5.1.10	Saccharopine dehydrogenase (NADP(+), L-glutamate	NF	NF	NF	NF
973	1.5.1.7	Saccharopine dehydrogenase (NAD(+), L-lysine-form	NF	NF	FOUND	NF
974	1.5.1.8	Saccharopine dehydrogenase (NADP(+), L-lysine-for	NF	NF	NF	NF
975	2.3.1.117	2,3,4,5-tetrahydropyridine-2,6-dicarboxylate N-succ	NF	NF	NF	NF
976	2.3.1.89	Tetrahydrodipicolinate N-acetyltransferase.	NF	NF	NF	NF
977	2.3.3.14	Homocitrate synthase.	NF	NF	NF	NF
978	2.6.1.-	Transaminases (aminotransferases).	FOUND	FOUND	FOUND	FOUND
979	2.6.1.11	Acetylornithine transaminase.	NF	NF	FOUND	FOUND
980	2.6.1.17	Succinyldiaminopimelate transaminase.	NF	NF	NF	NF
981	2.6.1.36	L-lysine 6-transaminase.	NF	NF	NF	NF
982	2.6.1.39	2-aminoadipate transaminase.	NF	NF	NF	NF
983	2.6.1.57	Aromatic-amino-acid transaminase.	NF	NF	NF	NF
984	2.6.1.83	LL-diaminopimelate aminotransferase.	NF	NF	NF	NF

985	2.7.2.-	Phosphotransferases with a carboxyl group as accep	FOUND	FOUND	FOUND	FOUND
986	2.7.2.4	Aspartate kinase.	FOUND	FOUND	FOUND	FOUND
987	3.5.1.-	In linear amides.	FOUND	FOUND	FOUND	FOUND
988	3.5.1.18	Succinyl-diaminopimelate desuccinylase.	NF	NF	NF	FOUND
989	3.5.1.47	N-acetyldiaminopimelate deacetylase.	NF	NF	NF	NF
990	4.1.1.20	Diaminopimelate decarboxylase.	NF	NF	FOUND	FOUND
991	4.2.1.36	Homoaconitate hydratase.	NF	NF	NF	NF
992	4.2.1.52	Dihydrodipicolinate synthase.	FOUND	FOUND	FOUND	FOUND
993	5.1.1.7	Diaminopimelate epimerase.	FOUND	FOUND	FOUND	FOUND
994	6.3.2.10	UDP-N-acetylmuramoyl-tripeptide--D-alanyl-D-alani	NF	NF	NF	NF
995	6.3.2.13	UDP-N-acetylmuramoyl-L-alanyl-D-glutamate--2,6-d	NF	NF	FOUND	NF
996	>Lysine degradation 00310					
997	1.1.1.35	3-hydroxyacyl-CoA dehydrogenase.	NF	NF	NF	NF
998	1.13.12.2	Lysine 2-monooxygenase.	NF	NF	NF	NF
999	1.14.11.1	Gamma-butyrobetaine dioxygenase.	NF	NF	NF	NF
1000	1.14.11.4	Procollagen-lysine 5-dioxygenase.	NF	NF	NF	NF
1001	1.14.11.8	Trimethyllysine dioxygenase.	NF	NF	NF	NF
1002	1.14.13.59	L-lysine 6-monooxygenase (NADPH).	NF	NF	NF	NF
1003	1.2.1.20	Glutarate-semialdehyde dehydrogenase.	NF	NF	NF	NF
1004	1.2.1.3	Aldehyde dehydrogenase (NAD(+)).	NF	NF	FOUND	FOUND
1005	1.2.1.31	L-aminoadipate-semialdehyde dehydrogenase.	NF	NF	NF	NF
1006	1.2.1.47	4-trimethylammoniobutyraldehyde dehydrogenase	NF	NF	NF	NF
1007	1.2.4.-	With a disulfide as acceptor.	FOUND	FOUND	FOUND	FOUND
1008	1.2.4.2	Oxoglutarate dehydrogenase (succinyl-transferring)	NF	NF	NF	NF
1009	1.3.99.7	Glutaryl-CoA dehydrogenase.	NF	NF	NF	NF
1010	1.4.1.11	L-erythro-3,5-diaminohexanoate dehydrogenase.	NF	NF	NF	NF
1011	1.4.1.12	2,4-diaminopentanoate dehydrogenase.	NF	NF	NF	NF
1012	1.4.3.14	L-lysine oxidase.	NF	NF	NF	NF
1013	1.5.1.1	Pyrroline-2-carboxylate reductase.	NF	NF	NF	NF
1014	1.5.1.10	Saccharopine dehydrogenase (NADP(+), L-glutamate	NF	NF	NF	NF
1015	1.5.1.16	D-lysopine dehydrogenase.	NF	NF	NF	NF
1016	1.5.1.21	Delta(1)-piperideine-2-carboxylate reductase.	NF	NF	NF	NF
1017	1.5.1.7	Saccharopine dehydrogenase (NAD(+), L-lysine-form	NF	NF	FOUND	NF
1018	1.5.1.8	Saccharopine dehydrogenase (NADP(+), L-lysine-for	NF	NF	NF	NF
1019	1.5.1.9	Saccharopine dehydrogenase (NAD(+), L-glutamate-	NF	NF	NF	NF
1020	1.5.3.7	L-pipecolate oxidase.	NF	NF	NF	NF
1021	1.5.99.3	L-pipecolate dehydrogenase.	NF	NF	NF	NF
1022	2.1.1.43	Histone-lysine N-methyltransferase.	NF	NF	NF	NF
1023	2.1.1.59	[Cytochrome c]-lysine N-methyltransferase.	NF	NF	NF	NF
1024	2.1.1.60	Calmodulin-lysine N-methyltransferase.	NF	NF	NF	NF
1025	2.3.1.-	Transferring groups other than amino-acyl groups.	FOUND	FOUND	FOUND	FOUND
1026	2.3.1.102	N(6)-hydroxylysine O-acetyltransferase.	NF	NF	NF	NF
1027	2.3.1.32	Lysine N-acetyltransferase.	NF	NF	NF	NF
1028	2.3.1.61	Dihydrolipoyllysine-residue succinyltransferase.	FOUND	NF	FOUND	NF
1029	2.3.1.9	Acetyl-CoA C-acetyltransferase.	FOUND	FOUND	NF	NF
1030	2.4.1.50	Procollagen galactosyltransferase.	NF	NF	NF	NF
1031	2.6.1.-	Transaminases (aminotransferases).	FOUND	FOUND	FOUND	FOUND
1032	2.6.1.21	D-amino-acid transaminase.	NF	NF	NF	NF
1033	2.6.1.39	2-aminoadipate transaminase.	NF	NF	NF	NF
1034	2.6.1.48	5-aminovalerate transaminase.	NF	NF	NF	NF
1035	2.6.1.65	N(6)-acetyl-beta-lysine transaminase.	NF	NF	NF	NF
1036	2.7.1.81	Hydroxylysine kinase.	NF	NF	NF	NF
1037	3.4.-.-	Acting on peptide bonds (peptide hydrolases).	FOUND	FOUND	FOUND	FOUND
1038	3.5.1.17	Acyl-lysine deacylase.	NF	NF	NF	NF
1039	3.5.1.30	5-aminopentanamidase.	NF	NF	NF	NF

1040	3.5.1.63	4-acetamidobutyrate deacetylase.	NF	NF	NF	NF
1041	4.1.1.18	Lysine decarboxylase.	NF	NF	NF	NF
1042	4.1.2.-	Aldehyde-lyases.	FOUND	FOUND	FOUND	FOUND
1043	4.2.1.17	Enoyl-CoA hydratase.	NF	NF	NF	NF
1044	5.1.1.5	Lysine racemase.	NF	NF	NF	NF
1045	5.1.1.9	Arginine racemase.	NF	NF	NF	NF
1046	5.4.3.2	Lysine 2,3-aminomutase.	NF	NF	NF	NF
1047	5.4.3.3	Beta-lysine 5,6-aminomutase.	NF	NF	NF	NF
1048	5.4.3.4	D-lysine 5,6-aminomutase.	NF	NF	NF	NF
1049	6.2.1.6	Glutarate--CoA ligase.	NF	NF	NF	NF
1050	6.3.2.27	Aerobactin synthase.	NF	NF	NF	NF
1051	>Penicillin and cephalosporin biosynthesis 00311					
1052	1.14.11.26	Deacetoxycephalosporin-C hydroxylase.	NF	NF	NF	NF
1053	1.14.20.1	Deacetoxycephalosporin-C synthase.	NF	NF	NF	NF
1054	1.21.3.1	Isopenicillin-N synthase.	NF	NF	NF	NF
1055	1.4.3.3	D-amino-acid oxidase.	NF	NF	NF	NF
1056	2.1.3.-	Carboxyl- and carbamoyltransferases.	FOUND	FOUND	FOUND	FOUND
1057	2.3.1.164	Isopenicillin-N N-acyltransferase.	NF	NF	NF	NF
1058	2.3.1.175	Deacetylcephalosporin-C acetyltransferase.	NF	NF	NF	NF
1059	2.6.1.74	Cephalosporin-C transaminase.	NF	NF	NF	NF
1060	3.1.1.41	Cephalosporin-C deacetylase.	NF	NF	NF	NF
1061	3.5.1.11	Penicillin amidase.	NF	NF	NF	NF
1062	3.5.1.93	Glutaryl-7-aminocephalosporanic-acid acylase.	NF	NF	NF	NF
1063	3.5.2.6	Beta-lactamase.	NF	NF	NF	FOUND
1064	5.1.1.17	Isopenicillin-N epimerase.	NF	NF	NF	NF
1065	6.3.2.26	N-(5-amino-5-carboxypentanoyl)-L-cysteinyl-D-valin	NF	NF	NF	NF
1066	>beta-Lactam resistance 00312					
1067	3.5.2.6	Beta-lactamase.	NF	NF	NF	FOUND
1068	>Arginine and proline metabolism 00330					
1069	1.1.1.104	4-oxoproline reductase.	NF	NF	NF	NF
1070	1.13.12.1	Arginine 2-monooxygenase.	NF	NF	NF	NF
1071	1.14.11.2	Procollagen-proline dioxygenase.	NF	NF	NF	NF
1072	1.14.13.39	Nitric-oxide synthase.	NF	NF	NF	NF
1073	1.2.1.-	With NAD(+) or NADP(+) as acceptor.	FOUND	FOUND	FOUND	FOUND
1074	1.2.1.19	Aminobutyraldehyde dehydrogenase.	NF	NF	NF	NF
1075	1.2.1.3	Aldehyde dehydrogenase (NAD(+)).	NF	NF	FOUND	FOUND
1076	1.2.1.38	N-acetyl-gamma-glutamyl-phosphate reductase.	NF	NF	FOUND	FOUND
1077	1.2.1.41	Glutamate-5-semialdehyde dehydrogenase.	NF	NF	FOUND	FOUND
1078	1.2.1.54	Gamma-guanidinobutyraldehyde dehydrogenase.	NF	NF	NF	NF
1079	1.2.1.71	Succinylglutamate-semialdehyde dehydrogenase.	NF	NF	NF	NF
1080	1.21.4.1	D-proline reductase (dithiol).	NF	NF	NF	NF
1081	1.4.1.12	2,4-diaminopentanoate dehydrogenase.	NF	NF	NF	NF
1082	1.4.1.2	Glutamate dehydrogenase.	NF	NF	FOUND	NF
1083	1.4.1.3	Glutamate dehydrogenase (NAD(P)(+)).	NF	NF	NF	NF
1084	1.4.1.4	Glutamate dehydrogenase (NADP(+)).	NF	FOUND	NF	FOUND
1085	1.4.3.-	With oxygen as acceptor.	NF	NF	FOUND	FOUND
1086	1.4.3.10	Putrescine oxidase.	NF	NF	NF	NF
1087	1.4.3.22	Diamine oxidase.	NF	NF	NF	NF
1088	1.4.3.3	D-amino-acid oxidase.	NF	NF	NF	NF
1089	1.4.3.4	Monoamine oxidase.	NF	NF	NF	NF
1090	1.5.-.-	Acting on the CH-NH group of donors.	FOUND	FOUND	FOUND	FOUND
1091	1.5.1.1	Pyrroline-2-carboxylate reductase.	NF	NF	NF	NF
1092	1.5.1.11	D-octopine dehydrogenase.	NF	NF	NF	NF
1093	1.5.1.12	1-pyrroline-5-carboxylate dehydrogenase.	NF	NF	NF	NF
1094	1.5.1.19	D-nopaline dehydrogenase.	NF	NF	NF	NF

1095	1.5.1.2	Pyrroline-5-carboxylate reductase.	NF	NF	FOUND	FOUND
1096	1.5.99.6	Spermidine dehydrogenase.	NF	NF	NF	NF
1097	1.5.99.8	Proline dehydrogenase.	NF	NF	NF	NF
1098	2.1.1.2	Guanidinoacetate N-methyltransferase.	NF	NF	NF	NF
1099	2.1.3.11	N-succinylornithine carbamoyltransferase.	NF	NF	NF	NF
1100	2.1.3.3	Ornithine carbamoyltransferase.	FOUND	FOUND	FOUND	FOUND
1101	2.1.3.9	N-acetylornithine carbamoyltransferase.	NF	NF	NF	NF
1102	2.1.4.1	Glycine amidinotransferase.	NF	NF	NF	NF
1103	2.3.1.-	Transferring groups other than amino-acyl groups.	FOUND	FOUND	FOUND	FOUND
1104	2.3.1.1	Amino-acid N-acetyltransferase.	NF	NF	FOUND	FOUND
1105	2.3.1.109	Arginine N-succinyltransferase.	NF	NF	NF	NF
1106	2.3.1.35	Glutamate N-acetyltransferase.	NF	NF	NF	NF
1107	2.3.1.57	Diamine N-acetyltransferase.	NF	NF	NF	NF
1108	2.3.1.64	Agmatine N(4)-coumaroyltransferase.	NF	NF	NF	NF
1109	2.5.1.16	Spermidine synthase.	NF	NF	FOUND	NF
1110	2.5.1.22	Spermine synthase.	NF	NF	NF	NF
1111	2.6.1.-	Transaminases (aminotransferases).	FOUND	FOUND	FOUND	FOUND
1112	2.6.1.1	Aspartate transaminase.	FOUND	FOUND	FOUND	FOUND
1113	2.6.1.11	Acetylornithine transaminase.	NF	NF	FOUND	FOUND
1114	2.6.1.13	Ornithine aminotransferase.	NF	NF	NF	NF
1115	2.6.1.21	D-amino-acid transaminase.	NF	NF	NF	NF
1116	2.6.1.23	4-hydroxyglutamate transaminase.	NF	NF	NF	NF
1117	2.6.1.29	Diamine transaminase.	NF	NF	NF	NF
1118	2.6.1.81	Succinylornithine transaminase.	NF	NF	NF	NF
1119	2.6.1.82	Putrescine aminotransferase.	NF	NF	NF	NF
1120	2.6.1.84	Arginine--pyruvate transaminase.	NF	NF	NF	NF
1121	2.7.2.11	Glutamate 5-kinase.	NF	NF	FOUND	FOUND
1122	2.7.2.2	Carbamate kinase.	FOUND	FOUND	NF	FOUND
1123	2.7.2.8	Acetylglutamate kinase.	NF	NF	FOUND	FOUND
1124	2.7.3.1	Guanidinoacetate kinase.	NF	NF	NF	NF
1125	2.7.3.2	Creatine kinase.	NF	NF	NF	NF
1126	2.7.3.3	Arginine kinase.	NF	NF	NF	NF
1127	3.4.11.5	Prolyl aminopeptidase.	NF	NF	NF	NF
1128	3.4.13.3	Xaa-His dipeptidase.	NF	NF	NF	NF
1129	3.5.1.14	Aminoacylase.	FOUND	FOUND	FOUND	FOUND
1130	3.5.1.16	Acetylornithine deacetylase.	NF	NF	NF	NF
1131	3.5.1.2	Glutaminase.	NF	NF	NF	NF
1132	3.5.1.38	Glutamin-(asparagin-)ase.	NF	NF	NF	NF
1133	3.5.1.4	Amidase.	NF	FOUND	NF	NF
1134	3.5.1.5	Urease.	NF	NF	NF	NF
1135	3.5.1.53	N-carbamoylputrescine amidase.	NF	NF	NF	NF
1136	3.5.1.54	Allophanate hydrolase.	NF	NF	NF	NF
1137	3.5.1.59	N-carbamoylsarcosine amidase.	NF	NF	NF	NF
1138	3.5.1.62	Acetylputrescine deacetylase.	NF	NF	NF	NF
1139	3.5.1.63	4-acetamidobutyrate deacetylase.	NF	NF	NF	NF
1140	3.5.1.94	Gamma-glutamyl-gamma-aminobutyrate hydrolase	NF	NF	NF	NF
1141	3.5.1.96	Succinylglutamate desuccinylase.	NF	NF	NF	NF
1142	3.5.2.10	Creatininase.	NF	NF	NF	NF
1143	3.5.2.14	N-methylhydantoinase (ATP-hydrolyzing).	NF	NF	NF	NF
1144	3.5.3.1	Arginase.	NF	NF	NF	NF
1145	3.5.3.11	Agmatinase.	NF	NF	FOUND	NF
1146	3.5.3.12	Agmatine deiminase.	NF	NF	NF	FOUND
1147	3.5.3.23	N-succinylarginine dihydrolase.	NF	NF	NF	NF
1148	3.5.3.3	Creatinase.	NF	NF	NF	NF
1149	3.5.3.6	Arginine deiminase.	NF	NF	NF	NF

1150	3.5.3.7	Guanidinobutyrase.	NF	NF	NF	NF
1151	3.5.4.1	Cytosine deaminase.	NF	NF	NF	NF
1152	3.5.4.21	Creatinine deaminase.	NF	NF	NF	NF
1153	3.5.4.22	1-pyrroline-4-hydroxy-2-carboxylate deaminase.	NF	NF	NF	NF
1154	4.1.1.-	Carboxy-lyases.	FOUND	FOUND	FOUND	FOUND
1155	4.1.1.17	Ornithine decarboxylase.	NF	NF	NF	NF
1156	4.1.1.19	Arginine decarboxylase.	NF	NF	FOUND	NF
1157	4.1.1.3	Oxaloacetate decarboxylase.	NF	NF	NF	NF
1158	4.1.1.50	Adenosylmethionine decarboxylase.	NF	NF	FOUND	NF
1159	4.1.1.75	5-guanidino-2-oxopentanoate decarboxylase.	NF	NF	NF	NF
1160	4.1.2.14	2-dehydro-3-deoxy-phosphogluconate aldolase.	FOUND	FOUND	NF	FOUND
1161	4.1.3.16	4-hydroxy-2-oxoglutarate aldolase.	FOUND	NF	NF	NF
1162	4.3.1.12	Ornithine cyclodeaminase.	NF	NF	NF	NF
1163	4.3.2.1	Argininosuccinate lyase.	NF	NF	FOUND	FOUND
1164	5.1.1.4	Proline racemase.	NF	NF	NF	NF
1165	5.1.1.8	4-hydroxyproline epimerase.	NF	NF	NF	NF
1166	6.3.1.11	Glutamate--putrescine ligase.	NF	NF	NF	NF
1167	6.3.1.2	Glutamate--ammonia ligase.	NF	FOUND	FOUND	FOUND
1168	6.3.2.11	Carnosine synthase.	NF	NF	NF	NF
1169	6.3.4.16	Carbamoyl-phosphate synthase (ammonia).	NF	NF	NF	NF
1170	6.3.4.5	Argininosuccinate synthase.	NF	NF	FOUND	FOUND
1171	6.3.4.6	Urea carboxylase.	NF	NF	NF	NF
1172	>Clavulanic acid biosynthesis 00331					
1173	1.14.11.21	Clavaminate synthase.	NF	NF	NF	NF
1174	2.5.1.66	N(2)-(2-carboxyethyl)arginine synthase.	NF	NF	NF	NF
1175	3.5.3.22	Proclavaminate amidinohydrolase.	NF	NF	NF	NF
1176	6.3.3.4	(Carboxyethyl)arginine beta-lactam-synthase.	NF	NF	NF	NF
1177	>Histidine metabolism 00340					
1178	1.1.1.23	Histidinol dehydrogenase.	NF	NF	FOUND	FOUND
1179	1.14.13.-	With NADH or NADPH as one donor, and incorporat	NF	NF	NF	NF
1180	1.14.13.5	Imidazoleacetate 4-monooxygenase.	NF	NF	NF	NF
1181	1.2.1.3	Aldehyde dehydrogenase (NAD(+)).	NF	NF	FOUND	FOUND
1182	1.2.1.5	Aldehyde dehydrogenase (NAD(P)(+)).	NF	NF	NF	NF
1183	1.4.3.22	Diamine oxidase.	NF	NF	NF	NF
1184	1.4.3.4	Monoamine oxidase.	NF	NF	NF	NF
1185	2.1.1.-	Methyltransferases.	FOUND	FOUND	FOUND	FOUND
1186	2.1.1.22	Carnosine N-methyltransferase.	NF	NF	NF	NF
1187	2.1.1.8	Histamine N-methyltransferase.	NF	NF	NF	NF
1188	2.1.2.5	Glutamate formimidoyltransferase.	NF	NF	NF	NF
1189	2.3.1.-	Transferring groups other than amino-acyl groups.	FOUND	FOUND	FOUND	FOUND
1190	2.4.2.17	ATP phosphoribosyltransferase.	NF	NF	FOUND	FOUND
1191	2.6.1.38	Histidine transaminase.	NF	NF	NF	NF
1192	2.6.1.9	Histidinol-phosphate transaminase.	NF	NF	FOUND	FOUND
1193	3.1.3.15	Histidinol-phosphatase.	NF	NF	FOUND	FOUND
1194	3.4.13.20	Beta-Ala-His dipeptidase.	NF	NF	NF	NF
1195	3.4.13.3	Xaa-His dipeptidase.	NF	NF	NF	NF
1196	3.4.13.5	Xaa-methyl-His dipeptidase.	NF	NF	NF	NF
1197	3.5.1.15	Aspartoacylase.	NF	NF	NF	NF
1198	3.5.1.68	N-formylglutamate deformylase.	NF	NF	NF	NF
1199	3.5.1.8	Formylaspartate deformylase.	NF	NF	NF	NF
1200	3.5.2.-	In cyclic amides.	NF	NF	FOUND	FOUND
1201	3.5.2.7	Imidazolonepropionase.	NF	NF	NF	NF
1202	3.5.3.13	Formimidoylglutamate deiminase.	NF	NF	NF	NF
1203	3.5.3.5	Formimidoylaspartate deiminase.	NF	NF	NF	NF
1204	3.5.3.8	Formimidoylglutamase.	NF	NF	NF	NF

1205	3.5.4.19	Phosphoribosyl-AMP cyclohydrolase.	NF	NF	FOUND	FOUND
1206	3.6.1.31	Phosphoribosyl-ATP diphosphatase.	NF	NF	NF	NF
1207	4.1.1.22	Histidine decarboxylase.	NF	NF	NF	NF
1208	4.1.1.28	Aromatic-L-amino-acid decarboxylase.	NF	NF	NF	NF
1209	4.2.1.19	Imidazoleglycerol-phosphate dehydratase.	NF	NF	FOUND	FOUND
1210	4.2.1.49	Urocanate hydratase.	NF	NF	NF	NF
1211	4.3.1.3	Histidine ammonia-lyase.	NF	NF	NF	NF
1212	5.3.1.16	1-(5-phosphoribosyl)-5- ((5-phosphoribosylamino)m	NF	NF	FOUND	FOUND
1213	6.3.2.11	Carnosine synthase.	NF	NF	NF	NF
1214	6.3.4.8	Imidazoleacetate--phosphoribosyldiphosphate ligas	NF	NF	NF	NF
1215	>Tyrosine metabolism 00350					
1216	1.1.1.1	Alcohol dehydrogenase.	FOUND	FOUND	FOUND	FOUND
1217	1.1.1.222	(R)-4-hydroxyphenyllactate dehydrogenase.	NF	NF	NF	NF
1218	1.1.1.237	Hydroxyphenylpyruvate reductase.	NF	NF	NF	NF
1219	1.1.1.90	Aryl-alcohol dehydrogenase.	NF	NF	NF	NF
1220	1.10.3.1	Catechol oxidase.	NF	NF	NF	NF
1221	1.11.1.8	Iodide peroxidase.	NF	NF	NF	NF
1222	1.13.11.15	3,4-dihydroxyphenylacetate 2,3-dioxygenase.	NF	NF	NF	NF
1223	1.13.11.27	4-hydroxyphenylpyruvate dioxygenase.	NF	NF	NF	NF
1224	1.13.11.29	Stizolobate synthase.	NF	NF	NF	NF
1225	1.13.11.30	Stizolobinate synthase.	NF	NF	NF	NF
1226	1.13.11.4	Gentisate 1,2-dioxygenase.	NF	NF	NF	NF
1227	1.13.11.5	Homogentisate 1,2-dioxygenase.	NF	NF	NF	NF
1228	1.13.12.-	With incorporation of one atom of oxygen.	NF	NF	FOUND	NF
1229	1.14.12.9	4-chlorophenylacetate 3,4-dioxygenase.	NF	NF	NF	NF
1230	1.14.13.18	4-hydroxyphenylacetate 1-monooxygenase.	NF	NF	NF	NF
1231	1.14.13.3	4-hydroxyphenylacetate 3-monooxygenase.	NF	NF	NF	NF
1232	1.14.16.2	Tyrosine 3-monooxygenase.	NF	NF	NF	NF
1233	1.14.17.1	Dopamine beta-monooxygenase.	NF	NF	NF	NF
1234	1.14.18.-	With another compound as one donor, and incorpo	NF	NF	NF	NF
1235	1.14.18.1	Monophenol monooxygenase.	NF	NF	NF	NF
1236	1.2.1.16	Succinate-semialdehyde dehydrogenase (NAD(P)(+)	NF	NF	NF	NF
1237	1.2.1.29	Aryl-aldehyde dehydrogenase.	NF	NF	NF	NF
1238	1.2.1.45	4-carboxy-2-hydroxymuconate-6-semialdehyde deh	NF	NF	NF	NF
1239	1.2.1.5	Aldehyde dehydrogenase (NAD(P)(+)).	NF	NF	NF	NF
1240	1.2.1.53	4-hydroxyphenylacetaldehyde dehydrogenase.	NF	NF	NF	NF
1241	1.2.1.60	5-carboxymethyl-2-hydroxymuconic-semialdehyde	NF	NF	NF	NF
1242	1.2.3.1	Aldehyde oxidase.	NF	NF	NF	NF
1243	1.2.3.13	4-hydroxyphenylpyruvate oxidase.	NF	NF	NF	NF
1244	1.4.3.2	L-amino-acid oxidase.	NF	NF	NF	NF
1245	1.4.3.21	Primary-amine oxidase.	NF	NF	FOUND	FOUND
1246	1.4.3.4	Monoamine oxidase.	NF	NF	NF	NF
1247	1.4.99.4	Aralkylamine dehydrogenase.	NF	NF	NF	NF
1248	2.1.1.-	Methyltransferases.	FOUND	FOUND	FOUND	FOUND
1249	2.1.1.27	Tyramine N-methyltransferase.	NF	NF	NF	NF
1250	2.1.1.28	Phenylethanolamine N-methyltransferase.	NF	NF	NF	NF
1251	2.1.1.6	Catechol O-methyltransferase.	NF	NF	NF	NF
1252	2.3.1.-	Transferring groups other than amino-acyl groups.	FOUND	FOUND	FOUND	FOUND
1253	2.3.1.14	Glutamine N-phenylacetyltransferase.	NF	NF	NF	NF
1254	2.3.1.140	Rosmarinate synthase.	NF	NF	NF	NF
1255	2.6.1.1	Aspartate transaminase.	FOUND	FOUND	FOUND	FOUND
1256	2.6.1.49	Dihydroxyphenylalanine transaminase.	NF	NF	NF	NF
1257	2.6.1.5	Tyrosine transaminase.	NF	NF	NF	NF
1258	2.6.1.57	Aromatic-amino-acid transaminase.	NF	NF	NF	NF
1259	2.6.1.58	Phenylalanine(histidine) transaminase.	NF	NF	NF	NF

1260	2.6.1.9	Histidinol-phosphate transaminase.	NF	NF	FOUND	FOUND
1261	3.7.1.2	Fumarylacetoacetase.	NF	NF	NF	FOUND
1262	3.7.1.5	Acylpyruvate hydrolase.	NF	NF	NF	NF
1263	4.1.1.-	Carboxy-lyases.	FOUND	FOUND	FOUND	FOUND
1264	4.1.1.25	Tyrosine decarboxylase.	NF	NF	NF	NF
1265	4.1.1.28	Aromatic-L-amino-acid decarboxylase.	NF	NF	NF	NF
1266	4.1.1.62	Gentisate decarboxylase.	NF	NF	NF	NF
1267	4.1.1.68	5-oxopent-3-ene-1,2,5-tricarboxylate decarboxylase	NF	NF	NF	NF
1268	4.1.1.80	4-hydroxyphenylpyruvate decarboxylase.	NF	NF	NF	NF
1269	4.1.2.-	Aldehyde-lyases.	FOUND	FOUND	FOUND	FOUND
1270	4.1.99.2	Tyrosine phenol-lyase.	NF	NF	NF	NF
1271	4.2.1.-	Hydro-lyases.	FOUND	FOUND	FOUND	FOUND
1272	4.3.1.22	3,4-dihydroxyphenylalanine reductive deaminase.	NF	NF	NF	NF
1273	5.2.1.2	Maleylacetoacetate isomerase.	NF	NF	NF	NF
1274	5.2.1.4	Maleylpyruvate isomerase.	NF	NF	NF	NF
1275	5.3.2.1	Phenylpyruvate tautomerase.	NF	NF	NF	NF
1276	5.3.3.-	Transposing C==C bonds.	FOUND	FOUND	FOUND	FOUND
1277	5.3.3.10	5-carboxymethyl-2-hydroxymuconate Delta-isomera	NF	NF	NF	FOUND
1278	5.3.3.12	L-dopachrome isomerase.	NF	NF	NF	NF
1279	5.4.3.6	Tyrosine 2,3-aminomutase.	NF	NF	NF	NF
1280	6.2.1.-	Acid--thiol ligases.	NF	NF	NF	NF
1281	>DDT degradation 00351					
1282	1.13.11.-	With incorporation of two atoms of oxygen.	NF	NF	NF	NF
1283	1.14.99.-	Miscellaneous (requires further characterization).	NF	NF	NF	NF
1284	1.2.-.-	Acting on the aldehyde or oxo group of donors.	FOUND	FOUND	FOUND	FOUND
1285	1.3.-.-	Acting on the CH-CH group of donors.	FOUND	FOUND	FOUND	FOUND
1286	1.3.1.-	With NAD(+) or NADP(+) as acceptor.	FOUND	FOUND	FOUND	FOUND
1287	1.97.1.-	Sole sub-subclass for oxidoreductases that do not b	NF	NF	FOUND	NF
1288	3.7.1.-	In ketonic substances.	NF	NF	NF	FOUND
1289	4.1.1.-	Carboxy-lyases.	FOUND	FOUND	FOUND	FOUND
1290	4.5.1.-	Carbon-halide lyases.	NF	NF	NF	NF
1291	4.5.1.1	DDT-dehydrochlorinase.	NF	NF	NF	NF
1292	>Phenylalanine metabolism 00360					
1293	1.1.1.222	(R)-4-hydroxyphenyllactate dehydrogenase.	NF	NF	NF	NF
1294	1.1.1.237	Hydroxyphenylpyruvate reductase.	NF	NF	NF	NF
1295	1.1.1.90	Aryl-alcohol dehydrogenase.	NF	NF	NF	NF
1296	1.11.1.7	Peroxidase.	FOUND	FOUND	FOUND	FOUND
1297	1.13.11.16	3-carboxyethylcatechol 2,3-dioxygenase.	NF	NF	NF	NF
1298	1.13.11.27	4-hydroxyphenylpyruvate dioxygenase.	NF	NF	NF	NF
1299	1.13.12.9	Phenylalanine 2-monooxygenase.	NF	NF	NF	NF
1300	1.14.12.19	3-phenylpropanoate dioxygenase.	NF	NF	NF	NF
1301	1.14.13.-	With NADH or NADPH as one donor, and incorporat	NF	NF	NF	NF
1302	1.14.13.11	Trans-cinnamate 4-monooxygenase.	NF	NF	NF	NF
1303	1.14.13.14	Trans-cinnamate 2-monooxygenase.	NF	NF	NF	NF
1304	1.14.13.4	Melilotate 3-monooxygenase.	NF	NF	NF	NF
1305	1.14.16.1	Phenylalanine 4-monooxygenase.	NF	NF	NF	NF
1306	1.17.5.1	Phenylacetyl-CoA dehydrogenase.	NF	NF	NF	NF
1307	1.2.1.39	Phenylacetaldehyde dehydrogenase.	NF	NF	NF	NF
1308	1.2.1.5	Aldehyde dehydrogenase (NAD(P)(+)).	NF	NF	NF	NF
1309	1.2.1.58	Phenylglyoxylate dehydrogenase (acylating).	NF	NF	NF	NF
1310	1.3.1.-	With NAD(+) or NADP(+) as acceptor.	FOUND	FOUND	FOUND	FOUND
1311	1.3.1.11	2-coumarate reductase.	NF	NF	NF	NF
1312	1.3.1.31	2-enoate reductase.	NF	NF	NF	NF
1313	1.4.1.20	Phenylalanine dehydrogenase.	NF	NF	NF	NF
1314	1.4.3.2	L-amino-acid oxidase.	NF	NF	NF	NF

1315	1.4.3.21	Primary-amine oxidase.	NF	NF	FOUND	FOUND
1316	1.4.3.4	Monoamine oxidase.	NF	NF	NF	NF
1317	1.4.99.1	D-amino-acid dehydrogenase.	NF	NF	FOUND	NF
1318	1.4.99.4	Aralkylamine dehydrogenase.	NF	NF	NF	NF
1319	1.5.1.18	Ephedrine dehydrogenase.	NF	NF	NF	NF
1320	2.1.1.-	Methyltransferases.	FOUND	FOUND	FOUND	FOUND
1321	2.1.1.104	Caffeoyl-CoA O-methyltransferase.	NF	NF	NF	NF
1322	2.3.1.13	Glycine N-acyltransferase.	NF	NF	NF	NF
1323	2.3.1.14	Glutamine N-phenylacetyltransferase.	NF	NF	NF	NF
1324	2.3.1.36	D-amino-acid N-acetyltransferase.	NF	NF	NF	NF
1325	2.3.1.53	Phenylalanine N-acetyltransferase.	NF	NF	NF	NF
1326	2.3.1.71	Glycine N-benzoyltransferase.	NF	NF	NF	NF
1327	2.6.1.-	Transaminases (aminotransferases).	FOUND	FOUND	FOUND	FOUND
1328	2.6.1.1	Aspartate transaminase.	FOUND	FOUND	FOUND	FOUND
1329	2.6.1.21	D-amino-acid transaminase.	NF	NF	NF	NF
1330	2.6.1.5	Tyrosine transaminase.	NF	NF	NF	NF
1331	2.6.1.57	Aromatic-amino-acid transaminase.	NF	NF	NF	NF
1332	2.6.1.58	Phenylalanine(histidine) transaminase.	NF	NF	NF	NF
1333	2.6.1.9	Histidinol-phosphate transaminase.	NF	NF	FOUND	FOUND
1334	3.1.2.25	Phenylacetyl-CoA hydrolase.	NF	NF	NF	NF
1335	3.5.1.32	Hippurate hydrolase.	NF	NF	NF	NF
1336	3.5.1.4	Amidase.	NF	FOUND	NF	NF
1337	3.7.1.-	In ketonic substances.	NF	NF	NF	FOUND
1338	4.1.1.-	Carboxy-lyases.	FOUND	FOUND	FOUND	FOUND
1339	4.1.1.28	Aromatic-L-amino-acid decarboxylase.	NF	NF	NF	NF
1340	4.1.1.43	Phenylpyruvate decarboxylase.	NF	NF	NF	NF
1341	4.1.1.53	Phenylalanine decarboxylase.	NF	NF	NF	NF
1342	4.1.2.41	Vanillin synthase.	NF	NF	NF	NF
1343	4.1.3.39	4-hydroxy-2-oxovalerate aldolase.	NF	NF	NF	NF
1344	4.2.1.101	Trans-feruloyl-CoA hydratase.	NF	NF	NF	NF
1345	4.2.1.80	2-oxopent-4-enoate hydratase.	NF	NF	NF	NF
1346	4.3.1.24	Phenylalanine ammonia-lyase.	NF	NF	NF	NF
1347	4.3.1.25	Phenylalanine/tyrosine ammonia-lyase.	NF	NF	NF	NF
1348	5.1.1.11	Phenylalanine racemase (ATP-hydrolyzing).	NF	NF	NF	NF
1349	5.3.2.1	Phenylpyruvate tautomerase.	NF	NF	NF	NF
1350	6.2.1.12	4-coumarate--CoA ligase.	NF	NF	NF	NF
1351	6.2.1.30	Phenylacetate--CoA ligase.	NF	NF	NF	NF
1352	>Chlorocyclohexane and chlorobenzene degradation 00361					
1353	1.13.11.1	Catechol 1,2-dioxygenase.	NF	NF	NF	NF
1354	1.13.11.2	Catechol 2,3-dioxygenase.	NF	NF	NF	NF
1355	1.13.11.37	Hydroxyquinol 1,2-dioxygenase.	NF	NF	NF	NF
1356	1.13.11.39	Biphenyl-2,3-diol 1,2-dioxygenase.	NF	NF	NF	NF
1357	1.14.-.-	Acting on paired donors, with incorporation or redu	NF	NF	NF	NF
1358	1.14.12.-	With NADH or NADPH as one donor, and incorporat	NF	NF	NF	NF
1359	1.14.12.3	Benzene 1,2-dioxygenase.	NF	NF	NF	NF
1360	1.14.13.-	With NADH or NADPH as one donor, and incorporat	NF	NF	NF	NF
1361	1.14.13.20	2,4-dichlorophenol 6-monooxygenase.	NF	NF	NF	NF
1362	1.14.13.50	Pentachlorophenol monooxygenase.	NF	NF	NF	NF
1363	1.14.13.7	Phenol 2-monooxygenase.	NF	NF	NF	NF
1364	1.2.1.61	4-hydroxymuconic-semialdehyde dehydrogenase.	NF	NF	NF	NF
1365	1.3.1.-	With NAD(+) or NADP(+) as acceptor.	FOUND	FOUND	FOUND	FOUND
1366	1.3.1.19	Cis-1,2-dihydrobenzene-1,2-diol dehydrogenase.	NF	NF	NF	NF
1367	1.3.1.32	Maleylacetate reductase.	NF	NF	NF	NF
1368	1.6.5.7	2-hydroxy-1,4-benzoquinone reductase.	NF	NF	NF	NF
1369	1.97.1.-	Sole sub-subclass for oxidoreductases that do not b	NF	NF	FOUND	NF

1370	3.1.1.45	Carboxymethylenebutenolidase.	NF	NF	NF	NF
1371	3.8.1.2	(S)-2-haloacid dehalogenase.	NF	NF	NF	NF
1372	3.8.1.3	Haloacetate dehalogenase.	NF	NF	NF	NF
1373	3.8.1.5	Haloalkane dehalogenase.	NF	NF	NF	NF
1374	4.5.1.-	Carbon-halide lyases.	NF	NF	NF	NF
1375	5.2.1.10	2-chloro-4-carboxymethylenebut-2-en-1,4-olide iso	NF	NF	NF	NF
1376	5.5.1.1	Muconate cycloisomerase.	NF	NF	NF	NF
1377	5.5.1.11	Dichloromuconate cycloisomerase.	NF	NF	NF	NF
1378	5.5.1.7	Chloromuconate cycloisomerase.	NF	NF	NF	NF
1379	>Benzoate degradation 00362					
1380	1.1.1.-	With NAD(+) or NADP(+) as acceptor.	FOUND	FOUND	FOUND	FOUND
1381	1.1.1.157	3-hydroxybutyryl-CoA dehydrogenase.	NF	NF	NF	NF
1382	1.1.1.259	3-hydroxypimeloyl-CoA dehydrogenase.	NF	NF	NF	NF
1383	1.13.11.-	With incorporation of two atoms of oxygen.	NF	NF	NF	NF
1384	1.13.11.1	Catechol 1,2-dioxygenase.	NF	NF	NF	NF
1385	1.13.11.14	2,3-dihydroxybenzoate 3,4-dioxygenase.	NF	NF	NF	NF
1386	1.13.11.2	Catechol 2,3-dioxygenase.	NF	NF	NF	NF
1387	1.13.11.3	Protocatechuate 3,4-dioxygenase.	NF	NF	NF	NF
1388	1.13.11.37	Hydroxyquinol 1,2-dioxygenase.	NF	NF	NF	NF
1389	1.13.11.8	Protocatechuate 4,5-dioxygenase.	NF	NF	NF	NF
1390	1.14.12.10	Benzoate 1,2-dioxygenase.	NF	NF	NF	NF
1391	1.14.12.14	2-aminobenzenesulfonate 2,3-dioxygenase.	NF	NF	NF	NF
1392	1.14.13.12	Benzoate 4-monooxygenase.	NF	NF	NF	NF
1393	1.14.13.2	4-hydroxybenzoate 3-monooxygenase.	NF	NF	NF	NF
1394	1.14.13.23	3-hydroxybenzoate 4-monooxygenase.	NF	NF	NF	NF
1395	1.14.13.24	3-hydroxybenzoate 6-monooxygenase.	NF	NF	NF	NF
1396	1.14.13.33	4-hydroxybenzoate 3-monooxygenase (NAD(P)H).	NF	NF	NF	NF
1397	1.14.13.64	4-hydroxybenzoate 1-hydroxylase.	NF	NF	NF	NF
1398	1.14.99.15	4-methoxybenzoate monooxygenase (O-demethyla	NF	NF	NF	NF
1399	1.14.99.23	3-hydroxybenzoate 2-monooxygenase.	NF	NF	NF	NF
1400	1.2.1.10	Acetaldehyde dehydrogenase (acetylating).	NF	NF	NF	NF
1401	1.2.1.32	Aminomuconate-semialdehyde dehydrogenase.	NF	NF	NF	NF
1402	1.2.1.45	4-carboxy-2-hydroxymuconate-6-semialdehyde deh	NF	NF	NF	NF
1403	1.3.1.25	1,6-dihydroxycyclohexa-2,4-diene-1-carboxylate de	NF	NF	NF	NF
1404	1.3.1.32	Maleylacetate reductase.	NF	NF	NF	NF
1405	1.3.1.62	Pimeloyl-CoA dehydrogenase.	NF	NF	NF	NF
1406	1.3.99.-	With other acceptors.	NF	NF	FOUND	FOUND
1407	1.3.99.15	Benzoyl-CoA reductase.	NF	NF	NF	NF
1408	1.3.99.20	4-hydroxybenzoyl-CoA reductase.	NF	NF	NF	NF
1409	1.3.99.7	Glutaryl-CoA dehydrogenase.	NF	NF	NF	NF
1410	2.3.1.-	Transferring groups other than amino-acyl groups.	FOUND	FOUND	FOUND	FOUND
1411	2.3.1.16	Acetyl-CoA C-acyltransferase.	NF	NF	NF	NF
1412	2.3.1.174	3-oxoadipyl-CoA thiolase.	NF	NF	NF	NF
1413	2.3.1.9	Acetyl-CoA C-acetyltransferase.	FOUND	FOUND	NF	NF
1414	2.8.3.6	3-oxoadipate CoA-transferase.	NF	NF	NF	NF
1415	3.1.1.24	3-oxoadipate enol-lactonase.	NF	NF	NF	NF
1416	3.1.1.57	2-pyrone-4,6-dicarboxylate lactonase.	NF	NF	NF	NF
1417	3.1.2.-	Thiolester hydrolases.	NF	FOUND	NF	NF
1418	3.1.2.23	4-hydroxybenzoyl-CoA thioesterase.	NF	NF	NF	NF
1419	3.7.1.-	In ketonic substances.	NF	NF	NF	FOUND
1420	3.7.1.9	2-hydroxymuconate-semialdehyde hydrolase.	NF	NF	NF	NF
1421	4.1.1.-	Carboxy-lyases.	FOUND	FOUND	FOUND	FOUND
1422	4.1.1.44	4-carboxymuconolactone decarboxylase.	NF	NF	NF	NF
1423	4.1.1.46	o-pyrocatechuate decarboxylase.	NF	NF	NF	NF
1424	4.1.1.63	Protocatechuate decarboxylase.	NF	NF	NF	NF

1425	4.1.1.70	Glutaconyl-CoA decarboxylase.	NF	NF	NF	NF
1426	4.1.1.77	4-oxalocrotonate decarboxylase.	NF	NF	NF	NF
1427	4.1.3.17	4-hydroxy-4-methyl-2-oxoglutarate aldolase.	NF	NF	NF	NF
1428	4.1.3.39	4-hydroxy-2-oxovalerate aldolase.	NF	NF	NF	NF
1429	4.2.1.-	Hydro-lyases.	FOUND	FOUND	FOUND	FOUND
1430	4.2.1.100	Cyclohexa-1,5-dienecarbonyl-CoA hydratase.	NF	NF	NF	NF
1431	4.2.1.17	Enoyl-CoA hydratase.	NF	NF	NF	NF
1432	4.2.1.80	2-oxopent-4-enoate hydratase.	NF	NF	NF	NF
1433	4.2.1.83	4-oxalmesaconate hydratase.	NF	NF	NF	NF
1434	5.3.2.-	Interconverting keto- and enol- groups.	NF	NF	NF	NF
1435	5.3.3.10	5-carboxymethyl-2-hydroxymuconate Delta-isomer	NF	NF	NF	FOUND
1436	5.3.3.4	Muconolactone Delta-isomerase.	NF	NF	NF	NF
1437	5.5.1.1	Muconate cycloisomerase.	NF	NF	NF	NF
1438	5.5.1.2	3-carboxy-cis,cis-muconate cycloisomerase.	NF	NF	NF	NF
1439	5.5.1.5	Carboxy-cis,cis-muconate cyclase.	NF	NF	NF	NF
1440	6.2.1.-	Acid--thiol ligases.	NF	NF	NF	NF
1441	6.2.1.25	Benzoate--CoA ligase.	NF	NF	NF	NF
1442	6.2.1.27	4-hydroxybenzoate--CoA ligase.	NF	NF	NF	NF
1443	>Bisphenol degradation 00363					
1444	1.1.-.-	Acting on the CH-OH group of donors.	FOUND	FOUND	FOUND	FOUND
1445	1.1.1.-	With NAD(+) or NADP(+) as acceptor.	FOUND	FOUND	FOUND	FOUND
1446	1.13.-.-	Acting on single donors with incorporation of molec	NF	NF	FOUND	FOUND
1447	1.13.11.41	2,4'-dihydroxyacetophenone dioxygenase.	NF	NF	NF	NF
1448	1.14.-.-	Acting on paired donors, with incorporation or redu	NF	NF	NF	NF
1449	1.14.13.-	With NADH or NADPH as one donor, and incorporat	NF	NF	NF	NF
1450	1.14.13.84	4-hydroxyacetophenone monooxygenase.	NF	NF	NF	NF
1451	1.3.-.-	Acting on the CH-CH group of donors.	FOUND	FOUND	FOUND	FOUND
1452	1.97.1.-	Sole sub-subclass for oxidoreductases that do not b	NF	NF	FOUND	NF
1453	3.1.1.-	Carboxylic ester hydrolases.	FOUND	FOUND	FOUND	FOUND
1454	3.1.1.2	Arylesterase.	NF	NF	NF	NF
1455	4.2.1.-	Hydro-lyases.	FOUND	FOUND	FOUND	FOUND
1456	>Fluorobenzoate degradation 00364					
1457	1.13.11.1	Catechol 1,2-dioxygenase.	NF	NF	NF	NF
1458	1.14.12.-	With NADH or NADPH as one donor, and incorporat	NF	NF	NF	NF
1459	1.14.12.10	Benzoate 1,2-dioxygenase.	NF	NF	NF	NF
1460	1.14.12.13	2-chlorobenzoate 1,2-dioxygenase.	NF	NF	NF	NF
1461	1.14.13.50	Pentachlorophenol monooxygenase.	NF	NF	NF	NF
1462	1.3.1.-	With NAD(+) or NADP(+) as acceptor.	FOUND	FOUND	FOUND	FOUND
1463	1.3.1.25	1,6-dihydroxycyclohexa-2,4-diene-1-carboxylate de	NF	NF	NF	NF
1464	1.3.1.32	Maleylacetate reductase.	NF	NF	NF	NF
1465	1.3.1.63	2,4-dichlorobenzoyl-CoA reductase.	NF	NF	NF	NF
1466	3.1.1.45	Carboxymethylenebutenolidase.	NF	NF	NF	NF
1467	3.5.1.-	In linear amides.	FOUND	FOUND	FOUND	FOUND
1468	3.5.5.6	Bromoxynil nitrilase.	NF	NF	NF	NF
1469	3.8.1.7	4-chlorobenzoyl-CoA dehalogenase.	NF	NF	NF	NF
1470	4.2.1.84	Nitrile hydratase.	NF	NF	NF	NF
1471	5.5.1.1	Muconate cycloisomerase.	NF	NF	NF	NF
1472	5.5.1.7	Chloromuconate cycloisomerase.	NF	NF	NF	NF
1473	6.2.1.-	Acid--thiol ligases.	NF	NF	NF	NF
1474	6.2.1.33	4-chlorobenzoate--CoA ligase.	NF	NF	NF	NF
1475	>Tryptophan metabolism 00380					
1476	1.1.1.110	Indolelactate dehydrogenase.	NF	NF	NF	NF
1477	1.1.1.190	Indole-3-acetaldehyde reductase (NADH).	NF	NF	NF	NF
1478	1.1.1.191	Indole-3-acetaldehyde reductase (NADPH).	NF	NF	NF	NF
1479	1.1.1.35	3-hydroxyacyl-CoA dehydrogenase.	NF	NF	NF	NF

1480	1.10.3.4	o-aminophenol oxidase.	NF	NF	NF	NF
1481	1.11.1.6	Catalase.	NF	NF	NF	NF
1482	1.13.11.-	With incorporation of two atoms of oxygen.	NF	NF	NF	NF
1483	1.13.11.10	7,8-dihydroxykynurenate 8,8a-dioxygenase.	NF	NF	NF	NF
1484	1.13.11.11	Tryptophan 2,3-dioxygenase.	NF	NF	NF	NF
1485	1.13.11.17	Indole 2,3-dioxygenase.	NF	NF	NF	NF
1486	1.13.11.23	2,3-dihydroxyindole 2,3-dioxygenase.	NF	NF	NF	NF
1487	1.13.11.52	Indoleamine 2,3-dioxygenase.	NF	NF	NF	NF
1488	1.13.11.6	3-hydroxyanthranilate 3,4-dioxygenase.	NF	NF	NF	NF
1489	1.13.12.3	Tryptophan 2-monooxygenase.	NF	NF	NF	NF
1490	1.13.99.3	Tryptophan 2'-dioxygenase.	NF	NF	NF	NF
1491	1.14.-.-	Acting on paired donors, with incorporation or redu	NF	NF	NF	NF
1492	1.14.13.-	With NADH or NADPH as one donor, and incorporat	NF	NF	NF	NF
1493	1.14.13.9	Kynurenine 3-monooxygenase.	NF	NF	NF	NF
1494	1.14.14.1	Unspecific monooxygenase.	NF	NF	NF	NF
1495	1.14.16.-	With reduced pteridine as one donor, and incorpora	NF	NF	NF	NF
1496	1.14.16.3	Anthranilate 3-monooxygenase.	NF	NF	NF	NF
1497	1.14.16.4	Tryptophan 5-monooxygenase.	NF	NF	NF	NF
1498	1.14.99.2	Kynurenine 7,8-hydroxylase.	NF	NF	NF	NF
1499	1.2.1.-	With NAD(+) or NADP(+) as acceptor.	FOUND	FOUND	FOUND	FOUND
1500	1.2.1.3	Aldehyde dehydrogenase (NAD(+)).	NF	NF	FOUND	FOUND
1501	1.2.1.32	Aminomuconate-semialdehyde dehydrogenase.	NF	NF	NF	NF
1502	1.2.3.1	Aldehyde oxidase.	NF	NF	NF	NF
1503	1.2.3.7	Indole-3-acetaldehyde oxidase.	NF	NF	NF	NF
1504	1.2.4.2	Oxoglutarate dehydrogenase (succinyl-transferring)	NF	NF	NF	NF
1505	1.3.1.-	With NAD(+) or NADP(+) as acceptor.	FOUND	FOUND	FOUND	FOUND
1506	1.3.1.18	Kynurenate-7,8-dihydrodiol dehydrogenase.	NF	NF	NF	NF
1507	1.3.99.7	Glutaryl-CoA dehydrogenase.	NF	NF	NF	NF
1508	1.4.3.2	L-amino-acid oxidase.	NF	NF	NF	NF
1509	1.4.3.22	Diamine oxidase.	NF	NF	NF	NF
1510	1.4.3.4	Monoamine oxidase.	NF	NF	NF	NF
1511	1.5.1.-	With NAD(+) or NADP(+) as acceptor.	FOUND	FOUND	FOUND	FOUND
1512	1.7.3.-	With oxygen as acceptor.	NF	NF	NF	NF
1513	1.7.3.2	Acetylindoxyl oxidase.	NF	NF	NF	NF
1514	2.1.1.-	Methyltransferases.	FOUND	FOUND	FOUND	FOUND
1515	2.1.1.4	Acetylserotonin O-methyltransferase.	NF	NF	NF	NF
1516	2.1.1.47	Indolepyruvate C-methyltransferase.	NF	NF	NF	NF
1517	2.1.1.49	Amine N-methyltransferase.	NF	NF	NF	NF
1518	2.3.1.87	Aralkylamine N-acetyltransferase.	NF	NF	NF	NF
1519	2.3.1.9	Acetyl-CoA C-acetyltransferase.	FOUND	FOUND	NF	NF
1520	2.4.1.195	N-hydroxythioamide S-beta-glucosyltransferase.	NF	NF	NF	NF
1521	2.5.1.-	Transferring alkyl or aryl groups, other than methyl	FOUND	FOUND	FOUND	FOUND
1522	2.6.1.-	Transaminases (aminotransferases).	FOUND	FOUND	FOUND	FOUND
1523	2.6.1.27	Tryptophan transaminase.	NF	NF	NF	NF
1524	2.6.1.7	Kynurenine--oxoglutarate transaminase.	NF	NF	NF	NF
1525	2.8.2.-	Sulfotransferases.	NF	NF	NF	NF
1526	3.2.1.147	Thioglucosidase.	NF	NF	NF	NF
1527	3.5.1.4	Amidase.	NF	FOUND	NF	NF
1528	3.5.1.9	Arylformamidase.	NF	NF	NF	NF
1529	3.5.5.1	Nitrilase.	NF	NF	NF	NF
1530	3.5.99.5	2-aminomuconate deaminase.	NF	NF	NF	NF
1531	3.7.1.3	Kynureninase.	NF	NF	NF	NF
1532	4.1.1.-	Carboxy-lyases.	FOUND	FOUND	FOUND	FOUND
1533	4.1.1.28	Aromatic-L-amino-acid decarboxylase.	NF	NF	NF	NF
1534	4.1.1.43	Phenylpyruvate decarboxylase.	NF	NF	NF	NF

1535	4.1.1.45	Aminocarboxymuconate-semialdehyde decarboxyla	NF	NF	NF	NF
1536	4.1.1.74	Indolepyruvate decarboxylase.	NF	NF	NF	NF
1537	4.1.99.1	Tryptophanase.	NF	NF	NF	NF
1538	4.2.1.17	Enoyl-CoA hydratase.	NF	NF	NF	NF
1539	4.2.1.84	Nitrile hydratase.	NF	NF	NF	NF
1540	4.4.1.-	Carbon-sulfur lyases.	FOUND	NF	FOUND	FOUND
1541	4.99.1.6	Indoleacetaldoxime dehydratase.	NF	NF	NF	NF
1542	6.1.1.2	Tryptophan--tRNA ligase.	FOUND	FOUND	FOUND	FOUND
1543	6.3.2.-	Acid--D-amino-acid ligases (peptide synthases).	FOUND	FOUND	FOUND	FOUND
1544	>Phenylalanine, tyrosine and tryptophan biosynthesis 00400					
1545	1.1.1.24	Quinate dehydrogenase.	NF	NF	NF	NF
1546	1.1.1.25	Shikimate dehydrogenase.	FOUND	FOUND	FOUND	FOUND
1547	1.1.1.282	Quinate/shikimate dehydrogenase.	NF	NF	NF	NF
1548	1.1.5.8	Quinate dehydrogenase (quinone).	NF	NF	NF	NF
1549	1.14.16.1	Phenylalanine 4-monooxygenase.	NF	NF	NF	NF
1550	1.3.1.12	Prephenate dehydrogenase.	NF	NF	NF	NF
1551	1.3.1.13	Prephenate dehydrogenase (NADP(+)).	NF	NF	FOUND	FOUND
1552	1.3.1.43	Arogenate dehydrogenase.	NF	NF	NF	NF
1553	1.4.1.-	With NAD(+) or NADP(+) as acceptor.	NF	FOUND	FOUND	FOUND
1554	1.4.1.20	Phenylalanine dehydrogenase.	NF	NF	NF	NF
1555	1.4.3.2	L-amino-acid oxidase.	NF	NF	NF	NF
1556	2.4.2.18	Anthranilate phosphoribosyltransferase.	NF	NF	FOUND	FOUND
1557	2.5.1.19	3-phosphoshikimate 1-carboxyvinyltransferase.	NF	NF	FOUND	FOUND
1558	2.5.1.54	3-deoxy-7-phosphoheptulonate synthase.	NF	NF	FOUND	FOUND
1559	2.6.1.1	Aspartate transaminase.	FOUND	FOUND	FOUND	FOUND
1560	2.6.1.5	Tyrosine transaminase.	NF	NF	NF	NF
1561	2.6.1.57	Aromatic-amino-acid transaminase.	NF	NF	NF	NF
1562	2.6.1.58	Phenylalanine(histidine) transaminase.	NF	NF	NF	NF
1563	2.6.1.9	Histidinol-phosphate transaminase.	NF	NF	FOUND	FOUND
1564	2.7.1.71	Shikimate kinase.	NF	FOUND	FOUND	FOUND
1565	4.1.1.48	Indole-3-glycerol-phosphate synthase.	NF	NF	FOUND	FOUND
1566	4.1.2.-	Aldehyde-lyases.	FOUND	FOUND	FOUND	FOUND
1567	4.1.3.27	Anthranilate synthase.	NF	NF	FOUND	FOUND
1568	4.2.1.-	Hydro-lyases.	FOUND	FOUND	FOUND	FOUND
1569	4.2.1.10	3-dehydroquinate dehydratase.	NF	NF	FOUND	FOUND
1570	4.2.1.20	Tryptophan synthase.	NF	NF	FOUND	FOUND
1571	4.2.1.51	Prephenate dehydratase.	NF	NF	FOUND	FOUND
1572	4.2.1.91	Arogenate dehydratase.	NF	NF	NF	NF
1573	4.2.3.4	3-dehydroquinate synthase.	NF	NF	FOUND	FOUND
1574	4.2.3.5	Chorismate synthase.	NF	NF	FOUND	FOUND
1575	5.3.1.24	Phosphoribosylanthranilate isomerase.	NF	NF	FOUND	FOUND
1576	5.4.99.5	Chorismate mutase.	NF	NF	FOUND	FOUND
1577	>Novobiocin biosynthesis 00401					
1578	1.3.1.12	Prephenate dehydrogenase.	NF	NF	NF	NF
1579	1.3.1.43	Arogenate dehydrogenase.	NF	NF	NF	NF
1580	2.6.1.1	Aspartate transaminase.	FOUND	FOUND	FOUND	FOUND
1581	2.6.1.5	Tyrosine transaminase.	NF	NF	NF	NF
1582	2.6.1.57	Aromatic-amino-acid transaminase.	NF	NF	NF	NF
1583	2.6.1.9	Histidinol-phosphate transaminase.	NF	NF	FOUND	FOUND
1584	>Benzoxazinoid biosynthesis 00402					
1585	1.14.-.-	Acting on paired donors, with incorporation or redu	NF	NF	NF	NF
1586	1.14.11.-	With 2-oxoglutarate as one donor, and incorporatio	NF	NF	NF	NF
1587	2.1.1.-	Methyltransferases.	FOUND	FOUND	FOUND	FOUND
1588	2.4.1.202	2,4-dihydroxy-7-methoxy-2H-1,4-benzoxazin-3(4H)-	NF	NF	NF	NF
1589	4.1.2.8	Indole-3-glycerol-phosphate lyase.	NF	NF	NF	NF

1590	>beta-Alanine metabolism 00410					
1591	1.1.1.59	3-hydroxypropionate dehydrogenase.	NF	NF	NF	NF
1592	1.2.1.15	Malonate-semialdehyde dehydrogenase.	NF	NF	NF	NF
1593	1.2.1.18	Malonate-semialdehyde dehydrogenase (acetylatin	NF	NF	NF	NF
1594	1.2.1.19	Aminobutyraldehyde dehydrogenase.	NF	NF	NF	NF
1595	1.2.1.3	Aldehyde dehydrogenase (NAD(+)).	NF	NF	FOUND	FOUND
1596	1.3.1.1	Dihydrouracil dehydrogenase (NAD(+)).	NF	NF	NF	NF
1597	1.3.1.2	Dihydropyrimidine dehydrogenase (NADP(+)).	NF	NF	NF	NF
1598	1.3.99.3	Acyl-CoA dehydrogenase.	NF	NF	FOUND	FOUND
1599	1.4.3.21	Primary-amine oxidase.	NF	NF	FOUND	FOUND
1600	1.5.99.6	Spermidine dehydrogenase.	NF	NF	NF	NF
1601	2.5.1.16	Spermidine synthase.	NF	NF	FOUND	NF
1602	2.5.1.22	Spermine synthase.	NF	NF	NF	NF
1603	2.6.1.18	Beta-alanine--pyruvate transaminase.	NF	NF	NF	NF
1604	2.6.1.19	4-aminobutyrate transaminase.	NF	NF	NF	FOUND
1605	2.6.1.55	Taurine--2-oxoglutarate transaminase.	NF	NF	NF	NF
1606	2.8.3.3	Malonate CoA-transferase.	NF	NF	NF	NF
1607	3.1.2.4	3-hydroxyisobutyryl-CoA hydrolase.	NF	NF	NF	NF
1608	3.4.13.20	Beta-Ala-His dipeptidase.	NF	NF	NF	NF
1609	3.4.13.3	Xaa-His dipeptidase.	NF	NF	NF	NF
1610	3.4.13.4	Xaa-Arg dipeptidase.	NF	NF	NF	NF
1611	3.4.13.5	Xaa-methyl-His dipeptidase.	NF	NF	NF	NF
1612	3.5.1.21	N-acetyl-beta-alanine deacetylase.	NF	NF	NF	NF
1613	3.5.1.6	Beta-ureidopropionase.	NF	NF	NF	NF
1614	3.5.2.2	Dihydropyrimidinase.	NF	NF	NF	FOUND
1615	4.1.1.11	Aspartate 1-decarboxylase.	NF	NF	NF	NF
1616	4.1.1.15	Glutamate decarboxylase.	NF	NF	NF	NF
1617	4.1.1.9	Malonyl-CoA decarboxylase.	NF	NF	NF	NF
1618	4.2.1.17	Enoyl-CoA hydratase.	NF	NF	NF	NF
1619	4.2.1.27	Acetylenecarboxylate hydratase.	NF	NF	NF	NF
1620	4.3.1.6	Beta-alanyl-CoA ammonia-lyase.	NF	NF	NF	NF
1621	6.3.2.1	Pantoate--beta-alanine ligase.	NF	NF	NF	NF
1622	6.3.2.11	Carnosine synthase.	NF	NF	NF	NF
1623	>Taurine and hypotaurine metabolism 00430					
1624	1.13.11.19	Cysteamine dioxygenase.	NF	NF	NF	NF
1625	1.13.11.20	Cysteine dioxygenase.	NF	NF	NF	NF
1626	1.14.11.17	Taurine dioxygenase.	NF	NF	NF	NF
1627	1.2.1.73	Sulfoacetaldehyde dehydrogenase.	NF	NF	NF	NF
1628	1.4.1.1	Alanine dehydrogenase.	NF	NF	NF	NF
1629	1.4.2.-	With a cytochrome as acceptor.	NF	NF	NF	NF
1630	1.8.1.3	Hypotaurine dehydrogenase.	NF	NF	NF	NF
1631	2.3.1.65	Bile acid-CoA:amino acid N-acyltransferase.	NF	NF	NF	NF
1632	2.3.1.8	Phosphate acetyltransferase.	FOUND	FOUND	FOUND	FOUND
1633	2.3.2.2	Gamma-glutamyltransferase.	NF	NF	NF	NF
1634	2.3.3.15	Sulfoacetaldehyde acetyltransferase.	NF	NF	NF	NF
1635	2.6.1.77	Taurine--pyruvate aminotransferase.	NF	NF	NF	NF
1636	2.7.2.1	Acetate kinase.	FOUND	FOUND	FOUND	FOUND
1637	2.7.3.4	Taurocyamine kinase.	NF	NF	NF	NF
1638	4.1.1.15	Glutamate decarboxylase.	NF	NF	NF	NF
1639	4.1.1.29	Sulfinoalanine decarboxylase.	NF	NF	NF	NF
1640	4.4.1.10	Cysteine lyase.	NF	NF	NF	NF
1641	>Phosphonate and phosphinate metabolism 00440					
1642	1.1.99.-	With other acceptors.	NF	NF	NF	NF
1643	2.6.1.-	Transaminases (aminotransferases).	FOUND	FOUND	FOUND	FOUND
1644	2.6.1.37	2-aminoethylphosphonate--pyruvate transaminase.	NF	NF	NF	NF

1645	2.7.7.14	Ethanolamine-phosphate cytidylyltransferase.	NF	NF	NF	NF
1646	2.7.7.15	Choline-phosphate cytidylyltransferase.	NF	NF	NF	NF
1647	2.7.8.-	Transferases for other substituted phosphate group	FOUND	FOUND	FOUND	FOUND
1648	2.7.8.1	Ethanolaminephosphotransferase.	NF	NF	NF	NF
1649	2.7.8.2	Diacylglycerol cholinephosphotransferase.	NF	NF	NF	NF
1650	3.11.1.1	Phosphonoacetaldehyde hydrolase.	NF	NF	NF	FOUND
1651	3.11.1.2	Phosphonoacetate hydrolase.	NF	NF	NF	NF
1652	4.1.1.82	Phosphonopyruvate decarboxylase.	NF	NF	NF	NF
1653	4.99.1.-	Sole sub-subclass for lyases that do not belong in th	NF	NF	NF	NF
1654	5.4.2.9	Phosphoenolpyruvate mutase.	NF	NF	NF	NF
1655	>Selenocompound metabolism 00450					
1656	1.8.1.9	Thioredoxin-disulfide reductase.	NF	NF	FOUND	NF
1657	1.97.1.9	Selenate reductase.	NF	NF	NF	NF
1658	2.1.1.-	Methyltransferases.	FOUND	FOUND	FOUND	FOUND
1659	2.1.1.12	Methionine S-methyltransferase.	NF	NF	NF	NF
1660	2.1.1.13	Methionine synthase.	NF	NF	FOUND	FOUND
1661	2.1.1.14	5-methyltetrahydropteroyltriglutamate--homocyste	FOUND	FOUND	FOUND	NF
1662	2.1.1.96	Thioether S-methyltransferase.	NF	NF	NF	NF
1663	2.5.1.48	Cystathionine gamma-synthase.	NF	NF	FOUND	NF
1664	2.6.1.-	Transaminases (aminotransferases).	FOUND	FOUND	FOUND	FOUND
1665	2.7.1.164	O-phosphoseryl-tRNA(Sec) kinase.	NF	NF	NF	NF
1666	2.7.7.4	Sulfate adenylyltransferase.	NF	NF	FOUND	NF
1667	2.7.9.3	Selenide, water dikinase.	NF	NF	NF	FOUND
1668	2.9.1.1	L-seryl-tRNA(Sec) selenium transferase.	NF	NF	NF	NF
1669	2.9.1.2	O-phospho-L-seryl-tRNA(Sec):L-selenocysteinyl-tRN	NF	NF	NF	NF
1670	4.4.1.1	Cystathionine gamma-lyase.	NF	NF	FOUND	NF
1671	4.4.1.11	Methionine gamma-lyase.	NF	NF	NF	NF
1672	4.4.1.16	Selenocysteine lyase.	NF	NF	NF	NF
1673	4.4.1.8	Cystathionine beta-lyase.	FOUND	NF	FOUND	NF
1674	6.1.1.10	Methionine--tRNA ligase.	FOUND	FOUND	FOUND	FOUND
1675	>Cyanoamino acid metabolism 00460					
1676	1.14.13.-	With NADH or NADPH as one donor, and incorporat	NF	NF	NF	NF
1677	1.14.13.41	Tyrosine N-monooxygenase.	NF	NF	NF	NF
1678	1.14.13.68	4-hydroxyphenylacetaldehyde oxime monooxygena	NF	NF	NF	NF
1679	1.4.99.5	Glycine dehydrogenase (cyanide-forming).	NF	NF	NF	NF
1680	2.1.2.1	Glycine hydroxymethyltransferase.	NF	NF	FOUND	FOUND
1681	2.3.2.2	Gamma-glutamyltransferase.	NF	NF	NF	NF
1682	2.4.1.178	Hydroxymandelonitrile glucosyltransferase.	NF	NF	NF	NF
1683	2.4.1.63	Linamarin synthase.	NF	NF	NF	NF
1684	2.4.1.85	Cyanohydrin beta-glucosyltransferase.	NF	NF	NF	NF
1685	3.2.1.117	Amygdalin beta-glucosidase.	NF	NF	NF	NF
1686	3.2.1.118	Prunasin beta-glucosidase.	NF	NF	NF	NF
1687	3.2.1.21	Beta-glucosidase.	NF	NF	NF	NF
1688	3.5.1.1	Asparaginase.	NF	NF	NF	NF
1689	3.5.1.4	Amidase.	NF	FOUND	NF	NF
1690	3.5.5.1	Nitrilase.	NF	NF	NF	NF
1691	3.5.5.4	Cyanoalanine nitrilase.	NF	NF	NF	NF
1692	3.5.5.5	Arylacetonitrilase.	NF	NF	NF	NF
1693	4.1.2.10	Mandelonitrile lyase.	NF	NF	NF	NF
1694	4.1.2.11	Hydroxymandelonitrile lyase.	NF	NF	NF	NF
1695	4.1.2.46	???	NF	NF	NF	NF
1696	4.1.2.47	???	NF	NF	NF	NF
1697	4.2.1.65	3-cyanoalanine hydratase.	NF	NF	NF	NF
1698	4.2.1.66	Cyanide hydratase.	NF	NF	NF	NF
1699	4.2.1.84	Nitrile hydratase.	NF	NF	NF	NF

1700	4.4.1.9	L-3-cyanoalanine synthase.	NF	NF	NF	NF
1701	4.99.1.5	Aliphatic aldoxime dehydratase.	NF	NF	NF	NF
1702	4.99.1.7	Phenylacetaldoxime dehydratase.	NF	NF	NF	NF
1703	6.3.1.1	Aspartate--ammonia ligase.	FOUND	FOUND	FOUND	FOUND
1704	>D-Glutamine and D-glutamate metabolism 00471					
1705	1.4.1.3	Glutamate dehydrogenase (NAD(P)(+)).	NF	NF	NF	NF
1706	1.4.3.15	D-glutamate(D-aspartate) oxidase.	NF	NF	NF	NF
1707	1.4.3.7	D-glutamate oxidase.	NF	NF	NF	NF
1708	2.3.2.1	D-glutamyltransferase.	NF	NF	NF	NF
1709	3.5.1.2	Glutaminase.	NF	NF	NF	NF
1710	3.5.1.35	D-glutaminase.	NF	NF	NF	NF
1711	3.5.1.38	Glutamin-(asparagin-)ase.	NF	NF	NF	NF
1712	4.2.1.48	D-glutamate cyclase.	NF	NF	NF	NF
1713	5.1.1.10	Amino-acid racemase.	NF	NF	NF	NF
1714	5.1.1.3	Glutamate racemase.	FOUND	FOUND	FOUND	FOUND
1715	6.3.2.8	UDP-N-acetylmuramate--L-alanine ligase.	FOUND	FOUND	FOUND	FOUND
1716	6.3.2.9	UDP-N-acetylmuramoyl-L-alanine--D-glutamate liga	FOUND	FOUND	FOUND	FOUND
1717	>D-Arginine and D-ornithine metabolism 00472					
1718	1.4.1.12	2,4-diaminopentanoate dehydrogenase.	NF	NF	NF	NF
1719	1.4.3.3	D-amino-acid oxidase.	NF	NF	NF	NF
1720	2.6.1.21	D-amino-acid transaminase.	NF	NF	NF	NF
1721	3.5.3.10	D-arginase.	NF	NF	NF	NF
1722	5.1.1.10	Amino-acid racemase.	NF	NF	NF	NF
1723	5.1.1.12	Ornithine racemase.	NF	NF	NF	NF
1724	5.1.1.9	Arginine racemase.	NF	NF	NF	NF
1725	5.4.3.5	D-ornithine 4,5-aminomutase.	NF	NF	NF	NF
1726	>D-Alanine metabolism 00473					
1727	2.6.1.21	D-amino-acid transaminase.	NF	NF	NF	NF
1728	2.6.1.41	D-methionine--pyruvate transaminase.	NF	NF	NF	NF
1729	5.1.1.1	Alanine racemase.	FOUND	FOUND	FOUND	FOUND
1730	6.1.1.13	D-alanine--poly(phosphoribitol) ligase.	NF	NF	NF	NF
1731	6.3.2.16	D-alanine--alanyl-poly(glycerolphosphate) ligase.	NF	NF	NF	NF
1732	6.3.2.4	D-alanine--D-alanine ligase.	FOUND	FOUND	FOUND	FOUND
1733	>Glutathione metabolism 00480					
1734	1.1.1.42	Isocitrate dehydrogenase (NADP(+)).	NF	NF	FOUND	FOUND
1735	1.1.1.43	Phosphogluconate 2-dehydrogenase.	NF	NF	NF	NF
1736	1.1.1.44	Phosphogluconate dehydrogenase (decarboxylating	NF	NF	NF	NF
1737	1.1.1.49	Glucose-6-phosphate dehydrogenase.	NF	NF	NF	NF
1738	1.11.1.-	Peroxidases.	FOUND	FOUND	FOUND	FOUND
1739	1.11.1.11	L-ascorbate peroxidase.	NF	NF	NF	NF
1740	1.11.1.12	Phospholipid-hydroperoxide glutathione peroxidase	NF	NF	NF	NF
1741	1.11.1.15	Peroxiredoxin.	NF	NF	NF	NF
1742	1.11.1.9	Glutathione peroxidase.	NF	NF	NF	NF
1743	1.17.4.1	Ribonucleoside-diphosphate reductase.	FOUND	FOUND	FOUND	FOUND
1744	1.5.4.1	Pyrimidodiazepine synthase.	NF	NF	NF	NF
1745	1.8.1.12	Trypanothione-disulfide reductase.	NF	NF	NF	NF
1746	1.8.1.13	Bis-gamma-glutamylcystine reductase.	NF	NF	NF	NF
1747	1.8.1.7	Glutathione-disulfide reductase.	NF	NF	NF	NF
1748	1.8.3.3	Glutathione oxidase.	NF	NF	NF	NF
1749	1.8.4.1	Glutathione--homocystine transhydrogenase.	NF	NF	NF	NF
1750	1.8.4.2	Protein-disulfide reductase (glutathione).	NF	NF	NF	NF
1751	1.8.4.3	Glutathione--CoA-glutathione transhydrogenase.	NF	NF	NF	NF
1752	1.8.4.4	Glutathione--cystine transhydrogenase.	NF	NF	NF	NF
1753	1.8.4.7	Enzyme-thiol transhydrogenase (glutathione-disulfi	NF	NF	NF	NF
1754	1.8.5.1	Glutathione dehydrogenase (ascorbate).	NF	NF	NF	NF

1755	2.3.1.80	Cysteine-S-conjugate N-acetyltransferase.	NF	NF	NF	NF
1756	2.3.2.2	Gamma-glutamyltransferase.	NF	NF	NF	NF
1757	2.3.2.4	Gamma-glutamylcyclotransferase.	NF	NF	NF	NF
1758	2.5.1.16	Spermidine synthase.	NF	NF	FOUND	NF
1759	2.5.1.18	Glutathione transferase.	NF	NF	NF	NF
1760	2.5.1.22	Spermine synthase.	NF	NF	NF	NF
1761	2.8.1.3	Thiosulfate--thiol sulfurtransferase.	NF	NF	NF	NF
1762	3.4.11.1	Leucyl aminopeptidase.	FOUND	FOUND	NF	NF
1763	3.4.11.2	Membrane alanyl aminopeptidase.	NF	NF	NF	NF
1764	3.4.11.23	PepB aminopeptidase.	NF	NF	NF	NF
1765	3.4.13.3	Xaa-His dipeptidase.	NF	NF	NF	NF
1766	3.5.1.-	In linear amides.	FOUND	FOUND	FOUND	FOUND
1767	3.5.1.78	Glutathionylspermidine amidase.	NF	NF	NF	NF
1768	3.5.2.9	5-oxoprolinase (ATP-hydrolyzing).	NF	NF	NF	NF
1769	4.1.1.17	Ornithine decarboxylase.	NF	NF	NF	NF
1770	6.3.1.8	Glutathionylspermidine synthase.	NF	NF	NF	NF
1771	6.3.1.9	Trypanothione synthase.	NF	NF	NF	NF
1772	6.3.2.2	Glutamate--cysteine ligase.	NF	NF	NF	NF
1773	6.3.2.3	Glutathione synthase.	NF	NF	NF	NF
1774	>Starch and sucrose metabolism 00500					
1775	1.1.1.22	UDP-glucose 6-dehydrogenase.	NF	NF	NF	FOUND
1776	1.1.99.13	Glucoside 3-dehydrogenase.	NF	NF	NF	NF
1777	2.4.1.1	Phosphorylase.	FOUND	FOUND	FOUND	FOUND
1778	2.4.1.10	Levansucrase.	NF	NF	NF	NF
1779	2.4.1.11	Glycogen(starch) synthase.	NF	NF	NF	NF
1780	2.4.1.12	Cellulose synthase (UDP-forming).	NF	NF	NF	NF
1781	2.4.1.13	Sucrose synthase.	NF	NF	NF	NF
1782	2.4.1.14	Sucrose-phosphate synthase.	NF	NF	NF	NF
1783	2.4.1.15	Alpha,alpha-trehalose-phosphate synthase (UDP-fo	NF	NF	NF	NF
1784	2.4.1.17	Glucuronosyltransferase.	NF	NF	NF	NF
1785	2.4.1.18	1,4-alpha-glucan branching enzyme.	NF	NF	NF	NF
1786	2.4.1.20	Cellobiose phosphorylase.	NF	NF	NF	NF
1787	2.4.1.21	Starch synthase.	FOUND	FOUND	FOUND	FOUND
1788	2.4.1.25	4-alpha-glucanotransferase.	FOUND	FOUND	FOUND	FOUND
1789	2.4.1.29	Cellulose synthase (GDP-forming).	NF	NF	NF	NF
1790	2.4.1.34	1,3-beta-glucan synthase.	NF	NF	NF	NF
1791	2.4.1.35	Phenol beta-glucosyltransferase.	NF	NF	NF	NF
1792	2.4.1.4	Amylosucrase.	NF	NF	NF	NF
1793	2.4.1.43	Polygalacturonate 4-alpha-galacturonosyltransferas	NF	NF	NF	NF
1794	2.4.1.5	Dextransucrase.	NF	NF	NF	NF
1795	2.4.1.64	Alpha,alpha-trehalose phosphorylase.	NF	NF	NF	NF
1796	2.4.1.7	Sucrose phosphorylase.	NF	NF	NF	FOUND
1797	2.4.1.8	Maltose phosphorylase.	NF	NF	NF	NF
1798	2.4.2.24	1,4-beta-D-xylan synthase.	NF	NF	NF	NF
1799	2.7.1.1	Hexokinase.	NF	NF	NF	NF
1800	2.7.1.10	Phosphoglucokinase.	NF	NF	NF	NF
1801	2.7.1.106	Glucose-1,6-bisphosphate synthase.	NF	NF	NF	NF
1802	2.7.1.2	Glucokinase.	FOUND	FOUND	FOUND	FOUND
1803	2.7.1.4	Fructokinase.	NF	NF	FOUND	FOUND
1804	2.7.1.41	Glucose-1-phosphate phosphodismutase.	NF	NF	NF	NF
1805	2.7.1.69	Protein-N(pi)-phosphohistidine--sugar phosphotran	FOUND	FOUND	FOUND	FOUND
1806	2.7.7.27	Glucose-1-phosphate adenylyltransferase.	FOUND	FOUND	FOUND	FOUND
1807	2.7.7.33	Glucose-1-phosphate cytidylyltransferase.	NF	NF	NF	NF
1808	2.7.7.34	Glucose-1-phosphate guanylyltransferase.	NF	NF	NF	NF
1809	2.7.7.9	UTP--glucose-1-phosphate uridylyltransferase.	FOUND	FOUND	FOUND	FOUND

1810	3.1.1.11	Pectinesterase.	NF	NF	NF	NF
1811	3.1.2.21	Dodecanoyl-[acyl-carrier-protein] hydrolase.	NF	NF	NF	NF
1812	3.1.3.12	Trehalose-phosphatase.	NF	NF	NF	NF
1813	3.1.3.24	Sucrose-phosphate phosphatase.	NF	NF	NF	NF
1814	3.1.3.9	Glucose-6-phosphatase.	NF	NF	NF	NF
1815	3.2.1.1	Alpha-amylase.	NF	FOUND	NF	NF
1816	3.2.1.10	Oligo-1,6-glucosidase.	NF	NF	FOUND	NF
1817	3.2.1.122	Maltose-6'-phosphate glucosidase.	NF	NF	NF	FOUND
1818	3.2.1.15	Polygalacturonase.	NF	NF	NF	FOUND
1819	3.2.1.2	Beta-amylase.	NF	NF	NF	NF
1820	3.2.1.20	Alpha-glucosidase.	NF	NF	NF	NF
1821	3.2.1.21	Beta-glucosidase.	NF	NF	NF	NF
1822	3.2.1.26	Beta-fructofuranosidase.	NF	NF	NF	NF
1823	3.2.1.28	Alpha,alpha-trehalase.	NF	NF	NF	FOUND
1824	3.2.1.3	Glucan 1,4-alpha-glucosidase.	NF	NF	NF	NF
1825	3.2.1.31	Beta-glucuronidase.	NF	NF	NF	NF
1826	3.2.1.33	Amylo-alpha-1,6-glucosidase.	NF	NF	NF	NF
1827	3.2.1.37	Xylan 1,4-beta-xylosidase.	NF	NF	NF	NF
1828	3.2.1.39	Glucan endo-1,3-beta-D-glucosidase.	NF	NF	NF	NF
1829	3.2.1.4	Cellulase.	NF	NF	NF	NF
1830	3.2.1.48	Sucrose alpha-glucosidase.	NF	NF	NF	NF
1831	3.2.1.54	Cyclomaltodextrinase.	NF	NF	NF	NF
1832	3.2.1.58	Glucan 1,3-beta-glucosidase.	NF	NF	NF	NF
1833	3.2.1.65	Levanase.	NF	NF	NF	NF
1834	3.2.1.67	Galacturan 1,4-alpha-galacturonidase.	NF	NF	NF	NF
1835	3.2.1.74	Glucan 1,4-beta-glucosidase.	NF	NF	NF	NF
1836	3.2.1.91	Cellulose 1,4-beta-cellobiosidase.	NF	NF	NF	NF
1837	3.2.1.93	Alpha,alpha-phosphotrehalase.	NF	NF	FOUND	FOUND
1838	3.6.1.21	ADP-sugar diphosphatase.	NF	NF	NF	NF
1839	3.6.1.9	Nucleotide diphosphatase.	NF	NF	NF	NF
1840	4.1.1.35	UDP-glucuronate decarboxylase.	NF	NF	NF	NF
1841	5.1.3.6	UDP-glucuronate 4-epimerase.	NF	NF	NF	NF
1842	5.3.1.9	Glucose-6-phosphate isomerase.	FOUND	FOUND	FOUND	FOUND
1843	5.4.2.2	Phosphoglucomutase.	FOUND	FOUND	FOUND	FOUND
1844	5.4.2.6	Beta-phosphoglucomutase.	NF	NF	NF	FOUND
1845	5.4.99.16	Maltose alpha-D-glucosyltransferase.	NF	NF	NF	NF
1846	>N-Glycan biosynthesis 00510					
1847	2.4.1.-	Hexosyltransferases.	FOUND	FOUND	FOUND	FOUND
1848	2.4.1.101	Alpha-1,3-mannosyl-glycoprotein 2-beta-N-acetylgl	NF	NF	NF	NF
1849	2.4.1.117	Dolichyl-phosphate beta-glucosyltransferase.	NF	NF	NF	NF
1850	2.4.1.119	Dolichyl-diphosphooligosaccharide--protein glycotr	NF	NF	NF	NF
1851	2.4.1.130	Dolichyl-phosphate-mannose--glycolipid alpha-man	NF	NF	NF	NF
1852	2.4.1.131	Glycolipid 2-alpha-mannosyltransferase.	NF	NF	NF	NF
1853	2.4.1.132	Glycolipid 3-alpha-mannosyltransferase.	NF	NF	NF	NF
1854	2.4.1.141	N-acetylglucosaminyldiphosphodolichol N-acetylglu	NF	NF	NF	NF
1855	2.4.1.142	Chitobiosyldiphosphodolichol beta-mannosyltransf	NF	NF	NF	NF
1856	2.4.1.143	Alpha-1,6-mannosyl-glycoprotein 2-beta-N-acetylgl	NF	NF	NF	NF
1857	2.4.1.144	Beta-1,4-mannosyl-glycoprotein 4-beta-N-acetylglu	NF	NF	NF	NF
1858	2.4.1.145	Alpha-1,3-mannosyl-glycoprotein 4-beta-N-acetylgl	NF	NF	NF	NF
1859	2.4.1.155	Alpha-1,6-mannosyl-glycoprotein 6-beta-N-acetylgl	NF	NF	NF	NF
1860	2.4.1.201	Alpha-1,6-mannosyl-glycoprotein 4-beta-N-acetylgl	NF	NF	NF	NF
1861	2.4.1.38	Beta-N-acetylglucosaminylglycopeptide beta-1,4-ga	NF	NF	NF	NF
1862	2.4.1.68	Glycoprotein 6-alpha-L-fucosyltransferase.	NF	NF	NF	NF
1863	2.4.1.83	Dolichyl-phosphate beta-D-mannosyltransferase.	NF	NF	NF	FOUND
1864	2.4.99.1	Beta-galactoside alpha-2,6-sialyltransferase.	NF	NF	NF	NF

1865	2.7.1.108	Dolichol kinase.	NF	NF	NF	NF
1866	2.7.8.15	UDP-N-acetylglucosamine--dolichyl-phosphate N-ac	NF	NF	NF	NF
1867	3.1.3.51	Dolichyl-phosphatase.	NF	NF	NF	NF
1868	3.1.4.48	Dolichylphosphate-glucose phosphodiesterase.	NF	NF	NF	NF
1869	3.2.1.106	Mannosyl-oligosaccharide glucosidase.	NF	NF	NF	FOUND
1870	3.2.1.113	Mannosyl-oligosaccharide 1,2-alpha-mannosidase.	NF	NF	NF	NF
1871	3.2.1.114	Mannosyl-oligosaccharide 1,3-1,6-alpha-mannosida	NF	NF	NF	NF
1872	3.2.1.84	Glucan 1,3-alpha-glucosidase.	NF	NF	NF	NF
1873	3.6.1.43	Dolichyldiphosphatase.	NF	NF	NF	NF
1874	>Other glycan degradation 00511					
1875	3.2.1.18	Exo-alpha-sialidase.	NF	FOUND	NF	NF
1876	3.2.1.23	Beta-galactosidase.	NF	NF	NF	NF
1877	3.2.1.24	Alpha-mannosidase.	NF	NF	NF	NF
1878	3.2.1.25	Beta-mannosidase.	NF	NF	NF	NF
1879	3.2.1.45	Glucosylceramidase.	NF	NF	NF	NF
1880	3.2.1.51	Alpha-L-fucosidase.	NF	NF	NF	NF
1881	3.2.1.52	Beta-N-acetylhexosaminidase.	NF	NF	NF	NF
1882	3.2.1.96	Mannosyl-glycoprotein endo-beta-N-acetylglucosan	NF	NF	NF	NF
1883	3.5.1.26	N(4)-(beta-N-acetylglucosaminyl)-L-asparaginase.	NF	NF	NF	NF
1884	>Mucin type O-Glycan biosynthesis 00512					
1885	2.4.1.-	Hexosyltransferases.	FOUND	FOUND	FOUND	FOUND
1886	2.4.1.102	Beta-1,3-galactosyl-O-glycosyl-glycoprotein beta-1,6	NF	NF	NF	NF
1887	2.4.1.122	Glycoprotein-N-acetylgalactosamine 3-beta-galacto	NF	NF	NF	NF
1888	2.4.1.147	Acetylgalactosaminyl-O-glycosyl-glycoprotein beta-1	NF	NF	NF	NF
1889	2.4.1.148	Acetylgalactosaminyl-O-glycosyl-glycoprotein beta-1	NF	NF	NF	NF
1890	2.4.1.41	Polypeptide N-acetylgalactosaminyltransferase.	NF	NF	NF	NF
1891	2.4.99.3	Alpha-N-acetylgalactosaminide alpha-2,6-sialyltrans	NF	NF	NF	NF
1892	2.4.99.4	Beta-galactoside alpha-2,3-sialyltransferase.	NF	NF	NF	NF
1893	>Various types of N-glycan biosynthesis 00513					
1894	2.4.1.-	Hexosyltransferases.	FOUND	FOUND	FOUND	FOUND
1895	2.4.1.101	Alpha-1,3-mannosyl-glycoprotein 2-beta-N-acetylglu	NF	NF	NF	NF
1896	2.4.1.119	Dolichyl-diphosphooligosaccharide--protein glycotra	NF	NF	NF	NF
1897	2.4.1.130	Dolichyl-phosphate-mannose--glycolipid alpha-man	NF	NF	NF	NF
1898	2.4.1.131	Glycolipid 2-alpha-mannosyltransferase.	NF	NF	NF	NF
1899	2.4.1.132	Glycolipid 3-alpha-mannosyltransferase.	NF	NF	NF	NF
1900	2.4.1.141	N-acetylglucosaminyldiphosphodolichol N-acetylglu	NF	NF	NF	NF
1901	2.4.1.142	Chitobiosyldiphosphodolichol beta-mannosyltransfe	NF	NF	NF	NF
1902	2.4.1.143	Alpha-1,6-mannosyl-glycoprotein 2-beta-N-acetylglu	NF	NF	NF	NF
1903	2.4.1.145	Alpha-1,3-mannosyl-glycoprotein 4-beta-N-acetylglu	NF	NF	NF	NF
1904	2.4.1.214	Glycoprotein 3-alpha-L-fucosyltransferase.	NF	NF	NF	NF
1905	2.4.1.232	Initiation-specific alpha-1,6-mannosyltransferase.	NF	NF	NF	NF
1906	2.4.1.244	N-acetyl-beta-glucosaminyl-glycoprotein 4-beta-N-	NF	NF	NF	NF
1907	2.4.1.38	Beta-N-acetylglucosaminylglycopeptide beta-1,4-ga	NF	NF	NF	NF
1908	2.4.1.68	Glycoprotein 6-alpha-L-fucosyltransferase.	NF	NF	NF	NF
1909	2.4.2.38	Glycoprotein 2-beta-D-xylosyltransferase.	NF	NF	NF	NF
1910	2.4.99.6	N-acetyllactosaminide alpha-2,3-sialyltransferase.	NF	NF	NF	NF
1911	2.7.8.-	Transferases for other substituted phosphate group	FOUND	FOUND	FOUND	FOUND
1912	2.8.2.-	Sulfotransferases.	NF	NF	NF	NF
1913	3.2.1.113	Mannosyl-oligosaccharide 1,2-alpha-mannosidase.	NF	NF	NF	NF
1914	3.2.1.114	Mannosyl-oligosaccharide 1,3-1,6-alpha-mannosida	NF	NF	NF	NF
1915	3.2.1.52	Beta-N-acetylhexosaminidase.	NF	NF	NF	NF
1916	>Other types of O-glycan biosynthesis 00514					
1917	2.4.1.-	Hexosyltransferases.	FOUND	FOUND	FOUND	FOUND
1918	2.4.1.109	Dolichyl-phosphate-mannose-protein mannosyltran	NF	NF	NF	NF
1919	2.4.1.152	4-galactosyl-N-acetylglucosaminide 3-alpha-L-fucos	NF	NF	NF	NF

1920	2.4.1.17	Glucuronosyltransferase.	NF	NF	NF	NF
1921	2.4.1.221	Peptide-O-fucosyltransferase.	NF	NF	NF	NF
1922	2.4.1.222	O-fucosylpeptide 3-beta-N-acetylglucosaminyltrans	NF	NF	NF	NF
1923	2.4.1.50	Procollagen galactosyltransferase.	NF	NF	NF	NF
1924	2.4.1.66	Procollagen glucosyltransferase.	NF	NF	NF	NF
1925	2.4.2.-	Pentosyltransferases.	FOUND	FOUND	FOUND	FOUND
1926	2.4.99.1	Beta-galactoside alpha-2,6-sialyltransferase.	NF	NF	NF	NF
1927	2.4.99.6	N-acetyllactosaminide alpha-2,3-sialyltransferase.	NF	NF	NF	NF
1928	2.8.2.-	Sulfotransferases.	NF	NF	NF	NF
1929	>Amino sugar and nucleotide sugar metabolism 00520					
1930	1.-.-.-	Oxidoreductases.	FOUND	FOUND	FOUND	FOUND
1931	1.1.1.-	With NAD(+) or NADP(+) as acceptor.	FOUND	FOUND	FOUND	FOUND
1932	1.1.1.132	GDP-mannose 6-dehydrogenase.	NF	NF	NF	NF
1933	1.1.1.135	GDP-6-deoxy-D-talose 4-dehydrogenase.	NF	NF	NF	NF
1934	1.1.1.136	UDP-N-acetylglucosamine 6-dehydrogenase.	NF	NF	NF	NF
1935	1.1.1.158	UDP-N-acetylmuramate dehydrogenase.	FOUND	FOUND	FOUND	FOUND
1936	1.1.1.187	GDP-4-dehydro-D-rhamnose reductase.	NF	NF	NF	NF
1937	1.1.1.22	UDP-glucose 6-dehydrogenase.	NF	NF	NF	FOUND
1938	1.1.1.271	GDP-L-fucose synthase.	NF	NF	NF	NF
1939	1.1.1.281	GDP-4-dehydro-6-deoxy-D-mannose reductase.	NF	NF	NF	NF
1940	1.1.1.305	UDP-glucuronic acid oxidase (UDP-4-keto-hexauron	NF	NF	NF	NF
1941	1.14.18.2	CMP-N-acetylneuraminate monooxygenase.	NF	NF	NF	NF
1942	1.17.1.1	CDP-4-dehydro-6-deoxyglucose reductase.	NF	NF	NF	NF
1943	1.6.2.2	Cytochrome-b5 reductase.	NF	NF	NF	NF
1944	2.1.2.-	Hydroxymethyl-, formyl- and related transferases.	FOUND	FOUND	FOUND	FOUND
1945	2.3.1.157	Glucosamine-1-phosphate N-acetyltransferase.	NF	NF	NF	NF
1946	2.3.1.3	Glucosamine N-acetyltransferase.	NF	NF	NF	NF
1947	2.3.1.4	Glucosamine-phosphate N-acetyltransferase.	NF	NF	NF	NF
1948	2.4.1.16	Chitin synthase.	NF	NF	NF	NF
1949	2.4.1.43	Polygalacturonate 4-alpha-galacturonosyltransferas	NF	NF	NF	NF
1950	2.4.2.24	1,4-beta-D-xylan synthase.	NF	NF	NF	NF
1951	2.5.1.56	N-acetylneuraminate synthase.	NF	NF	NF	NF
1952	2.5.1.57	N-acylneuraminate-9-phosphate synthase.	NF	NF	NF	NF
1953	2.5.1.7	UDP-N-acetylglucosamine 1-carboxyvinyltransferase	FOUND	FOUND	FOUND	FOUND
1954	2.6.1.16	Glutamine--fructose-6-phosphate transaminase (iso	FOUND	NF	FOUND	FOUND
1955	2.6.1.87	UDP-4-amino-4-deoxy-L-arabinose aminotransferas	NF	NF	NF	NF
1956	2.7.1.1	Hexokinase.	NF	NF	NF	NF
1957	2.7.1.2	Glucokinase.	FOUND	FOUND	FOUND	FOUND
1958	2.7.1.4	Fructokinase.	NF	NF	FOUND	FOUND
1959	2.7.1.43	Glucuronokinase.	NF	NF	NF	NF
1960	2.7.1.44	Galacturonokinase.	NF	NF	NF	NF
1961	2.7.1.46	L-arabinokinase.	NF	NF	NF	NF
1962	2.7.1.52	Fucokinase.	NF	NF	NF	NF
1963	2.7.1.59	N-acetylglucosamine kinase.	NF	NF	NF	NF
1964	2.7.1.6	Galactokinase.	NF	FOUND	FOUND	FOUND
1965	2.7.1.60	N-acylmannosamine kinase.	NF	NF	NF	NF
1966	2.7.1.63	Polyphosphate--glucose phosphotransferase.	NF	NF	NF	NF
1967	2.7.1.69	Protein-N(pi)-phosphohistidine--sugar phosphotran	FOUND	FOUND	FOUND	FOUND
1968	2.7.1.7	Mannokinase.	NF	NF	NF	NF
1969	2.7.1.8	Glucosamine kinase.	NF	NF	NF	NF
1970	2.7.7.-	Nucleotidyltransferases.	FOUND	FOUND	FOUND	FOUND
1971	2.7.7.10	UTP--hexose-1-phosphate uridylyltransferase.	NF	NF	NF	NF
1972	2.7.7.11	UTP--xylose-1-phosphate uridylyltransferase.	NF	NF	NF	NF
1973	2.7.7.12	UDP-glucose--hexose-1-phosphate uridylyltransfera	FOUND	FOUND	FOUND	FOUND
1974	2.7.7.13	Mannose-1-phosphate guanylyltransferase.	FOUND	NF	FOUND	FOUND

1975	2.7.7.22	Mannose-1-phosphate guanylyltransferase (GDP).	FOUND	NF	FOUND	FOUND
1976	2.7.7.23	UDP-N-acetylglucosamine diphosphorylase.	FOUND	FOUND	FOUND	FOUND
1977	2.7.7.27	Glucose-1-phosphate adenylyltransferase.	FOUND	FOUND	FOUND	FOUND
1978	2.7.7.30	Fucose-1-phosphate guanylyltransferase.	NF	NF	NF	NF
1979	2.7.7.33	Glucose-1-phosphate cytidylyltransferase.	NF	NF	NF	NF
1980	2.7.7.34	Glucose-1-phosphate guanylyltransferase.	NF	NF	NF	NF
1981	2.7.7.43	N-acylneuraminate cytidylyltransferase.	NF	NF	NF	NF
1982	2.7.7.44	Glucuronate-1-phosphate uridylyltransferase.	NF	NF	NF	NF
1983	2.7.7.64	UTP-monosaccharide-1-phosphate uridylyltransfera	NF	NF	NF	NF
1984	2.7.7.9	UTP--glucose-1-phosphate uridylyltransferase.	FOUND	FOUND	FOUND	FOUND
1985	2.7.8.30	Undecaprenyl-phosphate 4-deoxy-4-formamido-L-a	NF	NF	NF	NF
1986	3.1.3.29	N-acylneuraminate-9-phosphatase.	NF	NF	NF	NF
1987	3.13.1.1	UDP-sulfoquinovose synthase.	NF	NF	NF	NF
1988	3.2.1.-	Glycosidases, i.e. enzymes hydrolyzing O- and S-glyc	FOUND	FOUND	FOUND	FOUND
1989	3.2.1.132	Chitosanase.	NF	NF	NF	NF
1990	3.2.1.14	Chitinase.	NF	NF	NF	NF
1991	3.2.1.37	Xylan 1,4-beta-xylosidase.	NF	NF	NF	NF
1992	3.2.1.52	Beta-N-acetylhexosaminidase.	NF	NF	NF	NF
1993	3.2.1.55	Alpha-N-arabinofuranosidase.	NF	NF	NF	NF
1994	3.4.1.-	???	NF	NF	NF	NF
1995	3.5.1.-	In linear amides.	FOUND	FOUND	FOUND	FOUND
1996	3.5.1.25	N-acetylglucosamine-6-phosphate deacetylase.	FOUND	FOUND	FOUND	FOUND
1997	3.5.1.33	N-acetylglucosamine deacetylase.	NF	NF	NF	NF
1998	3.5.1.41	Chitin deacetylase.	NF	NF	NF	NF
1999	3.5.99.6	Glucosamine-6-phosphate deaminase.	FOUND	FOUND	FOUND	FOUND
2000	4.1.1.35	UDP-glucuronate decarboxylase.	NF	NF	NF	NF
2001	4.1.1.67	UDP-galacturonate decarboxylase.	NF	NF	NF	NF
2002	4.1.3.3	N-acetylneuraminate lyase.	NF	FOUND	NF	NF
2003	4.2.-.-	Carbon-oxygen lyases.	FOUND	FOUND	FOUND	FOUND
2004	4.2.1.-	Hydro-lyases.	FOUND	FOUND	FOUND	FOUND
2005	4.2.1.45	CDP-glucose 4,6-dehydratase.	NF	NF	NF	NF
2006	4.2.1.47	GDP-mannose 4,6-dehydratase.	NF	NF	NF	NF
2007	4.2.1.76	UDP-glucose 4,6-dehydratase.	NF	NF	NF	NF
2008	5.1.-.-	Racemases and epimerases.	FOUND	FOUND	FOUND	FOUND
2009	5.1.3.-	Acting on carbohydrates and derivatives.	FOUND	FOUND	FOUND	FOUND
2010	5.1.3.10	CDP-paratose 2-epimerase.	NF	NF	NF	NF
2011	5.1.3.12	UDP-glucuronate 5'-epimerase.	NF	NF	NF	NF
2012	5.1.3.14	UDP-N-acetylglucosamine 2-epimerase.	NF	NF	NF	NF
2013	5.1.3.18	GDP-mannose 3,5-epimerase.	NF	NF	NF	NF
2014	5.1.3.2	UDP-glucose 4-epimerase.	NF	FOUND	FOUND	FOUND
2015	5.1.3.5	UDP-arabinose 4-epimerase.	NF	NF	NF	NF
2016	5.1.3.6	UDP-glucuronate 4-epimerase.	NF	NF	NF	NF
2017	5.1.3.8	N-acylglucosamine 2-epimerase.	NF	NF	NF	NF
2018	5.1.3.9	N-acylglucosamine-6-phosphate 2-epimerase.	NF	FOUND	NF	FOUND
2019	5.3.1.8	Mannose-6-phosphate isomerase.	FOUND	FOUND	FOUND	FOUND
2020	5.3.1.9	Glucose-6-phosphate isomerase.	FOUND	FOUND	FOUND	FOUND
2021	5.4.2.10	Phosphoglucosamine mutase.	FOUND	FOUND	FOUND	FOUND
2022	5.4.2.2	Phosphoglucomutase.	FOUND	FOUND	FOUND	FOUND
2023	5.4.2.3	Phosphoacetylglucosamine mutase.	NF	NF	NF	NF
2024	5.4.2.5	Phosphoglucomutase (glucose-cofactor).	NF	NF	NF	NF
2025	5.4.2.8	Phosphomannomutase.	NF	NF	NF	FOUND
2026	>Streptomycin biosynthesis 00521					
2027	1.1.1.133	dTDP-4-dehydrorhamnose reductase.	NF	NF	FOUND	FOUND
2028	1.1.1.18	Inositol 2-dehydrogenase.	NF	NF	NF	NF
2029	2.1.4.2	Scyllo-inosamine-4-phosphate amidinotransferase.	NF	NF	NF	NF

2030	2.4.2.27	dTDP-dihydrostreptose--streptidine-6-phosphate di	NF	NF	NF	NF
2031	2.6.1.50	Glutamine--scyllo-inositol transaminase.	NF	NF	NF	NF
2032	2.6.1.56	1D-1-guanidino-3-amino-1,3-dideoxy-scyllo-inositol	NF	NF	NF	NF
2033	2.7.1.1	Hexokinase.	NF	NF	NF	NF
2034	2.7.1.2	Glucokinase.	FOUND	FOUND	FOUND	FOUND
2035	2.7.1.65	Scyllo-inosamine 4-kinase.	NF	NF	NF	NF
2036	2.7.1.72	Streptomycin 6-kinase.	NF	NF	NF	NF
2037	2.7.7.24	Glucose-1-phosphate thymidylyltransferase.	NF	NF	FOUND	FOUND
2038	3.1.3.25	Inositol-phosphate phosphatase.	NF	NF	NF	NF
2039	3.1.3.39	Streptomycin-6-phosphatase.	NF	NF	NF	NF
2040	3.1.3.40	Guanidinodeoxy-scyllo-inositol-4-phosphatase.	NF	NF	NF	NF
2041	4.2.1.46	dTDP-glucose 4,6-dehydratase.	NF	NF	FOUND	FOUND
2042	5.1.3.13	dTDP-4-dehydrorhamnose 3,5-epimerase.	NF	NF	FOUND	FOUND
2043	5.4.2.2	Phosphoglucomutase.	FOUND	FOUND	FOUND	FOUND
2044	5.5.1.4	Inositol-3-phosphate synthase.	NF	NF	NF	NF
2045	>Biosynthesis of 12-, 14- and 16-membered macrolides 00522					
2046	1.14.-.-	Acting on paired donors, with incorporation or redu	NF	NF	NF	NF
2047	2.1.1.-	Methyltransferases.	FOUND	FOUND	FOUND	FOUND
2048	2.1.1.101	Macrocin O-methyltransferase.	NF	NF	NF	NF
2049	2.1.1.102	Demethylmacrocin O-methyltransferase.	NF	NF	NF	NF
2050	2.3.1.94	6-deoxyerythronolide-B synthase.	NF	NF	NF	NF
2051	2.4.1.-	Hexosyltransferases.	FOUND	FOUND	FOUND	FOUND
2052	>Polyketide sugar unit biosynthesis 00523					
2053	1.1.1.-	With NAD(+) or NADP(+) as acceptor.	FOUND	FOUND	FOUND	FOUND
2054	1.1.1.133	dTDP-4-dehydrorhamnose reductase.	NF	NF	FOUND	FOUND
2055	1.1.1.266	dTDP-4-dehydro-6-deoxyglucose reductase.	NF	NF	NF	NF
2056	2.6.1.33	dTDP-4-amino-4,6-dideoxy-D-glucose transaminase	NF	NF	NF	NF
2057	2.7.7.24	Glucose-1-phosphate thymidylyltransferase.	NF	NF	FOUND	FOUND
2058	4.2.1.46	dTDP-glucose 4,6-dehydratase.	NF	NF	FOUND	FOUND
2059	5.1.3.-	Acting on carbohydrates and derivatives.	FOUND	FOUND	FOUND	FOUND
2060	5.1.3.13	dTDP-4-dehydrorhamnose 3,5-epimerase.	NF	NF	FOUND	FOUND
2061	>Butirosin and neomycin biosynthesis 00524					
2062	1.1.-.-	Acting on the CH-OH group of donors.	FOUND	FOUND	FOUND	FOUND
2063	1.1.1.-	With NAD(+) or NADP(+) as acceptor.	FOUND	FOUND	FOUND	FOUND
2064	1.1.3.-	With oxygen as acceptor.	NF	NF	NF	FOUND
2065	1.14.14.-	With reduced flavin or flavoprotein as one donor, a	NF	NF	NF	NF
2066	1.5.1.29	FMN reductase.	NF	NF	NF	FOUND
2067	2.3.2.-	Aminoacyltransferases.	NF	NF	NF	NF
2068	2.4.1.-	Hexosyltransferases.	FOUND	FOUND	FOUND	FOUND
2069	2.4.2.-	Pentosyltransferases.	FOUND	FOUND	FOUND	FOUND
2070	2.6.1.-	Transaminases (aminotransferases).	FOUND	FOUND	FOUND	FOUND
2071	2.7.1.1	Hexokinase.	NF	NF	NF	NF
2072	2.7.1.2	Glucokinase.	FOUND	FOUND	FOUND	FOUND
2073	3.1.3.-	Phosphoric monoester hydrolases.	FOUND	FOUND	FOUND	FOUND
2074	3.5.1.-	In linear amides.	FOUND	FOUND	FOUND	FOUND
2075	4.1.1.-	Carboxy-lyases.	FOUND	FOUND	FOUND	FOUND
2076	4.2.3.-	Acting on phosphates.	NF	NF	FOUND	FOUND
2077	6.2.1.-	Acid--thiol ligases.	NF	NF	NF	NF
2078	>Glycosaminoglycan degradation 00531					
2079	2.3.1.78	Heparan-alpha-glucosaminide N-acetyltransferase.	NF	NF	NF	NF
2080	3.1.6.12	N-acetylgalactosamine-4-sulfatase.	NF	NF	NF	NF
2081	3.1.6.13	Iduronate-2-sulfatase.	NF	NF	NF	NF
2082	3.1.6.14	N-acetylglucosamine-6-sulfatase.	NF	FOUND	NF	NF
2083	3.1.6.18	Glucuronate-2-sulfatase.	NF	NF	NF	NF
2084	3.1.6.4	N-acetylgalactosamine-6-sulfatase.	NF	NF	NF	NF

2085	3.10.1.1	N-sulfoglucosamine sulfohydrolase.	NF	NF	NF	NF
2086	3.2.-.-	Glycosylases.	FOUND	FOUND	FOUND	FOUND
2087	3.2.1.-	Glycosidases, i.e. enzymes hydrolyzing O- and S-glyc	FOUND	FOUND	FOUND	FOUND
2088	3.2.1.103	Keratan-sulfate endo-1,4-beta-galactosidase.	NF	NF	NF	NF
2089	3.2.1.23	Beta-galactosidase.	NF	NF	NF	NF
2090	3.2.1.31	Beta-glucuronidase.	NF	NF	NF	NF
2091	3.2.1.35	Hyaluronoglucosaminidase.	NF	NF	NF	NF
2092	3.2.1.50	Alpha-N-acetylglucosaminidase.	NF	NF	NF	NF
2093	3.2.1.52	Beta-N-acetylhexosaminidase.	NF	NF	NF	NF
2094	3.2.1.76	L-iduronidase.	NF	NF	NF	NF
2095	>Glycosaminoglycan biosynthesis - chondroitin sulfate 00532					
2096	2.4.1.133	Xylosylprotein 4-beta-galactosyltransferase.	NF	NF	NF	NF
2097	2.4.1.134	Galactosylxylosylprotein 3-beta-galactosyltransfera	NF	NF	NF	NF
2098	2.4.1.135	Galactosylgalactosylxylosylprotein 3-beta-glucurono	NF	NF	NF	NF
2099	2.4.1.174	Glucuronylgalactosylproteoglycan 4-beta-N- acetylg	NF	NF	NF	NF
2100	2.4.1.175	Glucuronosyl-N-acetylgalactosaminyl-proteoglycan	NF	NF	NF	NF
2101	2.4.1.226	N-acetylgalactosaminyl-proteoglycan 3-beta-glucuro	NF	NF	NF	NF
2102	2.4.2.26	Protein xylosyltransferase.	NF	NF	NF	NF
2103	2.8.2.-	Sulfotransferases.	NF	NF	NF	NF
2104	2.8.2.17	Chondroitin 6-sulfotransferase.	NF	NF	NF	NF
2105	2.8.2.33	N-acetylgalactosamine 4-sulfate 6-O-sulfotransferas	NF	NF	NF	NF
2106	2.8.2.5	Chondroitin 4-sulfotransferase.	NF	NF	NF	NF
2107	5.1.3.19	Chondroitin-glucuronate 5-epimerase.	NF	NF	NF	NF
2108	>Glycosaminoglycan biosynthesis - keratan sulfate 00533					
2109	2.4.1.-	Hexosyltransferases.	FOUND	FOUND	FOUND	FOUND
2110	2.4.1.149	N-acetyllactosaminide beta-1,3-N-acetylglucosamin	NF	NF	NF	NF
2111	2.4.1.38	Beta-N-acetylglucosaminylglycopeptide beta-1,4-ga	NF	NF	NF	NF
2112	2.4.1.68	Glycoprotein 6-alpha-L-fucosyltransferase.	NF	NF	NF	NF
2113	2.4.99.4	Beta-galactoside alpha-2,3-sialyltransferase.	NF	NF	NF	NF
2114	2.4.99.6	N-acetyllactosaminide alpha-2,3-sialyltransferase.	NF	NF	NF	NF
2115	2.8.2.-	Sulfotransferases.	NF	NF	NF	NF
2116	2.8.2.21	Keratan sulfotransferase.	NF	NF	NF	NF
2117	>Glycosaminoglycan biosynthesis - heparan sulfate 00534					
2118	2.4.1.133	Xylosylprotein 4-beta-galactosyltransferase.	NF	NF	NF	NF
2119	2.4.1.134	Galactosylxylosylprotein 3-beta-galactosyltransfera	NF	NF	NF	NF
2120	2.4.1.135	Galactosylgalactosylxylosylprotein 3-beta-glucurono	NF	NF	NF	NF
2121	2.4.1.223	Glucuronyl-galactosyl-proteoglycan 4-alpha-N- acet	NF	NF	NF	NF
2122	2.4.1.224	Glucuronosyl-N-acetylglucosaminyl-proteoglycan 4-	NF	NF	NF	NF
2123	2.4.1.225	N-acetylglucosaminyl-proteoglycan 4-beta-glucuron	NF	NF	NF	NF
2124	2.4.2.26	Protein xylosyltransferase.	NF	NF	NF	NF
2125	2.8.2.-	Sulfotransferases.	NF	NF	NF	NF
2126	2.8.2.23	[Heparan sulfate]-glucosamine 3-sulfotransferase 1.	NF	NF	NF	NF
2127	2.8.2.29	[Heparan sulfate]-glucosamine 3-sulfotransferase 2.	NF	NF	NF	NF
2128	2.8.2.30	[Heparan sulfate]-glucosamine 3-sulfotransferase 3.	NF	NF	NF	NF
2129	5.1.3.17	Heparosan-N-sulfate-glucuronate 5-epimerase.	NF	NF	NF	NF
2130	>Lipopolysaccharide biosynthesis 00540					
2131	2.-.-.-	Transferases.	FOUND	FOUND	FOUND	FOUND
2132	2.3.1.-	Transferring groups other than amino-acyl groups.	FOUND	FOUND	FOUND	FOUND
2133	2.3.1.129	Acyl-[acyl-carrier-protein]--UDP-N-acetylglucosamin	NF	NF	FOUND	FOUND
2134	2.4.-.-	Glycosyltransferases.	FOUND	FOUND	FOUND	FOUND
2135	2.4.1.-	Hexosyltransferases.	FOUND	FOUND	FOUND	FOUND
2136	2.4.1.182	Lipid-A-disaccharide synthase.	NF	NF	FOUND	FOUND
2137	2.4.1.44	Lipopolysaccharide 3-alpha-galactosyltransferase.	NF	NF	NF	NF
2138	2.4.1.56	Lipopolysaccharide N-acetylglucosaminyltransferase	NF	NF	NF	NF
2139	2.4.1.58	Lipopolysaccharide glucosyltransferase I.	NF	NF	NF	NF

2140	2.5.1.55	3-deoxy-8-phosphooctulonate synthase.	NF	NF	FOUND	FOUND
2141	2.7.-.-	Transferring phosphorous-containing groups.	FOUND	FOUND	FOUND	FOUND
2142	2.7.1.-	Phosphotransferases with an alcohol group as accep	FOUND	FOUND	FOUND	FOUND
2143	2.7.1.130	Tetraacyldisaccharide 4'-kinase.	NF	NF	FOUND	FOUND
2144	2.7.7.-	Nucleotidyltransferases.	FOUND	FOUND	FOUND	FOUND
2145	2.7.7.38	3-deoxy-manno-octulosonate cytidylyltransferase.	NF	NF	FOUND	FOUND
2146	3.1.3.-	Phosphoric monoester hydrolases.	FOUND	FOUND	FOUND	FOUND
2147	3.1.3.45	3-deoxy-manno-octulosonate-8-phosphatase.	NF	NF	FOUND	NF
2148	3.5.1.-	In linear amides.	FOUND	FOUND	FOUND	FOUND
2149	3.6.1.-	In phosphorous-containing anhydrides.	FOUND	FOUND	FOUND	FOUND
2150	5.-.-.-	Isomerases.	FOUND	FOUND	FOUND	FOUND
2151	5.1.3.20	ADP-glyceromanno-heptose 6-epimerase.	NF	NF	NF	FOUND
2152	6.-.-.-	Ligases.	FOUND	FOUND	FOUND	FOUND
2153	>Peptidoglycan biosynthesis 00550					
2154	1.1.1.158	UDP-N-acetylmuramate dehydrogenase.	FOUND	FOUND	FOUND	FOUND
2155	2.3.2.-	Aminoacyltransferases.	NF	NF	NF	NF
2156	2.3.2.10	UDP-N-acetylmuramoylpentapeptide-lysine N(6)-ala	NF	NF	NF	NF
2157	2.4.1.-	Hexosyltransferases.	FOUND	FOUND	FOUND	FOUND
2158	2.4.1.129	Peptidoglycan glycosyltransferase.	FOUND	FOUND	FOUND	FOUND
2159	2.4.1.227	Undecaprenyldiphospho-muramoylpentapeptide be	NF	NF	NF	NF
2160	2.5.1.7	UDP-N-acetylglucosamine 1-carboxyvinyltransferase	FOUND	FOUND	FOUND	FOUND
2161	2.7.1.66	Undecaprenol kinase.	NF	NF	NF	NF
2162	2.7.8.13	Phospho-N-acetylmuramoyl-pentapeptide-transfera	FOUND	FOUND	FOUND	FOUND
2163	3.4.-.-	Acting on peptide bonds (peptide hydrolases).	FOUND	FOUND	FOUND	FOUND
2164	3.4.16.4	Serine-type D-Ala-D-Ala carboxypeptidase.	FOUND	FOUND	FOUND	FOUND
2165	3.6.1.27	Undecaprenyl-diphosphatase.	NF	NF	NF	NF
2166	6.3.2.10	UDP-N-acetylmuramoyl-tripeptide--D-alanyl-D-alani	NF	NF	NF	NF
2167	6.3.2.13	UDP-N-acetylmuramoyl-L-alanyl-D-glutamate--2,6-d	NF	NF	FOUND	NF
2168	6.3.2.4	D-alanine--D-alanine ligase.	FOUND	FOUND	FOUND	FOUND
2169	6.3.2.7	UDP-N-acetylmuramoyl-L-alanyl-D-glutamate--L-lysi	NF	NF	NF	NF
2170	6.3.2.8	UDP-N-acetylmuramate--L-alanine ligase.	FOUND	FOUND	FOUND	FOUND
2171	6.3.2.9	UDP-N-acetylmuramoyl-L-alanine--D-glutamate liga	FOUND	FOUND	FOUND	FOUND
2172	>Glycerolipid metabolism 00561					
2173	1.1.1.156	Glycerol 2-dehydrogenase (NADP(+)).	NF	NF	NF	NF
2174	1.1.1.2	Alcohol dehydrogenase (NADP(+)).	NF	NF	FOUND	NF
2175	1.1.1.202	1,3-propanediol dehydrogenase.	NF	NF	NF	NF
2176	1.1.1.21	Aldehyde reductase.	NF	NF	FOUND	FOUND
2177	1.1.1.6	Glycerol dehydrogenase.	NF	NF	FOUND	FOUND
2178	1.1.1.72	Glycerol dehydrogenase (NADP(+)).	NF	NF	NF	NF
2179	1.2.1.3	Aldehyde dehydrogenase (NAD(+)).	NF	NF	FOUND	FOUND
2180	2.3.1.134	Galactolipid O-acyltransferase.	NF	NF	NF	NF
2181	2.3.1.141	Galactosylacylglycerol O-acyltransferase.	NF	NF	NF	NF
2182	2.3.1.15	Glycerol-3-phosphate O-acyltransferase.	NF	NF	NF	NF
2183	2.3.1.158	Phospholipid:diacylglycerol acyltransferase.	NF	NF	NF	NF
2184	2.3.1.20	Diacylglycerol O-acyltransferase.	NF	NF	NF	NF
2185	2.3.1.22	2-acylglycerol O-acyltransferase.	NF	NF	NF	NF
2186	2.3.1.51	1-acylglycerol-3-phosphate O-acyltransferase.	NF	NF	FOUND	FOUND
2187	2.4.1.-	Hexosyltransferases.	FOUND	FOUND	FOUND	FOUND
2188	2.4.1.157	1,2-diacylglycerol 3-glucosyltransferase.	NF	NF	NF	NF
2189	2.4.1.184	Galactolipid galactosyltransferase.	NF	NF	NF	NF
2190	2.4.1.241	Digalactosyldiacylglycerol synthase.	NF	NF	NF	NF
2191	2.4.1.46	Monogalactosyldiacylglycerol synthase.	NF	NF	NF	NF
2192	2.4.99.5	Galactosyldiacylglycerol alpha-2,3-sialyltransferase.	NF	NF	NF	NF
2193	2.7.1.107	Diacylglycerol kinase.	FOUND	FOUND	FOUND	FOUND
2194	2.7.1.29	Glycerone kinase.	FOUND	FOUND	FOUND	FOUND

2195	2.7.1.30	Glycerol kinase.	NF	NF	FOUND	FOUND
2196	2.7.1.31	Glycerate kinase.	FOUND	NF	NF	FOUND
2197	2.7.1.94	Acylglycerol kinase.	NF	NF	NF	NF
2198	2.7.8.20	Phosphatidylglycerol--membrane-oligosaccharide gl	NF	NF	NF	NF
2199	3.1.1.23	Acylglycerol lipase.	NF	NF	NF	NF
2200	3.1.1.26	Galactolipase.	NF	NF	NF	NF
2201	3.1.1.3	Triacylglycerol lipase.	NF	NF	FOUND	NF
2202	3.1.1.34	Lipoprotein lipase.	NF	NF	NF	NF
2203	3.1.3.21	Glycerol-1-phosphatase.	NF	NF	NF	NF
2204	3.1.3.4	Phosphatidate phosphatase.	NF	NF	NF	NF
2205	3.13.1.1	UDP-sulfoquinovose synthase.	NF	NF	NF	NF
2206	3.2.1.22	Alpha-galactosidase.	NF	NF	NF	NF
2207	4.2.1.28	Propanediol dehydratase.	NF	NF	NF	NF
2208	4.2.1.30	Glycerol dehydratase.	NF	NF	NF	NF
2209	>Inositol phosphate metabolism 00562					
2210	1.1.1.18	Inositol 2-dehydrogenase.	NF	NF	NF	NF
2211	1.13.99.1	Inositol oxygenase.	NF	NF	NF	NF
2212	1.2.1.18	Malonate-semialdehyde dehydrogenase (acetylatin	NF	NF	NF	NF
2213	1.2.1.27	Methylmalonate-semialdehyde dehydrogenase (acy	NF	NF	NF	FOUND
2214	2.1.1.39	Inositol 3-methyltransferase.	NF	NF	NF	NF
2215	2.1.1.40	Inositol 1-methyltransferase.	NF	NF	NF	NF
2216	2.7.1.127	Inositol-trisphosphate 3-kinase.	NF	NF	NF	NF
2217	2.7.1.134	Inositol-tetrakisphosphate 1-kinase.	NF	NF	NF	NF
2218	2.7.1.137	Phosphatidylinositol 3-kinase.	NF	NF	NF	NF
2219	2.7.1.140	Inositol-tetrakisphosphate 5-kinase.	NF	NF	NF	NF
2220	2.7.1.149	1-phosphatidylinositol-5-phosphate 4-kinase.	NF	NF	NF	NF
2221	2.7.1.150	1-phosphatidylinositol-3-phosphate 5-kinase.	NF	NF	NF	NF
2222	2.7.1.151	Inositol-polyphosphate multikinase.	NF	NF	NF	NF
2223	2.7.1.153	Phosphatidylinositol-4,5-bisphosphate 3-kinase.	NF	NF	NF	NF
2224	2.7.1.154	Phosphatidylinositol-4-phosphate 3-kinase.	NF	NF	NF	NF
2225	2.7.1.158	Inositol-pentakisphosphate 2-kinase.	NF	NF	NF	NF
2226	2.7.1.159	Inositol-1,3,4-trisphosphate 5/6-kinase.	NF	NF	NF	NF
2227	2.7.1.64	Inositol 3-kinase.	NF	NF	NF	NF
2228	2.7.1.67	1-phosphatidylinositol 4-kinase.	NF	NF	NF	NF
2229	2.7.1.68	1-phosphatidylinositol-4-phosphate 5-kinase.	NF	NF	NF	NF
2230	2.7.1.92	5-dehydro-2-deoxygluconokinase.	NF	NF	NF	FOUND
2231	2.7.8.11	CDP-diacylglycerol--inositol 3-phosphatidyltransfera	NF	NF	NF	NF
2232	3.1.3.25	Inositol-phosphate phosphatase.	NF	NF	NF	NF
2233	3.1.3.26	4-phytase.	NF	NF	FOUND	NF
2234	3.1.3.36	Phosphoinositide 5-phosphatase.	NF	NF	NF	NF
2235	3.1.3.56	Inositol-polyphosphate 5-phosphatase.	NF	NF	NF	NF
2236	3.1.3.57	Inositol-1,4-bisphosphate 1-phosphatase.	NF	NF	NF	NF
2237	3.1.3.62	Multiple inositol-polyphosphate phosphatase.	NF	NF	NF	NF
2238	3.1.3.64	Phosphatidylinositol-3-phosphatase.	NF	NF	NF	NF
2239	3.1.3.66	Phosphatidylinositol-3,4-bisphosphate 4-phosphata	NF	NF	NF	NF
2240	3.1.3.67	Phosphatidylinositol-3,4,5-trisphosphate 3-phospha	NF	NF	NF	NF
2241	3.1.3.8	3-phytase.	NF	NF	NF	NF
2242	3.1.4.11	Phosphoinositide phospholipase C.	NF	NF	NF	NF
2243	3.1.4.3	Phospholipase C.	NF	NF	NF	NF
2244	3.7.1.-	In ketonic substances.	NF	NF	NF	FOUND
2245	4.1.2.29	5-dehydro-2-deoxyphosphogluconate aldolase.	NF	NF	NF	NF
2246	4.2.1.44	Myo-inosose-2 dehydratase.	NF	NF	NF	FOUND
2247	4.6.1.13	Phosphatidylinositol diacylglycerol-lyase.	NF	NF	NF	NF
2248	5.3.1.-	Interconverting aldoses and ketoses, and related co	FOUND	FOUND	FOUND	FOUND
2249	5.3.1.1	Triose-phosphate isomerase.	FOUND	FOUND	FOUND	FOUND

2250	5.5.1.4	Inositol-3-phosphate synthase.	NF	NF	NF	NF
2251	>Glycosylphosphatidylinositol(GPI)-anchor biosynthesis 00563					
2252	2.3.-.-	Acyltransferases.	FOUND	FOUND	FOUND	FOUND
2253	2.4.1.-	Hexosyltransferases.	FOUND	FOUND	FOUND	FOUND
2254	2.4.1.198	Phosphatidylinositol N-acetylglucosaminyltransfera	NF	NF	NF	NF
2255	2.6.-.-	Transferring nitrogenous groups.	FOUND	FOUND	FOUND	FOUND
2256	2.7.-.-	Transferring phosphorous-containing groups.	FOUND	FOUND	FOUND	FOUND
2257	3.-.-.-	Hydrolases.	FOUND	FOUND	FOUND	FOUND
2258	3.1.4.50	Glycosylphosphatidylinositol phospholipase D.	NF	NF	NF	NF
2259	3.5.1.89	N-acetylglucosaminylphosphatidylinositol deacetyla	NF	NF	NF	NF
2260	>Glycerophospholipid metabolism 00564					
2261	1.1.1.101	Acylglycerone-phosphate reductase.	NF	NF	NF	NF
2262	1.1.1.8	Glycerol-3-phosphate dehydrogenase (NAD(+)).	FOUND	FOUND	FOUND	FOUND
2263	1.1.1.94	Glycerol-3-phosphate dehydrogenase (NAD(P)(+)).	NF	NF	NF	NF
2264	1.1.3.21	Glycerol-3-phosphate oxidase.	NF	NF	NF	NF
2265	1.1.5.3	Glycerol-3-phosphate dehydrogenase.	NF	NF	NF	NF
2266	2.1.1.103	Phosphoethanolamine N-methyltransferase.	NF	NF	NF	NF
2267	2.1.1.17	Phosphatidylethanolamine N-methyltransferase.	NF	NF	NF	NF
2268	2.1.1.71	Phosphatidyl-N-methylethanolamine N-methyltrans	NF	NF	NF	NF
2269	2.3.1.-	Transferring groups other than amino-acyl groups.	FOUND	FOUND	FOUND	FOUND
2270	2.3.1.15	Glycerol-3-phosphate O-acyltransferase.	NF	NF	NF	NF
2271	2.3.1.23	1-acylglycerophosphocholine O-acyltransferase.	NF	NF	NF	NF
2272	2.3.1.40	Acyl-[acyl-carrier-protein]--phospholipid O-acyltran	NF	NF	NF	NF
2273	2.3.1.42	Glycerone-phosphate O-acyltransferase.	NF	NF	NF	NF
2274	2.3.1.43	Phosphatidylcholine--sterol O-acyltransferase.	NF	NF	NF	NF
2275	2.3.1.51	1-acylglycerol-3-phosphate O-acyltransferase.	NF	NF	FOUND	FOUND
2276	2.3.1.52	2-acylglycerol-3-phosphate O-acyltransferase.	NF	NF	NF	NF
2277	2.3.1.6	Choline O-acetyltransferase.	NF	NF	NF	NF
2278	2.3.1.62	2-acylglycerophosphocholine O-acyltransferase.	NF	NF	NF	NF
2279	2.7.1.107	Diacylglycerol kinase.	FOUND	FOUND	FOUND	FOUND
2280	2.7.1.32	Choline kinase.	NF	NF	NF	NF
2281	2.7.1.82	Ethanolamine kinase.	NF	NF	NF	NF
2282	2.7.7.14	Ethanolamine-phosphate cytidylyltransferase.	NF	NF	NF	NF
2283	2.7.7.15	Choline-phosphate cytidylyltransferase.	NF	NF	NF	NF
2284	2.7.7.39	Glycerol-3-phosphate cytidylyltransferase.	NF	NF	NF	NF
2285	2.7.7.41	Phosphatidate cytidylyltransferase.	FOUND	FOUND	FOUND	FOUND
2286	2.7.8.-	Transferases for other substituted phosphate group	FOUND	FOUND	FOUND	FOUND
2287	2.7.8.1	Ethanolaminephosphotransferase.	NF	NF	NF	NF
2288	2.7.8.11	CDP-diacylglycerol--inositol 3-phosphatidyltransfera	NF	NF	NF	NF
2289	2.7.8.2	Diacylglycerol cholinephosphotransferase.	NF	NF	NF	NF
2290	2.7.8.24	Phosphatidylcholine synthase.	NF	NF	NF	NF
2291	2.7.8.4	Serine-phosphoethanolamine synthase.	NF	NF	NF	NF
2292	2.7.8.5	CDP-diacylglycerol--glycerol-3-phosphate 3-phospha	FOUND	FOUND	FOUND	FOUND
2293	2.7.8.8	CDP-diacylglycerol--serine O-phosphatidyltransfera	NF	NF	NF	NF
2294	3.1.1.-	Carboxylic ester hydrolases.	FOUND	FOUND	FOUND	FOUND
2295	3.1.1.32	Phospholipase A(1).	NF	NF	NF	NF
2296	3.1.1.4	Phospholipase A(2).	NF	NF	NF	NF
2297	3.1.1.5	Lysophospholipase.	NF	NF	NF	NF
2298	3.1.1.52	Phosphatidylinositol deacylase.	NF	NF	NF	NF
2299	3.1.1.7	Acetylcholinesterase.	NF	NF	NF	NF
2300	3.1.3.27	Phosphatidylglycerophosphatase.	NF	NF	NF	NF
2301	3.1.3.4	Phosphatidate phosphatase.	NF	NF	NF	NF
2302	3.1.3.75	Phosphoethanolamine/phosphocholine phosphatas	NF	NF	NF	NF
2303	3.1.4.13	Serine-ethanolaminephosphate phosphodiesterase.	NF	NF	NF	NF
2304	3.1.4.2	Glycerophosphocholine phosphodiesterase.	NF	NF	NF	NF

2305	3.1.4.3	Phospholipase C.	NF	NF	NF	NF
2306	3.1.4.4	Phospholipase D.	NF	NF	NF	NF
2307	3.1.4.46	Glycerophosphodiester phosphodiesterase.	NF	NF	NF	NF
2308	3.6.1.16	CDP-glycerol diphosphatase.	NF	NF	NF	NF
2309	3.6.1.26	CDP-diacylglycerol diphosphatase.	NF	NF	NF	NF
2310	4.1.1.65	Phosphatidylserine decarboxylase.	NF	NF	NF	FOUND
2311	4.3.1.7	Ethanolamine ammonia-lyase.	NF	NF	FOUND	FOUND
2312	4.3.3.-	Amine-lyases.	NF	NF	NF	NF
2313	>Ether lipid metabolism 00565					
2314	1.1.1.101	Acylglycerone-phosphate reductase.	NF	NF	NF	NF
2315	1.14.99.19	Plasmanylethanolamine desaturase.	NF	NF	NF	NF
2316	2.3.1.-	Transferring groups other than amino-acyl groups.	FOUND	FOUND	FOUND	FOUND
2317	2.3.1.104	1-alkenylglycerophosphocholine O-acyltransferase.	NF	NF	NF	NF
2318	2.3.1.105	Alkylglycerophosphate 2-O-acetyltransferase.	NF	NF	NF	NF
2319	2.3.1.121	1-alkenylglycerophosphoethanolamine O-acyltransf	NF	NF	NF	NF
2320	2.3.1.125	1-alkyl-2-acetylglycerol O-acyltransferase.	NF	NF	NF	NF
2321	2.3.1.25	Plasmalogen synthase.	NF	NF	NF	NF
2322	2.3.1.63	1-alkylglycerophosphocholine O-acyltransferase.	NF	NF	NF	NF
2323	2.3.1.67	1-alkylglycerophosphocholine O-acetyltransferase.	NF	NF	NF	NF
2324	2.5.1.26	Alkylglycerone-phosphate synthase.	NF	NF	NF	NF
2325	2.6.-.-	Transferring nitrogenous groups.	FOUND	FOUND	FOUND	FOUND
2326	2.7.1.93	Alkylglycerol kinase.	NF	NF	NF	NF
2327	2.7.8.1	Ethanolaminephosphotransferase.	NF	NF	NF	NF
2328	2.7.8.2	Diacylglycerol cholinephosphotransferase.	NF	NF	NF	NF
2329	2.7.8.22	1-alkenyl-2-acylglycerol choline phosphotransferase	NF	NF	NF	NF
2330	3.1.1.4	Phospholipase A(2).	NF	NF	NF	NF
2331	3.1.1.47	1-alkyl-2-acetylglycerophosphocholine esterase.	NF	NF	NF	NF
2332	3.1.3.-	Phosphoric monoester hydrolases.	FOUND	FOUND	FOUND	FOUND
2333	3.1.3.4	Phosphatidate phosphatase.	NF	NF	NF	NF
2334	3.1.3.59	Alkylacetylglycerophosphatase.	NF	NF	NF	NF
2335	3.1.4.-	Phosphoric diester hydrolases.	FOUND	FOUND	FOUND	FOUND
2336	3.1.4.3	Phospholipase C.	NF	NF	NF	NF
2337	3.1.4.39	Alkylglycerophosphoethanolamine phosphodiester	NF	NF	NF	NF
2338	3.1.4.4	Phospholipase D.	NF	NF	NF	NF
2339	3.3.2.2	Alkenylglycerophosphocholine hydrolase.	NF	NF	NF	NF
2340	3.3.2.5	Alkenylglycerophosphoethanolamine hydrolase.	NF	NF	NF	NF
2341	>Arachidonic acid metabolism 00590					
2342	1.1.1.184	Carbonyl reductase (NADPH).	NF	NF	NF	NF
2343	1.1.1.188	Prostaglandin-F synthase.	NF	NF	NF	NF
2344	1.1.1.189	Prostaglandin-E(2) 9-reductase.	NF	NF	NF	NF
2345	1.1.1.196	15-hydroxyprostaglandin-D dehydrogenase (NADP(-	NF	NF	NF	NF
2346	1.1.1.232	15-hydroxyicosatetraenoate dehydrogenase.	NF	NF	NF	NF
2347	1.11.1.9	Glutathione peroxidase.	NF	NF	NF	NF
2348	1.13.11.-	With incorporation of two atoms of oxygen.	NF	NF	NF	NF
2349	1.13.11.31	Arachidonate 12-lipoxygenase.	NF	NF	NF	NF
2350	1.13.11.33	Arachidonate 15-lipoxygenase.	NF	NF	NF	NF
2351	1.13.11.34	Arachidonate 5-lipoxygenase.	NF	NF	NF	NF
2352	1.13.11.40	Arachidonate 8-lipoxygenase.	NF	NF	NF	NF
2353	1.14.13.30	Leukotriene-B(4) 20-monooxygenase.	NF	NF	NF	NF
2354	1.14.13.34	Leukotriene-E(4) 20-monooxygenase.	NF	NF	NF	NF
2355	1.14.14.1	Unspecific monooxygenase.	NF	NF	NF	NF
2356	1.14.15.3	Alkane 1-monooxygenase.	NF	NF	NF	NF
2357	1.14.99.1	Prostaglandin-endoperoxide synthase.	NF	NF	NF	NF
2358	2.3.2.-	Aminoacyltransferases.	NF	NF	NF	NF
2359	2.3.2.2	Gamma-glutamyltransferase.	NF	NF	NF	NF

2360	3.1.1.4	Phospholipase A(2).	NF	NF	NF	NF
2361	3.3.2.-	Ether hydrolases.	NF	NF	NF	FOUND
2362	3.3.2.10	Soluble epoxide hydrolase.	NF	NF	NF	NF
2363	3.3.2.6	Leukotriene-A(4) hydrolase.	NF	NF	NF	NF
2364	3.3.2.7	Hepoxilin-epoxide hydrolase.	NF	NF	NF	NF
2365	4.4.1.20	Leukotriene-C(4) synthase.	NF	NF	NF	NF
2366	5.3.99.2	Prostaglandin-D synthase.	NF	NF	NF	NF
2367	5.3.99.3	Prostaglandin-E synthase.	NF	NF	NF	NF
2368	5.3.99.4	Prostaglandin-I synthase.	NF	NF	NF	NF
2369	5.3.99.5	Thromboxane-A synthase.	NF	NF	NF	NF
2370	5.4.4.-	Transferring hydroxy groups.	NF	NF	NF	NF
2371	>Linoleic acid metabolism 00591					
2372	1.1.1.-	With NAD(+) or NADP(+) as acceptor.	FOUND	FOUND	FOUND	FOUND
2373	1.13.11.12	Lipoxygenase.	NF	NF	NF	NF
2374	1.13.11.33	Arachidonate 15-lipoxygenase.	NF	NF	NF	NF
2375	1.13.11.44	Linoleate diol synthase.	NF	NF	NF	NF
2376	1.13.11.45	Linoleate 11-lipoxygenase.	NF	NF	NF	NF
2377	1.14.14.1	Unspecific monooxygenase.	NF	NF	NF	NF
2378	1.14.19.3	Linoleoyl-CoA desaturase.	NF	NF	NF	NF
2379	1.14.99.33	Delta(12)-fatty acid dehydrogenase.	NF	NF	NF	NF
2380	3.1.1.4	Phospholipase A(2).	NF	NF	NF	NF
2381	3.3.2.-	Ether hydrolases.	NF	NF	NF	FOUND
2382	5.2.1.5	Linoleate isomerase.	NF	NF	NF	NF
2383	>alpha-Linolenic acid metabolism 00592					
2384	1.-.-.-	Oxidoreductases.	FOUND	FOUND	FOUND	FOUND
2385	1.13.11.12	Lipoxygenase.	NF	NF	NF	NF
2386	1.14.19.-	With oxidation of a pair of donors resulting in the re	NF	NF	NF	NF
2387	1.3.1.42	12-oxophytodienoate reductase.	NF	NF	NF	NF
2388	1.3.3.6	Acyl-CoA oxidase.	NF	NF	NF	NF
2389	2.1.1.141	Jasmonate O-methyltransferase.	NF	NF	NF	NF
2390	2.3.1.16	Acetyl-CoA C-acyltransferase.	NF	NF	NF	NF
2391	3.1.1.32	Phospholipase A(1).	NF	NF	NF	NF
2392	3.1.1.4	Phospholipase A(2).	NF	NF	NF	NF
2393	3.1.2.-	Thiolester hydrolases.	NF	FOUND	NF	NF
2394	4.1.2.-	Aldehyde-lyases.	FOUND	FOUND	FOUND	FOUND
2395	4.2.1.17	Enoyl-CoA hydratase.	NF	NF	NF	NF
2396	4.2.1.92	Hydroperoxide dehydratase.	NF	NF	NF	NF
2397	5.3.3.-	Transposing C==C bonds.	FOUND	FOUND	FOUND	FOUND
2398	5.3.99.6	Allene-oxide cyclase.	NF	NF	NF	NF
2399	6.2.1.-	Acid--thiol ligases.	NF	NF	NF	NF
2400	>Sphingolipid metabolism 00600					
2401	1.1.1.102	3-dehydrosphinganine reductase.	NF	NF	NF	NF
2402	1.14.-.-	Acting on paired donors, with incorporation or redu	NF	NF	NF	NF
2403	2.3.1.24	Sphingosine N-acyltransferase.	NF	NF	NF	NF
2404	2.3.1.50	Serine C-palmitoyltransferase.	NF	NF	NF	NF
2405	2.4.1.-	Hexosyltransferases.	FOUND	FOUND	FOUND	FOUND
2406	2.4.1.23	Sphingosine beta-galactosyltransferase.	NF	NF	NF	NF
2407	2.4.1.47	N-acylsphingosine galactosyltransferase.	NF	NF	NF	NF
2408	2.4.1.80	Ceramide glucosyltransferase.	NF	NF	FOUND	FOUND
2409	2.7.1.138	Ceramide kinase.	NF	NF	NF	NF
2410	2.7.1.91	Sphinganine kinase.	NF	NF	NF	NF
2411	2.7.8.-	Transferases for other substituted phosphate group	FOUND	FOUND	FOUND	FOUND
2412	2.7.8.10	Sphingosine cholinephosphotransferase.	NF	NF	NF	NF
2413	2.7.8.27	Sphingomyelin synthase.	NF	NF	NF	NF
2414	2.7.8.3	Ceramide cholinephosphotransferase.	NF	NF	NF	NF

2415	2.8.2.11	Galactosylceramide sulfotransferase.	NF	NF	NF	NF
2416	3.1.3.-	Phosphoric monoester hydrolases.	FOUND	FOUND	FOUND	FOUND
2417	3.1.3.4	Phosphatidate phosphatase.	NF	NF	NF	NF
2418	3.1.4.12	Sphingomyelin phosphodiesterase.	NF	NF	NF	NF
2419	3.1.4.41	Sphingomyelin phosphodiesterase D.	NF	NF	NF	NF
2420	3.1.6.1	Arylsulfatase.	NF	NF	NF	FOUND
2421	3.1.6.8	Cerebroside-sulfatase.	NF	NF	NF	NF
2422	3.2.1.18	Exo-alpha-sialidase.	NF	FOUND	NF	NF
2423	3.2.1.22	Alpha-galactosidase.	NF	NF	NF	NF
2424	3.2.1.23	Beta-galactosidase.	NF	NF	NF	NF
2425	3.2.1.45	Glucosylceramidase.	NF	NF	NF	NF
2426	3.2.1.46	Galactosylceramidase.	NF	NF	NF	NF
2427	3.5.1.-	In linear amides.	FOUND	FOUND	FOUND	FOUND
2428	3.5.1.23	Ceramidase.	NF	NF	NF	NF
2429	4.1.2.27	Sphinganine-1-phosphate aldolase.	NF	NF	NF	NF
2430	>Glycosphingolipid biosynthesis - lacto and neolacto series 00601					
2431	2.4.1.-	Hexosyltransferases.	FOUND	FOUND	FOUND	FOUND
2432	2.4.1.149	N-acetyllactosaminide beta-1,3-N-acetylglucosamin	NF	NF	NF	NF
2433	2.4.1.150	N-acetyllactosaminide beta-1,6-N-acetylglucosamin	NF	NF	NF	NF
2434	2.4.1.152	4-galactosyl-N-acetylglucosaminide 3-alpha-L-fucosy	NF	NF	NF	NF
2435	2.4.1.163	Beta-galactosyl-N-acetylglucosaminylgalactosylgluc	NF	NF	NF	NF
2436	2.4.1.206	Lactosylceramide 1,3-N-acetyl-beta-D-glucosaminyl	NF	NF	NF	NF
2437	2.4.1.37	Fucosylgalactoside 3-alpha-galactosyltransferase.	NF	NF	NF	NF
2438	2.4.1.40	Glycoprotein-fucosylgalactoside alpha-N-acetylgala	NF	NF	NF	NF
2439	2.4.1.65	3-galactosyl-N-acetylglucosaminide 4-alpha-L-fucos	NF	NF	NF	NF
2440	2.4.1.69	Galactoside 2-alpha-L-fucosyltransferase.	NF	NF	NF	NF
2441	2.4.1.86	Glucosaminylgalactosylglucosylceramide beta-galac	NF	NF	NF	NF
2442	2.4.1.87	N-acetyllactosaminide 3-alpha-galactosyltransferase	NF	NF	NF	NF
2443	2.4.99.-	Transferring other glycosyl groups.	NF	NF	NF	NF
2444	2.4.99.10	Neolactotetraosylceramide alpha-2,3-sialyltransfera	NF	NF	NF	NF
2445	2.4.99.4	Beta-galactoside alpha-2,3-sialyltransferase.	NF	NF	NF	NF
2446	2.4.99.6	N-acetyllactosaminide alpha-2,3-sialyltransferase.	NF	NF	NF	NF
2447	2.4.99.8	Alpha-N-acetylneuraminate alpha-2,8-sialyltransfera	NF	NF	NF	NF
2448	>Glycosphingolipid biosynthesis - globo series 00603					
2449	2.4.1.-	Hexosyltransferases.	FOUND	FOUND	FOUND	FOUND
2450	2.4.1.152	4-galactosyl-N-acetylglucosaminide 3-alpha-L-fucosy	NF	NF	NF	NF
2451	2.4.1.179	Lactosylceramide beta-1,3-galactosyltransferase.	NF	NF	NF	NF
2452	2.4.1.228	Lactosylceramide 4-alpha-galactosyltransferase.	NF	NF	NF	NF
2453	2.4.1.69	Galactoside 2-alpha-L-fucosyltransferase.	NF	NF	NF	NF
2454	2.4.1.79	Globotriaosylceramide 3-beta-N-acetylgalactosamin	NF	NF	NF	NF
2455	2.4.1.88	Globoside alpha-N-acetylgalactosaminyltransferase	NF	NF	NF	NF
2456	2.4.99.-	Transferring other glycosyl groups.	NF	NF	NF	NF
2457	2.4.99.4	Beta-galactoside alpha-2,3-sialyltransferase.	NF	NF	NF	NF
2458	2.4.99.8	Alpha-N-acetylneuraminate alpha-2,8-sialyltransfera	NF	NF	NF	NF
2459	3.2.1.22	Alpha-galactosidase.	NF	NF	NF	NF
2460	3.2.1.47	Galactosylgalactosylglucosylceramidase.	NF	NF	NF	NF
2461	3.2.1.49	Alpha-N-acetylgalactosaminidase.	NF	NF	NF	NF
2462	3.2.1.52	Beta-N-acetylhexosaminidase.	NF	NF	NF	NF
2463	>Glycosphingolipid biosynthesis - ganglio series 00604					
2464	2.3.1.-	Transferring groups other than amino-acyl groups.	FOUND	FOUND	FOUND	FOUND
2465	2.4.1.-	Hexosyltransferases.	FOUND	FOUND	FOUND	FOUND
2466	2.4.1.62	Ganglioside galactosyltransferase.	NF	NF	NF	NF
2467	2.4.1.92	(N-acetylneuraminyl)-galactosylglucosylceramide N-	NF	NF	NF	NF
2468	2.4.99.-	Transferring other glycosyl groups.	NF	NF	NF	NF
2469	2.4.99.4	Beta-galactoside alpha-2,3-sialyltransferase.	NF	NF	NF	NF

2470	2.4.99.7	Alpha-N-acetylneuraminyl-2,3-beta-galactosyl-1,3-N	NF	NF	NF	NF
2471	2.4.99.8	Alpha-N-acetylneuraminate alpha-2,8-sialyltransfera	NF	NF	NF	NF
2472	2.4.99.9	Lactosylceramide alpha-2,3-sialyltransferase.	NF	NF	NF	NF
2473	3.2.1.23	Beta-galactosidase.	NF	NF	NF	NF
2474	3.2.1.52	Beta-N-acetylhexosaminidase.	NF	NF	NF	NF
2475	>Pyruvate metabolism 00620					
2476	1.1.1.21	Aldehyde reductase.	NF	NF	FOUND	FOUND
2477	1.1.1.27	L-lactate dehydrogenase.	FOUND	FOUND	NF	FOUND
2478	1.1.1.28	D-lactate dehydrogenase.	FOUND	FOUND	FOUND	FOUND
2479	1.1.1.37	Malate dehydrogenase.	NF	NF	NF	NF
2480	1.1.1.38	Malate dehydrogenase (oxaloacetate-decarboxylati	NF	NF	NF	NF
2481	1.1.1.39	Malate dehydrogenase (decarboxylating).	NF	NF	NF	NF
2482	1.1.1.40	Malate dehydrogenase (oxaloacetate-decarboxylati	NF	NF	FOUND	NF
2483	1.1.1.77	Lactaldehyde reductase.	NF	NF	FOUND	NF
2484	1.1.1.78	Methylglyoxal reductase (NADH-dependent).	NF	NF	NF	NF
2485	1.1.1.79	Glyoxylate reductase (NADP(+)).	NF	NF	NF	NF
2486	1.1.1.82	Malate dehydrogenase (NADP(+)).	NF	NF	NF	NF
2487	1.1.2.3	L-lactate dehydrogenase (cytochrome).	NF	NF	NF	NF
2488	1.1.2.4	D-lactate dehydrogenase (cytochrome).	NF	NF	NF	NF
2489	1.1.2.5	D-lactate dehydrogenase (cytochrome c-553).	NF	NF	NF	NF
2490	1.1.3.3	Malate oxidase.	NF	NF	NF	NF
2491	1.1.5.4	Malate dehydrogenase (quinone).	NF	NF	NF	NF
2492	1.1.99.7	Lactate--malate transhydrogenase.	NF	NF	NF	NF
2493	1.13.12.4	Lactate 2-monooxygenase.	NF	NF	NF	NF
2494	1.2.1.10	Acetaldehyde dehydrogenase (acetylating).	NF	NF	NF	NF
2495	1.2.1.22	Lactaldehyde dehydrogenase.	NF	NF	NF	NF
2496	1.2.1.23	2-oxoaldehyde dehydrogenase (NAD(+)).	NF	NF	NF	NF
2497	1.2.1.3	Aldehyde dehydrogenase (NAD(+)).	NF	NF	FOUND	FOUND
2498	1.2.1.49	2-oxoaldehyde dehydrogenase (NADP(+)).	NF	NF	NF	NF
2499	1.2.3.3	Pyruvate oxidase.	NF	NF	NF	NF
2500	1.2.3.6	Pyruvate oxidase (CoA-acetylating).	NF	NF	NF	NF
2501	1.2.4.1	Pyruvate dehydrogenase (acetyl-transferring).	FOUND	FOUND	FOUND	FOUND
2502	1.2.5.1	Pyruvate dehydrogenase (quinone).	NF	NF	NF	NF
2503	1.2.7.1	Pyruvate synthase.	NF	NF	NF	NF
2504	1.2.99.3	Aldehyde dehydrogenase (pyrroloquinoline-quinone	NF	NF	NF	NF
2505	1.2.99.6	Carboxylate reductase.	NF	NF	NF	NF
2506	1.8.1.4	Dihydrolipoyl dehydrogenase.	FOUND	FOUND	FOUND	FOUND
2507	2.3.1.12	Dihydrolipoyllysine-residue acetyltransferase.	FOUND	FOUND	FOUND	FOUND
2508	2.3.1.54	Formate C-acetyltransferase.	FOUND	FOUND	FOUND	FOUND
2509	2.3.1.8	Phosphate acetyltransferase.	FOUND	FOUND	FOUND	FOUND
2510	2.3.1.9	Acetyl-CoA C-acetyltransferase.	FOUND	FOUND	NF	NF
2511	2.3.3.13	2-isopropylmalate synthase.	NF	NF	FOUND	FOUND
2512	2.3.3.14	Homocitrate synthase.	NF	NF	NF	NF
2513	2.3.3.6	2-ethylmalate synthase.	NF	NF	NF	NF
2514	2.3.3.9	Malate synthase.	NF	NF	NF	NF
2515	2.7.1.40	Pyruvate kinase.	FOUND	FOUND	FOUND	FOUND
2516	2.7.2.1	Acetate kinase.	FOUND	FOUND	FOUND	FOUND
2517	2.7.2.12	Acetate kinase (diphosphate).	NF	NF	NF	NF
2518	2.7.9.1	Pyruvate, phosphate dikinase.	NF	NF	NF	NF
2519	2.7.9.2	Pyruvate, water dikinase.	NF	NF	NF	FOUND
2520	2.8.3.1	Propionate CoA-transferase.	NF	NF	NF	NF
2521	3.1.2.1	Acetyl-CoA hydrolase.	NF	NF	NF	NF
2522	3.1.2.6	Hydroxyacylglutathione hydrolase.	NF	FOUND	NF	NF
2523	3.6.1.7	Acylphosphatase.	NF	NF	NF	NF
2524	4.1.1.-	Carboxy-lyases.	FOUND	FOUND	FOUND	FOUND

2525	4.1.1.3	Oxaloacetate decarboxylase.	NF	NF	NF	NF
2526	4.1.1.31	Phosphoenolpyruvate carboxylase.	FOUND	FOUND	FOUND	NF
2527	4.1.1.32	Phosphoenolpyruvate carboxykinase (GTP).	NF	NF	NF	NF
2528	4.1.1.38	Phosphoenolpyruvate carboxykinase (diphosphate).	NF	NF	NF	NF
2529	4.1.1.49	Phosphoenolpyruvate carboxykinase (ATP).	NF	NF	FOUND	FOUND
2530	4.1.1.78	Acetylenedicarboxylate decarboxylase.	NF	NF	NF	NF
2531	4.1.2.36	Lactate aldolase.	NF	NF	NF	NF
2532	4.1.3.-	Oxo-acid-lyases.	FOUND	FOUND	FOUND	FOUND
2533	4.2.3.3	Methylglyoxal synthase.	NF	NF	NF	FOUND
2534	4.4.1.5	Lactoylglutathione lyase.	NF	NF	FOUND	FOUND
2535	5.1.2.1	Lactate racemase.	NF	NF	NF	NF
2536	6.2.1.1	Acetate--CoA ligase.	NF	NF	NF	NF
2537	6.2.1.13	Acetate--CoA ligase (ADP-forming).	NF	NF	NF	NF
2538	6.4.1.1	Pyruvate carboxylase.	NF	NF	NF	NF
2539	6.4.1.2	Acetyl-CoA carboxylase.	NF	NF	FOUND	FOUND
2540	>Dioxin degradation 00621					
2541	1.13.11.39	Biphenyl-2,3-diol 1,2-dioxygenase.	NF	NF	NF	NF
2542	1.14.12.18	Biphenyl 2,3-dioxygenase.	NF	NF	NF	NF
2543	1.14.13.1	Salicylate 1-monooxygenase.	NF	NF	NF	NF
2544	1.2.1.10	Acetaldehyde dehydrogenase (acetylating).	NF	NF	NF	NF
2545	1.3.1.56	Cis-2,3-dihydrobiphenyl-2,3-diol dehydrogenase.	NF	NF	NF	NF
2546	3.7.1.8	2,6-dioxo-6-phenylhexa-3-enoate hydrolase.	NF	NF	NF	NF
2547	4.1.1.-	Carboxy-lyases.	FOUND	FOUND	FOUND	FOUND
2548	4.1.1.77	4-oxalocrotonate decarboxylase.	NF	NF	NF	NF
2549	4.1.3.39	4-hydroxy-2-oxovalerate aldolase.	NF	NF	NF	NF
2550	4.2.1.80	2-oxopent-4-enoate hydratase.	NF	NF	NF	NF
2551	5.3.2.-	Interconverting keto- and enol- groups.	NF	NF	NF	NF
2552	>Xylene degradation 00622					
2553	1.1.1.-	With NAD(+) or NADP(+) as acceptor.	FOUND	FOUND	FOUND	FOUND
2554	1.1.1.90	Aryl-alcohol dehydrogenase.	NF	NF	NF	NF
2555	1.13.11.-	With incorporation of two atoms of oxygen.	NF	NF	NF	NF
2556	1.13.11.2	Catechol 2,3-dioxygenase.	NF	NF	NF	NF
2557	1.14.12.-	With NADH or NADPH as one donor, and incorporat	NF	NF	NF	NF
2558	1.14.13.-	With NADH or NADPH as one donor, and incorporat	NF	NF	NF	NF
2559	1.2.1.10	Acetaldehyde dehydrogenase (acetylating).	NF	NF	NF	NF
2560	1.2.1.28	Benzaldehyde dehydrogenase (NAD(+)).	NF	NF	NF	NF
2561	1.2.1.29	Aryl-aldehyde dehydrogenase.	NF	NF	NF	NF
2562	1.2.1.32	Aminomuconate-semialdehyde dehydrogenase.	NF	NF	NF	NF
2563	1.2.1.7	Benzaldehyde dehydrogenase (NADP(+)).	NF	NF	NF	NF
2564	1.3.1.-	With NAD(+) or NADP(+) as acceptor.	FOUND	FOUND	FOUND	FOUND
2565	1.3.1.58	2,3-dihydroxy-2,3-dihydro-p-cumate dehydrogenase	NF	NF	NF	NF
2566	1.3.1.67	Cis-1,2-dihydroxy-4-methylcyclohexa-3,5-diene-1-ca	NF	NF	NF	NF
2567	1.3.1.68	1,2-dihydroxy-6-methylcyclohexa-3,5-dienecarboxy	NF	NF	NF	NF
2568	3.7.1.-	In ketonic substances.	NF	NF	NF	FOUND
2569	3.7.1.9	2-hydroxymuconate-semialdehyde hydrolase.	NF	NF	NF	NF
2570	4.1.1.-	Carboxy-lyases.	FOUND	FOUND	FOUND	FOUND
2571	4.1.1.77	4-oxalocrotonate decarboxylase.	NF	NF	NF	NF
2572	4.1.3.39	4-hydroxy-2-oxovalerate aldolase.	NF	NF	NF	NF
2573	4.2.1.80	2-oxopent-4-enoate hydratase.	NF	NF	NF	NF
2574	>Toluene degradation 00623					
2575	1.1.1.257	4-(hydroxymethyl)benzenesulfonate dehydrogenase	NF	NF	NF	NF
2576	1.1.1.35	3-hydroxyacyl-CoA dehydrogenase.	NF	NF	NF	NF
2577	1.1.1.90	Aryl-alcohol dehydrogenase.	NF	NF	NF	NF
2578	1.1.1.97	3-hydroxybenzyl-alcohol dehydrogenase.	NF	NF	NF	NF
2579	1.13.11.-	With incorporation of two atoms of oxygen.	NF	NF	NF	NF

2580	1.13.11.1	Catechol 1,2-dioxygenase.	NF	NF	NF	NF
2581	1.14.12.-	With NADH or NADPH as one donor, and incorporat	NF	NF	NF	NF
2582	1.14.12.11	Toluene dioxygenase.	NF	NF	NF	NF
2583	1.14.12.8	4-sulfobenzoate 3,4-dioxygenase.	NF	NF	NF	NF
2584	1.14.13.-	With NADH or NADPH as one donor, and incorporat	NF	NF	NF	NF
2585	1.14.13.7	Phenol 2-monooxygenase.	NF	NF	NF	NF
2586	1.17.99.1	4-cresol dehydrogenase (hydroxylating).	NF	NF	NF	NF
2587	1.2.1.28	Benzaldehyde dehydrogenase (NAD(+)).	NF	NF	NF	NF
2588	1.2.1.62	4-formylbenzenesulfonate dehydrogenase.	NF	NF	NF	NF
2589	1.2.1.64	4-hydroxybenzaldehyde dehydrogenase.	NF	NF	NF	NF
2590	1.2.1.7	Benzaldehyde dehydrogenase (NADP(+)).	NF	NF	NF	NF
2591	1.3.1.-	With NAD(+) or NADP(+) as acceptor.	FOUND	FOUND	FOUND	FOUND
2592	1.3.1.19	Cis-1,2-dihydrobenzene-1,2-diol dehydrogenase.	NF	NF	NF	NF
2593	1.3.1.32	Maleylacetate reductase.	NF	NF	NF	NF
2594	1.3.99.1	Succinate dehydrogenase.	NF	NF	NF	NF
2595	1.3.99.21	(R)-benzylsuccinyl-CoA dehydrogenase.	NF	NF	NF	NF
2596	2.3.1.-	Transferring groups other than amino-acyl groups.	FOUND	FOUND	FOUND	FOUND
2597	2.8.3.15	Succinyl-CoA:(R)-benzylsuccinate CoA-transferase.	NF	NF	NF	NF
2598	3.1.1.-	Carboxylic ester hydrolases.	FOUND	FOUND	FOUND	FOUND
2599	3.1.1.45	Carboxymethylenebutenolidase.	NF	NF	NF	NF
2600	4.1.99.11	Benzylsuccinate synthase.	NF	NF	NF	NF
2601	4.2.1.-	Hydro-lyases.	FOUND	FOUND	FOUND	FOUND
2602	5.4.99.14	4-carboxymethyl-4-methylbutenolide mutase.	NF	NF	NF	NF
2603	5.5.1.-	Intramolecular lyases.	NF	NF	NF	NF
2604	5.5.1.1	Muconate cycloisomerase.	NF	NF	NF	NF
2605	5.5.1.7	Chloromuconate cycloisomerase.	NF	NF	NF	NF
2606	>Polycyclic aromatic hydrocarbon degradation 00624					
2607	1.1.-.-	Acting on the CH-OH group of donors.	FOUND	FOUND	FOUND	FOUND
2608	1.1.1.256	Fluoren-9-ol dehydrogenase.	NF	NF	NF	NF
2609	1.13.11.-	With incorporation of two atoms of oxygen.	NF	NF	NF	NF
2610	1.13.11.3	Protocatechuate 3,4-dioxygenase.	NF	NF	NF	NF
2611	1.13.11.38	1-hydroxy-2-naphthoate 1,2-dioxygenase.	NF	NF	NF	NF
2612	1.13.11.8	Protocatechuate 4,5-dioxygenase.	NF	NF	NF	NF
2613	1.14.-.-	Acting on paired donors, with incorporation or redu	NF	NF	NF	NF
2614	1.14.12.-	With NADH or NADPH as one donor, and incorporat	NF	NF	NF	NF
2615	1.14.12.12	Naphthalene 1,2-dioxygenase.	NF	NF	NF	NF
2616	1.14.12.15	Terephthalate 1,2-dioxygenase.	NF	NF	NF	NF
2617	1.14.12.7	Phthalate 4,5-dioxygenase.	NF	NF	NF	NF
2618	1.14.13.-	With NADH or NADPH as one donor, and incorporat	NF	NF	NF	NF
2619	1.14.13.1	Salicylate 1-monooxygenase.	NF	NF	NF	NF
2620	1.14.13.23	3-hydroxybenzoate 4-monooxygenase.	NF	NF	NF	NF
2621	1.2.1.-	With NAD(+) or NADP(+) as acceptor.	FOUND	FOUND	FOUND	FOUND
2622	1.3.-.-	Acting on the CH-CH group of donors.	FOUND	FOUND	FOUND	FOUND
2623	1.3.1.-	With NAD(+) or NADP(+) as acceptor.	FOUND	FOUND	FOUND	FOUND
2624	1.3.1.19	Cis-1,2-dihydrobenzene-1,2-diol dehydrogenase.	NF	NF	NF	NF
2625	1.3.1.29	Cis-1,2-dihydro-1,2-dihydroxynaphthalene dehydro	NF	NF	NF	NF
2626	1.3.1.49	Cis-3,4-dihydrophenanthrene-3,4-diol dehydrogena	NF	NF	NF	NF
2627	1.3.1.53	(3S,4R)-3,4-dihydroxycyclohexa-1,5-diene-1,4-dicar	NF	NF	NF	NF
2628	1.3.1.64	Phthalate 4,5-cis-dihydrodiol dehydrogenase.	NF	NF	NF	NF
2629	1.6.-.-	Acting on NADH or NADPH.	FOUND	NF	FOUND	FOUND
2630	1.6.5.-	With a quinone or similar compound as acceptor.	NF	NF	NF	FOUND
2631	2.1.1.-	Methyltransferases.	FOUND	FOUND	FOUND	FOUND
2632	2.8.2.-	Sulfotransferases.	NF	NF	NF	NF
2633	3.1.1.35	Dihydrocoumarin hydrolase.	NF	NF	NF	NF
2634	3.3.2.-	Ether hydrolases.	NF	NF	NF	FOUND

2635	3.7.1.-	In ketonic substances.	NF	NF	NF	FOUND
2636	4.1.1.-	Carboxy-lyases.	FOUND	FOUND	FOUND	FOUND
2637	4.1.1.55	4,5-dihydroxyphthalate decarboxylase.	NF	NF	NF	NF
2638	4.1.1.69	3,4-dihydroxyphthalate decarboxylase.	NF	NF	NF	NF
2639	4.1.2.-	Aldehyde-lyases.	FOUND	FOUND	FOUND	FOUND
2640	4.1.2.34	4-(2-carboxyphenyl)-2-oxobut-3-enoate aldolase.	NF	NF	NF	NF
2641	4.1.3.-	Oxo-acid-lyases.	FOUND	FOUND	FOUND	FOUND
2642	5.1.2.-	Acting on hydroxy acids and derivatives.	NF	NF	NF	NF
2643	>Chloroalkane and chloroalkene degradation 00625					
2644	1.1.-.-	Acting on the CH-OH group of donors.	FOUND	FOUND	FOUND	FOUND
2645	1.1.1.-	With NAD(+) or NADP(+) as acceptor.	FOUND	FOUND	FOUND	FOUND
2646	1.1.1.1	Alcohol dehydrogenase.	FOUND	FOUND	FOUND	FOUND
2647	1.1.2.7	Methanol dehydrogenase (cytochrome c).	NF	NF	NF	NF
2648	1.1.2.8	Alcohol dehydrogenase (cytochrome c).	NF	NF	NF	NF
2649	1.13.12.-	With incorporation of one atom of oxygen.	NF	NF	FOUND	NF
2650	1.14.-.-	Acting on paired donors, with incorporation or redu	NF	NF	NF	NF
2651	1.14.12.11	Toluene dioxygenase.	NF	NF	NF	NF
2652	1.14.13.-	With NADH or NADPH as one donor, and incorporat	NF	NF	NF	NF
2653	1.14.13.69	Alkene monooxygenase.	NF	NF	NF	NF
2654	1.18.6.1	Nitrogenase.	NF	NF	NF	NF
2655	1.2.1.3	Aldehyde dehydrogenase (NAD(+)).	NF	NF	FOUND	FOUND
2656	1.2.1.46	Formaldehyde dehydrogenase.	NF	NF	NF	NF
2657	1.97.1.-	Sole sub-subclass for oxidoreductases that do not b	NF	NF	FOUND	NF
2658	1.97.1.8	Tetrachloroethene reductive dehalogenase.	NF	NF	NF	NF
2659	3.3.2.10	Soluble epoxide hydrolase.	NF	NF	NF	NF
2660	3.8.1.-	In C-halide compounds.	NF	NF	NF	NF
2661	3.8.1.2	(S)-2-haloacid dehalogenase.	NF	NF	NF	NF
2662	3.8.1.3	Haloacetate dehalogenase.	NF	NF	NF	NF
2663	3.8.1.5	Haloalkane dehalogenase.	NF	NF	NF	NF
2664	4.1.1.-	Carboxy-lyases.	FOUND	FOUND	FOUND	FOUND
2665	4.2.1.112	Acetylene hydratase.	NF	NF	NF	NF
2666	4.5.1.3	Dichloromethane dehalogenase.	NF	NF	NF	NF
2667	>Naphthalene degradation 00626					
2668	1.1.-.-	Acting on the CH-OH group of donors.	FOUND	FOUND	FOUND	FOUND
2669	1.1.1.1	Alcohol dehydrogenase.	FOUND	FOUND	FOUND	FOUND
2670	1.13.-.-	Acting on single donors with incorporation of molec	NF	NF	FOUND	FOUND
2671	1.13.11.-	With incorporation of two atoms of oxygen.	NF	NF	NF	NF
2672	1.13.11.56	1,2-dihydroxynaphthalene dioxygenase.	NF	NF	NF	NF
2673	1.14.12.-	With NADH or NADPH as one donor, and incorporat	NF	NF	NF	NF
2674	1.14.12.12	Naphthalene 1,2-dioxygenase.	NF	NF	NF	NF
2675	1.14.13.-	With NADH or NADPH as one donor, and incorporat	NF	NF	NF	NF
2676	1.14.13.1	Salicylate 1-monooxygenase.	NF	NF	NF	NF
2677	1.14.99.-	Miscellaneous (requires further characterization).	NF	NF	NF	NF
2678	1.2.1.-	With NAD(+) or NADP(+) as acceptor.	FOUND	FOUND	FOUND	FOUND
2679	1.2.1.65	Salicylaldehyde dehydrogenase.	NF	NF	NF	NF
2680	1.2.3.-	With oxygen as acceptor.	NF	NF	NF	NF
2681	1.3.1.29	Cis-1,2-dihydro-1,2-dihydroxynaphthalene dehydro	NF	NF	NF	NF
2682	1.3.99.-	With other acceptors.	NF	NF	FOUND	FOUND
2683	2.3.1.-	Transferring groups other than amino-acyl groups.	FOUND	FOUND	FOUND	FOUND
2684	2.8.3.-	CoA-transferases.	NF	NF	NF	NF
2685	4.1.1.-	Carboxy-lyases.	FOUND	FOUND	FOUND	FOUND
2686	4.1.2.45	Trans-o-hydroxybenzylidenepyruvate hydratase-ald	NF	NF	NF	NF
2687	4.1.99.-	Other carbon-carbon lyases.	NF	NF	NF	FOUND
2688	4.2.1.-	Hydro-lyases.	FOUND	FOUND	FOUND	FOUND
2689	5.3.99.-	Other intramolecular oxidoreductases.	NF	NF	NF	NF

2690	5.99.1.4	2-hydroxychromene-2-carboxylate isomerase.	NF	NF	NF	NF
2691	>Aminobenzoate degradation 00627					
2692	1.1.-.-	Acting on the CH-OH group of donors.	FOUND	FOUND	FOUND	FOUND
2693	1.1.3.19	4-hydroxymandelate oxidase.	NF	NF	NF	NF
2694	1.1.3.38	Vanillyl-alcohol oxidase.	NF	NF	NF	NF
2695	1.1.99.31	(S)-mandelate dehydrogenase.	NF	NF	NF	NF
2696	1.13.11.-	With incorporation of two atoms of oxygen.	NF	NF	NF	NF
2697	1.13.11.3	Protocatechuate 3,4-dioxygenase.	NF	NF	NF	NF
2698	1.13.11.8	Protocatechuate 4,5-dioxygenase.	NF	NF	NF	NF
2699	1.14.-.-	Acting on paired donors, with incorporation or redu	NF	NF	NF	NF
2700	1.14.12.-	With NADH or NADPH as one donor, and incorporat	NF	NF	NF	NF
2701	1.14.12.1	Anthranilate 1,2-dioxygenase (deaminating, decarb	NF	NF	NF	NF
2702	1.14.13.-	With NADH or NADPH as one donor, and incorporat	NF	NF	NF	NF
2703	1.14.13.12	Benzoate 4-monooxygenase.	NF	NF	NF	NF
2704	1.14.13.27	4-aminobenzoate 1-monooxygenase.	NF	NF	NF	NF
2705	1.14.13.29	4-nitrophenol 2-monooxygenase.	NF	NF	NF	NF
2706	1.14.13.35	Anthranilate 3-monooxygenase (deaminating).	NF	NF	NF	NF
2707	1.14.13.40	Anthraniloyl-CoA monooxygenase.	NF	NF	NF	NF
2708	1.14.13.7	Phenol 2-monooxygenase.	NF	NF	NF	NF
2709	1.14.13.82	Vanillate monooxygenase.	NF	NF	NF	NF
2710	1.14.14.1	Unspecific monooxygenase.	NF	NF	NF	NF
2711	1.14.16.6	Mandelate 4-monooxygenase.	NF	NF	NF	NF
2712	1.2.1.28	Benzaldehyde dehydrogenase (NAD(+)).	NF	NF	NF	NF
2713	1.2.1.64	4-hydroxybenzaldehyde dehydrogenase.	NF	NF	NF	NF
2714	1.2.1.67	Vanillin dehydrogenase.	NF	NF	NF	NF
2715	1.2.1.7	Benzaldehyde dehydrogenase (NADP(+)).	NF	NF	NF	NF
2716	1.2.3.-	With oxygen as acceptor.	NF	NF	NF	NF
2717	1.3.1.57	Phloroglucinol reductase.	NF	NF	NF	NF
2718	1.3.99.20	4-hydroxybenzoyl-CoA reductase.	NF	NF	NF	NF
2719	1.4.-.-	Acting on the CH-NH(2) group of donors.	NF	FOUND	FOUND	FOUND
2720	1.6.5.6	p-benzoquinone reductase (NADPH).	NF	NF	NF	NF
2721	1.6.5.7	2-hydroxy-1,4-benzoquinone reductase.	NF	NF	NF	NF
2722	1.7.1.-	With NAD(+) or NADP(+) as acceptor.	NF	NF	NF	NF
2723	1.97.1.2	Pyrogallol hydroxytransferase.	NF	NF	NF	NF
2724	2.3.1.-	Transferring groups other than amino-acyl groups.	FOUND	FOUND	FOUND	FOUND
2725	2.7.9.-	Phosphotransferases with paired acceptors.	NF	NF	NF	FOUND
2726	2.8.3.8	Acetate CoA-transferase.	NF	NF	NF	NF
2727	3.1.1.-	Carboxylic ester hydrolases.	FOUND	FOUND	FOUND	FOUND
2728	3.1.1.57	2-pyrone-4,6-dicarboxylate lactonase.	NF	NF	NF	NF
2729	3.1.3.1	Alkaline phosphatase.	FOUND	FOUND	FOUND	FOUND
2730	3.1.3.2	Acid phosphatase.	FOUND	FOUND	FOUND	FOUND
2731	3.1.3.41	4-nitrophenylphosphatase.	NF	NF	NF	NF
2732	3.1.8.1	Aryldialkylphosphatase.	NF	NF	NF	FOUND
2733	3.5.1.4	Amidase.	NF	FOUND	NF	NF
2734	3.5.5.1	Nitrilase.	NF	NF	NF	NF
2735	3.6.1.7	Acylphosphatase.	NF	NF	NF	NF
2736	3.7.1.-	In ketonic substances.	NF	NF	NF	FOUND
2737	4.1.1.46	o-pyrocatechuate decarboxylase.	NF	NF	NF	NF
2738	4.1.1.59	Gallate decarboxylase.	NF	NF	NF	NF
2739	4.1.1.61	4-hydroxybenzoate decarboxylase.	NF	NF	NF	NF
2740	4.1.1.63	Protocatechuate decarboxylase.	NF	NF	NF	NF
2741	4.1.1.7	Benzoylformate decarboxylase.	NF	NF	NF	NF
2742	4.2.1.17	Enoyl-CoA hydratase.	NF	NF	NF	NF
2743	4.2.1.84	Nitrile hydratase.	NF	NF	NF	NF
2744	5.1.2.2	Mandelate racemase.	NF	NF	NF	NF

2745	5.4.4.1	(Hydroxyamino)benzene mutase.	NF	NF	NF	NF
2746	5.5.1.-	Intramolecular lyases.	NF	NF	NF	NF
2747	6.2.1.-	Acid--thiol ligases.	NF	NF	NF	NF
2748	6.2.1.25	Benzoate--CoA ligase.	NF	NF	NF	NF
2749	6.2.1.27	4-hydroxybenzoate--CoA ligase.	NF	NF	NF	NF
2750	6.2.1.32	Anthranilate--CoA ligase.	NF	NF	NF	NF
2751	6.4.1.-	Ligases that form carbon-carbon bonds.	NF	NF	FOUND	FOUND
2752	>Glyoxylate and dicarboxylate metabolism 00630					
2753	1.1.1.26	Glyoxylate reductase.	NF	NF	NF	NF
2754	1.1.1.29	Glycerate dehydrogenase.	NF	NF	NF	NF
2755	1.1.1.36	Acetoacetyl-CoA reductase.	NF	NF	NF	NF
2756	1.1.1.37	Malate dehydrogenase.	NF	NF	NF	NF
2757	1.1.1.60	2-hydroxy-3-oxopropionate reductase.	NF	NF	NF	NF
2758	1.1.1.77	Lactaldehyde reductase.	NF	NF	FOUND	NF
2759	1.1.1.79	Glyoxylate reductase (NADP(+)).	NF	NF	NF	NF
2760	1.1.1.81	Hydroxypyruvate reductase.	NF	NF	NF	NF
2761	1.1.1.92	Oxaloglycolate reductase (decarboxylating).	NF	NF	NF	NF
2762	1.1.1.93	Tartrate dehydrogenase.	NF	NF	NF	NF
2763	1.1.3.15	(S)-2-hydroxy-acid oxidase.	NF	NF	NF	FOUND
2764	1.1.99.14	Glycolate dehydrogenase.	NF	NF	NF	NF
2765	1.2.1.17	Glyoxylate dehydrogenase (acylating).	NF	NF	NF	NF
2766	1.2.1.2	Formate dehydrogenase.	NF	NF	NF	FOUND
2767	1.2.1.21	Glycolaldehyde dehydrogenase.	NF	NF	NF	NF
2768	1.2.2.1	Formate dehydrogenase (cytochrome).	NF	NF	NF	NF
2769	1.2.3.4	Oxalate oxidase.	NF	NF	NF	NF
2770	1.2.3.5	Glyoxylate oxidase.	NF	NF	NF	NF
2771	1.3.1.7	Meso-tartrate dehydrogenase.	NF	NF	NF	NF
2772	2.2.1.5	2-hydroxy-3-oxoadipate synthase.	NF	NF	NF	NF
2773	2.3.1.9	Acetyl-CoA C-acetyltransferase.	FOUND	FOUND	NF	NF
2774	2.3.3.1	Citrate (Si)-synthase.	NF	NF	FOUND	FOUND
2775	2.3.3.12	3-propylmalate synthase.	NF	NF	NF	NF
2776	2.3.3.7	3-ethylmalate synthase.	NF	NF	NF	NF
2777	2.3.3.9	Malate synthase.	NF	NF	NF	NF
2778	2.7.1.31	Glycerate kinase.	FOUND	NF	NF	FOUND
2779	2.7.2.6	Formate kinase.	NF	NF	NF	NF
2780	2.8.3.2	Oxalate CoA-transferase.	NF	NF	NF	NF
2781	3.1.2.10	Formyl-CoA hydrolase.	NF	NF	NF	NF
2782	3.1.3.18	Phosphoglycolate phosphatase.	FOUND	FOUND	FOUND	FOUND
2783	3.3.2.4	Trans-epoxysuccinate hydrolase.	NF	NF	NF	NF
2784	3.5.1.10	Formyltetrahydrofolate deformylase.	NF	NF	NF	NF
2785	3.5.1.27	N-formylmethionylaminoacyl-tRNA deformylase.	NF	NF	NF	NF
2786	3.5.1.31	Formylmethionine deformylase.	NF	NF	NF	NF
2787	3.5.1.49	Formamidase.	NF	NF	NF	NF
2788	3.5.1.56	N,N-dimethylformamidase.	NF	NF	NF	NF
2789	3.5.1.68	N-formylglutamate deformylase.	NF	NF	NF	NF
2790	3.5.1.8	Formylaspartate deformylase.	NF	NF	NF	NF
2791	3.5.1.9	Arylformamidase.	NF	NF	NF	NF
2792	4.1.1.2	Oxalate decarboxylase.	NF	NF	NF	NF
2793	4.1.1.39	Ribulose-bisphosphate carboxylase.	NF	NF	NF	NF
2794	4.1.1.40	Hydroxypyruvate decarboxylase.	NF	NF	NF	NF
2795	4.1.1.47	Tartronate-semialdehyde synthase.	NF	NF	NF	NF
2796	4.1.1.54	Dihydroxyfumarate decarboxylase.	NF	NF	NF	NF
2797	4.1.1.8	Oxalyl-CoA decarboxylase.	NF	NF	NF	NF
2798	4.1.3.1	Isocitrate lyase.	NF	NF	NF	NF
2799	4.1.3.13	Oxalomalate lyase.	NF	NF	NF	NF

2800	4.1.3.16	4-hydroxy-2-oxoglutarate aldolase.	FOUND	NF	NF	NF
2801	4.1.3.24	Malyl-CoA lyase.	NF	NF	NF	NF
2802	4.2.1.3	Aconitate hydratase.	NF	NF	FOUND	NF
2803	4.2.1.32	L(+)-tartrate dehydratase.	NF	NF	NF	NF
2804	4.2.1.55	3-hydroxybutyryl-CoA dehydratase.	NF	NF	NF	NF
2805	5.1.2.5	Tartrate epimerase.	NF	NF	NF	NF
2806	5.1.99.1	Methylmalonyl-CoA epimerase.	NF	NF	NF	NF
2807	5.3.1.22	Hydroxypyruvate isomerase.	NF	NF	NF	NF
2808	5.4.99.2	Methylmalonyl-CoA mutase.	NF	NF	NF	NF
2809	6.2.1.8	Oxalate--CoA ligase.	NF	NF	NF	NF
2810	6.2.1.9	Malate--CoA ligase.	NF	NF	NF	NF
2811	6.4.1.3	Propionyl-CoA carboxylase.	NF	NF	NF	NF
2812	>Nitrotoluene degradation 00633					
2813	1.-.-.-	Oxidoreductases.	FOUND	FOUND	FOUND	FOUND
2814	1.1.1.-	With NAD(+) or NADP(+) as acceptor.	FOUND	FOUND	FOUND	FOUND
2815	1.12.99.6	Hydrogenase (acceptor).	NF	NF	NF	NF
2816	1.13.11.-	With incorporation of two atoms of oxygen.	NF	NF	NF	NF
2817	1.13.12.-	With incorporation of one atom of oxygen.	NF	NF	FOUND	NF
2818	1.14.12.-	With NADH or NADPH as one donor, and incorporat	NF	NF	NF	NF
2819	1.2.7.1	Pyruvate synthase.	NF	NF	NF	NF
2820	1.2.99.2	Carbon-monoxide dehydrogenase (acceptor).	NF	NF	NF	NF
2821	1.7.1.-	With NAD(+) or NADP(+) as acceptor.	NF	NF	NF	NF
2822	1.8.99.3	Hydrogensulfite reductase.	NF	NF	NF	NF
2823	2.3.1.5	Arylamine N-acetyltransferase.	NF	NF	NF	NF
2824	3.7.1.-	In ketonic substances.	NF	NF	NF	FOUND
2825	>Propanoate metabolism 00640					
2826	1.1.1.27	L-lactate dehydrogenase.	FOUND	FOUND	NF	FOUND
2827	1.1.1.59	3-hydroxypropionate dehydrogenase.	NF	NF	NF	NF
2828	1.1.1.80	Isopropanol dehydrogenase (NADP(+)).	NF	NF	NF	NF
2829	1.1.2.8	Alcohol dehydrogenase (cytochrome c).	NF	NF	NF	NF
2830	1.2.1.18	Malonate-semialdehyde dehydrogenase (acetylatin	NF	NF	NF	NF
2831	1.2.1.27	Methylmalonate-semialdehyde dehydrogenase (acy	NF	NF	NF	FOUND
2832	1.2.1.3	Aldehyde dehydrogenase (NAD(+)).	NF	NF	FOUND	FOUND
2833	1.2.7.1	Pyruvate synthase.	NF	NF	NF	NF
2834	1.2.7.2	2-oxobutyrate synthase.	NF	NF	NF	NF
2835	1.2.99.3	Aldehyde dehydrogenase (pyrroloquinoline-quinon	NF	NF	NF	NF
2836	1.3.1.-	With NAD(+) or NADP(+) as acceptor.	FOUND	FOUND	FOUND	FOUND
2837	1.3.99.3	Acyl-CoA dehydrogenase.	NF	NF	FOUND	FOUND
2838	2.1.3.1	Methylmalonyl-CoA carboxytransferase.	NF	NF	NF	NF
2839	2.3.1.54	Formate C-acetyltransferase.	FOUND	FOUND	FOUND	FOUND
2840	2.3.1.8	Phosphate acetyltransferase.	FOUND	FOUND	FOUND	FOUND
2841	2.3.1.9	Acetyl-CoA C-acetyltransferase.	FOUND	FOUND	NF	NF
2842	2.3.3.5	2-methylcitrate synthase.	NF	NF	NF	NF
2843	2.6.1.18	Beta-alanine--pyruvate transaminase.	NF	NF	NF	NF
2844	2.6.1.19	4-aminobutyrate transaminase.	NF	NF	NF	FOUND
2845	2.7.2.1	Acetate kinase.	FOUND	FOUND	FOUND	FOUND
2846	2.7.2.15	Propionate kinase.	NF	NF	NF	NF
2847	2.8.3.1	Propionate CoA-transferase.	NF	NF	NF	NF
2848	2.8.3.3	Malonate CoA-transferase.	NF	NF	NF	NF
2849	2.8.3.8	Acetate CoA-transferase.	NF	NF	NF	NF
2850	3.1.2.17	(S)-methylmalonyl-CoA hydrolase.	NF	NF	NF	NF
2851	3.1.2.4	3-hydroxyisobutyryl-CoA hydrolase.	NF	NF	NF	NF
2852	3.5.99.7	1-aminocyclopropane-1-carboxylate deaminase.	NF	NF	NF	NF
2853	4.1.1.4	Acetoacetate decarboxylase.	NF	NF	NF	NF
2854	4.1.1.41	Methylmalonyl-CoA decarboxylase.	NF	NF	NF	NF

2855	4.1.1.9	Malonyl-CoA decarboxylase.	NF	NF	NF	NF
2856	4.1.3.30	Methylisocitrate lyase.	NF	NF	NF	NF
2857	4.2.1.17	Enoyl-CoA hydratase.	NF	NF	NF	NF
2858	4.2.1.27	Acetylenecarboxylate hydratase.	NF	NF	NF	NF
2859	4.2.1.54	Lactoyl-CoA dehydratase.	NF	NF	NF	NF
2860	4.2.1.79	2-methylcitrate dehydratase.	NF	NF	NF	NF
2861	4.2.1.99	2-methylisocitrate dehydratase.	NF	NF	NF	NF
2862	4.3.1.6	Beta-alanyl-CoA ammonia-lyase.	NF	NF	NF	NF
2863	5.1.99.1	Methylmalonyl-CoA epimerase.	NF	NF	NF	NF
2864	5.4.99.2	Methylmalonyl-CoA mutase.	NF	NF	NF	NF
2865	6.2.1.-	Acid--thiol ligases.	NF	NF	NF	NF
2866	6.2.1.1	Acetate--CoA ligase.	NF	NF	NF	NF
2867	6.2.1.13	Acetate--CoA ligase (ADP-forming).	NF	NF	NF	NF
2868	6.2.1.17	Propionate--CoA ligase.	NF	NF	NF	NF
2869	6.2.1.4	Succinate--CoA ligase (GDP-forming).	NF	NF	NF	NF
2870	6.2.1.5	Succinate--CoA ligase (ADP-forming).	NF	NF	NF	NF
2871	6.4.1.2	Acetyl-CoA carboxylase.	NF	NF	FOUND	FOUND
2872	6.4.1.3	Propionyl-CoA carboxylase.	NF	NF	NF	NF
2873	>Ethylbenzene degradation 00642					
2874	1.1.-.-	Acting on the CH-OH group of donors.	FOUND	FOUND	FOUND	FOUND
2875	1.14.12.12	Naphthalene 1,2-dioxygenase.	NF	NF	NF	NF
2876	1.17.99.2	Ethylbenzene hydroxylase.	NF	NF	NF	NF
2877	1.3.1.66	Cis-dihydroethylcatechol dehydrogenase.	NF	NF	NF	NF
2878	2.3.1.-	Transferring groups other than amino-acyl groups.	FOUND	FOUND	FOUND	FOUND
2879	2.3.1.16	Acetyl-CoA C-acyltransferase.	NF	NF	NF	NF
2880	3.7.1.-	In ketonic substances.	NF	NF	NF	FOUND
2881	6.2.1.-	Acid--thiol ligases.	NF	NF	NF	NF
2882	6.4.1.-	Ligases that form carbon-carbon bonds.	NF	NF	FOUND	FOUND
2883	>Styrene degradation 00643					
2884	1.13.11.2	Catechol 2,3-dioxygenase.	NF	NF	NF	NF
2885	1.13.11.5	Homogentisate 1,2-dioxygenase.	NF	NF	NF	NF
2886	1.14.12.-	With NADH or NADPH as one donor, and incorporat	NF	NF	NF	NF
2887	1.14.13.-	With NADH or NADPH as one donor, and incorporat	NF	NF	NF	NF
2888	1.14.13.63	3-hydroxyphenylacetate 6-hydroxylase.	NF	NF	NF	NF
2889	1.2.1.39	Phenylacetaldehyde dehydrogenase.	NF	NF	NF	NF
2890	1.3.1.19	Cis-1,2-dihydrobenzene-1,2-diol dehydrogenase.	NF	NF	NF	NF
2891	2.8.3.1	Propionate CoA-transferase.	NF	NF	NF	NF
2892	2.8.3.12	Glutaconate CoA-transferase.	NF	NF	NF	NF
2893	3.5.1.4	Amidase.	NF	FOUND	NF	NF
2894	3.5.5.1	Nitrilase.	NF	NF	NF	NF
2895	3.5.5.7	Aliphatic nitrilase.	NF	NF	NF	NF
2896	3.7.1.2	Fumarylacetoacetase.	NF	NF	NF	FOUND
2897	3.7.1.9	2-hydroxymuconate-semialdehyde hydrolase.	NF	NF	NF	NF
2898	4.2.1.54	Lactoyl-CoA dehydratase.	NF	NF	NF	NF
2899	4.2.1.84	Nitrile hydratase.	NF	NF	NF	NF
2900	4.99.1.7	Phenylacetaldoxime dehydratase.	NF	NF	NF	NF
2901	5.2.1.2	Maleylacetoacetate isomerase.	NF	NF	NF	NF
2902	5.3.99.7	Styrene-oxide isomerase.	NF	NF	NF	NF
2903	>Butanoate metabolism 00650					
2904	1.1.1.-	With NAD(+) or NADP(+) as acceptor.	FOUND	FOUND	FOUND	FOUND
2905	1.1.1.157	3-hydroxybutyryl-CoA dehydrogenase.	NF	NF	NF	NF
2906	1.1.1.30	3-hydroxybutyrate dehydrogenase.	NF	NF	NF	FOUND
2907	1.1.1.303	Diacetyl reductase ((R)-acetoin forming).	NF	NF	NF	NF
2908	1.1.1.304	Diacetyl reductase ((S)-acetoin forming).	NF	NF	NF	NF
2909	1.1.1.35	3-hydroxyacyl-CoA dehydrogenase.	NF	NF	NF	NF

2910	1.1.1.36	Acetoacetyl-CoA reductase.	NF	NF	NF	NF
2911	1.1.1.4	(R,R)-butanediol dehydrogenase.	NF	NF	FOUND	NF
2912	1.1.1.61	4-hydroxybutyrate dehydrogenase.	NF	NF	NF	NF
2913	1.1.1.76	(S,S)-butanediol dehydrogenase.	NF	NF	NF	NF
2914	1.1.1.83	D-malate dehydrogenase (decarboxylating).	NF	NF	NF	NF
2915	1.1.99.2	2-hydroxyglutarate dehydrogenase.	NF	NF	NF	NF
2916	1.2.1.10	Acetaldehyde dehydrogenase (acetylating).	NF	NF	NF	NF
2917	1.2.1.16	Succinate-semialdehyde dehydrogenase (NAD(P)(+)	NF	NF	NF	NF
2918	1.2.1.24	Succinate-semialdehyde dehydrogenase (NAD(+)).	NF	NF	NF	NF
2919	1.2.1.57	Butanal dehydrogenase.	NF	NF	NF	NF
2920	1.2.4.1	Pyruvate dehydrogenase (acetyl-transferring).	FOUND	FOUND	FOUND	FOUND
2921	1.2.7.1	Pyruvate synthase.	NF	NF	NF	NF
2922	1.3.1.44	Trans-2-enoyl-CoA reductase (NAD(+)).	NF	NF	NF	NF
2923	1.3.99.1	Succinate dehydrogenase.	NF	NF	NF	NF
2924	1.3.99.2	Butyryl-CoA dehydrogenase.	NF	NF	NF	NF
2925	2.2.1.6	Acetolactate synthase.	NF	NF	FOUND	FOUND
2926	2.3.1.-	Transferring groups other than amino-acyl groups.	FOUND	FOUND	FOUND	FOUND
2927	2.3.1.19	Phosphate butyryltransferase.	NF	NF	NF	NF
2928	2.3.1.54	Formate C-acetyltransferase.	FOUND	FOUND	FOUND	FOUND
2929	2.3.1.9	Acetyl-CoA C-acetyltransferase.	FOUND	FOUND	NF	NF
2930	2.3.3.10	Hydroxymethylglutaryl-CoA synthase.	FOUND	FOUND	FOUND	NF
2931	2.6.1.19	4-aminobutyrate transaminase.	NF	NF	NF	FOUND
2932	2.7.2.7	Butyrate kinase.	NF	NF	NF	NF
2933	2.8.3.12	Glutaconate CoA-transferase.	NF	NF	NF	NF
2934	2.8.3.5	3-oxoacid CoA-transferase.	NF	NF	NF	NF
2935	2.8.3.8	Acetate CoA-transferase.	NF	NF	NF	NF
2936	3.1.1.22	Hydroxybutyrate-dimer hydrolase.	NF	NF	NF	NF
2937	3.1.1.75	Poly(3-hydroxybutyrate) depolymerase.	NF	NF	NF	NF
2938	3.1.2.11	Acetoacetyl-CoA hydrolase.	NF	NF	NF	NF
2939	4.1.1.15	Glutamate decarboxylase.	NF	NF	NF	NF
2940	4.1.1.5	Acetolactate decarboxylase.	NF	NF	NF	NF
2941	4.1.1.70	Glutaconyl-CoA decarboxylase.	NF	NF	NF	NF
2942	4.1.3.4	Hydroxymethylglutaryl-CoA lyase.	NF	NF	NF	NF
2943	4.2.1.-	Hydro-lyases.	FOUND	FOUND	FOUND	FOUND
2944	4.2.1.17	Enoyl-CoA hydratase.	NF	NF	NF	NF
2945	4.2.1.27	Acetylenecarboxylate hydratase.	NF	NF	NF	NF
2946	4.2.1.31	Maleate hydratase.	NF	NF	NF	NF
2947	4.2.1.55	3-hydroxybutyryl-CoA dehydratase.	NF	NF	NF	NF
2948	5.1.2.3	3-hydroxybutyryl-CoA epimerase.	NF	NF	NF	NF
2949	5.1.2.4	Acetoin racemase.	NF	NF	NF	NF
2950	5.2.1.1	Maleate isomerase.	NF	NF	NF	NF
2951	5.3.3.3	Vinylacetyl-CoA Delta-isomerase.	NF	NF	NF	NF
2952	6.2.1.16	Acetoacetate--CoA ligase.	NF	NF	NF	NF
2953	6.2.1.2	Butyrate--CoA ligase.	NF	NF	NF	NF
2954	>C5-Branched dibasic acid metabolism 00660					
2955	2.2.1.6	Acetolactate synthase.	NF	NF	FOUND	FOUND
2956	2.3.3.11	2-hydroxyglutarate synthase.	NF	NF	NF	NF
2957	2.8.3.11	Citramalate CoA-transferase.	NF	NF	NF	NF
2958	2.8.3.7	Succinate--citramalate CoA-transferase.	NF	NF	NF	NF
2959	3.5.1.67	4-methyleneglutaminase.	NF	NF	NF	NF
2960	4.1.1.5	Acetolactate decarboxylase.	NF	NF	NF	NF
2961	4.1.1.6	Aconitate decarboxylase.	NF	NF	NF	NF
2962	4.1.3.17	4-hydroxy-4-methyl-2-oxoglutarate aldolase.	NF	NF	NF	NF
2963	4.1.3.22	Citramalate lyase.	NF	NF	NF	NF
2964	4.1.3.24	Malyl-CoA lyase.	NF	NF	NF	NF

2965	4.1.3.25	Citramalyl-CoA lyase.	NF	NF	NF	NF
2966	4.2.1.34	(S)-2-methylmalate dehydratase.	NF	NF	NF	NF
2967	4.2.1.35	(R)-2-methylmalate dehydratase.	NF	NF	NF	NF
2968	4.2.1.56	Itaconyl-CoA hydratase.	NF	NF	NF	NF
2969	4.3.1.2	Methylaspartate ammonia-lyase.	NF	NF	NF	NF
2970	5.4.99.1	Methylaspartate mutase.	NF	NF	NF	NF
2971	6.2.1.5	Succinate--CoA ligase (ADP-forming).	NF	NF	NF	NF
2972	6.3.1.7	4-methyleneglutamate--ammonia ligase.	NF	NF	NF	NF
2973	>One carbon pool by folate 00670					
2974	1.5.1.15	Methylenetetrahydrofolate dehydrogenase (NAD(+	NF	NF	NF	NF
2975	1.5.1.20	Methylenetetrahydrofolate reductase (NAD(P)H).	NF	NF	FOUND	FOUND
2976	1.5.1.3	Dihydrofolate reductase.	FOUND	FOUND	FOUND	FOUND
2977	1.5.1.5	Methylenetetrahydrofolate dehydrogenase (NADP(	FOUND	NF	NF	FOUND
2978	1.5.1.6	Formyltetrahydrofolate dehydrogenase.	NF	NF	NF	NF
2979	1.5.7.1	Methylenetetrahydrofolate reductase (ferredoxin).	NF	NF	NF	NF
2980	2.1.1.13	Methionine synthase.	NF	NF	FOUND	FOUND
2981	2.1.1.148	Thymidylate synthase (FAD).	NF	NF	NF	NF
2982	2.1.1.19	Trimethylsulfonium--tetrahydrofolate N-methyltran	NF	NF	NF	NF
2983	2.1.1.45	Thymidylate synthase.	FOUND	NF	FOUND	FOUND
2984	2.1.2.1	Glycine hydroxymethyltransferase.	NF	NF	FOUND	FOUND
2985	2.1.2.10	Aminomethyltransferase.	NF	NF	NF	NF
2986	2.1.2.2	Phosphoribosylglycinamide formyltransferase.	NF	FOUND	FOUND	FOUND
2987	2.1.2.3	Phosphoribosylaminoimidazolecarboxamide formyl	NF	NF	NF	NF
2988	2.1.2.4	Glycine formimidoyltransferase.	NF	NF	NF	NF
2989	2.1.2.5	Glutamate formimidoyltransferase.	NF	NF	NF	NF
2990	2.1.2.7	D-alanine 2-hydroxymethyltransferase.	NF	NF	NF	NF
2991	2.1.2.8	Deoxycytidylate 5-hydroxymethyltransferase.	NF	NF	NF	NF
2992	2.1.2.9	Methionyl-tRNA formyltransferase.	FOUND	FOUND	FOUND	FOUND
2993	3.5.1.10	Formyltetrahydrofolate deformylase.	NF	NF	NF	NF
2994	3.5.4.9	Methenyltetrahydrofolate cyclohydrolase.	FOUND	FOUND	FOUND	FOUND
2995	4.3.1.4	Formimidoyltetrahydrofolate cyclodeaminase.	NF	NF	NF	NF
2996	6.3.3.2	5-formyltetrahydrofolate cyclo-ligase.	NF	NF	FOUND	FOUND
2997	6.3.4.3	Formate--tetrahydrofolate ligase.	FOUND	FOUND	FOUND	FOUND
2998	>Methane metabolism 00680					
2999	1.1.1.244	Methanol dehydrogenase.	NF	NF	NF	NF
3000	1.1.1.272	(R)-2-hydroxyacid dehydrogenase.	NF	NF	NF	NF
3001	1.1.1.284	S-(hydroxymethyl)glutathione dehydrogenase.	NF	NF	NF	NF
3002	1.1.1.29	Glycerate dehydrogenase.	NF	NF	NF	NF
3003	1.1.1.37	Malate dehydrogenase.	NF	NF	NF	NF
3004	1.1.1.95	Phosphoglycerate dehydrogenase.	FOUND	NF	FOUND	FOUND
3005	1.1.2.7	Methanol dehydrogenase (cytochrome c).	NF	NF	NF	NF
3006	1.1.3.13	Alcohol oxidase.	NF	NF	NF	NF
3007	1.11.1.6	Catalase.	NF	NF	NF	NF
3008	1.11.1.7	Peroxidase.	FOUND	FOUND	FOUND	FOUND
3009	1.12.1.2	Hydrogen dehydrogenase.	NF	NF	NF	NF
3010	1.12.2.-	With a cytochrome as acceptor.	NF	NF	NF	NF
3011	1.12.7.2	Ferredoxin hydrogenase.	NF	NF	NF	FOUND
3012	1.12.98.1	Coenzyme F420 hydrogenase.	NF	NF	NF	NF
3013	1.12.98.2	5,10-methenyltetrahydromethanopterin hydrogena	NF	NF	NF	NF
3014	1.12.99.-	With other acceptors.	NF	NF	NF	NF
3015	1.14.13.25	Methane monooxygenase.	NF	NF	NF	NF
3016	1.14.13.8	Flavin-containing monooxygenase.	NF	NF	NF	NF
3017	1.2.1.2	Formate dehydrogenase.	NF	NF	NF	FOUND
3018	1.2.1.43	Formate dehydrogenase (NADP(+)).	NF	NF	NF	NF
3019	1.2.1.46	Formaldehyde dehydrogenase.	NF	NF	NF	NF

3020	1.2.7.1	Pyruvate synthase.	NF	NF	NF	NF
3021	1.2.7.4	Carbon-monoxide dehydrogenase (ferredoxin).	NF	NF	NF	NF
3022	1.2.99.2	Carbon-monoxide dehydrogenase (acceptor).	NF	NF	NF	NF
3023	1.2.99.5	Formylmethanofuran dehydrogenase.	NF	NF	NF	NF
3024	1.4.99.3	Amine dehydrogenase.	NF	NF	NF	NF
3025	1.5.1.-	With NAD(+) or NADP(+) as acceptor.	FOUND	FOUND	FOUND	FOUND
3026	1.5.1.20	Methylenetetrahydrofolate reductase (NAD(P)H).	NF	NF	FOUND	FOUND
3027	1.5.8.1	Dimethylamine dehydrogenase.	NF	NF	NF	NF
3028	1.5.8.2	Trimethylamine dehydrogenase.	NF	NF	NF	NF
3029	1.5.99.11	5,10-methylenetetrahydromethanopterin reductase	NF	NF	NF	NF
3030	1.5.99.5	Methylglutamate dehydrogenase.	NF	NF	NF	NF
3031	1.5.99.9	Methylenetetrahydromethanopterin dehydrogenas	NF	NF	NF	NF
3032	1.6.6.9	Trimethylamine-N-oxide reductase.	NF	NF	NF	NF
3033	1.8.98.1	CoB--CoM heterodisulfide reductase.	NF	NF	NF	NF
3034	2.1.1.-	Methyltransferases.	FOUND	FOUND	FOUND	FOUND
3035	2.1.1.21	Methylamine--glutamate N-methyltransferase.	NF	NF	NF	NF
3036	2.1.1.86	Tetrahydromethanopterin S-methyltransferase.	NF	NF	NF	NF
3037	2.1.1.90	Methanol--5-hydroxybenzimidazolylcobamide Co-m	NF	NF	NF	NF
3038	2.1.2.1	Glycine hydroxymethyltransferase.	NF	NF	FOUND	FOUND
3039	2.2.1.3	Formaldehyde transketolase.	NF	NF	NF	NF
3040	2.3.1.-	Transferring groups other than amino-acyl groups.	FOUND	FOUND	FOUND	FOUND
3041	2.3.1.101	Formylmethanofuran--tetrahydromethanopterin N-	NF	NF	NF	NF
3042	2.3.1.169	CO-methylating acetyl-CoA synthase.	NF	NF	NF	NF
3043	2.3.1.8	Phosphate acetyltransferase.	FOUND	FOUND	FOUND	FOUND
3044	2.6.1.45	Serine--glyoxylate transaminase.	NF	NF	NF	NF
3045	2.6.1.52	Phosphoserine transaminase.	NF	NF	NF	NF
3046	2.7.1.11	6-phosphofructokinase.	FOUND	FOUND	FOUND	FOUND
3047	2.7.1.29	Glycerone kinase.	FOUND	FOUND	FOUND	FOUND
3048	2.7.1.31	Glycerate kinase.	FOUND	NF	NF	FOUND
3049	2.7.2.1	Acetate kinase.	FOUND	FOUND	FOUND	FOUND
3050	2.7.9.2	Pyruvate, water dikinase.	NF	NF	NF	FOUND
3051	2.8.4.1	Coenzyme-B sulfoethylthiotransferase.	NF	NF	NF	NF
3052	3.1.2.12	S-formylglutathione hydrolase.	NF	NF	NF	NF
3053	3.1.3.11	Fructose-bisphosphatase.	NF	NF	NF	FOUND
3054	3.1.3.3	Phosphoserine phosphatase.	NF	NF	NF	NF
3055	3.1.3.71	2-phosphosulfolactate phosphatase.	NF	NF	NF	NF
3056	3.5.4.27	Methenyltetrahydromethanopterin cyclohydrolase.	NF	NF	NF	NF
3057	3.6.3.14	H(+)-transporting two-sector ATPase.	FOUND	NF	FOUND	NF
3058	4.1.1.31	Phosphoenolpyruvate carboxylase.	FOUND	FOUND	FOUND	NF
3059	4.1.1.79	Sulfopyruvate decarboxylase.	NF	NF	NF	NF
3060	4.1.2.13	Fructose-bisphosphate aldolase.	FOUND	FOUND	FOUND	FOUND
3061	4.1.2.32	Trimethylamine-oxide aldolase.	NF	NF	NF	NF
3062	4.1.2.43	3-hexulose-6-phosphate synthase.	NF	NF	NF	NF
3063	4.1.2.9	Phosphoketolase.	NF	NF	NF	NF
3064	4.1.3.24	Malyl-CoA lyase.	NF	NF	NF	NF
3065	4.2.1.11	Phosphopyruvate hydratase.	FOUND	FOUND	FOUND	FOUND
3066	4.3.-.-	Carbon-nitrogen lyases.	FOUND	FOUND	FOUND	FOUND
3067	4.4.1.19	Phosphosulfolactate synthase.	NF	NF	NF	NF
3068	4.4.1.22	S-(hydroxymethyl)glutathione synthase.	NF	NF	NF	NF
3069	5.3.1.27	6-phospho-3-hexuloisomerase.	NF	NF	NF	NF
3070	5.4.2.1	Phosphoglycerate mutase.	FOUND	FOUND	FOUND	FOUND
3071	6.2.1.1	Acetate--CoA ligase.	NF	NF	NF	NF
3072	6.2.1.9	Malate--CoA ligase.	NF	NF	NF	NF
3073	>Carbon fixation in photosynthetic organisms 00710					
3074	1.1.1.37	Malate dehydrogenase.	NF	NF	NF	NF

3075	1.1.1.39	Malate dehydrogenase (decarboxylating).	NF	NF	NF	NF
3076	1.1.1.40	Malate dehydrogenase (oxaloacetate-decarboxylati	NF	NF	FOUND	NF
3077	1.1.1.82	Malate dehydrogenase (NADP(+)).	NF	NF	NF	NF
3078	1.2.1.13	Glyceraldehyde-3-phosphate dehydrogenase (NADF	NF	NF	NF	NF
3079	1.2.1.59	Glyceraldehyde-3-phosphate dehydrogenase (NAD(	NF	NF	NF	NF
3080	2.2.1.1	Transketolase.	FOUND	FOUND	FOUND	FOUND
3081	2.6.1.1	Aspartate transaminase.	FOUND	FOUND	FOUND	FOUND
3082	2.6.1.2	Alanine transaminase.	NF	NF	NF	NF
3083	2.7.1.14	Sedoheptulokinase.	NF	NF	NF	NF
3084	2.7.1.19	Phosphoribulokinase.	NF	NF	NF	NF
3085	2.7.1.40	Pyruvate kinase.	FOUND	FOUND	FOUND	FOUND
3086	2.7.2.3	Phosphoglycerate kinase.	FOUND	FOUND	FOUND	FOUND
3087	2.7.9.1	Pyruvate, phosphate dikinase.	NF	NF	NF	NF
3088	3.1.3.11	Fructose-bisphosphatase.	NF	NF	NF	FOUND
3089	3.1.3.37	Sedoheptulose-bisphosphatase.	NF	NF	NF	NF
3090	4.1.1.31	Phosphoenolpyruvate carboxylase.	FOUND	FOUND	FOUND	NF
3091	4.1.1.39	Ribulose-bisphosphate carboxylase.	NF	NF	NF	NF
3092	4.1.1.49	Phosphoenolpyruvate carboxykinase (ATP).	NF	NF	FOUND	FOUND
3093	4.1.2.13	Fructose-bisphosphate aldolase.	FOUND	FOUND	FOUND	FOUND
3094	4.1.2.22	Fructose-6-phosphate phosphoketolase.	NF	NF	NF	NF
3095	4.1.2.9	Phosphoketolase.	NF	NF	NF	NF
3096	5.1.3.1	Ribulose-phosphate 3-epimerase.	FOUND	FOUND	FOUND	FOUND
3097	5.3.1.1	Triose-phosphate isomerase.	FOUND	FOUND	FOUND	FOUND
3098	5.3.1.6	Ribose-5-phosphate isomerase.	FOUND	FOUND	FOUND	FOUND
3099	>Carbon fixation pathways in prokaryotes 00720					
3100	1.1.1.-	With NAD(+) or NADP(+) as acceptor.	FOUND	FOUND	FOUND	FOUND
3101	1.1.1.298	3-hydroxypropionate dehydrogenase (NADP(+)).	NF	NF	NF	NF
3102	1.1.1.35	3-hydroxyacyl-CoA dehydrogenase.	NF	NF	NF	NF
3103	1.1.1.37	Malate dehydrogenase.	NF	NF	NF	NF
3104	1.1.1.42	Isocitrate dehydrogenase (NADP(+)).	NF	NF	FOUND	FOUND
3105	1.2.1.43	Formate dehydrogenase (NADP(+)).	NF	NF	NF	NF
3106	1.2.1.75	Malonyl CoA reductase (malonate semialdehyde-fo	NF	NF	NF	NF
3107	1.2.1.76	Succinate-semialdehyde dehydrogenase (acetylatin	NF	NF	NF	NF
3108	1.2.7.1	Pyruvate synthase.	NF	NF	NF	NF
3109	1.2.7.3	2-oxoglutarate synthase.	NF	NF	NF	NF
3110	1.2.7.4	Carbon-monoxide dehydrogenase (ferredoxin).	NF	NF	NF	NF
3111	1.2.99.2	Carbon-monoxide dehydrogenase (acceptor).	NF	NF	NF	NF
3112	1.3.1.84	Acrylyl-CoA reductase (NADPH).	NF	NF	NF	NF
3113	1.3.99.1	Succinate dehydrogenase.	NF	NF	NF	NF
3114	1.5.1.20	Methylenetetrahydrofolate reductase (NAD(P)H).	NF	NF	FOUND	FOUND
3115	1.5.1.5	Methylenetetrahydrofolate dehydrogenase (NADP(	FOUND	NF	NF	FOUND
3116	2.3.1.169	CO-methylating acetyl-CoA synthase.	NF	NF	NF	NF
3117	2.3.1.8	Phosphate acetyltransferase.	FOUND	FOUND	FOUND	FOUND
3118	2.3.1.9	Acetyl-CoA C-acetyltransferase.	FOUND	FOUND	NF	NF
3119	2.3.3.8	ATP citrate synthase.	NF	NF	NF	NF
3120	2.7.2.1	Acetate kinase.	FOUND	FOUND	FOUND	FOUND
3121	2.7.9.2	Pyruvate, water dikinase.	NF	NF	NF	FOUND
3122	2.8.3.-	CoA-transferases.	NF	NF	NF	NF
3123	3.5.4.9	Methenyltetrahydrofolate cyclohydrolase.	FOUND	FOUND	FOUND	FOUND
3124	4.1.1.31	Phosphoenolpyruvate carboxylase.	FOUND	FOUND	FOUND	NF
3125	4.1.3.24	Malyl-CoA lyase.	NF	NF	NF	NF
3126	4.2.1.116	3-hydroxypropionyl-CoA dehydratase.	NF	NF	NF	NF
3127	4.2.1.120	4-hydroxybutanoyl-CoA dehydratase.	NF	NF	NF	NF
3128	4.2.1.2	Fumarate hydratase.	NF	NF	NF	FOUND
3129	4.2.1.3	Aconitate hydratase.	NF	NF	FOUND	NF

3130	4.2.1.55	3-hydroxybutyryl-CoA dehydratase.	NF	NF	NF	NF
3131	5.1.99.1	Methylmalonyl-CoA epimerase.	NF	NF	NF	NF
3132	5.3.3.3	Vinylacetyl-CoA Delta-isomerase.	NF	NF	NF	NF
3133	5.4.99.2	Methylmalonyl-CoA mutase.	NF	NF	NF	NF
3134	6.2.1.-	Acid--thiol ligases.	NF	NF	NF	NF
3135	6.2.1.1	Acetate--CoA ligase.	NF	NF	NF	NF
3136	6.2.1.36	3-hydroxypropionyl-CoA synthase.	NF	NF	NF	NF
3137	6.2.1.5	Succinate--CoA ligase (ADP-forming).	NF	NF	NF	NF
3138	6.3.4.3	Formate--tetrahydrofolate ligase.	FOUND	FOUND	FOUND	FOUND
3139	6.4.1.2	Acetyl-CoA carboxylase.	NF	NF	FOUND	FOUND
3140	6.4.1.3	Propionyl-CoA carboxylase.	NF	NF	NF	NF
3141	>Thiamine metabolism 00730					
3142	1.1.3.23	Thiamine oxidase.	NF	NF	NF	NF
3143	1.4.3.19	Glycine oxidase.	NF	NF	NF	NF
3144	2.5.1.2	Thiamine pyridinylase.	NF	NF	NF	NF
3145	2.5.1.3	Thiamine-phosphate diphosphorylase.	NF	NF	NF	FOUND
3146	2.7.1.49	Hydroxymethylpyrimidine kinase.	NF	NF	NF	NF
3147	2.7.1.50	Hydroxyethylthiazole kinase.	NF	NF	NF	FOUND
3148	2.7.1.89	Thiamine kinase.	NF	NF	NF	NF
3149	2.7.4.15	Thiamine-diphosphate kinase.	NF	NF	NF	NF
3150	2.7.4.16	Thiamine-phosphate kinase.	NF	NF	NF	NF
3151	2.7.4.7	Phosphomethylpyrimidine kinase.	NF	NF	FOUND	FOUND
3152	2.7.6.2	Thiamine diphosphokinase.	FOUND	FOUND	FOUND	FOUND
3153	2.8.1.7	Cysteine desulfurase.	NF	NF	NF	FOUND
3154	3.1.3.-	Phosphoric monoester hydrolases.	FOUND	FOUND	FOUND	FOUND
3155	3.5.99.2	Thiaminase.	NF	NF	NF	NF
3156	3.6.1.15	Nucleoside-triphosphatase.	FOUND	FOUND	FOUND	FOUND
3157	3.6.1.28	Thiamine-triphosphatase.	NF	NF	NF	NF
3158	>Riboflavin metabolism 00740					
3159	1.1.1.193	5-amino-6-(5-phosphoribosylamino)uracil reductase	NF	NF	FOUND	FOUND
3160	1.1.1.302	2,5-diamino-6-(ribosylamino)-4(3H)-pyrimidinone 5	NF	NF	NF	NF
3161	1.14.18.1	Monophenol monooxygenase.	NF	NF	NF	NF
3162	1.14.99.40	5,6-dimethylbenzimidazole synthase.	NF	NF	NF	NF
3163	1.5.1.29	FMN reductase.	NF	NF	NF	FOUND
3164	1.5.1.30	Flavin reductase.	NF	NF	NF	NF
3165	2.5.1.78	6,7-dimethyl-8-ribityllumazine synthase.	NF	NF	NF	NF
3166	2.5.1.9	Riboflavin synthase.	NF	NF	FOUND	FOUND
3167	2.7.1.161	CTP-dependent riboflavin kinase.	NF	NF	NF	NF
3168	2.7.1.26	Riboflavin kinase.	NF	NF	NF	NF
3169	2.7.7.2	FAD synthetase.	FOUND	FOUND	FOUND	FOUND
3170	3.1.3.-	Phosphoric monoester hydrolases.	FOUND	FOUND	FOUND	FOUND
3171	3.1.3.2	Acid phosphatase.	FOUND	FOUND	FOUND	FOUND
3172	3.5.1.102	2-amino-5-formylamino-6-ribosylaminopyrimidin-4(	NF	NF	NF	NF
3173	3.5.4.25	GTP cyclohydrolase II.	NF	NF	FOUND	FOUND
3174	3.5.4.26	Diaminohydroxyphosphoribosylaminopyrimidine de	NF	NF	NF	NF
3175	3.5.4.29	GTP cyclohydrolase IIa.	NF	NF	NF	NF
3176	3.5.99.1	Riboflavinase.	NF	NF	NF	NF
3177	3.6.1.18	FAD diphosphatase.	NF	NF	NF	NF
3178	3.6.1.9	Nucleotide diphosphatase.	NF	NF	NF	NF
3179	4.1.99.12	3,4-dihydroxy-2-butanone-4-phosphate synthase.	NF	NF	NF	NF
3180	>Vitamin B6 metabolism 00750					
3181	1.1.1.107	Pyridoxal 4-dehydrogenase.	NF	NF	NF	NF
3182	1.1.1.262	4-hydroxythreonine-4-phosphate dehydrogenase.	NF	NF	NF	NF
3183	1.1.1.290	4-phosphoerythronate dehydrogenase.	NF	NF	NF	NF
3184	1.1.1.65	Pyridoxine 4-dehydrogenase.	NF	NF	NF	NF

3185	1.1.3.12	Pyridoxine 4-oxidase.	NF	NF	NF	NF
3186	1.1.99.9	Pyridoxine 5-dehydrogenase.	NF	NF	NF	NF
3187	1.14.12.4	3-hydroxy-2-methylpyridinecarboxylate dioxygenas	NF	NF	NF	NF
3188	1.14.12.5	5-pyridoxate dioxygenase.	NF	NF	NF	NF
3189	1.2.1.72	Erythrose-4-phosphate dehydrogenase.	NF	NF	NF	NF
3190	1.2.3.1	Aldehyde oxidase.	NF	NF	NF	NF
3191	1.2.3.8	Pyridoxal oxidase.	NF	NF	NF	NF
3192	1.4.3.5	Pyridoxal 5'-phosphate synthase.	NF	NF	FOUND	NF
3193	2.6.-.-	Transferring nitrogenous groups.	FOUND	FOUND	FOUND	FOUND
3194	2.6.1.30	Pyridoxamine--pyruvate transaminase.	NF	NF	NF	NF
3195	2.6.1.31	Pyridoxamine--oxaloacetate transaminase.	NF	NF	NF	NF
3196	2.6.1.52	Phosphoserine transaminase.	NF	NF	NF	NF
3197	2.6.1.54	Pyridoxamine-phosphate transaminase.	NF	NF	NF	NF
3198	2.6.99.2	Pyridoxine 5'-phosphate synthase.	NF	NF	NF	NF
3199	2.7.1.35	Pyridoxal kinase.	NF	NF	FOUND	NF
3200	3.1.1.27	4-pyridoxolactonase.	NF	NF	NF	NF
3201	3.1.3.74	Pyridoxal phosphatase.	NF	NF	NF	NF
3202	3.5.1.29	2-(acetamidomethylene)succinate hydrolase.	NF	NF	NF	NF
3203	3.5.1.66	2-(hydroxymethyl)-3-(acetamidomethylene)succina	NF	NF	NF	NF
3204	4.-.-.-	Lyases.	FOUND	FOUND	FOUND	FOUND
3205	4.1.1.51	3-hydroxy-2-methylpyridine-4,5-dicarboxylate 4-de	NF	NF	NF	NF
3206	4.2.3.1	Threonine synthase.	NF	NF	FOUND	FOUND
3207	>Nicotinate and nicotinamide metabolism 00760					
3208	1.1.1.291	2-hydroxymethylglutarate dehydrogenase.	NF	NF	NF	NF
3209	1.13.11.9	2,5-dihydroxypyridine 5,6-dioxygenase.	NF	NF	NF	NF
3210	1.14.13.10	2,6-dihydroxypyridine 3-monooxygenase.	NF	NF	NF	NF
3211	1.14.18.-	With another compound as one donor, and incorpo	NF	NF	NF	NF
3212	1.14.99.-	Miscellaneous (requires further characterization).	NF	NF	NF	NF
3213	1.17.1.5	Nicotinate dehydrogenase.	NF	NF	NF	NF
3214	1.17.3.3	6-hydroxynicotinate dehydrogenase.	NF	NF	NF	NF
3215	1.2.3.1	Aldehyde oxidase.	NF	NF	NF	NF
3216	1.3.7.1	6-hydroxynicotinate reductase.	NF	NF	NF	NF
3217	1.4.1.21	Aspartate dehydrogenase.	NF	NF	NF	NF
3218	1.4.3.16	L-aspartate oxidase.	NF	NF	NF	NF
3219	1.5.3.5	(S)-6-hydroxynicotine oxidase.	NF	NF	NF	NF
3220	1.5.3.6	(R)-6-hydroxynicotine oxidase.	NF	NF	NF	NF
3221	1.5.99.4	Nicotine dehydrogenase.	NF	NF	NF	NF
3222	1.6.1.1	NAD(P)(+) transhydrogenase (B-specific).	NF	NF	NF	NF
3223	1.6.1.2	NAD(P)(+) transhydrogenase (AB-specific).	NF	NF	NF	NF
3224	2.1.1.1	Nicotinamide N-methyltransferase.	NF	NF	NF	NF
3225	2.1.1.7	Nicotinate N-methyltransferase.	NF	NF	NF	NF
3226	2.4.2.1	Purine-nucleoside phosphorylase.	FOUND	FOUND	FOUND	FOUND
3227	2.4.2.11	Nicotinate phosphoribosyltransferase.	NF	NF	NF	NF
3228	2.4.2.12	Nicotinamide phosphoribosyltransferase.	NF	NF	NF	NF
3229	2.4.2.19	Nicotinate-nucleotide diphosphorylase (carboxylati	FOUND	FOUND	FOUND	FOUND
3230	2.7.1.-	Phosphotransferases with an alcohol group as acce	FOUND	FOUND	FOUND	FOUND
3231	2.7.1.22	Ribosylnicotinamide kinase.	NF	NF	NF	NF
3232	2.7.1.23	NAD(+) kinase.	FOUND	FOUND	FOUND	FOUND
3233	2.7.7.1	Nicotinamide-nucleotide adenylyltransferase.	FOUND	FOUND	FOUND	NF
3234	2.7.7.18	Nicotinate-nucleotide adenylyltransferase.	NF	NF	NF	FOUND
3235	3.1.3.-	Phosphoric monoester hydrolases.	FOUND	FOUND	FOUND	FOUND
3236	3.1.3.5	5'-nucleotidase.	FOUND	FOUND	FOUND	FOUND
3237	3.2.2.1	Purine nucleosidase.	NF	FOUND	FOUND	FOUND
3238	3.2.2.14	NMN nucleosidase.	NF	NF	NF	NF
3239	3.2.2.5	NAD(+) nucleosidase.	NF	NF	NF	NF

3240	3.2.2.6	NAD(P)(+) nucleosidase.	NF	NF	NF	NF
3241	3.5.1.107	Maleamate amidohydrolase.	NF	NF	NF	NF
3242	3.5.1.19	Nicotinamidase.	NF	NF	NF	FOUND
3243	3.5.1.42	Nicotinamide-nucleotide amidase.	NF	NF	NF	NF
3244	3.5.2.18	Enamidase.	NF	NF	NF	NF
3245	3.6.1.22	NAD(+) diphosphatase.	NF	NF	FOUND	FOUND
3246	3.6.1.9	Nucleotide diphosphatase.	NF	NF	NF	NF
3247	3.7.1.-	In ketonic substances.	NF	NF	NF	FOUND
3248	4.1.3.32	2,3-dimethylmalate lyase.	NF	NF	NF	NF
3249	4.2.1.85	Dimethylmaleate hydratase.	NF	NF	NF	NF
3250	5.2.1.1	Maleate isomerase.	NF	NF	NF	NF
3251	5.3.3.6	Methylitaconate Delta-isomerase.	NF	NF	NF	NF
3252	5.4.99.4	2-methyleneglutarate mutase.	NF	NF	NF	NF
3253	6.3.1.5	NAD(+) synthase.	NF	NF	NF	FOUND
3254	6.3.5.1	NAD(+) synthase (glutamine-hydrolyzing).	NF	NF	FOUND	FOUND
3255	>Pantothenate and CoA biosynthesis 00770					
3256	1.1.1.106	Pantoate 4-dehydrogenase.	NF	NF	NF	NF
3257	1.1.1.169	2-dehydropantoate 2-reductase.	FOUND	FOUND	NF	NF
3258	1.1.1.84	Dimethylmalate dehydrogenase.	NF	NF	NF	NF
3259	1.1.1.86	Ketol-acid reductoisomerase.	NF	NF	FOUND	FOUND
3260	1.2.1.33	(R)-dehydropantoate dehydrogenase.	NF	NF	NF	NF
3261	1.3.1.1	Dihydrouracil dehydrogenase (NAD(+)).	NF	NF	NF	NF
3262	1.3.1.2	Dihydropyrimidine dehydrogenase (NADP(+)).	NF	NF	NF	NF
3263	2.1.2.11	3-methyl-2-oxobutanoate hydroxymethyltransferas	NF	NF	NF	NF
3264	2.2.1.6	Acetolactate synthase.	NF	NF	FOUND	FOUND
3265	2.6.1.42	Branched-chain-amino-acid transaminase.	NF	NF	FOUND	FOUND
3266	2.6.1.6	Leucine transaminase.	NF	NF	NF	NF
3267	2.7.1.24	Dephospho-CoA kinase.	FOUND	FOUND	FOUND	FOUND
3268	2.7.1.33	Pantothenate kinase.	NF	FOUND	FOUND	FOUND
3269	2.7.1.34	Pantetheine kinase.	NF	NF	NF	NF
3270	2.7.7.3	Pantetheine-phosphate adenylyltransferase.	FOUND	FOUND	FOUND	FOUND
3271	2.7.8.7	Holo-[acyl-carrier-protein] synthase.	FOUND	FOUND	FOUND	FOUND
3272	3.1.4.14	[Acyl-carrier-protein] phosphodiesterase.	NF	NF	FOUND	FOUND
3273	3.5.1.22	Pantothenase.	NF	NF	NF	NF
3274	3.5.1.6	Beta-ureidopropionase.	NF	NF	NF	NF
3275	3.5.1.92	Pantetheine hydrolase.	NF	NF	NF	NF
3276	3.5.2.2	Dihydropyrimidinase.	NF	NF	NF	FOUND
3277	3.6.1.9	Nucleotide diphosphatase.	NF	NF	NF	NF
3278	4.1.1.11	Aspartate 1-decarboxylase.	NF	NF	NF	NF
3279	4.1.1.30	Pantothenoylcysteine decarboxylase.	NF	NF	NF	NF
3280	4.1.1.36	Phosphopantothenoylcysteine decarboxylase.	NF	NF	NF	NF
3281	4.2.1.9	Dihydroxy-acid dehydratase.	NF	NF	FOUND	FOUND
3282	6.3.2.1	Pantoate--beta-alanine ligase.	NF	NF	NF	NF
3283	6.3.2.5	Phosphopantothenate--cysteine ligase.	FOUND	FOUND	FOUND	FOUND
3284	>Biotin metabolism 00780					
3285	2.3.1.47	8-amino-7-oxononanoate synthase.	NF	NF	NF	NF
3286	2.6.1.62	Adenosylmethionine--8-amino-7-oxononanoate tra	NF	NF	NF	NF
3287	2.8.1.6	Biotin synthase.	NF	NF	FOUND	NF
3288	3.4.-.-	Acting on peptide bonds (peptide hydrolases).	FOUND	FOUND	FOUND	FOUND
3289	3.5.1.12	Biotinidase.	NF	NF	NF	NF
3290	6.2.1.11	Biotin--CoA ligase.	NF	NF	NF	NF
3291	6.2.1.14	6-carboxyhexanoate--CoA ligase.	NF	NF	NF	NF
3292	6.3.3.3	Dethiobiotin synthase.	FOUND	FOUND	FOUND	FOUND
3293	6.3.4.10	Biotin--[propionyl-CoA-carboxylase (ATP-hydrolyzin	NF	NF	NF	NF
3294	6.3.4.11	Biotin--[methylcrotonoyl-CoA-carboxylase] ligase.	NF	NF	NF	NF

3295	6.3.4.15	Biotin--[acetyl-CoA-carboxylase] ligase.	NF	NF	FOUND	FOUND
3296	6.3.4.9	Biotin--[methylmalonyl-CoA-carboxytransferase] lig	NF	NF	NF	NF
3297	>Lipoic acid metabolism 00785					
3298	2.3.1.181	Lipoyl(octanoyl) transferase.	NF	NF	NF	NF
3299	2.7.7.63	Lipoate--protein ligase.	NF	NF	NF	NF
3300	2.8.1.8	Lipoyl synthase.	NF	NF	NF	NF
3301	6.-.-.-	Ligases.	FOUND	FOUND	FOUND	FOUND
3302	>Folate biosynthesis 00790					
3303	1.1.1.153	Sepiapterin reductase.	NF	NF	NF	NF
3304	1.1.1.220	6-pyruvoyltetrahydropterin 2'-reductase.	NF	NF	NF	NF
3305	1.5.1.3	Dihydrofolate reductase.	FOUND	FOUND	FOUND	FOUND
3306	1.5.1.34	6,7-dihydropteridine reductase.	NF	NF	NF	FOUND
3307	2.5.1.15	Dihydropteroate synthase.	NF	NF	FOUND	FOUND
3308	2.6.1.85	Aminodeoxychorismate synthase.	NF	NF	NF	NF
3309	2.7.6.3	2-amino-4-hydroxy-6-hydroxymethyldihydropteridi	NF	NF	FOUND	FOUND
3310	3.1.3.1	Alkaline phosphatase.	FOUND	FOUND	FOUND	FOUND
3311	3.4.19.9	Gamma-glutamyl hydrolase.	NF	NF	NF	NF
3312	3.5.4.16	GTP cyclohydrolase I.	NF	NF	FOUND	FOUND
3313	3.6.1.-	In phosphorous-containing anhydrides.	FOUND	FOUND	FOUND	FOUND
3314	4.1.2.25	Dihydroneopterin aldolase.	NF	NF	FOUND	NF
3315	4.1.3.38	Aminodeoxychorismate lyase.	NF	NF	NF	FOUND
3316	4.2.3.12	6-pyruvoyltetrahydropterin synthase.	NF	NF	NF	NF
3317	6.3.2.12	Dihydrofolate synthase.	NF	NF	NF	NF
3318	6.3.2.17	Tetrahydrofolate synthase.	FOUND	FOUND	FOUND	FOUND
3319	>Atrazine degradation 00791					
3320	1.14.15.-	With a reduced iron-sulfur protein as one donor, an	NF	NF	NF	NF
3321	3.5.1.5	Urease.	NF	NF	NF	NF
3322	3.5.1.54	Allophanate hydrolase.	NF	NF	NF	NF
3323	3.5.1.84	Biuret amidohydrolase.	NF	NF	NF	NF
3324	3.5.2.15	Cyanuric acid amidohydrolase.	NF	NF	NF	NF
3325	3.5.4.-	In cyclic amidines.	FOUND	FOUND	FOUND	FOUND
3326	3.5.99.-	In other compounds.	FOUND	FOUND	FOUND	FOUND
3327	3.5.99.3	Hydroxydechloroatrazine ethylaminohydrolase.	NF	NF	NF	NF
3328	3.5.99.4	N-isopropylammelide isopropylaminohydrolase.	NF	NF	NF	NF
3329	3.8.1.-	In C-halide compounds.	NF	NF	NF	NF
3330	3.8.1.8	Atrazine chlorohydrolase.	NF	NF	NF	NF
3331	4.2.1.69	Cyanamide hydratase.	NF	NF	NF	NF
3332	>Retinol metabolism 00830					
3333	1.1.-.-	Acting on the CH-OH group of donors.	FOUND	FOUND	FOUND	FOUND
3334	1.1.1.-	With NAD(+) or NADP(+) as acceptor.	FOUND	FOUND	FOUND	FOUND
3335	1.1.1.1	Alcohol dehydrogenase.	FOUND	FOUND	FOUND	FOUND
3336	1.14.14.1	Unspecific monooxygenase.	NF	NF	NF	NF
3337	1.14.15.3	Alkane 1-monooxygenase.	NF	NF	NF	NF
3338	1.14.99.36	Beta-carotene 15,15'-monooxygenase.	NF	NF	NF	NF
3339	1.2.1.36	Retinal dehydrogenase.	NF	NF	NF	NF
3340	1.2.3.11	Retinal oxidase.	NF	NF	NF	NF
3341	1.3.99.23	All-trans-retinol 13,14-reductase.	NF	NF	NF	NF
3342	2.3.1.135	Phosphatidylcholine--retinol O-acyltransferase.	NF	NF	NF	NF
3343	2.3.1.76	Retinol O-fatty-acyltransferase.	NF	NF	NF	NF
3344	2.4.1.17	Glucuronosyltransferase.	NF	NF	NF	NF
3345	3.1.1.-	Carboxylic ester hydrolases.	FOUND	FOUND	FOUND	FOUND
3346	3.1.1.21	Retinyl-palmitate esterase.	NF	NF	NF	NF
3347	3.1.1.63	11-cis-retinyl-palmitate hydrolase.	NF	NF	NF	NF
3348	3.1.1.64	All-trans-retinyl-palmitate hydrolase.	NF	NF	NF	NF
3349	5.2.1.3	Retinal isomerase.	NF	NF	NF	NF

3350	5.2.1.7	Retinol isomerase.	NF	NF	NF	NF
3351	>Porphyrin and chlorophyll metabolism 00860					
3352	1.-.-.-	Oxidoreductases.	FOUND	FOUND	FOUND	FOUND
3353	1.1.1.294	Chlorophyll(ide) b reductase.	NF	NF	NF	NF
3354	1.13.12.14	Chlorophyllide-a oxygenase.	NF	NF	NF	NF
3355	1.14.12.20	Pheophorbide a oxygenase.	NF	NF	NF	NF
3356	1.14.13.81	Magnesium-protoporphyrin IX monomethyl ester (c	NF	NF	NF	NF
3357	1.14.13.83	Precorrin-3B synthase.	NF	NF	NF	NF
3358	1.14.99.3	Heme oxygenase.	NF	NF	NF	NF
3359	1.16.1.2	Diferric-transferrin reductase.	NF	NF	NF	NF
3360	1.16.1.3	Aquacobalamin reductase.	NF	NF	NF	NF
3361	1.16.1.4	Cob(II)alamin reductase.	NF	NF	NF	NF
3362	1.16.1.5	Aquacobalamin reductase (NADPH).	NF	NF	NF	NF
3363	1.16.1.6	Cyanocobalamin reductase (cyanide-eliminating).	NF	NF	NF	NF
3364	1.16.3.1	Ferroxidase.	NF	NF	FOUND	FOUND
3365	1.16.8.1	Cob(II)yrinic acid a,c-diamide reductase.	NF	NF	NF	NF
3366	1.18.-.-	Acting on iron-sulfur proteins as donors.	NF	NF	NF	NF
3367	1.2.1.70	Glutamyl-tRNA reductase.	NF	NF	NF	NF
3368	1.3.1.24	Biliverdin reductase.	NF	NF	NF	NF
3369	1.3.1.33	Protochlorophyllide reductase.	NF	NF	NF	NF
3370	1.3.1.54	Precorrin-6A reductase.	NF	NF	FOUND	FOUND
3371	1.3.1.75	Divinyl chlorophyllide a 8-vinyl-reductase.	NF	NF	NF	NF
3372	1.3.1.76	Precorrin-2 dehydrogenase.	NF	NF	NF	NF
3373	1.3.1.80	Red chlorophyll catabolite reductase.	NF	NF	NF	NF
3374	1.3.3.3	Coproporphyrinogen oxidase.	FOUND	FOUND	FOUND	FOUND
3375	1.3.3.4	Protoporphyrinogen oxidase.	NF	NF	NF	NF
3376	1.3.3.5	Bilirubin oxidase.	NF	NF	NF	NF
3377	1.3.7.2	15,16-dihydrobiliverdin:ferredoxin oxidoreductase.	NF	NF	NF	NF
3378	1.3.7.3	Phycoerythrobilin:ferredoxin oxidoreductase.	NF	NF	NF	NF
3379	1.3.7.4	Phytochromobilin:ferredoxin oxidoreductase.	NF	NF	NF	NF
3380	1.3.7.5	Phycocyanobilin:ferredoxin oxidoreductase.	NF	NF	NF	NF
3381	1.3.99.22	Coproporphyrinogen dehydrogenase.	NF	NF	NF	NF
3382	2.1.1.-	Methyltransferases.	FOUND	FOUND	FOUND	FOUND
3383	2.1.1.107	Uroporphyrinogen-III C-methyltransferase.	NF	NF	FOUND	FOUND
3384	2.1.1.11	Magnesium protoporphyrin IX methyltransferase.	NF	NF	NF	NF
3385	2.1.1.130	Precorrin-2 C(20)-methyltransferase.	NF	NF	NF	NF
3386	2.1.1.131	Precorrin-3B C(17)-methyltransferase.	NF	NF	NF	NF
3387	2.1.1.132	Precorrin-6Y C(5,15)-methyltransferase (decarboxyl	NF	NF	NF	NF
3388	2.1.1.133	Precorrin-4 C(11)-methyltransferase.	NF	NF	NF	NF
3389	2.1.1.151	Cobalt-factor II C(20)-methyltransferase.	NF	NF	NF	NF
3390	2.1.1.152	Precorrin-6A synthase (deacetylating).	NF	NF	NF	NF
3391	2.3.1.37	5-aminolevulinate synthase.	NF	NF	NF	NF
3392	2.4.1.17	Glucuronosyltransferase.	NF	NF	NF	NF
3393	2.4.2.21	Nicotinate-nucleotide--dimethylbenzimidazole phos	NF	NF	FOUND	FOUND
3394	2.5.1.-	Transferring alkyl or aryl groups, other than methyl	FOUND	FOUND	FOUND	FOUND
3395	2.5.1.17	Cob(I)yrinic acid a,c-diamide adenosyltransferase.	NF	NF	FOUND	FOUND
3396	2.5.1.61	Hydroxymethylbilane synthase.	NF	NF	FOUND	FOUND
3397	2.5.1.62	Chlorophyll synthase.	NF	NF	NF	NF
3398	2.6.1.43	Aminolevulinate transaminase.	NF	NF	NF	NF
3399	2.7.1.156	Adenosylcobinamide kinase.	NF	NF	FOUND	FOUND
3400	2.7.7.62	Adenosylcobinamide-phosphate guanylyltransferase	NF	NF	FOUND	FOUND
3401	2.7.8.26	Adenosylcobinamide-GDP ribazoletransferase.	NF	NF	NF	NF
3402	3.1.1.14	Chlorophyllase.	NF	NF	NF	FOUND
3403	3.1.1.82	Pheophorbidase.	NF	NF	NF	NF
3404	3.1.3.73	Alpha-ribazole phosphatase.	NF	NF	NF	FOUND

3405	3.2.1.31	Beta-glucuronidase.	NF	NF	NF	NF
3406	3.5.1.90	Adenosylcobinamide hydrolase.	NF	NF	NF	NF
3407	4.-.-.-	Lyases.	FOUND	FOUND	FOUND	FOUND
3408	4.1.1.37	Uroporphyrinogen decarboxylase.	NF	NF	NF	NF
3409	4.1.1.81	Threonine-phosphate decarboxylase.	NF	NF	NF	NF
3410	4.2.1.-	Hydro-lyases.	FOUND	FOUND	FOUND	FOUND
3411	4.2.1.24	Porphobilinogen synthase.	NF	NF	FOUND	FOUND
3412	4.2.1.75	Uroporphyrinogen-III synthase.	NF	NF	FOUND	FOUND
3413	4.4.1.17	Holocytochrome-c synthase.	NF	NF	NF	NF
3414	4.99.1.1	Ferrochelatase.	NF	NF	NF	NF
3415	4.99.1.3	Sirohydrochlorin cobaltochelatase.	NF	NF	NF	NF
3416	4.99.1.4	Sirohydrochlorin ferrochelatase.	NF	NF	NF	NF
3417	5.4.1.2	Precorrin-8X methylmutase.	NF	NF	FOUND	FOUND
3418	5.4.3.8	Glutamate-1-semialdehyde 2,1-aminomutase.	NF	NF	FOUND	NF
3419	6.1.1.17	Glutamate--tRNA ligase.	FOUND	FOUND	FOUND	FOUND
3420	6.3.1.-	Acid--ammonia (or amide) ligases (amide synthases)	FOUND	FOUND	FOUND	FOUND
3421	6.3.1.10	Adenosylcobinamide-phosphate synthase.	NF	NF	NF	NF
3422	6.3.5.10	Adenosylcobyric acid synthase (glutamine-hydrolyzi	NF	NF	NF	NF
3423	6.3.5.9	Hydrogenobyrinic acid a,c-diamide synthase (glutam	NF	NF	NF	NF
3424	6.6.1.1	Magnesium chelatase.	NF	NF	FOUND	FOUND
3425	6.6.1.2	Cobaltochelatase.	NF	NF	NF	NF
3426	>Terpenoid backbone biosynthesis 00900					
3427	1.1.1.267	1-deoxy-D-xylulose-5-phosphate reductoisomerase.	NF	NF	NF	NF
3428	1.1.1.34	Hydroxymethylglutaryl-CoA reductase (NADPH).	FOUND	FOUND	FOUND	FOUND
3429	1.1.1.88	Hydroxymethylglutaryl-CoA reductase.	NF	NF	NF	NF
3430	1.17.1.2	4-hydroxy-3-methylbut-2-enyl diphosphate reducta	NF	NF	NF	FOUND
3431	1.17.7.1	(E)-4-hydroxy-3-methylbut-2-enyl-diphosphate synt	NF	NF	NF	FOUND
3432	1.3.1.83	Geranylgeranyl diphosphate reductase.	NF	NF	NF	NF
3433	2.2.1.7	1-deoxy-D-xylulose-5-phosphate synthase.	NF	NF	FOUND	FOUND
3434	2.3.1.9	Acetyl-CoA C-acetyltransferase.	FOUND	FOUND	NF	NF
3435	2.3.3.10	Hydroxymethylglutaryl-CoA synthase.	FOUND	FOUND	FOUND	NF
3436	2.5.1.-	Transferring alkyl or aryl groups, other than methyl	FOUND	FOUND	FOUND	FOUND
3437	2.5.1.1	Dimethylallyltranstransferase.	NF	NF	FOUND	FOUND
3438	2.5.1.10	(2E,6E)-farnesyl diphosphate synthase.	FOUND	NF	NF	NF
3439	2.5.1.20	Rubber cis-polyprenylcistransferase.	NF	NF	NF	NF
3440	2.5.1.29	Farnesyltranstransferase.	NF	NF	NF	NF
3441	2.5.1.30	Heptaprenyl diphosphate synthase.	NF	NF	NF	NF
3442	2.5.1.31	Di-trans,poly-cis-undecaprenyl-diphosphate synthas	FOUND	FOUND	FOUND	FOUND
3443	2.5.1.68	(2Z,6E)-farnesyl diphosphate synthase.	NF	NF	NF	NF
3444	2.5.1.82	Hexaprenyl diphosphate synthase (geranylgeranyl-d	NF	NF	NF	NF
3445	2.5.1.83	Hexaprenyl-diphosphate synthase ((2E,6E)-farnesyl-	NF	NF	NF	NF
3446	2.5.1.84	All-trans-nonaprenyl-diphosphate synthase (gerany	NF	NF	NF	NF
3447	2.5.1.85	All-trans-nonaprenyl-diphosphate synthase (gerany	NF	NF	NF	NF
3448	2.5.1.86	Trans,poly-cis-decaprenyl diphosphate synthase.	NF	NF	NF	NF
3449	2.7.1.148	4-(cytidine 5'-diphospho)-2-C-methyl-D-erythritol ki	NF	NF	NF	FOUND
3450	2.7.1.36	Mevalonate kinase.	FOUND	FOUND	FOUND	NF
3451	2.7.4.2	Phosphomevalonate kinase.	FOUND	FOUND	FOUND	NF
3452	2.7.7.60	2-C-methyl-D-erythritol 4-phosphate cytidylyltransf	NF	NF	NF	NF
3453	4.1.1.33	Diphosphomevalonate decarboxylase.	FOUND	FOUND	FOUND	NF
3454	4.2.3.27	Isoprene synthase.	NF	NF	NF	NF
3455	4.6.1.12	2-C-methyl-D-erythritol 2,4-cyclodiphosphate synth	NF	NF	NF	FOUND
3456	5.3.3.2	Isopentenyl-diphosphate Delta-isomerase.	FOUND	FOUND	FOUND	FOUND
3457	>Indole alkaloid biosynthesis 00901					
3458	1.1.1.273	Vellosimine dehydrogenase.	NF	NF	NF	NF
3459	1.14.11.20	Deacetoxyvindoline 4-hydroxylase.	NF	NF	NF	NF

3460	1.14.13.73	Tabersonine 16-hydroxylase.	NF	NF	NF	NF
3461	1.14.13.75	Vinorine hydroxylase.	NF	NF	NF	NF
3462	1.3.1.36	Geissoschizine dehydrogenase.	NF	NF	NF	NF
3463	1.3.1.73	1,2-dihydrovomilenine reductase.	NF	NF	NF	NF
3464	1.5.1.32	Vomilenine reductase.	NF	NF	NF	NF
3465	2.1.1.94	Tabersonine 16-O-methyltransferase.	NF	NF	NF	NF
3466	2.1.1.99	3-hydroxy-16-methoxy-2,3-dihydrotabersonine N-m	NF	NF	NF	NF
3467	2.3.1.107	Deacetylvindoline O-acetyltransferase.	NF	NF	NF	NF
3468	2.3.1.160	Vinorine synthase.	NF	NF	NF	NF
3469	2.4.1.219	Vomilenine glucosyltransferase.	NF	NF	NF	NF
3470	3.1.1.78	Polyneuridine-aldehyde esterase.	NF	NF	NF	NF
3471	3.2.1.105	3-alpha-(S)-strictosidine beta-glucosidase.	NF	NF	NF	NF
3472	3.2.1.125	Raucaffricine beta-glucosidase.	NF	NF	NF	NF
3473	4.1.1.28	Aromatic-L-amino-acid decarboxylase.	NF	NF	NF	NF
3474	4.3.3.2	Strictosidine synthase.	NF	NF	NF	NF
3475	>Monoterpenoid biosynthesis 00902					
3476	1.1.1.207	(-)-menthol dehydrogenase.	NF	NF	NF	NF
3477	1.1.1.208	(+)-neomenthol dehydrogenase.	NF	NF	NF	NF
3478	1.1.1.223	Isopiperitenol dehydrogenase.	NF	NF	NF	NF
3479	1.1.1.241	6-endo-hydroxycineole dehydrogenase.	NF	NF	NF	NF
3480	1.1.1.243	Carveol dehydrogenase.	NF	NF	NF	NF
3481	1.1.1.275	(+)-trans-carveol dehydrogenase.	NF	NF	NF	NF
3482	1.14.13.47	(S)-limonene 3-monooxygenase.	NF	NF	NF	NF
3483	1.14.13.48	(S)-limonene 6-monooxygenase.	NF	NF	NF	NF
3484	1.14.13.49	(S)-limonene 7-monooxygenase.	NF	NF	NF	NF
3485	1.14.13.51	6-oxocineole dehydrogenase.	NF	NF	NF	NF
3486	1.14.13.74	7-deoxyloganin 7-hydroxylase.	NF	NF	NF	NF
3487	1.14.13.80	(R)-limonene 6-monooxygenase.	NF	NF	NF	NF
3488	1.3.1.82	(-)-isopiperitenone reductase.	NF	NF	NF	NF
3489	1.3.3.9	Secologanin synthase.	NF	NF	NF	NF
3490	2.1.1.50	Loganate O-methyltransferase.	NF	NF	NF	NF
3491	3.1.7.3	Monoterpenyl-diphosphatase.	NF	NF	NF	NF
3492	4.2.3.-	Acting on phosphates.	NF	NF	FOUND	FOUND
3493	4.2.3.10	(-)-endo-fenchol synthase.	NF	NF	NF	NF
3494	4.2.3.11	Sabinene-hydrate synthase.	NF	NF	NF	NF
3495	4.2.3.14	Pinene synthase.	NF	NF	NF	NF
3496	4.2.3.15	Myrcene synthase.	NF	NF	NF	NF
3497	4.2.3.16	(4S)-limonene synthase.	NF	NF	NF	NF
3498	4.2.3.20	(R)-limonene synthase.	NF	NF	NF	NF
3499	4.2.3.26	R-linalool synthase.	NF	NF	NF	NF
3500	5.5.1.8	Bornyl diphosphate synthase.	NF	NF	NF	NF
3501	>Limonene and pinene degradation 00903					
3502	1.1.-.-	Acting on the CH-OH group of donors.	FOUND	FOUND	FOUND	FOUND
3503	1.1.1.144	Perillyl-alcohol dehydrogenase.	NF	NF	NF	NF
3504	1.1.1.243	Carveol dehydrogenase.	NF	NF	NF	NF
3505	1.1.1.275	(+)-trans-carveol dehydrogenase.	NF	NF	NF	NF
3506	1.1.1.296	Dihydrocarveol dehydrogenase.	NF	NF	NF	NF
3507	1.1.1.297	Limonene-1,2-diol dehydrogenase.	NF	NF	NF	NF
3508	1.14.-.-	Acting on paired donors, with incorporation or redu	NF	NF	NF	NF
3509	1.14.13.-	With NADH or NADPH as one donor, and incorporat	NF	NF	NF	NF
3510	1.14.13.105	Monocyclic monoterpene ketone monooxygenase.	NF	NF	NF	NF
3511	1.14.13.107	Limonene 1,2-monooxygenase.	NF	NF	NF	NF
3512	1.14.13.48	(S)-limonene 6-monooxygenase.	NF	NF	NF	NF
3513	1.14.13.49	(S)-limonene 7-monooxygenase.	NF	NF	NF	NF
3514	1.14.13.80	(R)-limonene 6-monooxygenase.	NF	NF	NF	NF

3515	1.2.-.-	Acting on the aldehyde or oxo group of donors.	FOUND	FOUND	FOUND	FOUND
3516	1.2.1.-	With NAD(+) or NADP(+) as acceptor.	FOUND	FOUND	FOUND	FOUND
3517	1.2.1.3	Aldehyde dehydrogenase (NAD(+)).	NF	NF	FOUND	FOUND
3518	1.3.99.25	Carvone reductase.	NF	NF	NF	NF
3519	2.3.1.-	Transferring groups other than amino-acyl groups.	FOUND	FOUND	FOUND	FOUND
3520	3.1.1.83	Monoterpene epsilon-lactone hydrolase.	NF	NF	NF	NF
3521	3.1.2.-	Thiolester hydrolases.	NF	FOUND	NF	NF
3522	3.3.2.8	Limonene-1,2-epoxide hydrolase.	NF	NF	NF	NF
3523	3.7.1.-	In ketonic substances.	NF	NF	NF	FOUND
3524	4.2.1.-	Hydro-lyases.	FOUND	FOUND	FOUND	FOUND
3525	4.2.1.17	Enoyl-CoA hydratase.	NF	NF	NF	NF
3526	5.5.1.10	Alpha-pinene-oxide decyclase.	NF	NF	NF	NF
3527	6.2.1.-	Acid--thiol ligases.	NF	NF	NF	NF
3528	>Diterpenoid biosynthesis 00904					
3529	1.1.1.295	Momilactone-A synthase.	NF	NF	NF	NF
3530	1.14.-.-	Acting on paired donors, with incorporation or redu	NF	NF	NF	NF
3531	1.14.11.-	With 2-oxoglutarate as one donor, and incorporatio	NF	NF	NF	NF
3532	1.14.11.12	Gibberellin-44 dioxygenase.	NF	NF	NF	NF
3533	1.14.11.13	Gibberellin 2-beta-dioxygenase.	NF	NF	NF	NF
3534	1.14.11.15	Gibberellin 3-beta-dioxygenase.	NF	NF	NF	NF
3535	1.14.13.-	With NADH or NADPH as one donor, and incorporat	NF	NF	NF	NF
3536	1.14.13.108	Abietadiene hydroxylase.	NF	NF	NF	NF
3537	1.14.13.109	Abietadienol hydroxylase.	NF	NF	NF	NF
3538	1.14.13.110	Geranylgeraniol 18-hydroxylase.	NF	NF	NF	NF
3539	1.14.13.76	Taxane 10-beta-hydroxylase.	NF	NF	NF	NF
3540	1.14.13.77	Taxane 13-alpha-hydroxylase.	NF	NF	NF	NF
3541	1.14.13.78	Ent-kaurene oxidase.	NF	NF	NF	NF
3542	1.14.13.79	Ent-kaurenoic acid oxidase.	NF	NF	NF	NF
3543	1.14.99.37	Taxadiene 5-alpha-hydroxylase.	NF	NF	NF	NF
3544	1.2.1.74	Abietadienal dehydrogenase.	NF	NF	NF	NF
3545	2.3.1.-	Transferring groups other than amino-acyl groups.	FOUND	FOUND	FOUND	FOUND
3546	2.3.1.162	Taxadien-5-alpha-ol O-acetyltransferase.	NF	NF	NF	NF
3547	2.3.1.166	2-alpha-hydroxytaxane 2-O-benzoyltransferase.	NF	NF	NF	NF
3548	2.3.1.167	10-deacetylbaccatin III 10-O-acetyltransferase.	NF	NF	NF	NF
3549	2.3.2.-	Aminoacyltransferases.	NF	NF	NF	NF
3550	3.1.7.4	Sclareol cyclase.	NF	NF	NF	NF
3551	3.1.7.5	Geranylgeranyl diphosphate diphosphatase.	NF	NF	NF	NF
3552	4.2.3.17	Taxadiene synthase.	NF	NF	NF	NF
3553	4.2.3.18	Abietadiene synthase.	NF	NF	NF	NF
3554	4.2.3.19	Ent-kaurene synthase.	NF	NF	NF	NF
3555	4.2.3.28	Ent-cassa-12,15-diene synthase.	NF	NF	NF	NF
3556	4.2.3.29	Ent-sandaracopimaradiene synthase.	NF	NF	NF	NF
3557	4.2.3.30	Ent-pimara-8(14),15-diene synthase.	NF	NF	NF	NF
3558	4.2.3.32	Levopimaradiene synthase.	NF	NF	NF	NF
3559	4.2.3.33	Stemar-13-ene synthase.	NF	NF	NF	NF
3560	4.2.3.34	Stemod-13(17)-ene synthase.	NF	NF	NF	NF
3561	4.2.3.35	Syn-pimara-7,15-diene synthase.	NF	NF	NF	NF
3562	4.2.3.42	Aphidicolan-16-beta-ol synthase.	NF	NF	NF	NF
3563	4.2.3.43	Fusicocca-2,10(14)-diene synthase.	NF	NF	NF	NF
3564	4.2.3.44	Isopimara-7,15-diene synthase.	NF	NF	NF	NF
3565	4.2.3.8	Casbene synthase.	NF	NF	NF	NF
3566	5.5.1.12	Copalyl diphosphate synthase.	NF	NF	NF	NF
3567	5.5.1.13	Ent-copalyl diphosphate synthase.	NF	NF	NF	NF
3568	5.5.1.14	Syn-copalyl-diphosphate synthase.	NF	NF	NF	NF
3569	>Brassinosteroid biosynthesis 00905					

3570	1.14.-.-	Acting on paired donors, with incorporation or redu	NF	NF	NF	NF
3571	1.14.13.-	With NADH or NADPH as one donor, and incorporat	NF	NF	NF	NF
3572	1.14.13.112	3-epi-6-deoxocathasterone 23-monooxygenase.	NF	NF	NF	NF
3573	1.3.99.-	With other acceptors.	NF	NF	FOUND	FOUND
3574	>Carotenoid biosynthesis 00906					
3575	1.-.-.-	Oxidoreductases.	FOUND	FOUND	FOUND	FOUND
3576	1.1.1.288	Xanthoxin dehydrogenase.	NF	NF	NF	NF
3577	1.10.99.3	Violaxanthin de-epoxidase.	NF	NF	NF	NF
3578	1.13.11.51	9-cis-epoxycarotenoid dioxygenase.	NF	NF	NF	NF
3579	1.14.-.-	Acting on paired donors, with incorporation or redu	NF	NF	NF	NF
3580	1.14.13.-	With NADH or NADPH as one donor, and incorporat	NF	NF	NF	NF
3581	1.14.13.90	Zeaxanthin epoxidase.	NF	NF	NF	NF
3582	1.14.13.93	(+)-abscisic acid 8'-hydroxylase.	NF	NF	NF	NF
3583	1.14.99.-	Miscellaneous (requires further characterization).	NF	NF	NF	NF
3584	1.14.99.30	Carotene 7,8-desaturase.	NF	NF	NF	NF
3585	1.2.3.14	Abscisic-aldehyde oxidase.	NF	NF	NF	NF
3586	2.1.1.-	Methyltransferases.	FOUND	FOUND	FOUND	FOUND
3587	2.3.1.-	Transferring groups other than amino-acyl groups.	FOUND	FOUND	FOUND	FOUND
3588	2.4.1.-	Hexosyltransferases.	FOUND	FOUND	FOUND	FOUND
3589	2.5.1.-	Transferring alkyl or aryl groups, other than methyl	FOUND	FOUND	FOUND	FOUND
3590	2.5.1.32	Phytoene synthase.	NF	NF	NF	NF
3591	5.-.-.-	Isomerases.	FOUND	FOUND	FOUND	FOUND
3592	5.3.99.8	Capsanthin/capsorubin synthase.	NF	NF	NF	NF
3593	5.3.99.9	Neoxanthin synthase.	NF	NF	NF	NF
3594	>Zeatin biosynthesis 00908					
3595	1.3.1.69	Zeatin reductase.	NF	NF	NF	NF
3596	1.5.99.12	Cytokinin dehydrogenase.	NF	NF	NF	NF
3597	2.4.1.-	Hexosyltransferases.	FOUND	FOUND	FOUND	FOUND
3598	2.4.1.203	Trans-zeatin O-beta-D-glucosyltransferase.	NF	NF	NF	NF
3599	2.4.1.215	Cis-zeatin O-beta-D-glucosyltransferase.	NF	NF	NF	NF
3600	2.4.2.40	Zeatin O-beta-D-xylosyltransferase.	NF	NF	NF	NF
3601	2.5.1.24	Discadenine synthase.	NF	NF	NF	NF
3602	2.5.1.27	Adenylate dimethylallyltransferase.	NF	NF	NF	NF
3603	2.5.1.50	Zeatin 9-aminocarboxyethyltransferase.	NF	NF	NF	NF
3604	2.5.1.75	tRNA dimethylallyltransferase.	FOUND	NF	NF	NF
3605	>Sesquiterpenoid biosynthesis 00909					
3606	1.1.1.216	Farnesol dehydrogenase.	NF	NF	NF	NF
3607	1.14.13.106	Epi-isozizaene 5-monooxygenase.	NF	NF	NF	NF
3608	3.1.7.-	Diphosphoric monoester hydrolases.	NF	NF	NF	NF
3609	4.2.3.-	Acting on phosphates.	NF	NF	FOUND	FOUND
3610	4.2.3.13	(+)-delta-cadinene synthase.	NF	NF	NF	NF
3611	4.2.3.21	Vetispiradiene synthase.	NF	NF	NF	NF
3612	4.2.3.22	Germacradienol synthase.	NF	NF	NF	NF
3613	4.2.3.23	Germacrene-A synthase.	NF	NF	NF	NF
3614	4.2.3.24	Amorpha-4,11-diene synthase.	NF	NF	NF	NF
3615	4.2.3.37	Epi-isozizaene synthase.	NF	NF	NF	NF
3616	4.2.3.38	Alpha-bisabolene synthase.	NF	NF	NF	NF
3617	4.2.3.39	Epi-cedrol synthase.	NF	NF	NF	NF
3618	4.2.3.46	Alpha-farnesene synthase.	NF	NF	NF	NF
3619	4.2.3.47	Beta-farnesene synthase.	NF	NF	NF	NF
3620	4.2.3.6	Trichodiene synthase.	NF	NF	NF	NF
3621	4.2.3.7	Pentalenene synthase.	NF	NF	NF	NF
3622	4.2.3.9	Aristolochene synthase.	NF	NF	NF	NF
3623	5.2.1.9	Farnesol 2-isomerase.	NF	NF	NF	NF
3624	>Nitrogen metabolism 00910					

3625	1.13.12.-	With incorporation of one atom of oxygen.	NF	NF	FOUND	NF
3626	1.13.12.16	Nitronate monooxygenase.	NF	NF	FOUND	NF
3627	1.14.12.1	Anthranilate 1,2-dioxygenase (deaminating, decarb	NF	NF	NF	NF
3628	1.14.13.35	Anthranilate 3-monooxygenase (deaminating).	NF	NF	NF	NF
3629	1.18.6.1	Nitrogenase.	NF	NF	NF	NF
3630	1.19.6.1	Nitrogenase (flavodoxin).	NF	NF	NF	NF
3631	1.4.1.13	Glutamate synthase (NADPH).	NF	NF	NF	FOUND
3632	1.4.1.14	Glutamate synthase (NADH).	NF	NF	NF	FOUND
3633	1.4.1.2	Glutamate dehydrogenase.	NF	NF	FOUND	NF
3634	1.4.1.3	Glutamate dehydrogenase (NAD(P)(+)).	NF	NF	NF	NF
3635	1.4.1.4	Glutamate dehydrogenase (NADP(+)).	NF	FOUND	NF	FOUND
3636	1.4.2.-	With a cytochrome as acceptor.	NF	NF	NF	NF
3637	1.4.7.1	Glutamate synthase (ferredoxin).	NF	NF	NF	FOUND
3638	1.4.99.1	D-amino-acid dehydrogenase.	NF	NF	FOUND	NF
3639	1.7.1.1	Nitrate reductase (NADH).	NF	NF	NF	NF
3640	1.7.1.10	Hydroxylamine reductase (NADH).	NF	NF	NF	NF
3641	1.7.1.2	Nitrate reductase (NAD(P)H).	NF	NF	NF	NF
3642	1.7.1.3	Nitrate reductase (NADPH).	NF	NF	NF	NF
3643	1.7.1.4	Nitrite reductase (NAD(P)H).	NF	NF	NF	NF
3644	1.7.2.1	Nitrite reductase (NO-forming).	NF	NF	NF	NF
3645	1.7.2.2	Nitrite reductase (cytochrome; ammonia-forming).	NF	NF	NF	NF
3646	1.7.3.1	Nitroalkane oxidase.	NF	NF	NF	NF
3647	1.7.3.4	Hydroxylamine oxidase.	NF	NF	NF	NF
3648	1.7.7.1	Ferredoxin--nitrite reductase.	NF	NF	NF	NF
3649	1.7.7.2	Ferredoxin--nitrate reductase.	NF	NF	NF	NF
3650	1.7.99.1	Hydroxylamine reductase.	NF	NF	NF	NF
3651	1.7.99.4	Nitrate reductase.	NF	NF	NF	NF
3652	1.7.99.6	Nitrous-oxide reductase.	NF	NF	NF	NF
3653	1.7.99.7	Nitric-oxide reductase.	NF	NF	NF	NF
3654	1.9.6.1	Nitrate reductase (cytochrome).	NF	NF	NF	NF
3655	2.1.2.10	Aminomethyltransferase.	NF	NF	NF	NF
3656	2.7.2.2	Carbamate kinase.	FOUND	FOUND	NF	FOUND
3657	3.5.1.1	Asparaginase.	NF	NF	NF	NF
3658	3.5.1.2	Glutaminase.	NF	NF	NF	NF
3659	3.5.1.38	Glutamin-(asparagin-)ase.	NF	NF	NF	NF
3660	3.5.1.49	Formamidase.	NF	NF	NF	NF
3661	3.5.5.1	Nitrilase.	NF	NF	NF	NF
3662	3.5.5.2	Ricinine nitrilase.	NF	NF	NF	NF
3663	4.1.99.1	Tryptophanase.	NF	NF	NF	NF
3664	4.1.99.2	Tyrosine phenol-lyase.	NF	NF	NF	NF
3665	4.2.1.1	Carbonate dehydratase.	NF	NF	FOUND	FOUND
3666	4.2.1.104	Cyanase.	NF	NF	NF	NF
3667	4.3.1.1	Aspartate ammonia-lyase.	NF	NF	NF	NF
3668	4.3.1.2	Methylaspartate ammonia-lyase.	NF	NF	NF	NF
3669	4.3.1.23	Tyrosine ammonia-lyase.	NF	NF	NF	NF
3670	4.3.1.24	Phenylalanine ammonia-lyase.	NF	NF	NF	NF
3671	4.3.1.25	Phenylalanine/tyrosine ammonia-lyase.	NF	NF	NF	NF
3672	4.3.1.3	Histidine ammonia-lyase.	NF	NF	NF	NF
3673	4.4.1.1	Cystathionine gamma-lyase.	NF	NF	FOUND	NF
3674	4.4.1.2	Homocysteine desulfhydrase.	NF	NF	NF	NF
3675	4.4.1.8	Cystathionine beta-lyase.	FOUND	NF	FOUND	NF
3676	6.3.1.1	Aspartate--ammonia ligase.	FOUND	FOUND	FOUND	FOUND
3677	6.3.1.2	Glutamate--ammonia ligase.	NF	FOUND	FOUND	FOUND
3678	6.3.1.4	Aspartate--ammonia ligase (ADP-forming).	NF	NF	NF	NF
3679	6.3.1.5	NAD(+) synthase.	NF	NF	NF	FOUND

3680	6.3.4.16	Carbamoyl-phosphate synthase (ammonia).	NF	NF	NF	NF
3681	6.3.5.4	Asparagine synthase (glutamine-hydrolyzing).	NF	NF	NF	FOUND
3682	>Sulfur metabolism 00920					
3683	1.13.11.18	Sulfur dioxygenase.	NF	NF	NF	NF
3684	1.8.1.2	Sulfite reductase (NADPH).	NF	NF	NF	NF
3685	1.8.2.1	Sulfite dehydrogenase.	NF	NF	NF	NF
3686	1.8.3.1	Sulfite oxidase.	NF	NF	NF	NF
3687	1.8.4.8	Phosphoadenylyl-sulfate reductase (thioredoxin).	NF	NF	NF	NF
3688	1.8.7.1	Sulfite reductase (ferredoxin).	NF	NF	NF	NF
3689	1.8.99.1	Sulfite reductase.	NF	NF	NF	NF
3690	1.8.99.2	Adenylyl-sulfate reductase.	NF	NF	FOUND	NF
3691	2.3.1.30	Serine O-acetyltransferase.	NF	NF	FOUND	FOUND
3692	2.3.1.31	Homoserine O-acetyltransferase.	NF	NF	NF	NF
3693	2.3.1.46	Homoserine O-succinyltransferase.	NF	NF	FOUND	FOUND
3694	2.5.1.47	Cysteine synthase.	NF	NF	FOUND	FOUND
3695	2.5.1.48	Cystathionine gamma-synthase.	NF	NF	FOUND	NF
3696	2.5.1.65	O-phosphoserine sulfhydrylase.	NF	NF	NF	NF
3697	2.7.1.25	Adenylyl-sulfate kinase.	NF	NF	NF	NF
3698	2.7.7.4	Sulfate adenylyltransferase.	NF	NF	FOUND	NF
3699	2.7.7.5	Sulfate adenylyltransferase (ADP).	NF	NF	NF	NF
3700	2.8.1.5	Thiosulfate--dithiol sulfurtransferase.	NF	NF	NF	NF
3701	2.8.2.1	Aryl sulfotransferase.	NF	NF	NF	NF
3702	2.8.2.2	Alcohol sulfotransferase.	NF	NF	NF	NF
3703	2.8.2.3	Amine sulfotransferase.	NF	NF	NF	NF
3704	2.8.2.4	Estrone sulfotransferase.	NF	NF	NF	NF
3705	2.8.2.5	Chondroitin 4-sulfotransferase.	NF	NF	NF	NF
3706	2.8.2.6	Choline sulfotransferase.	NF	NF	NF	NF
3707	3.1.3.7	3'(2'),5'-bisphosphate nucleotidase.	NF	NF	NF	NF
3708	3.12.1.1	Trithionate hydrolase.	NF	NF	NF	NF
3709	3.6.2.1	Adenylylsulfatase.	NF	NF	NF	NF
3710	3.6.2.2	Phosphoadenylylsulfatase.	NF	NF	NF	NF
3711	4.4.1.2	Homocysteine desulfhydrase.	NF	NF	NF	NF
3712	4.4.1.8	Cystathionine beta-lyase.	FOUND	NF	FOUND	NF
3713	>Caprolactam degradation 00930					
3714	1.1.1.174	Cyclohexane-1,2-diol dehydrogenase.	NF	NF	NF	NF
3715	1.1.1.2	Alcohol dehydrogenase (NADP(+)).	NF	NF	FOUND	NF
3716	1.1.1.245	Cyclohexanol dehydrogenase.	NF	NF	NF	NF
3717	1.1.1.258	6-hydroxyhexanoate dehydrogenase.	NF	NF	NF	NF
3718	1.1.1.35	3-hydroxyacyl-CoA dehydrogenase.	NF	NF	NF	NF
3719	1.1.1.90	Aryl-alcohol dehydrogenase.	NF	NF	NF	NF
3720	1.14.13.22	Cyclohexanone monooxygenase.	NF	NF	NF	NF
3721	1.14.15.-	With a reduced iron-sulfur protein as one donor, an	NF	NF	NF	NF
3722	1.2.1.4	Aldehyde dehydrogenase (NADP(+)).	NF	NF	NF	NF
3723	1.2.1.63	6-oxohexanoate dehydrogenase.	NF	NF	NF	NF
3724	1.3.99.-	With other acceptors.	NF	NF	FOUND	FOUND
3725	1.4.3.12	Cyclohexylamine oxidase.	NF	NF	NF	NF
3726	2.6.1.-	Transaminases (aminotransferases).	FOUND	FOUND	FOUND	FOUND
3727	3.1.1.17	Gluconolactonase.	NF	NF	NF	NF
3728	3.10.1.2	Cyclamate sulfohydrolase.	NF	NF	NF	NF
3729	3.5.1.-	In linear amides.	FOUND	FOUND	FOUND	FOUND
3730	3.5.2.-	In cyclic amides.	NF	NF	FOUND	FOUND
3731	3.7.1.11	Cyclohexane-1,2-dione hydrolase.	NF	NF	NF	NF
3732	4.2.1.103	Cyclohexyl-isocyanide hydratase.	NF	NF	NF	NF
3733	4.2.1.17	Enoyl-CoA hydratase.	NF	NF	NF	NF
3734	6.2.1.-	Acid--thiol ligases.	NF	NF	NF	NF

3735	>Phenylpropanoid biosynthesis 00940					
3736	1.1.1.195	Cinnamyl-alcohol dehydrogenase.	NF	NF	NF	NF
3737	1.11.1.7	Peroxidase.	FOUND	FOUND	FOUND	FOUND
3738	1.13.11.22	Caffeate 3,4-dioxygenase.	NF	NF	NF	NF
3739	1.14.-.-	Acting on paired donors, with incorporation or redu	NF	NF	NF	NF
3740	1.14.13.-	With NADH or NADPH as one donor, and incorporat	NF	NF	NF	NF
3741	1.14.13.11	Trans-cinnamate 4-monooxygenase.	NF	NF	NF	NF
3742	1.14.13.14	Trans-cinnamate 2-monooxygenase.	NF	NF	NF	NF
3743	1.14.13.36	5-O-(4-coumaroyl)-D-quinate 3'-monooxygenase.	NF	NF	NF	NF
3744	1.2.1.44	Cinnamoyl-CoA reductase.	NF	NF	NF	NF
3745	1.2.1.68	Coniferyl-aldehyde dehydrogenase.	NF	NF	NF	NF
3746	2.1.1.-	Methyltransferases.	FOUND	FOUND	FOUND	FOUND
3747	2.1.1.104	Caffeoyl-CoA O-methyltransferase.	NF	NF	NF	NF
3748	2.1.1.68	Caffeate O-methyltransferase.	NF	NF	NF	NF
3749	2.3.1.133	Shikimate O-hydroxycinnamoyltransferase.	NF	NF	NF	NF
3750	2.3.1.91	Sinapoylglucose--choline O-sinapoyltransferase.	NF	NF	NF	NF
3751	2.3.1.92	Sinapoylglucose--malate O-sinapoyltransferase.	NF	NF	NF	NF
3752	2.3.1.99	Quinate O-hydroxycinnamoyltransferase.	NF	NF	NF	NF
3753	2.4.1.111	Coniferyl-alcohol glucosyltransferase.	NF	NF	NF	NF
3754	2.4.1.114	2-coumarate O-beta-glucosyltransferase.	NF	NF	NF	NF
3755	2.4.1.120	Sinapate 1-glucosyltransferase.	NF	NF	NF	NF
3756	2.4.1.128	Scopoletin glucosyltransferase.	NF	NF	NF	NF
3757	3.1.1.49	Sinapine esterase.	NF	NF	NF	NF
3758	3.2.1.126	Coniferin beta-glucosidase.	NF	NF	NF	NF
3759	3.2.1.21	Beta-glucosidase.	NF	NF	NF	NF
3760	4.1.1.-	Carboxy-lyases.	FOUND	FOUND	FOUND	FOUND
3761	4.3.1.23	Tyrosine ammonia-lyase.	NF	NF	NF	NF
3762	4.3.1.24	Phenylalanine ammonia-lyase.	NF	NF	NF	NF
3763	4.3.1.25	Phenylalanine/tyrosine ammonia-lyase.	NF	NF	NF	NF
3764	5.2.1.-	Cis-trans Isomerases.	FOUND	FOUND	FOUND	FOUND
3765	6.2.1.12	4-coumarate--CoA ligase.	NF	NF	NF	NF
3766	>Flavonoid biosynthesis 00941					
3767	1.1.1.219	Dihydrokaempferol 4-reductase.	NF	NF	NF	NF
3768	1.1.1.234	Flavanone 4-reductase.	NF	NF	NF	NF
3769	1.14.11.19	Leucocyanidin oxygenase.	NF	NF	NF	NF
3770	1.14.11.22	Flavone synthase.	NF	NF	NF	NF
3771	1.14.11.23	Flavonol synthase.	NF	NF	NF	NF
3772	1.14.11.9	Flavanone 3-dioxygenase.	NF	NF	NF	NF
3773	1.14.13.-	With NADH or NADPH as one donor, and incorporat	NF	NF	NF	NF
3774	1.14.13.11	Trans-cinnamate 4-monooxygenase.	NF	NF	NF	NF
3775	1.14.13.21	Flavonoid 3'-monooxygenase.	NF	NF	NF	NF
3776	1.14.13.36	5-O-(4-coumaroyl)-D-quinate 3'-monooxygenase.	NF	NF	NF	NF
3777	1.14.13.88	Flavonoid 3',5'-hydroxylase.	NF	NF	NF	NF
3778	1.17.1.3	Leucoanthocyanidin reductase.	NF	NF	NF	NF
3779	1.21.3.6	Aureusidin synthase.	NF	NF	NF	NF
3780	1.3.1.77	Anthocyanidin reductase.	NF	NF	NF	NF
3781	2.1.1.-	Methyltransferases.	FOUND	FOUND	FOUND	FOUND
3782	2.1.1.104	Caffeoyl-CoA O-methyltransferase.	NF	NF	NF	NF
3783	2.3.1.133	Shikimate O-hydroxycinnamoyltransferase.	NF	NF	NF	NF
3784	2.3.1.170	6'-deoxychalcone synthase.	NF	NF	NF	NF
3785	2.3.1.74	Naringenin-chalcone synthase.	NF	NF	NF	NF
3786	2.4.1.-	Hexosyltransferases.	FOUND	FOUND	FOUND	FOUND
3787	2.4.1.185	Flavanone 7-O-beta-glucosyltransferase.	NF	NF	NF	NF
3788	2.4.1.236	Flavanone 7-O-glucoside 2''-O-beta-L-rhamnosyltrar	NF	NF	NF	NF
3789	5.5.1.6	Chalcone isomerase.	NF	NF	NF	NF

3790	>Anthocyanin biosynthesis 00942					
3791	2.1.1.-	Methyltransferases.	FOUND	FOUND	FOUND	FOUND
3792	2.3.1.-	Transferring groups other than amino-acyl groups.	FOUND	FOUND	FOUND	FOUND
3793	2.3.1.153	Anthocyanin 5-aromatic acyltransferase.	NF	NF	NF	NF
3794	2.3.1.171	Anthocyanin 6''-O-malonyltransferase.	NF	NF	NF	NF
3795	2.3.1.172	Anthocyanin 5-O-glucoside 6'''-O-malonyltransferas	NF	NF	NF	NF
3796	2.4.1.-	Hexosyltransferases.	FOUND	FOUND	FOUND	FOUND
3797	2.4.1.115	Anthocyanidin 3-O-glucosyltransferase.	NF	NF	NF	NF
3798	2.4.1.116	Cyanidin 3-O-rutinoside 5-O-glucosyltransferase.	NF	NF	NF	NF
3799	2.4.1.238	Anthocyanin 3'-O-beta-glucosyltransferase.	NF	NF	NF	NF
3800	>Isoflavonoid biosynthesis 00943					
3801	1.1.1.246	Pterocarpin synthase.	NF	NF	NF	NF
3802	1.14.11.22	Flavone synthase.	NF	NF	NF	NF
3803	1.14.13.-	With NADH or NADPH as one donor, and incorporat	NF	NF	NF	NF
3804	1.14.13.28	3,9-dihydroxypterocarpan 6A-monooxygenase.	NF	NF	NF	NF
3805	1.14.13.52	Isoflavone 3'-hydroxylase.	NF	NF	NF	NF
3806	1.14.13.85	Glyceollin synthase.	NF	NF	NF	NF
3807	1.14.13.86	2-hydroxyisoflavanone synthase.	NF	NF	NF	NF
3808	1.14.13.89	Isoflavone 2'-hydroxylase.	NF	NF	NF	NF
3809	1.3.1.-	With NAD(+) or NADP(+) as acceptor.	FOUND	FOUND	FOUND	FOUND
3810	1.3.1.45	2'-hydroxyisoflavone reductase.	NF	NF	NF	NF
3811	1.3.1.46	Biochanin-A reductase.	NF	NF	NF	NF
3812	1.3.1.51	2'-hydroxydaidzein reductase.	NF	NF	NF	NF
3813	2.1.1.150	Isoflavone 7-O-methyltransferase.	NF	NF	NF	NF
3814	2.1.1.46	Isoflavone 4'-O-methyltransferase.	NF	NF	NF	NF
3815	2.3.1.115	Isoflavone-7-O-beta-glucoside 6''-O-malonyltransfer	NF	NF	NF	NF
3816	2.4.1.170	Isoflavone 7-O-glucosyltransferase.	NF	NF	NF	NF
3817	2.5.1.36	Trihydroxypterocarpan dimethylallyltransferase.	NF	NF	NF	NF
3818	4.2.1.105	2-hydroxyisoflavanone dehydratase.	NF	NF	NF	NF
3819	>Flavone and flavonol biosynthesis 00944					
3820	1.14.13.21	Flavonoid 3'-monooxygenase.	NF	NF	NF	NF
3821	1.14.13.88	Flavonoid 3',5'-hydroxylase.	NF	NF	NF	NF
3822	2.1.1.149	Myricetin O-methyltransferase.	NF	NF	NF	NF
3823	2.1.1.75	Apigenin 4'-O-methyltransferase.	NF	NF	NF	NF
3824	2.1.1.76	Quercetin 3-O-methyltransferase.	NF	NF	NF	NF
3825	2.1.1.82	3-methylquercetin 7-O-methyltransferase.	NF	NF	NF	NF
3826	2.1.1.83	3,7-dimethylquercetin 4'-O-methyltransferase.	NF	NF	NF	NF
3827	2.3.1.115	Isoflavone-7-O-beta-glucoside 6''-O-malonyltransfer	NF	NF	NF	NF
3828	2.3.1.116	Flavonol-3-O-beta-glucoside O-malonyltransferase.	NF	NF	NF	NF
3829	2.4.1.-	Hexosyltransferases.	FOUND	FOUND	FOUND	FOUND
3830	2.4.1.159	Flavonol-3-O-glucoside L-rhamnosyltransferase.	NF	NF	NF	NF
3831	2.4.1.189	Luteolin 7-O-glucuronosyltransferase.	NF	NF	NF	NF
3832	2.4.1.190	Luteolin-7-O-glucuronide 2''-O-glucuronosyltransfer	NF	NF	NF	NF
3833	2.4.1.191	Luteolin-7-O-diglucuronide 4'-O-glucuronosyltransfe	NF	NF	NF	NF
3834	2.4.1.234	Kaempferol 3-O-galactosyltransferase.	NF	NF	NF	NF
3835	2.4.1.239	Flavonol-3-O-glucoside glucosyltransferase.	NF	NF	NF	NF
3836	2.4.1.240	Flavonol-3-O-glycoside glucosyltransferase.	NF	NF	NF	NF
3837	2.4.1.81	Flavone 7-O-beta-glucosyltransferase.	NF	NF	NF	NF
3838	2.4.1.91	Flavonol 3-O-glucosyltransferase.	NF	NF	NF	NF
3839	2.4.2.25	Flavone apiosyltransferase.	NF	NF	NF	NF
3840	2.8.2.25	Flavonol 3-sulfotransferase.	NF	NF	NF	NF
3841	2.8.2.26	Quercetin-3-sulfate 3'-sulfotransferase.	NF	NF	NF	NF
3842	2.8.2.27	Quercetin-3-sulfate 4'-sulfotransferase.	NF	NF	NF	NF
3843	2.8.2.28	Quercetin-3,3'-bissulfate 7-sulfotransferase.	NF	NF	NF	NF
3844	3.2.1.31	Beta-glucuronidase.	NF	NF	NF	NF

3845	>Stilbenoid, diarylheptanoid and gingerol biosynthesis 00945					
3846	1.14.-.-	Acting on paired donors, with incorporation or redu	NF	NF	NF	NF
3847	1.14.13.-	With NADH or NADPH as one donor, and incorporat	NF	NF	NF	NF
3848	1.14.13.11	Trans-cinnamate 4-monooxygenase.	NF	NF	NF	NF
3849	1.14.13.36	5-O-(4-coumaroyl)-D-quinate 3'-monooxygenase.	NF	NF	NF	NF
3850	2.1.1.-	Methyltransferases.	FOUND	FOUND	FOUND	FOUND
3851	2.1.1.104	Caffeoyl-CoA O-methyltransferase.	NF	NF	NF	NF
3852	2.3.1.-	Transferring groups other than amino-acyl groups.	FOUND	FOUND	FOUND	FOUND
3853	2.3.1.133	Shikimate O-hydroxycinnamoyltransferase.	NF	NF	NF	NF
3854	2.3.1.146	Pinosylvin synthase.	NF	NF	NF	NF
3855	2.3.1.95	Trihydroxystilbene synthase.	NF	NF	NF	NF
3856	2.3.1.99	Quinate O-hydroxycinnamoyltransferase.	NF	NF	NF	NF
3857	2.4.1.-	Hexosyltransferases.	FOUND	FOUND	FOUND	FOUND
3858	>Isoquinoline alkaloid biosynthesis 00950					
3859	1.-.-.-	Oxidoreductases.	FOUND	FOUND	FOUND	FOUND
3860	1.1.1.-	With NAD(+) or NADP(+) as acceptor.	FOUND	FOUND	FOUND	FOUND
3861	1.1.1.218	Morphine 6-dehydrogenase.	NF	NF	NF	NF
3862	1.1.1.247	Codeinone reductase (NADPH).	NF	NF	NF	NF
3863	1.1.1.248	Salutaridine reductase (NADPH).	NF	NF	NF	NF
3864	1.10.3.1	Catechol oxidase.	NF	NF	NF	NF
3865	1.14.13.-	With NADH or NADPH as one donor, and incorporat	NF	NF	NF	NF
3866	1.14.13.37	Methyltetrahydroprotoberberine 14-monooxygena	NF	NF	NF	NF
3867	1.14.13.55	Protopine 6-monooxygenase.	NF	NF	NF	NF
3868	1.14.13.56	Dihydrosanguinarine 10-monooxygenase.	NF	NF	NF	NF
3869	1.14.13.57	Dihydrochelirubine 12-monooxygenase.	NF	NF	NF	NF
3870	1.14.13.71	N-methylcoclaurine 3'-monooxygenase.	NF	NF	NF	NF
3871	1.14.16.2	Tyrosine 3-monooxygenase.	NF	NF	NF	NF
3872	1.14.18.1	Monophenol monooxygenase.	NF	NF	NF	NF
3873	1.14.21.-	With NADH or NADPH as one donor, and the other	NF	NF	NF	NF
3874	1.14.21.1	(S)-stylopine synthase.	NF	NF	NF	NF
3875	1.14.21.2	(S)-cheilanthifoline synthase.	NF	NF	NF	NF
3876	1.14.21.3	Berbamunine synthase.	NF	NF	NF	NF
3877	1.14.21.4	Salutaridine synthase.	NF	NF	NF	NF
3878	1.14.21.5	(S)-canadine synthase.	NF	NF	NF	NF
3879	1.21.3.2	Columbamine oxidase.	NF	NF	NF	NF
3880	1.21.3.3	Reticuline oxidase.	NF	NF	NF	NF
3881	1.3.3.8	Tetrahydroberberine oxidase.	NF	NF	NF	NF
3882	1.4.3.2	L-amino-acid oxidase.	NF	NF	NF	NF
3883	1.4.3.21	Primary-amine oxidase.	NF	NF	FOUND	FOUND
3884	1.4.3.4	Monoamine oxidase.	NF	NF	NF	NF
3885	1.4.99.4	Aralkylamine dehydrogenase.	NF	NF	NF	NF
3886	1.5.-.-	Acting on the CH-NH group of donors.	FOUND	FOUND	FOUND	FOUND
3887	1.5.1.27	1,2-dehydroreticulinium reductase (NADPH).	NF	NF	NF	NF
3888	1.5.1.31	Berberine reductase.	NF	NF	NF	NF
3889	1.5.3.12	Dihydrobenzophenanthridine oxidase.	NF	NF	NF	NF
3890	2.1.1.-	Methyltransferases.	FOUND	FOUND	FOUND	FOUND
3891	2.1.1.115	(RS)-1-benzyl-1,2,3,4-tetrahydroisoquinoline N-met	NF	NF	NF	NF
3892	2.1.1.116	3'-hydroxy-N-methyl-(S)-coclaurine 4'-O-methyltran	NF	NF	NF	NF
3893	2.1.1.117	(S)-scoulerine 9-O-methyltransferase.	NF	NF	NF	NF
3894	2.1.1.118	Columbamine O-methyltransferase.	NF	NF	NF	NF
3895	2.1.1.119	10-hydroxydihydrosanguinarine 10-O-methyltransfe	NF	NF	NF	NF
3896	2.1.1.120	12-hydroxydihydrochelirubine 12-O-methyltransfer	NF	NF	NF	NF
3897	2.1.1.121	6-O-methylnorlaudanosoline 5'-O-methyltransferas	NF	NF	NF	NF
3898	2.1.1.122	(S)-tetrahydroprotoberberine N-methyltransferase.	NF	NF	NF	NF
3899	2.1.1.128	(RS)-norcoclaurine 6-O-methyltransferase.	NF	NF	NF	NF

3900	2.1.1.140	(S)-coclaurine-N-methyltransferase.	NF	NF	NF	NF
3901	2.1.1.147	Corydaline synthase.	NF	NF	NF	NF
3902	2.1.1.89	Tetrahydrocolumbamine 2-O-methyltransferase.	NF	NF	NF	NF
3903	2.3.1.-	Transferring groups other than amino-acyl groups.	FOUND	FOUND	FOUND	FOUND
3904	2.3.1.150	Salutaridinol 7-O-acetyltransferase.	NF	NF	NF	NF
3905	2.6.1.1	Aspartate transaminase.	FOUND	FOUND	FOUND	FOUND
3906	2.6.1.5	Tyrosine transaminase.	NF	NF	NF	NF
3907	2.6.1.57	Aromatic-amino-acid transaminase.	NF	NF	NF	NF
3908	4.1.1.25	Tyrosine decarboxylase.	NF	NF	NF	NF
3909	4.1.1.28	Aromatic-L-amino-acid decarboxylase.	NF	NF	NF	NF
3910	4.1.1.80	4-hydroxyphenylpyruvate decarboxylase.	NF	NF	NF	NF
3911	4.2.1.78	(S)-norcoclaurine synthase.	NF	NF	NF	NF
3912	4.3.3.3	Deacetylisoipecoside synthase.	NF	NF	NF	NF
3913	4.3.3.4	Deacetylipecoside synthase.	NF	NF	NF	NF
3914	>Tropane, piperidine and pyridine alkaloid biosynthesis 00960					
3915	1.1.1.206	Tropinone reductase I.	NF	NF	NF	NF
3916	1.1.1.222	(R)-4-hydroxyphenyllactate dehydrogenase.	NF	NF	NF	NF
3917	1.1.1.236	Tropinone reductase II.	NF	NF	NF	NF
3918	1.1.1.237	Hydroxyphenylpyruvate reductase.	NF	NF	NF	NF
3919	1.14.11.11	Hyoscyamine (6S)-dioxygenase.	NF	NF	NF	NF
3920	1.14.11.14	6-beta-hydroxyhyoscyamine epoxidase.	NF	NF	NF	NF
3921	1.14.13.101	Senecionine N-oxygenase.	NF	NF	NF	NF
3922	1.4.1.18	Lysine 6-dehydrogenase.	NF	NF	NF	NF
3923	1.4.3.14	L-lysine oxidase.	NF	NF	NF	NF
3924	1.4.3.21	Primary-amine oxidase.	NF	NF	FOUND	FOUND
3925	1.5.1.1	Pyrroline-2-carboxylate reductase.	NF	NF	NF	NF
3926	1.5.1.21	Delta(1)-piperideine-2-carboxylate reductase.	NF	NF	NF	NF
3927	2.1.1.53	Putrescine N-methyltransferase.	NF	NF	NF	NF
3928	2.3.1.-	Transferring groups other than amino-acyl groups.	FOUND	FOUND	FOUND	FOUND
3929	2.3.1.145	Piperidine N-piperoyltransferase.	NF	NF	NF	NF
3930	2.3.1.185	Tropine acyltransferase.	NF	NF	NF	NF
3931	2.3.1.186	Pseudotropine acyltransferase.	NF	NF	NF	NF
3932	2.3.1.93	13-hydroxylupinine O-tigloyltransferase.	NF	NF	NF	NF
3933	2.5.1.44	Homospermidine synthase.	NF	NF	NF	NF
3934	2.6.1.1	Aspartate transaminase.	FOUND	FOUND	FOUND	FOUND
3935	2.6.1.5	Tyrosine transaminase.	NF	NF	NF	NF
3936	2.6.1.57	Aromatic-amino-acid transaminase.	NF	NF	NF	NF
3937	2.6.1.58	Phenylalanine(histidine) transaminase.	NF	NF	NF	NF
3938	2.6.1.9	Histidinol-phosphate transaminase.	NF	NF	FOUND	FOUND
3939	2.8.3.17	Cinnamoyl-CoA:phenyllactate CoA-transferase.	NF	NF	NF	NF
3940	3.1.1.-	Carboxylic ester hydrolases.	FOUND	FOUND	FOUND	FOUND
3941	3.1.1.1	Carboxylesterase.	NF	NF	NF	FOUND
3942	3.1.1.10	Tropinesterase.	NF	NF	NF	NF
3943	4.1.1.18	Lysine decarboxylase.	NF	NF	NF	NF
3944	5.1.-.-	Racemases and epimerases.	FOUND	FOUND	FOUND	FOUND
3945	6.2.1.-	Acid--thiol ligases.	NF	NF	NF	NF
3946	>Betalain biosynthesis 00965					
3947	1.10.3.-	With oxygen as acceptor.	NF	NF	NF	NF
3948	1.13.11.-	With incorporation of two atoms of oxygen.	NF	NF	NF	NF
3949	1.14.18.-	With another compound as one donor, and incorpo	NF	NF	NF	NF
3950	1.14.18.1	Monophenol monooxygenase.	NF	NF	NF	NF
3951	2.1.1.6	Catechol O-methyltransferase.	NF	NF	NF	NF
3952	2.3.1.-	Transferring groups other than amino-acyl groups.	FOUND	FOUND	FOUND	FOUND
3953	2.4.1.-	Hexosyltransferases.	FOUND	FOUND	FOUND	FOUND
3954	4.1.1.28	Aromatic-L-amino-acid decarboxylase.	NF	NF	NF	NF

3955	>Glucosinolate biosynthesis 00966					
3956	1.1.1.-	With NAD(+) or NADP(+) as acceptor.	FOUND	FOUND	FOUND	FOUND
3957	1.14.-.-	Acting on paired donors, with incorporation or redu	NF	NF	NF	NF
3958	1.14.13.-	With NADH or NADPH as one donor, and incorporat	NF	NF	NF	NF
3959	1.14.13.41	Tyrosine N-monooxygenase.	NF	NF	NF	NF
3960	2.3.3.-	Acyl groups converted into alkyl on transfer.	FOUND	FOUND	FOUND	FOUND
3961	2.4.1.195	N-hydroxythioamide S-beta-glucosyltransferase.	NF	NF	NF	NF
3962	2.6.1.-	Transaminases (aminotransferases).	FOUND	FOUND	FOUND	FOUND
3963	2.6.1.42	Branched-chain-amino-acid transaminase.	NF	NF	FOUND	FOUND
3964	2.8.2.-	Sulfotransferases.	NF	NF	NF	NF
3965	2.8.2.24	Desulfoglucosinolate sulfotransferase.	NF	NF	NF	NF
3966	4.4.1.-	Carbon-sulfur lyases.	FOUND	NF	FOUND	FOUND
3967	5.4.4.-	Transferring hydroxy groups.	NF	NF	NF	NF
3968	>Aminoacyl-tRNA biosynthesis 00970					
3969	2.1.2.9	Methionyl-tRNA formyltransferase.	FOUND	FOUND	FOUND	FOUND
3970	2.5.1.73	O-phospho-L-seryl-tRNA:Cys-tRNA synthase.	NF	NF	NF	NF
3971	2.7.1.164	O-phosphoseryl-tRNA(Sec) kinase.	NF	NF	NF	NF
3972	2.9.1.1	L-seryl-tRNA(Sec) selenium transferase.	NF	NF	NF	NF
3973	2.9.1.2	O-phospho-L-seryl-tRNA(Sec):L-selenocysteinyl-tRN	NF	NF	NF	NF
3974	6.1.1.1	Tyrosine--tRNA ligase.	FOUND	FOUND	FOUND	FOUND
3975	6.1.1.10	Methionine--tRNA ligase.	FOUND	FOUND	FOUND	FOUND
3976	6.1.1.11	Serine--tRNA ligase.	FOUND	FOUND	FOUND	FOUND
3977	6.1.1.12	Aspartate--tRNA ligase.	FOUND	FOUND	FOUND	FOUND
3978	6.1.1.14	Glycine--tRNA ligase.	FOUND	FOUND	FOUND	FOUND
3979	6.1.1.15	Proline--tRNA ligase.	FOUND	FOUND	FOUND	FOUND
3980	6.1.1.16	Cysteine--tRNA ligase.	FOUND	FOUND	FOUND	FOUND
3981	6.1.1.17	Glutamate--tRNA ligase.	FOUND	FOUND	FOUND	FOUND
3982	6.1.1.18	Glutamine--tRNA ligase.	NF	NF	NF	NF
3983	6.1.1.19	Arginine--tRNA ligase.	FOUND	FOUND	FOUND	FOUND
3984	6.1.1.2	Tryptophan--tRNA ligase.	FOUND	FOUND	FOUND	FOUND
3985	6.1.1.20	Phenylalanine--tRNA ligase.	FOUND	FOUND	FOUND	FOUND
3986	6.1.1.21	Histidine--tRNA ligase.	FOUND	FOUND	FOUND	FOUND
3987	6.1.1.22	Asparagine--tRNA ligase.	NF	NF	NF	NF
3988	6.1.1.23	Aspartate--tRNA(Asn) ligase.	NF	NF	NF	NF
3989	6.1.1.24	Glutamate--tRNA(Gln) ligase.	NF	NF	NF	NF
3990	6.1.1.25	Lysine--tRNA(Pyl) ligase.	NF	NF	NF	NF
3991	6.1.1.26	Pyrrolysine--tRNA(Pyl) ligase.	NF	NF	NF	NF
3992	6.1.1.27	O-phosphoserine--tRNA ligase.	NF	NF	NF	NF
3993	6.1.1.3	Threonine--tRNA ligase.	FOUND	FOUND	FOUND	FOUND
3994	6.1.1.4	Leucine--tRNA ligase.	FOUND	FOUND	FOUND	FOUND
3995	6.1.1.5	Isoleucine--tRNA ligase.	FOUND	FOUND	FOUND	FOUND
3996	6.1.1.6	Lysine--tRNA ligase.	FOUND	FOUND	FOUND	FOUND
3997	6.1.1.7	Alanine--tRNA ligase.	FOUND	FOUND	FOUND	FOUND
3998	6.1.1.9	Valine--tRNA ligase.	FOUND	FOUND	FOUND	FOUND
3999	6.3.5.6	Asparaginyl-tRNA synthase (glutamine-hydrolyzing)	NF	NF	NF	NF
4000	6.3.5.7	Glutaminyl-tRNA synthase (glutamine-hydrolyzing).	NF	NF	NF	NF
4001	>Metabolism of xenobiotics by cytochrome P450 00980					
4002	1.1.1.1	Alcohol dehydrogenase.	FOUND	FOUND	FOUND	FOUND
4003	1.14.14.1	Unspecific monooxygenase.	NF	NF	NF	NF
4004	1.2.1.5	Aldehyde dehydrogenase (NAD(P)(+)).	NF	NF	NF	NF
4005	1.3.1.20	Trans-1,2-dihydrobenzene-1,2-diol dehydrogenase.	NF	NF	NF	NF
4006	2.4.1.17	Glucuronosyltransferase.	NF	NF	NF	NF
4007	2.5.1.18	Glutathione transferase.	NF	NF	NF	NF
4008	3.3.2.9	Microsomal epoxide hydrolase.	NF	NF	NF	NF
4009	>Insect hormone biosynthesis 00981					

4010	1.-.-.-	Oxidoreductases.	FOUND	FOUND	FOUND	FOUND
4011	1.1.1.-	With NAD(+) or NADP(+) as acceptor.	FOUND	FOUND	FOUND	FOUND
4012	1.1.3.16	Ecdysone oxidase.	NF	NF	NF	NF
4013	1.14.99.22	Ecdysone 20-monooxygenase.	NF	NF	NF	NF
4014	2.1.1.-	Methyltransferases.	FOUND	FOUND	FOUND	FOUND
4015	3.1.1.59	Juvenile-hormone esterase.	NF	NF	NF	NF
4016	>Drug metabolism - cytochrome P450 00982					
4017	1.1.1.1	Alcohol dehydrogenase.	FOUND	FOUND	FOUND	FOUND
4018	1.14.13.8	Flavin-containing monooxygenase.	NF	NF	NF	NF
4019	1.14.14.1	Unspecific monooxygenase.	NF	NF	NF	NF
4020	1.2.1.5	Aldehyde dehydrogenase (NAD(P)(+)).	NF	NF	NF	NF
4021	1.2.3.1	Aldehyde oxidase.	NF	NF	NF	NF
4022	1.4.3.4	Monoamine oxidase.	NF	NF	NF	NF
4023	2.4.1.17	Glucuronosyltransferase.	NF	NF	NF	NF
4024	2.5.1.18	Glutathione transferase.	NF	NF	NF	NF
4025	3.3.2.-	Ether hydrolases.	NF	NF	NF	FOUND
4026	>Drug metabolism - other enzymes 00983					
4027	1.1.1.205	IMP dehydrogenase.	FOUND	FOUND	FOUND	FOUND
4028	1.14.14.1	Unspecific monooxygenase.	NF	NF	NF	NF
4029	1.17.3.2	Xanthine oxidase.	NF	NF	NF	NF
4030	1.3.1.2	Dihydropyrimidine dehydrogenase (NADP(+)).	NF	NF	NF	NF
4031	2.1.1.67	Thiopurine S-methyltransferase.	NF	NF	NF	NF
4032	2.3.1.5	Arylamine N-acetyltransferase.	NF	NF	NF	NF
4033	2.4.1.17	Glucuronosyltransferase.	NF	NF	NF	NF
4034	2.4.2.10	Orotate phosphoribosyltransferase.	NF	NF	FOUND	FOUND
4035	2.4.2.3	Uridine phosphorylase.	NF	NF	NF	FOUND
4036	2.4.2.4	Thymidine phosphorylase.	FOUND	FOUND	FOUND	FOUND
4037	2.4.2.8	Hypoxanthine phosphoribosyltransferase.	FOUND	FOUND	FOUND	FOUND
4038	2.7.1.21	Thymidine kinase.	FOUND	FOUND	FOUND	FOUND
4039	2.7.1.48	Uridine kinase.	FOUND	FOUND	FOUND	FOUND
4040	2.7.4.-	Phosphotransferases with a phosphate group as acc	FOUND	FOUND	FOUND	FOUND
4041	3.-.-.-	Hydrolases.	FOUND	FOUND	FOUND	FOUND
4042	3.1.1.1	Carboxylesterase.	NF	NF	NF	FOUND
4043	3.2.1.31	Beta-glucuronidase.	NF	NF	NF	NF
4044	3.5.1.6	Beta-ureidopropionase.	NF	NF	NF	NF
4045	3.5.2.2	Dihydropyrimidinase.	NF	NF	NF	FOUND
4046	3.5.4.5	Cytidine deaminase.	FOUND	FOUND	FOUND	FOUND
4047	3.6.1.19	Nucleoside-triphosphate diphosphatase.	NF	NF	NF	NF
4048	6.3.5.2	GMP synthase (glutamine-hydrolyzing).	FOUND	FOUND	FOUND	FOUND
4049	>Overview of biosynthetic pathways 01010					
4050	>Biosynthesis of unsaturated fatty acids 01040					
4051	1.1.1.-	With NAD(+) or NADP(+) as acceptor.	FOUND	FOUND	FOUND	FOUND
4052	1.1.1.100	3-oxoacyl-[acyl-carrier-protein] reductase.	NF	NF	FOUND	FOUND
4053	1.1.1.211	Long-chain-3-hydroxyacyl-CoA dehydrogenase.	NF	NF	NF	NF
4054	1.14.19.-	With oxidation of a pair of donors resulting in the re	NF	NF	NF	NF
4055	1.14.19.1	Stearoyl-CoA 9-desaturase.	NF	NF	NF	NF
4056	1.14.19.2	Acyl-[acyl-carrier-protein] desaturase.	NF	NF	NF	NF
4057	1.3.1.-	With NAD(+) or NADP(+) as acceptor.	FOUND	FOUND	FOUND	FOUND
4058	1.3.1.38	Trans-2-enoyl-CoA reductase (NADPH).	NF	NF	NF	NF
4059	1.3.3.6	Acyl-CoA oxidase.	NF	NF	NF	NF
4060	2.3.1.-	Transferring groups other than amino-acyl groups.	FOUND	FOUND	FOUND	FOUND
4061	2.3.1.16	Acetyl-CoA C-acyltransferase.	NF	NF	NF	NF
4062	3.1.2.-	Thiolester hydrolases.	NF	FOUND	NF	NF
4063	3.1.2.2	Palmitoyl-CoA hydrolase.	NF	NF	NF	NF
4064	4.2.1.-	Hydro-lyases.	FOUND	FOUND	FOUND	FOUND

4065	4.2.1.17	Enoyl-CoA hydratase.	NF	NF	NF	NF
4066	>Biosynthesis of ansamycins 01051					
4067	2.2.1.1	Transketolase.	FOUND	FOUND	FOUND	FOUND
4068	5.3.1.-	Interconverting aldoses and ketoses, and related co	FOUND	FOUND	FOUND	FOUND
4069	>Type I polyketide structures 01052					
4070	>Biosynthesis of siderophore group nonribosomal peptides 01053					
4071	1.3.1.28	2,3-dihydro-2,3-dihydroxybenzoate dehydrogenase	NF	NF	NF	NF
4072	2.7.7.-	Nucleotidyltransferases.	FOUND	FOUND	FOUND	FOUND
4073	2.7.7.58	(2,3-dihydroxybenzoyl)adenylate synthase.	NF	NF	NF	NF
4074	3.3.2.1	Isochorismatase.	NF	NF	NF	FOUND
4075	4.1.99.-	Other carbon-carbon lyases.	NF	NF	NF	FOUND
4076	5.4.4.2	Isochorismate synthase.	NF	NF	NF	NF
4077	6.3.2.-	Acid--D-amino-acid ligases (peptide synthases).	FOUND	FOUND	FOUND	FOUND
4078	>Nonribosomal peptide structures 01054					
4079	>Biosynthesis of vancomycin group antibiotics 01055					
4080	4.2.1.46	dTDP-glucose 4,6-dehydratase.	NF	NF	FOUND	FOUND
4081	>Biosynthesis of type II polyketide backbone 01056					
4082	1.1.1.-	With NAD(+) or NADP(+) as acceptor.	FOUND	FOUND	FOUND	FOUND
4083	2.3.1.-	Transferring groups other than amino-acyl groups.	FOUND	FOUND	FOUND	FOUND
4084	4.2.1.-	Hydro-lyases.	FOUND	FOUND	FOUND	FOUND
4085	>Biosynthesis of type II polyketide products 01057					
4086	1.-.-.-	Oxidoreductases.	FOUND	FOUND	FOUND	FOUND
4087	1.1.1.-	With NAD(+) or NADP(+) as acceptor.	FOUND	FOUND	FOUND	FOUND
4088	1.1.3.-	With oxygen as acceptor.	NF	NF	NF	FOUND
4089	1.14.-.-	Acting on paired donors, with incorporation or redu	NF	NF	NF	NF
4090	1.14.13.-	With NADH or NADPH as one donor, and incorporat	NF	NF	NF	NF
4091	1.14.13.38	Anhydrotetracycline monooxygenase.	NF	NF	NF	NF
4092	1.14.14.-	With reduced flavin or flavoprotein as one donor, a	NF	NF	NF	NF
4093	1.3.3.-	With oxygen as acceptor.	FOUND	FOUND	FOUND	FOUND
4094	1.5.1.29	FMN reductase.	NF	NF	NF	FOUND
4095	2.1.1.-	Methyltransferases.	FOUND	FOUND	FOUND	FOUND
4096	2.4.1.-	Hexosyltransferases.	FOUND	FOUND	FOUND	FOUND
4097	4.-.-.-	Lyases.	FOUND	FOUND	FOUND	FOUND
4098	4.2.1.-	Hydro-lyases.	FOUND	FOUND	FOUND	FOUND
4099	>Acridone alkaloid biosynthesis 01058					
4100	2.1.1.111	Anthranilate N-methyltransferase.	NF	NF	NF	NF
4101	2.3.1.159	Acridone synthase.	NF	NF	NF	NF
4102	6.2.1.32	Anthranilate--CoA ligase.	NF	NF	NF	NF
4103	>Biosynthesis of plant secondary metabolites 01060					
4104	>Biosynthesis of phenylpropanoids 01061					
4105	1.1.1.195	Cinnamyl-alcohol dehydrogenase.	NF	NF	NF	NF
4106	1.1.1.219	Dihydrokaempferol 4-reductase.	NF	NF	NF	NF
4107	1.1.1.246	Pterocarpin synthase.	NF	NF	NF	NF
4108	1.1.1.25	Shikimate dehydrogenase.	FOUND	FOUND	FOUND	FOUND
4109	1.1.1.282	Quinate/shikimate dehydrogenase.	NF	NF	NF	NF
4110	1.1.1.37	Malate dehydrogenase.	NF	NF	NF	NF
4111	1.1.1.38	Malate dehydrogenase (oxaloacetate-decarboxylati	NF	NF	NF	NF
4112	1.1.1.42	Isocitrate dehydrogenase (NADP(+)).	NF	NF	FOUND	FOUND
4113	1.1.5.8	Quinate dehydrogenase (quinone).	NF	NF	NF	NF
4114	1.11.1.7	Peroxidase.	FOUND	FOUND	FOUND	FOUND
4115	1.14.-.-	Acting on paired donors, with incorporation or redu	NF	NF	NF	NF
4116	1.14.11.19	Leucocyanidin oxygenase.	NF	NF	NF	NF
4117	1.14.11.22	Flavone synthase.	NF	NF	NF	NF
4118	1.14.11.23	Flavonol synthase.	NF	NF	NF	NF
4119	1.14.11.9	Flavanone 3-dioxygenase.	NF	NF	NF	NF

4120	1.14.13.-	With NADH or NADPH as one donor, and incorporat	NF	NF	NF	NF
4121	1.14.13.11	Trans-cinnamate 4-monooxygenase.	NF	NF	NF	NF
4122	1.14.13.21	Flavonoid 3'-monooxygenase.	NF	NF	NF	NF
4123	1.14.13.36	5-O-(4-coumaroyl)-D-quinate 3'-monooxygenase.	NF	NF	NF	NF
4124	1.14.13.52	Isoflavone 3'-hydroxylase.	NF	NF	NF	NF
4125	1.14.13.53	4'-methoxyisoflavone 2'-hydroxylase.	NF	NF	NF	NF
4126	1.14.13.86	2-hydroxyisoflavanone synthase.	NF	NF	NF	NF
4127	1.14.13.89	Isoflavone 2'-hydroxylase.	NF	NF	NF	NF
4128	1.14.99.-	Miscellaneous (requires further characterization).	NF	NF	NF	NF
4129	1.17.1.3	Leucoanthocyanidin reductase.	NF	NF	NF	NF
4130	1.2.1.12	Glyceraldehyde-3-phosphate dehydrogenase (phos	FOUND	FOUND	FOUND	FOUND
4131	1.2.1.13	Glyceraldehyde-3-phosphate dehydrogenase (NADF	NF	NF	NF	NF
4132	1.2.1.28	Benzaldehyde dehydrogenase (NAD(+)).	NF	NF	NF	NF
4133	1.2.1.44	Cinnamoyl-CoA reductase.	NF	NF	NF	NF
4134	1.2.1.67	Vanillin dehydrogenase.	NF	NF	NF	NF
4135	1.2.1.7	Benzaldehyde dehydrogenase (NADP(+)).	NF	NF	NF	NF
4136	1.2.4.1	Pyruvate dehydrogenase (acetyl-transferring).	FOUND	FOUND	FOUND	FOUND
4137	1.2.4.2	Oxoglutarate dehydrogenase (succinyl-transferring)	NF	NF	NF	NF
4138	1.3.1.28	2,3-dihydro-2,3-dihydroxybenzoate dehydrogenase	NF	NF	NF	NF
4139	1.3.1.45	2'-hydroxyisoflavone reductase.	NF	NF	NF	NF
4140	1.3.1.77	Anthocyanidin reductase.	NF	NF	NF	NF
4141	1.3.5.1	Succinate dehydrogenase (ubiquinone).	NF	NF	NF	NF
4142	1.3.99.1	Succinate dehydrogenase.	NF	NF	NF	NF
4143	1.4.1.20	Phenylalanine dehydrogenase.	NF	NF	NF	NF
4144	1.5.1.3	Dihydrofolate reductase.	FOUND	FOUND	FOUND	FOUND
4145	1.8.1.4	Dihydrolipoyl dehydrogenase.	FOUND	FOUND	FOUND	FOUND
4146	2.1.1.104	Caffeoyl-CoA O-methyltransferase.	NF	NF	NF	NF
4147	2.1.1.150	Isoflavone 7-O-methyltransferase.	NF	NF	NF	NF
4148	2.1.1.46	Isoflavone 4'-O-methyltransferase.	NF	NF	NF	NF
4149	2.1.1.68	Caffeate O-methyltransferase.	NF	NF	NF	NF
4150	2.1.1.69	5-hydroxyfuranocoumarin 5-O-methyltransferase.	NF	NF	NF	NF
4151	2.2.1.1	Transketolase.	FOUND	FOUND	FOUND	FOUND
4152	2.2.1.2	Transaldolase.	FOUND	NF	FOUND	FOUND
4153	2.3.1.12	Dihydrolipoyllysine-residue acetyltransferase.	FOUND	FOUND	FOUND	FOUND
4154	2.3.1.133	Shikimate O-hydroxycinnamoyltransferase.	NF	NF	NF	NF
4155	2.3.1.143	Beta-glucogallin--tetrakisgalloylglucose O-galloyltra	NF	NF	NF	NF
4156	2.3.1.61	Dihydrolipoyllysine-residue succinyltransferase.	FOUND	NF	FOUND	NF
4157	2.3.1.74	Naringenin-chalcone synthase.	NF	NF	NF	NF
4158	2.3.1.90	Beta-glucogallin O-galloyltransferase.	NF	NF	NF	NF
4159	2.3.1.95	Trihydroxystilbene synthase.	NF	NF	NF	NF
4160	2.3.3.1	Citrate (Si)-synthase.	NF	NF	FOUND	FOUND
4161	2.3.3.8	ATP citrate synthase.	NF	NF	NF	NF
4162	2.4.1.128	Scopoletin glucosyltransferase.	NF	NF	NF	NF
4163	2.4.1.136	Gallate 1-beta-glucosyltransferase.	NF	NF	NF	NF
4164	2.4.2.18	Anthranilate phosphoribosyltransferase.	NF	NF	FOUND	FOUND
4165	2.5.1.15	Dihydropteroate synthase.	NF	NF	FOUND	FOUND
4166	2.5.1.19	3-phosphoshikimate 1-carboxyvinyltransferase.	NF	NF	FOUND	FOUND
4167	2.5.1.54	3-deoxy-7-phosphoheptulonate synthase.	NF	NF	FOUND	FOUND
4168	2.6.1.1	Aspartate transaminase.	FOUND	FOUND	FOUND	FOUND
4169	2.6.1.5	Tyrosine transaminase.	NF	NF	NF	NF
4170	2.6.1.57	Aromatic-amino-acid transaminase.	NF	NF	NF	NF
4171	2.6.1.85	Aminodeoxychorismate synthase.	NF	NF	NF	NF
4172	2.6.1.9	Histidinol-phosphate transaminase.	NF	NF	FOUND	FOUND
4173	2.7.1.1	Hexokinase.	NF	NF	NF	NF
4174	2.7.1.11	6-phosphofructokinase.	FOUND	FOUND	FOUND	FOUND

4175	2.7.1.2	Glucokinase.	FOUND	FOUND	FOUND	FOUND
4176	2.7.1.40	Pyruvate kinase.	FOUND	FOUND	FOUND	FOUND
4177	2.7.1.71	Shikimate kinase.	NF	FOUND	FOUND	FOUND
4178	2.7.2.3	Phosphoglycerate kinase.	FOUND	FOUND	FOUND	FOUND
4179	3.1.3.11	Fructose-bisphosphatase.	NF	NF	NF	FOUND
4180	3.1.3.13	Bisphosphoglycerate phosphatase.	NF	NF	NF	NF
4181	3.3.2.1	Isochorismatase.	NF	NF	NF	FOUND
4182	4.1.1.48	Indole-3-glycerol-phosphate synthase.	NF	NF	FOUND	FOUND
4183	4.1.2.13	Fructose-bisphosphate aldolase.	FOUND	FOUND	FOUND	FOUND
4184	4.1.3.27	Anthranilate synthase.	NF	NF	FOUND	FOUND
4185	4.1.3.38	Aminodeoxychorismate lyase.	NF	NF	NF	FOUND
4186	4.1.99.-	Other carbon-carbon lyases.	NF	NF	NF	FOUND
4187	4.2.1.10	3-dehydroquinate dehydratase.	NF	NF	FOUND	FOUND
4188	4.2.1.105	2-hydroxyisoflavanone dehydratase.	NF	NF	NF	NF
4189	4.2.1.11	Phosphopyruvate hydratase.	FOUND	FOUND	FOUND	FOUND
4190	4.2.1.2	Fumarate hydratase.	NF	NF	NF	FOUND
4191	4.2.1.20	Tryptophan synthase.	NF	NF	FOUND	FOUND
4192	4.2.1.3	Aconitate hydratase.	NF	NF	FOUND	NF
4193	4.2.1.51	Prephenate dehydratase.	NF	NF	FOUND	FOUND
4194	4.2.1.91	Arogenate dehydratase.	NF	NF	NF	NF
4195	4.2.3.4	3-dehydroquinate synthase.	NF	NF	FOUND	FOUND
4196	4.2.3.5	Chorismate synthase.	NF	NF	FOUND	FOUND
4197	4.3.1.24	Phenylalanine ammonia-lyase.	NF	NF	NF	NF
4198	5.3.1.24	Phosphoribosylanthranilate isomerase.	NF	NF	FOUND	FOUND
4199	5.3.1.9	Glucose-6-phosphate isomerase.	FOUND	FOUND	FOUND	FOUND
4200	5.4.2.1	Phosphoglycerate mutase.	FOUND	FOUND	FOUND	FOUND
4201	5.4.2.4	Bisphosphoglycerate mutase.	NF	NF	NF	NF
4202	5.4.4.2	Isochorismate synthase.	NF	NF	NF	NF
4203	5.4.99.5	Chorismate mutase.	NF	NF	FOUND	FOUND
4204	5.5.1.6	Chalcone isomerase.	NF	NF	NF	NF
4205	6.2.1.12	4-coumarate--CoA ligase.	NF	NF	NF	NF
4206	6.2.1.4	Succinate--CoA ligase (GDP-forming).	NF	NF	NF	NF
4207	6.2.1.5	Succinate--CoA ligase (ADP-forming).	NF	NF	NF	NF
4208	6.3.2.12	Dihydrofolate synthase.	NF	NF	NF	NF
4209	6.3.2.17	Tetrahydrofolate synthase.	FOUND	FOUND	FOUND	FOUND
4210	>Biosynthesis of terpenoids and steroids 01062					
4211	1.1.1.145	3-beta-hydroxy-Delta(5)-steroid dehydrogenase.	NF	NF	NF	NF
4212	1.1.1.267	1-deoxy-D-xylulose-5-phosphate reductoisomerase.	NF	NF	NF	NF
4213	1.1.1.288	Xanthoxin dehydrogenase.	NF	NF	NF	NF
4214	1.1.1.34	Hydroxymethylglutaryl-CoA reductase (NADPH).	FOUND	FOUND	FOUND	FOUND
4215	1.1.1.37	Malate dehydrogenase.	NF	NF	NF	NF
4216	1.1.1.38	Malate dehydrogenase (oxaloacetate-decarboxylati	NF	NF	NF	NF
4217	1.1.1.39	Malate dehydrogenase (decarboxylating).	NF	NF	NF	NF
4218	1.1.1.40	Malate dehydrogenase (oxaloacetate-decarboxylati	NF	NF	FOUND	NF
4219	1.1.1.41	Isocitrate dehydrogenase (NAD(+)).	NF	NF	FOUND	FOUND
4220	1.1.1.42	Isocitrate dehydrogenase (NADP(+)).	NF	NF	FOUND	FOUND
4221	1.13.11.51	9-cis-epoxycarotenoid dioxygenase.	NF	NF	NF	NF
4222	1.14.-.-	Acting on paired donors, with incorporation or redu	NF	NF	NF	NF
4223	1.14.11.12	Gibberellin-44 dioxygenase.	NF	NF	NF	NF
4224	1.14.11.15	Gibberellin 3-beta-dioxygenase.	NF	NF	NF	NF
4225	1.14.13.-	With NADH or NADPH as one donor, and incorporat	NF	NF	NF	NF
4226	1.14.13.70	Sterol 14-demethylase.	NF	NF	NF	NF
4227	1.14.13.74	7-deoxyloganin 7-hydroxylase.	NF	NF	NF	NF
4228	1.14.13.77	Taxane 13-alpha-hydroxylase.	NF	NF	NF	NF
4229	1.14.13.78	Ent-kaurene oxidase.	NF	NF	NF	NF

4230	1.14.13.79	Ent-kaurenoic acid oxidase.	NF	NF	NF	NF
4231	1.14.13.90	Zeaxanthin epoxidase.	NF	NF	NF	NF
4232	1.14.21.6	Lathosterol oxidase.	NF	NF	NF	NF
4233	1.14.99.-	Miscellaneous (requires further characterization).	NF	NF	NF	NF
4234	1.14.99.30	Carotene 7,8-desaturase.	NF	NF	NF	NF
4235	1.14.99.37	Taxadiene 5-alpha-hydroxylase.	NF	NF	NF	NF
4236	1.14.99.7	Squalene monooxygenase.	NF	NF	NF	NF
4237	1.17.1.2	4-hydroxy-3-methylbut-2-enyl diphosphate reducta	NF	NF	NF	FOUND
4238	1.17.7.1	(E)-4-hydroxy-3-methylbut-2-enyl-diphosphate synt	NF	NF	NF	FOUND
4239	1.2.1.12	Glyceraldehyde-3-phosphate dehydrogenase (phos	FOUND	FOUND	FOUND	FOUND
4240	1.2.1.13	Glyceraldehyde-3-phosphate dehydrogenase (NADF	NF	NF	NF	NF
4241	1.2.3.14	Abscisic-aldehyde oxidase.	NF	NF	NF	NF
4242	1.2.4.1	Pyruvate dehydrogenase (acetyl-transferring).	FOUND	FOUND	FOUND	FOUND
4243	1.2.4.2	Oxoglutarate dehydrogenase (succinyl-transferring)	NF	NF	NF	NF
4244	1.3.1.21	7-dehydrocholesterol reductase.	NF	NF	NF	NF
4245	1.3.1.70	Delta(14)-sterol reductase.	NF	NF	NF	NF
4246	1.3.1.72	Delta(24)-sterol reductase.	NF	NF	NF	NF
4247	1.3.3.9	Secologanin synthase.	NF	NF	NF	NF
4248	1.3.5.1	Succinate dehydrogenase (ubiquinone).	NF	NF	NF	NF
4249	1.3.99.-	With other acceptors.	NF	NF	FOUND	FOUND
4250	1.8.1.4	Dihydrolipoyl dehydrogenase.	FOUND	FOUND	FOUND	FOUND
4251	2.1.1.143	24-methylenesterol C-methyltransferase.	NF	NF	NF	NF
4252	2.1.1.41	Sterol 24-C-methyltransferase.	NF	NF	NF	NF
4253	2.2.1.7	1-deoxy-D-xylulose-5-phosphate synthase.	NF	NF	FOUND	FOUND
4254	2.3.1.-	Transferring groups other than amino-acyl groups.	FOUND	FOUND	FOUND	FOUND
4255	2.3.1.12	Dihydrolipoyllysine-residue acetyltransferase.	FOUND	FOUND	FOUND	FOUND
4256	2.3.1.166	2-alpha-hydroxytaxane 2-O-benzoyltransferase.	NF	NF	NF	NF
4257	2.3.1.167	10-deacetylbaccatin III 10-O-acetyltransferase.	NF	NF	NF	NF
4258	2.3.1.61	Dihydrolipoyllysine-residue succinyltransferase.	FOUND	NF	FOUND	NF
4259	2.3.2.-	Aminoacyltransferases.	NF	NF	NF	NF
4260	2.3.3.1	Citrate (Si)-synthase.	NF	NF	FOUND	FOUND
4261	2.3.3.10	Hydroxymethylglutaryl-CoA synthase.	FOUND	FOUND	FOUND	NF
4262	2.3.3.8	ATP citrate synthase.	NF	NF	NF	NF
4263	2.5.1.-	Transferring alkyl or aryl groups, other than methyl	FOUND	FOUND	FOUND	FOUND
4264	2.5.1.1	Dimethylallyltranstransferase.	NF	NF	FOUND	FOUND
4265	2.5.1.10	(2E,6E)-farnesyl diphosphate synthase.	FOUND	NF	NF	NF
4266	2.5.1.21	Squalene synthase.	NF	NF	NF	NF
4267	2.5.1.29	Farnesyltranstransferase.	NF	NF	NF	NF
4268	2.5.1.31	Di-trans,poly-cis-undecaprenyl-diphosphate synthas	FOUND	FOUND	FOUND	FOUND
4269	2.5.1.32	Phytoene synthase.	NF	NF	NF	NF
4270	2.7.1.1	Hexokinase.	NF	NF	NF	NF
4271	2.7.1.11	6-phosphofructokinase.	FOUND	FOUND	FOUND	FOUND
4272	2.7.1.148	4-(cytidine 5'-diphospho)-2-C-methyl-D-erythritol ki	NF	NF	NF	FOUND
4273	2.7.1.2	Glucokinase.	FOUND	FOUND	FOUND	FOUND
4274	2.7.1.36	Mevalonate kinase.	FOUND	FOUND	FOUND	NF
4275	2.7.1.40	Pyruvate kinase.	FOUND	FOUND	FOUND	FOUND
4276	2.7.2.3	Phosphoglycerate kinase.	FOUND	FOUND	FOUND	FOUND
4277	2.7.4.2	Phosphomevalonate kinase.	FOUND	FOUND	FOUND	NF
4278	2.7.7.60	2-C-methyl-D-erythritol 4-phosphate cytidylyltransf	NF	NF	NF	NF
4279	3.1.3.51	Dolichyl-phosphatase.	NF	NF	NF	NF
4280	3.1.7.3	Monoterpenyl-diphosphatase.	NF	NF	NF	NF
4281	3.6.1.43	Dolichyldiphosphatase.	NF	NF	NF	NF
4282	4.1.1.33	Diphosphomevalonate decarboxylase.	FOUND	FOUND	FOUND	NF
4283	4.1.2.13	Fructose-bisphosphate aldolase.	FOUND	FOUND	FOUND	FOUND
4284	4.2.1.11	Phosphopyruvate hydratase.	FOUND	FOUND	FOUND	FOUND

4285	4.2.1.2	Fumarate hydratase.	NF	NF	NF	FOUND
4286	4.2.1.3	Aconitate hydratase.	NF	NF	FOUND	NF
4287	4.2.3.-	Acting on phosphates.	NF	NF	FOUND	FOUND
4288	4.2.3.10	(-)-endo-fenchol synthase.	NF	NF	NF	NF
4289	4.2.3.11	Sabinene-hydrate synthase.	NF	NF	NF	NF
4290	4.2.3.13	(+)-delta-cadinene synthase.	NF	NF	NF	NF
4291	4.2.3.14	Pinene synthase.	NF	NF	NF	NF
4292	4.2.3.15	Myrcene synthase.	NF	NF	NF	NF
4293	4.2.3.16	(4S)-limonene synthase.	NF	NF	NF	NF
4294	4.2.3.17	Taxadiene synthase.	NF	NF	NF	NF
4295	4.2.3.18	Abietadiene synthase.	NF	NF	NF	NF
4296	4.2.3.20	(R)-limonene synthase.	NF	NF	NF	NF
4297	4.2.3.21	Vetispiradiene synthase.	NF	NF	NF	NF
4298	4.2.3.22	Germacradienol synthase.	NF	NF	NF	NF
4299	4.2.3.23	Germacrene-A synthase.	NF	NF	NF	NF
4300	4.2.3.25	S-linalool synthase.	NF	NF	NF	NF
4301	4.2.3.26	R-linalool synthase.	NF	NF	NF	NF
4302	4.2.3.27	Isoprene synthase.	NF	NF	NF	NF
4303	4.2.3.8	Casbene synthase.	NF	NF	NF	NF
4304	4.2.3.9	Aristolochene synthase.	NF	NF	NF	NF
4305	4.6.1.12	2-C-methyl-D-erythritol 2,4-cyclodiphosphate synth	NF	NF	NF	FOUND
4306	5.3.1.9	Glucose-6-phosphate isomerase.	FOUND	FOUND	FOUND	FOUND
4307	5.3.3.1	Steroid Delta-isomerase.	NF	NF	NF	NF
4308	5.3.3.2	Isopentenyl-diphosphate Delta-isomerase.	FOUND	FOUND	FOUND	FOUND
4309	5.3.3.5	Cholestenol Delta-isomerase.	NF	NF	NF	NF
4310	5.3.99.9	Neoxanthin synthase.	NF	NF	NF	NF
4311	5.4.2.1	Phosphoglycerate mutase.	FOUND	FOUND	FOUND	FOUND
4312	5.4.99.8	Cycloartenol synthase.	NF	NF	NF	NF
4313	5.5.1.12	Copalyl diphosphate synthase.	NF	NF	NF	NF
4314	5.5.1.13	Ent-copalyl diphosphate synthase.	NF	NF	NF	NF
4315	5.5.1.8	Bornyl diphosphate synthase.	NF	NF	NF	NF
4316	5.5.1.9	Cycloeucalenol cycloisomerase.	NF	NF	NF	NF
4317	6.2.1.4	Succinate--CoA ligase (GDP-forming).	NF	NF	NF	NF
4318	6.2.1.5	Succinate--CoA ligase (ADP-forming).	NF	NF	NF	NF
4319	>Biosynthesis of alkaloids derived from shikimate pathway 01063					
4320	1.1.1.-	With NAD(+) or NADP(+) as acceptor.	FOUND	FOUND	FOUND	FOUND
4321	1.1.1.218	Morphine 6-dehydrogenase.	NF	NF	NF	NF
4322	1.1.1.247	Codeinone reductase (NADPH).	NF	NF	NF	NF
4323	1.1.1.248	Salutaridine reductase (NADPH).	NF	NF	NF	NF
4324	1.1.1.25	Shikimate dehydrogenase.	FOUND	FOUND	FOUND	FOUND
4325	1.1.1.37	Malate dehydrogenase.	NF	NF	NF	NF
4326	1.1.1.39	Malate dehydrogenase (decarboxylating).	NF	NF	NF	NF
4327	1.1.1.40	Malate dehydrogenase (oxaloacetate-decarboxylati	NF	NF	FOUND	NF
4328	1.1.1.41	Isocitrate dehydrogenase (NAD(+)).	NF	NF	FOUND	FOUND
4329	1.1.1.42	Isocitrate dehydrogenase (NADP(+)).	NF	NF	FOUND	FOUND
4330	1.14.11.20	Deacetoxyvindoline 4-hydroxylase.	NF	NF	NF	NF
4331	1.14.13.-	With NADH or NADPH as one donor, and incorporat	NF	NF	NF	NF
4332	1.14.13.11	Trans-cinnamate 4-monooxygenase.	NF	NF	NF	NF
4333	1.14.13.37	Methyltetrahydroprotoberberine 14-monooxygena	NF	NF	NF	NF
4334	1.14.13.55	Protopine 6-monooxygenase.	NF	NF	NF	NF
4335	1.14.13.56	Dihydrosanguinarine 10-monooxygenase.	NF	NF	NF	NF
4336	1.14.13.57	Dihydrochelirubine 12-monooxygenase.	NF	NF	NF	NF
4337	1.14.13.73	Tabersonine 16-hydroxylase.	NF	NF	NF	NF
4338	1.14.14.1	Unspecific monooxygenase.	NF	NF	NF	NF
4339	1.14.16.4	Tryptophan 5-monooxygenase.	NF	NF	NF	NF

4340	1.14.18.1	Monophenol monooxygenase.	NF	NF	NF	NF
4341	1.14.21.1	(S)-stylopine synthase.	NF	NF	NF	NF
4342	1.14.21.2	(S)-cheilanthifoline synthase.	NF	NF	NF	NF
4343	1.14.21.4	Salutaridine synthase.	NF	NF	NF	NF
4344	1.2.1.12	Glyceraldehyde-3-phosphate dehydrogenase (phos	FOUND	FOUND	FOUND	FOUND
4345	1.2.1.13	Glyceraldehyde-3-phosphate dehydrogenase (NADF	NF	NF	NF	NF
4346	1.2.4.1	Pyruvate dehydrogenase (acetyl-transferring).	FOUND	FOUND	FOUND	FOUND
4347	1.2.4.2	Oxoglutarate dehydrogenase (succinyl-transferring)	NF	NF	NF	NF
4348	1.2.4.4	3-methyl-2-oxobutanoate dehydrogenase (2-methy	NF	NF	NF	FOUND
4349	1.21.3.2	Columbamine oxidase.	NF	NF	NF	NF
4350	1.21.3.3	Reticuline oxidase.	NF	NF	NF	NF
4351	1.3.-.-	Acting on the CH-CH group of donors.	FOUND	FOUND	FOUND	FOUND
4352	1.3.3.8	Tetrahydroberberine oxidase.	NF	NF	NF	NF
4353	1.3.5.1	Succinate dehydrogenase (ubiquinone).	NF	NF	NF	NF
4354	1.5.-.-	Acting on the CH-NH group of donors.	FOUND	FOUND	FOUND	FOUND
4355	1.5.1.27	1,2-dehydroreticulinium reductase (NADPH).	NF	NF	NF	NF
4356	1.5.3.12	Dihydrobenzophenanthridine oxidase.	NF	NF	NF	NF
4357	1.8.1.4	Dihydrolipoyl dehydrogenase.	FOUND	FOUND	FOUND	FOUND
4358	2.1.1.-	Methyltransferases.	FOUND	FOUND	FOUND	FOUND
4359	2.1.1.104	Caffeoyl-CoA O-methyltransferase.	NF	NF	NF	NF
4360	2.1.1.111	Anthranilate N-methyltransferase.	NF	NF	NF	NF
4361	2.1.1.116	3'-hydroxy-N-methyl-(S)-coclaurine 4'-O-methyltran	NF	NF	NF	NF
4362	2.1.1.117	(S)-scoulerine 9-O-methyltransferase.	NF	NF	NF	NF
4363	2.1.1.119	10-hydroxydihydrosanguinarine 10-O-methyltransfe	NF	NF	NF	NF
4364	2.1.1.120	12-hydroxydihydrochelirubine 12-O-methyltransfer	NF	NF	NF	NF
4365	2.1.1.122	(S)-tetrahydroprotoberberine N-methyltransferase.	NF	NF	NF	NF
4366	2.1.1.128	(RS)-norcoclaurine 6-O-methyltransferase.	NF	NF	NF	NF
4367	2.1.1.140	(S)-coclaurine-N-methyltransferase.	NF	NF	NF	NF
4368	2.1.1.94	Tabersonine 16-O-methyltransferase.	NF	NF	NF	NF
4369	2.1.1.99	3-hydroxy-16-methoxy-2,3-dihydrotabersonine N-m	NF	NF	NF	NF
4370	2.2.1.1	Transketolase.	FOUND	FOUND	FOUND	FOUND
4371	2.2.1.2	Transaldolase.	FOUND	NF	FOUND	FOUND
4372	2.3.1.107	Deacetylvindoline O-acetyltransferase.	NF	NF	NF	NF
4373	2.3.1.12	Dihydrolipoyllysine-residue acetyltransferase.	FOUND	FOUND	FOUND	FOUND
4374	2.3.1.150	Salutaridinol 7-O-acetyltransferase.	NF	NF	NF	NF
4375	2.3.1.159	Acridone synthase.	NF	NF	NF	NF
4376	2.3.1.168	Dihydrolipoyllysine-residue (2-methylpropanoyl)tra	NF	NF	NF	NF
4377	2.3.1.61	Dihydrolipoyllysine-residue succinyltransferase.	FOUND	NF	FOUND	NF
4378	2.3.3.1	Citrate (Si)-synthase.	NF	NF	FOUND	FOUND
4379	2.3.3.8	ATP citrate synthase.	NF	NF	NF	NF
4380	2.4.2.18	Anthranilate phosphoribosyltransferase.	NF	NF	FOUND	FOUND
4381	2.5.1.19	3-phosphoshikimate 1-carboxyvinyltransferase.	NF	NF	FOUND	FOUND
4382	2.5.1.54	3-deoxy-7-phosphoheptulonate synthase.	NF	NF	FOUND	FOUND
4383	2.6.1.42	Branched-chain-amino-acid transaminase.	NF	NF	FOUND	FOUND
4384	2.6.1.5	Tyrosine transaminase.	NF	NF	NF	NF
4385	2.6.1.57	Aromatic-amino-acid transaminase.	NF	NF	NF	NF
4386	2.7.1.1	Hexokinase.	NF	NF	NF	NF
4387	2.7.1.11	6-phosphofructokinase.	FOUND	FOUND	FOUND	FOUND
4388	2.7.1.2	Glucokinase.	FOUND	FOUND	FOUND	FOUND
4389	2.7.1.40	Pyruvate kinase.	FOUND	FOUND	FOUND	FOUND
4390	2.7.1.71	Shikimate kinase.	NF	FOUND	FOUND	FOUND
4391	2.7.2.3	Phosphoglycerate kinase.	FOUND	FOUND	FOUND	FOUND
4392	3.2.1.105	3-alpha-(S)-strictosidine beta-glucosidase.	NF	NF	NF	NF
4393	4.1.1.25	Tyrosine decarboxylase.	NF	NF	NF	NF
4394	4.1.1.28	Aromatic-L-amino-acid decarboxylase.	NF	NF	NF	NF

4395	4.1.1.48	Indole-3-glycerol-phosphate synthase.	NF	NF	FOUND	FOUND
4396	4.1.1.80	4-hydroxyphenylpyruvate decarboxylase.	NF	NF	NF	NF
4397	4.1.2.13	Fructose-bisphosphate aldolase.	FOUND	FOUND	FOUND	FOUND
4398	4.1.2.41	Vanillin synthase.	NF	NF	NF	NF
4399	4.1.3.27	Anthranilate synthase.	NF	NF	FOUND	FOUND
4400	4.2.1.10	3-dehydroquinate dehydratase.	NF	NF	FOUND	FOUND
4401	4.2.1.101	Trans-feruloyl-CoA hydratase.	NF	NF	NF	NF
4402	4.2.1.11	Phosphopyruvate hydratase.	FOUND	FOUND	FOUND	FOUND
4403	4.2.1.2	Fumarate hydratase.	NF	NF	NF	FOUND
4404	4.2.1.20	Tryptophan synthase.	NF	NF	FOUND	FOUND
4405	4.2.1.3	Aconitate hydratase.	NF	NF	FOUND	NF
4406	4.2.1.51	Prephenate dehydratase.	NF	NF	FOUND	FOUND
4407	4.2.1.78	(S)-norcoclaurine synthase.	NF	NF	NF	NF
4408	4.2.1.91	Arogenate dehydratase.	NF	NF	NF	NF
4409	4.2.3.4	3-dehydroquinate synthase.	NF	NF	FOUND	FOUND
4410	4.2.3.5	Chorismate synthase.	NF	NF	FOUND	FOUND
4411	4.3.1.24	Phenylalanine ammonia-lyase.	NF	NF	NF	NF
4412	4.3.1.25	Phenylalanine/tyrosine ammonia-lyase.	NF	NF	NF	NF
4413	4.3.3.2	Strictosidine synthase.	NF	NF	NF	NF
4414	4.3.3.3	Deacetylisoipecoside synthase.	NF	NF	NF	NF
4415	5.3.1.24	Phosphoribosylanthranilate isomerase.	NF	NF	FOUND	FOUND
4416	5.3.1.9	Glucose-6-phosphate isomerase.	FOUND	FOUND	FOUND	FOUND
4417	5.4.2.1	Phosphoglycerate mutase.	FOUND	FOUND	FOUND	FOUND
4418	5.4.99.5	Chorismate mutase.	NF	NF	FOUND	FOUND
4419	6.2.1.12	4-coumarate--CoA ligase.	NF	NF	NF	NF
4420	6.2.1.32	Anthranilate--CoA ligase.	NF	NF	NF	NF
4421	6.2.1.4	Succinate--CoA ligase (GDP-forming).	NF	NF	NF	NF
4422	6.2.1.5	Succinate--CoA ligase (ADP-forming).	NF	NF	NF	NF
4423	>Biosynthesis of alkaloids derived from ornithine, lysine and nicotinic acid 01064					
4424	1.1.1.206	Tropinone reductase I.	NF	NF	NF	NF
4425	1.1.1.222	(R)-4-hydroxyphenyllactate dehydrogenase.	NF	NF	NF	NF
4426	1.1.1.237	Hydroxyphenylpyruvate reductase.	NF	NF	NF	NF
4427	1.1.1.37	Malate dehydrogenase.	NF	NF	NF	NF
4428	1.1.1.39	Malate dehydrogenase (decarboxylating).	NF	NF	NF	NF
4429	1.1.1.40	Malate dehydrogenase (oxaloacetate-decarboxylati	NF	NF	FOUND	NF
4430	1.1.1.41	Isocitrate dehydrogenase (NAD(+)).	NF	NF	FOUND	FOUND
4431	1.1.1.42	Isocitrate dehydrogenase (NADP(+)).	NF	NF	FOUND	FOUND
4432	1.14.11.11	Hyoscyamine (6S)-dioxygenase.	NF	NF	NF	NF
4433	1.14.11.14	6-beta-hydroxyhyoscyamine epoxidase.	NF	NF	NF	NF
4434	1.2.1.11	Aspartate-semialdehyde dehydrogenase.	FOUND	FOUND	FOUND	FOUND
4435	1.2.1.12	Glyceraldehyde-3-phosphate dehydrogenase (phos	FOUND	FOUND	FOUND	FOUND
4436	1.2.1.13	Glyceraldehyde-3-phosphate dehydrogenase (NADP	NF	NF	NF	NF
4437	1.2.1.38	N-acetyl-gamma-glutamyl-phosphate reductase.	NF	NF	FOUND	FOUND
4438	1.2.4.1	Pyruvate dehydrogenase (acetyl-transferring).	FOUND	FOUND	FOUND	FOUND
4439	1.2.4.2	Oxoglutarate dehydrogenase (succinyl-transferring)	NF	NF	NF	NF
4440	1.3.1.26	Dihydrodipicolinate reductase.	FOUND	FOUND	FOUND	FOUND
4441	1.3.5.1	Succinate dehydrogenase (ubiquinone).	NF	NF	NF	NF
4442	1.4.1.13	Glutamate synthase (NADPH).	NF	NF	NF	FOUND
4443	1.4.1.14	Glutamate synthase (NADH).	NF	NF	NF	FOUND
4444	1.4.1.18	Lysine 6-dehydrogenase.	NF	NF	NF	NF
4445	1.4.1.20	Phenylalanine dehydrogenase.	NF	NF	NF	NF
4446	1.4.1.3	Glutamate dehydrogenase (NAD(P)(+)).	NF	NF	NF	NF
4447	1.4.1.4	Glutamate dehydrogenase (NADP(+)).	NF	FOUND	NF	FOUND
4448	1.4.3.16	L-aspartate oxidase.	NF	NF	NF	NF
4449	1.4.3.21	Primary-amine oxidase.	NF	NF	FOUND	FOUND

4450	1.8.1.4	Dihydrolipoyl dehydrogenase.	FOUND	FOUND	FOUND	FOUND
4451	2.1.1.53	Putrescine N-methyltransferase.	NF	NF	NF	NF
4452	2.3.1.1	Amino-acid N-acetyltransferase.	NF	NF	FOUND	FOUND
4453	2.3.1.12	Dihydrolipoyllysine-residue acetyltransferase.	FOUND	FOUND	FOUND	FOUND
4454	2.3.1.145	Piperidine N-piperoyltransferase.	NF	NF	NF	NF
4455	2.3.1.35	Glutamate N-acetyltransferase.	NF	NF	NF	NF
4456	2.3.1.61	Dihydrolipoyllysine-residue succinyltransferase.	FOUND	NF	FOUND	NF
4457	2.3.1.71	Glycine N-benzoyltransferase.	NF	NF	NF	NF
4458	2.3.3.1	Citrate (Si)-synthase.	NF	NF	FOUND	FOUND
4459	2.3.3.8	ATP citrate synthase.	NF	NF	NF	NF
4460	2.4.2.11	Nicotinate phosphoribosyltransferase.	NF	NF	NF	NF
4461	2.4.2.19	Nicotinate-nucleotide diphosphorylase (carboxylati	FOUND	FOUND	FOUND	FOUND
4462	2.5.1.44	Homospermidine synthase.	NF	NF	NF	NF
4463	2.6.1.1	Aspartate transaminase.	FOUND	FOUND	FOUND	FOUND
4464	2.6.1.11	Acetylornithine transaminase.	NF	NF	FOUND	FOUND
4465	2.6.1.2	Alanine transaminase.	NF	NF	NF	NF
4466	2.6.1.5	Tyrosine transaminase.	NF	NF	NF	NF
4467	2.6.1.83	LL-diaminopimelate aminotransferase.	NF	NF	NF	NF
4468	2.6.1.9	Histidinol-phosphate transaminase.	NF	NF	FOUND	FOUND
4469	2.7.1.1	Hexokinase.	NF	NF	NF	NF
4470	2.7.1.11	6-phosphofructokinase.	FOUND	FOUND	FOUND	FOUND
4471	2.7.1.2	Glucokinase.	FOUND	FOUND	FOUND	FOUND
4472	2.7.1.40	Pyruvate kinase.	FOUND	FOUND	FOUND	FOUND
4473	2.7.2.3	Phosphoglycerate kinase.	FOUND	FOUND	FOUND	FOUND
4474	2.7.2.4	Aspartate kinase.	FOUND	FOUND	FOUND	FOUND
4475	2.7.2.8	Acetylglutamate kinase.	NF	NF	FOUND	FOUND
4476	2.8.3.17	Cinnamoyl-CoA:phenyllactate CoA-transferase.	NF	NF	NF	NF
4477	3.5.1.14	Aminoacylase.	FOUND	FOUND	FOUND	FOUND
4478	3.5.1.16	Acetylornithine deacetylase.	NF	NF	NF	NF
4479	3.5.1.32	Hippurate hydrolase.	NF	NF	NF	NF
4480	3.5.3.1	Arginase.	NF	NF	NF	NF
4481	4.1.1.17	Ornithine decarboxylase.	NF	NF	NF	NF
4482	4.1.1.18	Lysine decarboxylase.	NF	NF	NF	NF
4483	4.1.1.20	Diaminopimelate decarboxylase.	NF	NF	FOUND	FOUND
4484	4.1.2.13	Fructose-bisphosphate aldolase.	FOUND	FOUND	FOUND	FOUND
4485	4.2.1.11	Phosphopyruvate hydratase.	FOUND	FOUND	FOUND	FOUND
4486	4.2.1.3	Aconitate hydratase.	NF	NF	FOUND	NF
4487	4.2.1.52	Dihydrodipicolinate synthase.	FOUND	FOUND	FOUND	FOUND
4488	4.3.1.24	Phenylalanine ammonia-lyase.	NF	NF	NF	NF
4489	5.1.1.7	Diaminopimelate epimerase.	FOUND	FOUND	FOUND	FOUND
4490	5.3.1.9	Glucose-6-phosphate isomerase.	FOUND	FOUND	FOUND	FOUND
4491	5.4.2.1	Phosphoglycerate mutase.	FOUND	FOUND	FOUND	FOUND
4492	6.2.1.4	Succinate--CoA ligase (GDP-forming).	NF	NF	NF	NF
4493	6.2.1.5	Succinate--CoA ligase (ADP-forming).	NF	NF	NF	NF
4494	>Biosynthesis of alkaloids derived from histidine and purine 01065					
4495	1.1.1.205	IMP dehydrogenase.	FOUND	FOUND	FOUND	FOUND
4496	1.1.1.23	Histidinol dehydrogenase.	NF	NF	FOUND	FOUND
4497	1.1.1.37	Malate dehydrogenase.	NF	NF	NF	NF
4498	1.1.1.39	Malate dehydrogenase (decarboxylating).	NF	NF	NF	NF
4499	1.1.1.40	Malate dehydrogenase (oxaloacetate-decarboxylati	NF	NF	FOUND	NF
4500	1.1.1.41	Isocitrate dehydrogenase (NAD(+)).	NF	NF	FOUND	FOUND
4501	1.1.1.42	Isocitrate dehydrogenase (NADP(+)).	NF	NF	FOUND	FOUND
4502	1.1.1.44	Phosphogluconate dehydrogenase (decarboxylating	NF	NF	NF	NF
4503	1.1.1.49	Glucose-6-phosphate dehydrogenase.	NF	NF	NF	NF
4504	1.1.1.85	3-isopropylmalate dehydrogenase.	NF	NF	FOUND	FOUND

4505	1.1.1.86	Ketol-acid reductoisomerase.	NF	NF	FOUND	FOUND
4506	1.2.1.12	Glyceraldehyde-3-phosphate dehydrogenase (phos	FOUND	FOUND	FOUND	FOUND
4507	1.2.1.13	Glyceraldehyde-3-phosphate dehydrogenase (NADF	NF	NF	NF	NF
4508	1.2.4.1	Pyruvate dehydrogenase (acetyl-transferring).	FOUND	FOUND	FOUND	FOUND
4509	1.2.4.2	Oxoglutarate dehydrogenase (succinyl-transferring)	NF	NF	NF	NF
4510	1.3.5.1	Succinate dehydrogenase (ubiquinone).	NF	NF	NF	NF
4511	1.8.1.4	Dihydrolipoyl dehydrogenase.	FOUND	FOUND	FOUND	FOUND
4512	2.1.1.158	7-methylxanthosine synthase.	NF	NF	NF	NF
4513	2.1.1.159	Theobromine synthase.	NF	NF	NF	NF
4514	2.1.1.160	Caffeine synthase.	NF	NF	NF	NF
4515	2.1.2.3	Phosphoribosylaminoimidazolecarboxamide formyl	NF	NF	NF	NF
4516	2.2.1.6	Acetolactate synthase.	NF	NF	FOUND	FOUND
4517	2.3.1.12	Dihydrolipoyllysine-residue acetyltransferase.	FOUND	FOUND	FOUND	FOUND
4518	2.3.1.61	Dihydrolipoyllysine-residue succinyltransferase.	FOUND	NF	FOUND	NF
4519	2.3.3.1	Citrate (Si)-synthase.	NF	NF	FOUND	FOUND
4520	2.3.3.13	2-isopropylmalate synthase.	NF	NF	FOUND	FOUND
4521	2.3.3.8	ATP citrate synthase.	NF	NF	NF	NF
4522	2.4.2.-	Pentosyltransferases.	FOUND	FOUND	FOUND	FOUND
4523	2.4.2.1	Purine-nucleoside phosphorylase.	FOUND	FOUND	FOUND	FOUND
4524	2.4.2.17	ATP phosphoribosyltransferase.	NF	NF	FOUND	FOUND
4525	2.6.1.42	Branched-chain-amino-acid transaminase.	NF	NF	FOUND	FOUND
4526	2.6.1.9	Histidinol-phosphate transaminase.	NF	NF	FOUND	FOUND
4527	2.7.1.1	Hexokinase.	NF	NF	NF	NF
4528	2.7.1.11	6-phosphofructokinase.	FOUND	FOUND	FOUND	FOUND
4529	2.7.1.2	Glucokinase.	FOUND	FOUND	FOUND	FOUND
4530	2.7.1.40	Pyruvate kinase.	FOUND	FOUND	FOUND	FOUND
4531	2.7.2.3	Phosphoglycerate kinase.	FOUND	FOUND	FOUND	FOUND
4532	2.7.6.1	Ribose-phosphate diphosphokinase.	FOUND	FOUND	FOUND	FOUND
4533	3.1.1.31	6-phosphogluconolactonase.	NF	NF	NF	NF
4534	3.1.3.15	Histidinol-phosphatase.	NF	NF	FOUND	FOUND
4535	3.1.3.5	5'-nucleotidase.	FOUND	FOUND	FOUND	FOUND
4536	3.2.2.25	N-methyl nucleosidase.	NF	NF	NF	NF
4537	3.5.4.10	IMP cyclohydrolase.	NF	FOUND	FOUND	FOUND
4538	3.5.4.19	Phosphoribosyl-AMP cyclohydrolase.	NF	NF	FOUND	FOUND
4539	3.6.1.31	Phosphoribosyl-ATP diphosphatase.	NF	NF	NF	NF
4540	4.1.1.22	Histidine decarboxylase.	NF	NF	NF	NF
4541	4.1.2.13	Fructose-bisphosphate aldolase.	FOUND	FOUND	FOUND	FOUND
4542	4.1.3.-	Oxo-acid-lyases.	FOUND	FOUND	FOUND	FOUND
4543	4.2.1.11	Phosphopyruvate hydratase.	FOUND	FOUND	FOUND	FOUND
4544	4.2.1.19	Imidazoleglycerol-phosphate dehydratase.	NF	NF	FOUND	FOUND
4545	4.2.1.3	Aconitate hydratase.	NF	NF	FOUND	NF
4546	4.2.1.33	3-isopropylmalate dehydratase.	NF	NF	FOUND	FOUND
4547	4.2.1.9	Dihydroxy-acid dehydratase.	NF	NF	FOUND	FOUND
4548	4.3.2.2	Adenylosuccinate lyase.	FOUND	FOUND	FOUND	FOUND
4549	5.3.1.16	1-(5-phosphoribosyl)-5- ((5-phosphoribosylamino)m	NF	NF	FOUND	FOUND
4550	5.3.1.6	Ribose-5-phosphate isomerase.	FOUND	FOUND	FOUND	FOUND
4551	5.3.1.9	Glucose-6-phosphate isomerase.	FOUND	FOUND	FOUND	FOUND
4552	5.4.2.1	Phosphoglycerate mutase.	FOUND	FOUND	FOUND	FOUND
4553	6.2.1.4	Succinate--CoA ligase (GDP-forming).	NF	NF	NF	NF
4554	6.2.1.5	Succinate--CoA ligase (ADP-forming).	NF	NF	NF	NF
4555	6.3.4.4	Adenylosuccinate synthase.	FOUND	FOUND	FOUND	FOUND
4556	6.3.5.2	GMP synthase (glutamine-hydrolyzing).	FOUND	FOUND	FOUND	FOUND
4557	>Biosynthesis of alkaloids derived from terpenoid and polyketide 01066					
4558	1.1.1.170	Sterol-4-alpha-carboxylate 3-dehydrogenase (decar	NF	NF	NF	NF
4559	1.1.1.267	1-deoxy-D-xylulose-5-phosphate reductoisomerase.	NF	NF	NF	NF

4560	1.1.1.270	3-keto-steroid reductase.	NF	NF	NF	NF
4561	1.1.1.34	Hydroxymethylglutaryl-CoA reductase (NADPH).	FOUND	FOUND	FOUND	FOUND
4562	1.1.1.37	Malate dehydrogenase.	NF	NF	NF	NF
4563	1.1.1.38	Malate dehydrogenase (oxaloacetate-decarboxylati	NF	NF	NF	NF
4564	1.1.1.39	Malate dehydrogenase (decarboxylating).	NF	NF	NF	NF
4565	1.1.1.40	Malate dehydrogenase (oxaloacetate-decarboxylati	NF	NF	FOUND	NF
4566	1.1.1.41	Isocitrate dehydrogenase (NAD(+)).	NF	NF	FOUND	FOUND
4567	1.1.1.42	Isocitrate dehydrogenase (NADP(+)).	NF	NF	FOUND	FOUND
4568	1.14.13.15	Cholestanetriol 26-monooxygenase.	NF	NF	NF	NF
4569	1.14.13.70	Sterol 14-demethylase.	NF	NF	NF	NF
4570	1.14.13.72	Methylsterol monooxygenase.	NF	NF	NF	NF
4571	1.14.13.74	7-deoxyloganin 7-hydroxylase.	NF	NF	NF	NF
4572	1.14.21.6	Lathosterol oxidase.	NF	NF	NF	NF
4573	1.14.99.7	Squalene monooxygenase.	NF	NF	NF	NF
4574	1.17.1.2	4-hydroxy-3-methylbut-2-enyl diphosphate reducta	NF	NF	NF	FOUND
4575	1.17.7.1	(E)-4-hydroxy-3-methylbut-2-enyl-diphosphate synt	NF	NF	NF	FOUND
4576	1.2.1.12	Glyceraldehyde-3-phosphate dehydrogenase (phos	FOUND	FOUND	FOUND	FOUND
4577	1.2.1.13	Glyceraldehyde-3-phosphate dehydrogenase (NADF	NF	NF	NF	NF
4578	1.2.4.1	Pyruvate dehydrogenase (acetyl-transferring).	FOUND	FOUND	FOUND	FOUND
4579	1.2.4.2	Oxoglutarate dehydrogenase (succinyl-transferring)	NF	NF	NF	NF
4580	1.3.1.21	7-dehydrocholesterol reductase.	NF	NF	NF	NF
4581	1.3.1.70	Delta(14)-sterol reductase.	NF	NF	NF	NF
4582	1.3.1.72	Delta(24)-sterol reductase.	NF	NF	NF	NF
4583	1.3.3.9	Secologanin synthase.	NF	NF	NF	NF
4584	1.3.5.1	Succinate dehydrogenase (ubiquinone).	NF	NF	NF	NF
4585	1.5.1.-	With NAD(+) or NADP(+) as acceptor.	FOUND	FOUND	FOUND	FOUND
4586	1.8.1.4	Dihydrolipoyl dehydrogenase.	FOUND	FOUND	FOUND	FOUND
4587	2.2.1.7	1-deoxy-D-xylulose-5-phosphate synthase.	NF	NF	FOUND	FOUND
4588	2.3.1.12	Dihydrolipoyllysine-residue acetyltransferase.	FOUND	FOUND	FOUND	FOUND
4589	2.3.1.61	Dihydrolipoyllysine-residue succinyltransferase.	FOUND	NF	FOUND	NF
4590	2.3.3.1	Citrate (Si)-synthase.	NF	NF	FOUND	FOUND
4591	2.3.3.10	Hydroxymethylglutaryl-CoA synthase.	FOUND	FOUND	FOUND	NF
4592	2.3.3.8	ATP citrate synthase.	NF	NF	NF	NF
4593	2.5.1.1	Dimethylallyltranstransferase.	NF	NF	FOUND	FOUND
4594	2.5.1.10	(2E,6E)-farnesyl diphosphate synthase.	FOUND	NF	NF	NF
4595	2.5.1.21	Squalene synthase.	NF	NF	NF	NF
4596	2.5.1.29	Farnesyltranstransferase.	NF	NF	NF	NF
4597	2.7.1.1	Hexokinase.	NF	NF	NF	NF
4598	2.7.1.11	6-phosphofructokinase.	FOUND	FOUND	FOUND	FOUND
4599	2.7.1.148	4-(cytidine 5'-diphospho)-2-C-methyl-D-erythritol ki	NF	NF	NF	FOUND
4600	2.7.1.2	Glucokinase.	FOUND	FOUND	FOUND	FOUND
4601	2.7.1.36	Mevalonate kinase.	FOUND	FOUND	FOUND	NF
4602	2.7.1.40	Pyruvate kinase.	FOUND	FOUND	FOUND	FOUND
4603	2.7.2.3	Phosphoglycerate kinase.	FOUND	FOUND	FOUND	FOUND
4604	2.7.4.2	Phosphomevalonate kinase.	FOUND	FOUND	FOUND	NF
4605	2.7.7.60	2-C-methyl-D-erythritol 4-phosphate cytidylyltransf	NF	NF	NF	NF
4606	3.1.7.3	Monoterpenyl-diphosphatase.	NF	NF	NF	NF
4607	4.1.1.33	Diphosphomevalonate decarboxylase.	FOUND	FOUND	FOUND	NF
4608	4.1.2.13	Fructose-bisphosphate aldolase.	FOUND	FOUND	FOUND	FOUND
4609	4.2.1.11	Phosphopyruvate hydratase.	FOUND	FOUND	FOUND	FOUND
4610	4.2.1.2	Fumarate hydratase.	NF	NF	NF	FOUND
4611	4.2.1.3	Aconitate hydratase.	NF	NF	FOUND	NF
4612	4.6.1.12	2-C-methyl-D-erythritol 2,4-cyclodiphosphate synth	NF	NF	NF	FOUND
4613	5.3.1.9	Glucose-6-phosphate isomerase.	FOUND	FOUND	FOUND	FOUND
4614	5.3.3.2	Isopentenyl-diphosphate Delta-isomerase.	FOUND	FOUND	FOUND	FOUND

4615	5.3.3.5	Cholestenol Delta-isomerase.	NF	NF	NF	NF
4616	5.4.2.1	Phosphoglycerate mutase.	FOUND	FOUND	FOUND	FOUND
4617	5.4.99.7	Lanosterol synthase.	NF	NF	NF	NF
4618	6.2.1.4	Succinate--CoA ligase (GDP-forming).	NF	NF	NF	NF
4619	6.2.1.5	Succinate--CoA ligase (ADP-forming).	NF	NF	NF	NF
4620	>Biosynthesis of plant hormones 01070					
4621	1.1.1.145	3-beta-hydroxy-Delta(5)-steroid dehydrogenase.	NF	NF	NF	NF
4622	1.1.1.211	Long-chain-3-hydroxyacyl-CoA dehydrogenase.	NF	NF	NF	NF
4623	1.1.1.25	Shikimate dehydrogenase.	FOUND	FOUND	FOUND	FOUND
4624	1.1.1.267	1-deoxy-D-xylulose-5-phosphate reductoisomerase.	NF	NF	NF	NF
4625	1.1.1.288	Xanthoxin dehydrogenase.	NF	NF	NF	NF
4626	1.1.1.3	Homoserine dehydrogenase.	NF	NF	FOUND	FOUND
4627	1.1.1.34	Hydroxymethylglutaryl-CoA reductase (NADPH).	FOUND	FOUND	FOUND	FOUND
4628	1.1.1.35	3-hydroxyacyl-CoA dehydrogenase.	NF	NF	NF	NF
4629	1.1.1.37	Malate dehydrogenase.	NF	NF	NF	NF
4630	1.1.1.41	Isocitrate dehydrogenase (NAD(+)).	NF	NF	FOUND	FOUND
4631	1.1.1.42	Isocitrate dehydrogenase (NADP(+)).	NF	NF	FOUND	FOUND
4632	1.1.1.44	Phosphogluconate dehydrogenase (decarboxylating	NF	NF	NF	NF
4633	1.1.1.49	Glucose-6-phosphate dehydrogenase.	NF	NF	NF	NF
4634	1.13.11.12	Lipoxygenase.	NF	NF	NF	NF
4635	1.13.11.51	9-cis-epoxycarotenoid dioxygenase.	NF	NF	NF	NF
4636	1.13.12.3	Tryptophan 2-monooxygenase.	NF	NF	NF	NF
4637	1.14.-.-	Acting on paired donors, with incorporation or redu	NF	NF	NF	NF
4638	1.14.11.12	Gibberellin-44 dioxygenase.	NF	NF	NF	NF
4639	1.14.11.15	Gibberellin 3-beta-dioxygenase.	NF	NF	NF	NF
4640	1.14.13.-	With NADH or NADPH as one donor, and incorporat	NF	NF	NF	NF
4641	1.14.13.70	Sterol 14-demethylase.	NF	NF	NF	NF
4642	1.14.13.78	Ent-kaurene oxidase.	NF	NF	NF	NF
4643	1.14.13.79	Ent-kaurenoic acid oxidase.	NF	NF	NF	NF
4644	1.14.13.90	Zeaxanthin epoxidase.	NF	NF	NF	NF
4645	1.14.17.4	Aminocyclopropanecarboxylate oxidase.	NF	NF	NF	NF
4646	1.14.21.6	Lathosterol oxidase.	NF	NF	NF	NF
4647	1.14.99.-	Miscellaneous (requires further characterization).	NF	NF	NF	NF
4648	1.14.99.30	Carotene 7,8-desaturase.	NF	NF	NF	NF
4649	1.14.99.7	Squalene monooxygenase.	NF	NF	NF	NF
4650	1.17.1.2	4-hydroxy-3-methylbut-2-enyl diphosphate reducta	NF	NF	NF	FOUND
4651	1.17.7.1	(E)-4-hydroxy-3-methylbut-2-enyl-diphosphate synt	NF	NF	NF	FOUND
4652	1.2.1.11	Aspartate-semialdehyde dehydrogenase.	FOUND	FOUND	FOUND	FOUND
4653	1.2.1.12	Glyceraldehyde-3-phosphate dehydrogenase (phos	FOUND	FOUND	FOUND	FOUND
4654	1.2.1.13	Glyceraldehyde-3-phosphate dehydrogenase (NADF	NF	NF	NF	NF
4655	1.2.3.14	Abscisic-aldehyde oxidase.	NF	NF	NF	NF
4656	1.2.4.1	Pyruvate dehydrogenase (acetyl-transferring).	FOUND	FOUND	FOUND	FOUND
4657	1.2.4.2	Oxoglutarate dehydrogenase (succinyl-transferring)	NF	NF	NF	NF
4658	1.3.1.21	7-dehydrocholesterol reductase.	NF	NF	NF	NF
4659	1.3.1.42	12-oxophytodienoate reductase.	NF	NF	NF	NF
4660	1.3.1.70	Delta(14)-sterol reductase.	NF	NF	NF	NF
4661	1.3.1.72	Delta(24)-sterol reductase.	NF	NF	NF	NF
4662	1.3.3.6	Acyl-CoA oxidase.	NF	NF	NF	NF
4663	1.3.5.1	Succinate dehydrogenase (ubiquinone).	NF	NF	NF	NF
4664	1.3.99.-	With other acceptors.	NF	NF	FOUND	FOUND
4665	1.8.1.4	Dihydrolipoyl dehydrogenase.	FOUND	FOUND	FOUND	FOUND
4666	2.1.1.14	5-methyltetrahydropteroyltriglutamate--homocyste	FOUND	FOUND	FOUND	NF
4667	2.1.1.41	Sterol 24-C-methyltransferase.	NF	NF	NF	NF
4668	2.1.2.3	Phosphoribosylaminoimidazolecarboxamide formyl	NF	NF	NF	NF
4669	2.2.1.1	Transketolase.	FOUND	FOUND	FOUND	FOUND

4670	2.2.1.2	Transaldolase.	FOUND	NF	FOUND	FOUND
4671	2.2.1.7	1-deoxy-D-xylulose-5-phosphate synthase.	NF	NF	FOUND	FOUND
4672	2.3.1.12	Dihydrolipoyllysine-residue acetyltransferase.	FOUND	FOUND	FOUND	FOUND
4673	2.3.1.16	Acetyl-CoA C-acyltransferase.	NF	NF	NF	NF
4674	2.3.1.46	Homoserine O-succinyltransferase.	NF	NF	FOUND	FOUND
4675	2.3.1.61	Dihydrolipoyllysine-residue succinyltransferase.	FOUND	NF	FOUND	NF
4676	2.3.3.1	Citrate (Si)-synthase.	NF	NF	FOUND	FOUND
4677	2.3.3.10	Hydroxymethylglutaryl-CoA synthase.	FOUND	FOUND	FOUND	NF
4678	2.3.3.8	ATP citrate synthase.	NF	NF	NF	NF
4679	2.4.2.-	Pentosyltransferases.	FOUND	FOUND	FOUND	FOUND
4680	2.4.2.17	ATP phosphoribosyltransferase.	NF	NF	FOUND	FOUND
4681	2.4.2.18	Anthranilate phosphoribosyltransferase.	NF	NF	FOUND	FOUND
4682	2.5.1.1	Dimethylallyltranstransferase.	NF	NF	FOUND	FOUND
4683	2.5.1.10	(2E,6E)-farnesyl diphosphate synthase.	FOUND	NF	NF	NF
4684	2.5.1.19	3-phosphoshikimate 1-carboxyvinyltransferase.	NF	NF	FOUND	FOUND
4685	2.5.1.21	Squalene synthase.	NF	NF	NF	NF
4686	2.5.1.27	Adenylate dimethylallyltransferase.	NF	NF	NF	NF
4687	2.5.1.29	Farnesyltranstransferase.	NF	NF	NF	NF
4688	2.5.1.32	Phytoene synthase.	NF	NF	NF	NF
4689	2.5.1.48	Cystathionine gamma-synthase.	NF	NF	FOUND	NF
4690	2.5.1.54	3-deoxy-7-phosphoheptulonate synthase.	NF	NF	FOUND	FOUND
4691	2.5.1.6	Methionine adenosyltransferase.	FOUND	FOUND	FOUND	FOUND
4692	2.5.1.75	tRNA dimethylallyltransferase.	FOUND	NF	NF	NF
4693	2.6.1.1	Aspartate transaminase.	FOUND	FOUND	FOUND	FOUND
4694	2.6.1.78	Aspartate--prephenate aminotransferase.	NF	NF	NF	NF
4695	2.6.1.79	Glutamate--prephenate aminotransferase.	NF	NF	NF	NF
4696	2.7.1.1	Hexokinase.	NF	NF	NF	NF
4697	2.7.1.11	6-phosphofructokinase.	FOUND	FOUND	FOUND	FOUND
4698	2.7.1.148	4-(cytidine 5'-diphospho)-2-C-methyl-D-erythritol ki	NF	NF	NF	FOUND
4699	2.7.1.2	Glucokinase.	FOUND	FOUND	FOUND	FOUND
4700	2.7.1.36	Mevalonate kinase.	FOUND	FOUND	FOUND	NF
4701	2.7.1.40	Pyruvate kinase.	FOUND	FOUND	FOUND	FOUND
4702	2.7.1.71	Shikimate kinase.	NF	FOUND	FOUND	FOUND
4703	2.7.2.3	Phosphoglycerate kinase.	FOUND	FOUND	FOUND	FOUND
4704	2.7.2.4	Aspartate kinase.	FOUND	FOUND	FOUND	FOUND
4705	2.7.4.2	Phosphomevalonate kinase.	FOUND	FOUND	FOUND	NF
4706	2.7.6.1	Ribose-phosphate diphosphokinase.	FOUND	FOUND	FOUND	FOUND
4707	2.7.7.60	2-C-methyl-D-erythritol 4-phosphate cytidylyltransf	NF	NF	NF	NF
4708	3.1.1.31	6-phosphogluconolactonase.	NF	NF	NF	NF
4709	3.1.2.-	Thiolester hydrolases.	NF	FOUND	NF	NF
4710	3.5.1.4	Amidase.	NF	FOUND	NF	NF
4711	3.5.4.10	IMP cyclohydrolase.	NF	FOUND	FOUND	FOUND
4712	3.5.4.19	Phosphoribosyl-AMP cyclohydrolase.	NF	NF	FOUND	FOUND
4713	3.5.4.6	AMP deaminase.	NF	NF	NF	NF
4714	3.6.1.31	Phosphoribosyl-ATP diphosphatase.	NF	NF	NF	NF
4715	4.1.1.33	Diphosphomevalonate decarboxylase.	FOUND	FOUND	FOUND	NF
4716	4.1.1.48	Indole-3-glycerol-phosphate synthase.	NF	NF	FOUND	FOUND
4717	4.1.2.13	Fructose-bisphosphate aldolase.	FOUND	FOUND	FOUND	FOUND
4718	4.1.3.-	Oxo-acid-lyases.	FOUND	FOUND	FOUND	FOUND
4719	4.1.3.27	Anthranilate synthase.	NF	NF	FOUND	FOUND
4720	4.2.1.10	3-dehydroquinate dehydratase.	NF	NF	FOUND	FOUND
4721	4.2.1.11	Phosphopyruvate hydratase.	FOUND	FOUND	FOUND	FOUND
4722	4.2.1.17	Enoyl-CoA hydratase.	NF	NF	NF	NF
4723	4.2.1.2	Fumarate hydratase.	NF	NF	NF	FOUND
4724	4.2.1.20	Tryptophan synthase.	NF	NF	FOUND	FOUND

4725	4.2.1.3	Aconitate hydratase.	NF	NF	FOUND	NF
4726	4.2.1.91	Arogenate dehydratase.	NF	NF	NF	NF
4727	4.2.1.92	Hydroperoxide dehydratase.	NF	NF	NF	NF
4728	4.2.3.19	Ent-kaurene synthase.	NF	NF	NF	NF
4729	4.2.3.4	3-dehydroquinate synthase.	NF	NF	FOUND	FOUND
4730	4.2.3.5	Chorismate synthase.	NF	NF	FOUND	FOUND
4731	4.3.1.24	Phenylalanine ammonia-lyase.	NF	NF	NF	NF
4732	4.4.1.14	1-aminocyclopropane-1-carboxylate synthase.	NF	NF	NF	NF
4733	4.4.1.8	Cystathionine beta-lyase.	FOUND	NF	FOUND	NF
4734	4.6.1.12	2-C-methyl-D-erythritol 2,4-cyclodiphosphate synth	NF	NF	NF	FOUND
4735	5.3.1.16	1-(5-phosphoribosyl)-5- ((5-phosphoribosylamino)m	NF	NF	FOUND	FOUND
4736	5.3.1.24	Phosphoribosylanthranilate isomerase.	NF	NF	FOUND	FOUND
4737	5.3.1.6	Ribose-5-phosphate isomerase.	FOUND	FOUND	FOUND	FOUND
4738	5.3.1.9	Glucose-6-phosphate isomerase.	FOUND	FOUND	FOUND	FOUND
4739	5.3.3.1	Steroid Delta-isomerase.	NF	NF	NF	NF
4740	5.3.3.2	Isopentenyl-diphosphate Delta-isomerase.	FOUND	FOUND	FOUND	FOUND
4741	5.3.3.5	Cholestenol Delta-isomerase.	NF	NF	NF	NF
4742	5.3.99.6	Allene-oxide cyclase.	NF	NF	NF	NF
4743	5.3.99.9	Neoxanthin synthase.	NF	NF	NF	NF
4744	5.4.2.1	Phosphoglycerate mutase.	FOUND	FOUND	FOUND	FOUND
4745	5.4.4.2	Isochorismate synthase.	NF	NF	NF	NF
4746	5.4.99.8	Cycloartenol synthase.	NF	NF	NF	NF
4747	5.5.1.13	Ent-copalyl diphosphate synthase.	NF	NF	NF	NF
4748	5.5.1.9	Cycloeucalenol cycloisomerase.	NF	NF	NF	NF
4749	6.2.1.-	Acid--thiol ligases.	NF	NF	NF	NF
4750	6.2.1.4	Succinate--CoA ligase (GDP-forming).	NF	NF	NF	NF
4751	6.2.1.5	Succinate--CoA ligase (ADP-forming).	NF	NF	NF	NF
4752	>Metabolic pathways 01100					
4753	1.-.-.-	Oxidoreductases.	FOUND	FOUND	FOUND	FOUND
4754	1.1.-.-	Acting on the CH-OH group of donors.	FOUND	FOUND	FOUND	FOUND
4755	1.1.1.-	With NAD(+) or NADP(+) as acceptor.	FOUND	FOUND	FOUND	FOUND
4756	1.1.1.1	Alcohol dehydrogenase.	FOUND	FOUND	FOUND	FOUND
4757	1.1.1.10	L-xylulose reductase.	NF	NF	NF	NF
4758	1.1.1.100	3-oxoacyl-[acyl-carrier-protein] reductase.	NF	NF	FOUND	FOUND
4759	1.1.1.101	Acylglycerone-phosphate reductase.	NF	NF	NF	NF
4760	1.1.1.102	3-dehydrosphinganine reductase.	NF	NF	NF	NF
4761	1.1.1.12	L-arabinitol 4-dehydrogenase.	NF	NF	NF	NF
4762	1.1.1.122	D-threo-aldose 1-dehydrogenase.	NF	NF	NF	NF
4763	1.1.1.133	dTDP-4-dehydrorhamnose reductase.	NF	NF	FOUND	FOUND
4764	1.1.1.14	L-iditol 2-dehydrogenase.	NF	NF	NF	FOUND
4765	1.1.1.145	3-beta-hydroxy-Delta(5)-steroid dehydrogenase.	NF	NF	NF	NF
4766	1.1.1.146	11-beta-hydroxysteroid dehydrogenase.	NF	NF	NF	NF
4767	1.1.1.15	D-iditol 2-dehydrogenase.	NF	NF	NF	NF
4768	1.1.1.153	Sepiapterin reductase.	NF	NF	NF	NF
4769	1.1.1.156	Glycerol 2-dehydrogenase (NADP(+)).	NF	NF	NF	NF
4770	1.1.1.157	3-hydroxybutyryl-CoA dehydrogenase.	NF	NF	NF	NF
4771	1.1.1.158	UDP-N-acetylmuramate dehydrogenase.	FOUND	FOUND	FOUND	FOUND
4772	1.1.1.169	2-dehydropantoate 2-reductase.	FOUND	FOUND	NF	NF
4773	1.1.1.170	Sterol-4-alpha-carboxylate 3-dehydrogenase (decar	NF	NF	NF	NF
4774	1.1.1.178	3-hydroxy-2-methylbutyryl-CoA dehydrogenase.	NF	NF	NF	NF
4775	1.1.1.18	Inositol 2-dehydrogenase.	NF	NF	NF	NF
4776	1.1.1.181	Cholest-5-ene-3-beta,7-alpha-diol 3-beta-dehydrog	NF	NF	NF	NF
4777	1.1.1.184	Carbonyl reductase (NADPH).	NF	NF	NF	NF
4778	1.1.1.189	Prostaglandin-E(2) 9-reductase.	NF	NF	NF	NF
4779	1.1.1.19	Glucuronate reductase.	NF	NF	NF	NF

4780	1.1.1.193	5-amino-6-(5-phosphoribosylamino)uracil reductase	NF	NF	FOUND	FOUND
4781	1.1.1.195	Cinnamyl-alcohol dehydrogenase.	NF	NF	NF	NF
4782	1.1.1.2	Alcohol dehydrogenase (NADP(+)).	NF	NF	FOUND	NF
4783	1.1.1.205	IMP dehydrogenase.	FOUND	FOUND	FOUND	FOUND
4784	1.1.1.206	Tropinone reductase I.	NF	NF	NF	NF
4785	1.1.1.21	Aldehyde reductase.	NF	NF	FOUND	FOUND
4786	1.1.1.211	Long-chain-3-hydroxyacyl-CoA dehydrogenase.	NF	NF	NF	NF
4787	1.1.1.215	Gluconate 2-dehydrogenase.	NF	NF	NF	NF
4788	1.1.1.219	Dihydrokaempferol 4-reductase.	NF	NF	NF	NF
4789	1.1.1.22	UDP-glucose 6-dehydrogenase.	NF	NF	NF	FOUND
4790	1.1.1.220	6-pyruvoyltetrahydropterin 2'-reductase.	NF	NF	NF	NF
4791	1.1.1.23	Histidinol dehydrogenase.	NF	NF	FOUND	FOUND
4792	1.1.1.239	3-alpha-(17-beta)-hydroxysteroid dehydrogenase (N	NF	NF	NF	NF
4793	1.1.1.244	Methanol dehydrogenase.	NF	NF	NF	NF
4794	1.1.1.247	Codeinone reductase (NADPH).	NF	NF	NF	NF
4795	1.1.1.248	Salutaridine reductase (NADPH).	NF	NF	NF	NF
4796	1.1.1.25	Shikimate dehydrogenase.	FOUND	FOUND	FOUND	FOUND
4797	1.1.1.256	Fluoren-9-ol dehydrogenase.	NF	NF	NF	NF
4798	1.1.1.26	Glyoxylate reductase.	NF	NF	NF	NF
4799	1.1.1.262	4-hydroxythreonine-4-phosphate dehydrogenase.	NF	NF	NF	NF
4800	1.1.1.267	1-deoxy-D-xylulose-5-phosphate reductoisomerase.	NF	NF	NF	NF
4801	1.1.1.27	L-lactate dehydrogenase.	FOUND	FOUND	NF	FOUND
4802	1.1.1.270	3-keto-steroid reductase.	NF	NF	NF	NF
4803	1.1.1.271	GDP-L-fucose synthase.	NF	NF	NF	NF
4804	1.1.1.282	Quinate/shikimate dehydrogenase.	NF	NF	NF	NF
4805	1.1.1.288	Xanthoxin dehydrogenase.	NF	NF	NF	NF
4806	1.1.1.29	Glycerate dehydrogenase.	NF	NF	NF	NF
4807	1.1.1.290	4-phosphoerythronate dehydrogenase.	NF	NF	NF	NF
4808	1.1.1.294	Chlorophyll(ide) b reductase.	NF	NF	NF	NF
4809	1.1.1.3	Homoserine dehydrogenase.	NF	NF	FOUND	FOUND
4810	1.1.1.30	3-hydroxybutyrate dehydrogenase.	NF	NF	NF	FOUND
4811	1.1.1.302	2,5-diamino-6-(ribosylamino)-4(3H)-pyrimidinone 5	NF	NF	NF	NF
4812	1.1.1.31	3-hydroxyisobutyrate dehydrogenase.	NF	NF	NF	NF
4813	1.1.1.34	Hydroxymethylglutaryl-CoA reductase (NADPH).	FOUND	FOUND	FOUND	FOUND
4814	1.1.1.35	3-hydroxyacyl-CoA dehydrogenase.	NF	NF	NF	NF
4815	1.1.1.37	Malate dehydrogenase.	NF	NF	NF	NF
4816	1.1.1.39	Malate dehydrogenase (decarboxylating).	NF	NF	NF	NF
4817	1.1.1.40	Malate dehydrogenase (oxaloacetate-decarboxylati	NF	NF	FOUND	NF
4818	1.1.1.41	Isocitrate dehydrogenase (NAD(+)).	NF	NF	FOUND	FOUND
4819	1.1.1.42	Isocitrate dehydrogenase (NADP(+)).	NF	NF	FOUND	FOUND
4820	1.1.1.43	Phosphogluconate 2-dehydrogenase.	NF	NF	NF	NF
4821	1.1.1.44	Phosphogluconate dehydrogenase (decarboxylating	NF	NF	NF	NF
4822	1.1.1.45	L-gulonate 3-dehydrogenase.	NF	NF	NF	NF
4823	1.1.1.46	L-arabinose 1-dehydrogenase.	NF	NF	NF	NF
4824	1.1.1.49	Glucose-6-phosphate dehydrogenase.	NF	NF	NF	NF
4825	1.1.1.50	3-alpha-hydroxysteroid dehydrogenase (B-specific).	NF	NF	NF	NF
4826	1.1.1.51	3(or 17)-beta-hydroxysteroid dehydrogenase.	NF	NF	NF	NF
4827	1.1.1.53	3-alpha-(or 20-beta)-hydroxysteroid dehydrogenase	NF	NF	NF	NF
4828	1.1.1.56	Ribitol 2-dehydrogenase.	NF	NF	NF	NF
4829	1.1.1.57	Fructuronate reductase.	FOUND	NF	NF	NF
4830	1.1.1.58	Tagaturonate reductase.	NF	NF	NF	FOUND
4831	1.1.1.59	3-hydroxypropionate dehydrogenase.	NF	NF	NF	NF
4832	1.1.1.6	Glycerol dehydrogenase.	NF	NF	FOUND	FOUND
4833	1.1.1.60	2-hydroxy-3-oxopropionate reductase.	NF	NF	NF	NF
4834	1.1.1.62	Estradiol 17-beta-dehydrogenase.	NF	NF	NF	NF

4835	1.1.1.63	Testosterone 17-beta-dehydrogenase.	NF	NF	NF	NF
4836	1.1.1.64	Testosterone 17-beta-dehydrogenase (NADP(+)).	NF	NF	NF	NF
4837	1.1.1.65	Pyridoxine 4-dehydrogenase.	NF	NF	NF	NF
4838	1.1.1.79	Glyoxylate reductase (NADP(+)).	NF	NF	NF	NF
4839	1.1.1.81	Hydroxypyruvate reductase.	NF	NF	NF	NF
4840	1.1.1.82	Malate dehydrogenase (NADP(+)).	NF	NF	NF	NF
4841	1.1.1.85	3-isopropylmalate dehydrogenase.	NF	NF	FOUND	FOUND
4842	1.1.1.86	Ketol-acid reductoisomerase.	NF	NF	FOUND	FOUND
4843	1.1.1.87	Homoisocitrate dehydrogenase.	NF	NF	NF	NF
4844	1.1.1.9	D-xylulose reductase.	NF	NF	NF	NF
4845	1.1.1.90	Aryl-alcohol dehydrogenase.	NF	NF	NF	NF
4846	1.1.1.95	Phosphoglycerate dehydrogenase.	FOUND	NF	FOUND	FOUND
4847	1.1.2.3	L-lactate dehydrogenase (cytochrome).	NF	NF	NF	NF
4848	1.1.2.7	Methanol dehydrogenase (cytochrome c).	NF	NF	NF	NF
4849	1.1.2.8	Alcohol dehydrogenase (cytochrome c).	NF	NF	NF	NF
4850	1.1.3.13	Alcohol oxidase.	NF	NF	NF	NF
4851	1.1.3.15	(S)-2-hydroxy-acid oxidase.	NF	NF	NF	FOUND
4852	1.1.3.4	Glucose oxidase.	NF	NF	NF	NF
4853	1.1.3.5	Hexose oxidase.	NF	NF	NF	NF
4854	1.1.3.8	L-gulonolactone oxidase.	NF	NF	NF	NF
4855	1.1.5.2	Quinoprotein glucose dehydrogenase.	NF	NF	NF	NF
4856	1.1.5.8	Quinate dehydrogenase (quinone).	NF	NF	NF	NF
4857	1.1.99.10	Glucose dehydrogenase (acceptor).	NF	NF	NF	NF
4858	1.1.99.14	Glycolate dehydrogenase.	NF	NF	NF	NF
4859	1.1.99.3	Gluconate 2-dehydrogenase (acceptor).	NF	NF	NF	NF
4860	1.1.99.7	Lactate--malate transhydrogenase.	NF	NF	NF	NF
4861	1.10.2.-	With a cytochrome as acceptor.	NF	NF	NF	NF
4862	1.10.2.2	Ubiquinol--cytochrome-c reductase.	NF	NF	NF	NF
4863	1.10.3.-	With oxygen as acceptor.	NF	NF	NF	NF
4864	1.10.3.1	Catechol oxidase.	NF	NF	NF	NF
4865	1.10.3.3	L-ascorbate oxidase.	NF	NF	NF	NF
4866	1.10.99.3	Violaxanthin de-epoxidase.	NF	NF	NF	NF
4867	1.11.1.15	Peroxiredoxin.	NF	NF	NF	NF
4868	1.11.1.6	Catalase.	NF	NF	NF	NF
4869	1.11.1.7	Peroxidase.	FOUND	FOUND	FOUND	FOUND
4870	1.11.1.8	Iodide peroxidase.	NF	NF	NF	NF
4871	1.12.98.1	Coenzyme F420 hydrogenase.	NF	NF	NF	NF
4872	1.12.98.2	5,10-methenyltetrahydromethanopterin hydrogena	NF	NF	NF	NF
4873	1.13.11.-	With incorporation of two atoms of oxygen.	NF	NF	NF	NF
4874	1.13.11.1	Catechol 1,2-dioxygenase.	NF	NF	NF	NF
4875	1.13.11.11	Tryptophan 2,3-dioxygenase.	NF	NF	NF	NF
4876	1.13.11.12	Lipoxygenase.	NF	NF	NF	NF
4877	1.13.11.19	Cysteamine dioxygenase.	NF	NF	NF	NF
4878	1.13.11.2	Catechol 2,3-dioxygenase.	NF	NF	NF	NF
4879	1.13.11.20	Cysteine dioxygenase.	NF	NF	NF	NF
4880	1.13.11.27	4-hydroxyphenylpyruvate dioxygenase.	NF	NF	NF	NF
4881	1.13.11.3	Protocatechuate 3,4-dioxygenase.	NF	NF	NF	NF
4882	1.13.11.33	Arachidonate 15-lipoxygenase.	NF	NF	NF	NF
4883	1.13.11.34	Arachidonate 5-lipoxygenase.	NF	NF	NF	NF
4884	1.13.11.37	Hydroxyquinol 1,2-dioxygenase.	NF	NF	NF	NF
4885	1.13.11.39	Biphenyl-2,3-diol 1,2-dioxygenase.	NF	NF	NF	NF
4886	1.13.11.4	Gentisate 1,2-dioxygenase.	NF	NF	NF	NF
4887	1.13.11.5	Homogentisate 1,2-dioxygenase.	NF	NF	NF	NF
4888	1.13.11.51	9-cis-epoxycarotenoid dioxygenase.	NF	NF	NF	NF
4889	1.13.11.52	Indoleamine 2,3-dioxygenase.	NF	NF	NF	NF

4890	1.13.11.53	Acireductone dioxygenase (Ni(2+)-requiring).	NF	NF	NF	NF
4891	1.13.11.54	Acireductone dioxygenase (Fe(2+)-requiring).	NF	NF	NF	NF
4892	1.13.11.56	1,2-dihydroxynaphthalene dioxygenase.	NF	NF	NF	NF
4893	1.13.11.6	3-hydroxyanthranilate 3,4-dioxygenase.	NF	NF	NF	NF
4894	1.13.12.-	With incorporation of one atom of oxygen.	NF	NF	FOUND	NF
4895	1.13.12.14	Chlorophyllide-a oxygenase.	NF	NF	NF	NF
4896	1.14.-.-	Acting on paired donors, with incorporation or redu	NF	NF	NF	NF
4897	1.14.11.11	Hyoscyamine (6S)-dioxygenase.	NF	NF	NF	NF
4898	1.14.11.12	Gibberellin-44 dioxygenase.	NF	NF	NF	NF
4899	1.14.11.14	6-beta-hydroxyhyoscyamine epoxidase.	NF	NF	NF	NF
4900	1.14.11.15	Gibberellin 3-beta-dioxygenase.	NF	NF	NF	NF
4901	1.14.11.19	Leucocyanidin oxygenase.	NF	NF	NF	NF
4902	1.14.11.2	Procollagen-proline dioxygenase.	NF	NF	NF	NF
4903	1.14.11.22	Flavone synthase.	NF	NF	NF	NF
4904	1.14.11.23	Flavonol synthase.	NF	NF	NF	NF
4905	1.14.11.26	Deacetoxycephalosporin-C hydroxylase.	NF	NF	NF	NF
4906	1.14.11.9	Flavanone 3-dioxygenase.	NF	NF	NF	NF
4907	1.14.12.-	With NADH or NADPH as one donor, and incorporat	NF	NF	NF	NF
4908	1.14.12.10	Benzoate 1,2-dioxygenase.	NF	NF	NF	NF
4909	1.14.12.12	Naphthalene 1,2-dioxygenase.	NF	NF	NF	NF
4910	1.14.12.18	Biphenyl 2,3-dioxygenase.	NF	NF	NF	NF
4911	1.14.12.7	Phthalate 4,5-dioxygenase.	NF	NF	NF	NF
4912	1.14.13.-	With NADH or NADPH as one donor, and incorporat	NF	NF	NF	NF
4913	1.14.13.1	Salicylate 1-monooxygenase.	NF	NF	NF	NF
4914	1.14.13.108	Abietadiene hydroxylase.	NF	NF	NF	NF
4915	1.14.13.109	Abietadienol hydroxylase.	NF	NF	NF	NF
4916	1.14.13.11	Trans-cinnamate 4-monooxygenase.	NF	NF	NF	NF
4917	1.14.13.12	Benzoate 4-monooxygenase.	NF	NF	NF	NF
4918	1.14.13.13	Calcidiol 1-monooxygenase.	NF	NF	NF	NF
4919	1.14.13.15	Cholestanetriol 26-monooxygenase.	NF	NF	NF	NF
4920	1.14.13.17	Cholesterol 7-alpha-monooxygenase.	NF	NF	NF	NF
4921	1.14.13.2	4-hydroxybenzoate 3-monooxygenase.	NF	NF	NF	NF
4922	1.14.13.20	2,4-dichlorophenol 6-monooxygenase.	NF	NF	NF	NF
4923	1.14.13.21	Flavonoid 3'-monooxygenase.	NF	NF	NF	NF
4924	1.14.13.25	Methane monooxygenase.	NF	NF	NF	NF
4925	1.14.13.30	Leukotriene-B(4) 20-monooxygenase.	NF	NF	NF	NF
4926	1.14.13.33	4-hydroxybenzoate 3-monooxygenase (NAD(P)H).	NF	NF	NF	NF
4927	1.14.13.37	Methyltetrahydroprotoberberine 14-monooxygena	NF	NF	NF	NF
4928	1.14.13.39	Nitric-oxide synthase.	NF	NF	NF	NF
4929	1.14.13.50	Pentachlorophenol monooxygenase.	NF	NF	NF	NF
4930	1.14.13.55	Protopine 6-monooxygenase.	NF	NF	NF	NF
4931	1.14.13.56	Dihydrosanguinarine 10-monooxygenase.	NF	NF	NF	NF
4932	1.14.13.57	Dihydrochelirubine 12-monooxygenase.	NF	NF	NF	NF
4933	1.14.13.69	Alkene monooxygenase.	NF	NF	NF	NF
4934	1.14.13.7	Phenol 2-monooxygenase.	NF	NF	NF	NF
4935	1.14.13.70	Sterol 14-demethylase.	NF	NF	NF	NF
4936	1.14.13.71	N-methylcoclaurine 3'-monooxygenase.	NF	NF	NF	NF
4937	1.14.13.72	Methylsterol monooxygenase.	NF	NF	NF	NF
4938	1.14.13.74	7-deoxyloganin 7-hydroxylase.	NF	NF	NF	NF
4939	1.14.13.77	Taxane 13-alpha-hydroxylase.	NF	NF	NF	NF
4940	1.14.13.78	Ent-kaurene oxidase.	NF	NF	NF	NF
4941	1.14.13.79	Ent-kaurenoic acid oxidase.	NF	NF	NF	NF
4942	1.14.13.81	Magnesium-protoporphyrin IX monomethyl ester (c	NF	NF	NF	NF
4943	1.14.13.83	Precorrin-3B synthase.	NF	NF	NF	NF
4944	1.14.13.84	4-hydroxyacetophenone monooxygenase.	NF	NF	NF	NF

4945	1.14.13.9	Kynurenine 3-monooxygenase.	NF	NF	NF	NF
4946	1.14.13.90	Zeaxanthin epoxidase.	NF	NF	NF	NF
4947	1.14.13.95	7-alpha-hydroxycholest-4-en-3-one 12-alpha-hydro:	NF	NF	NF	NF
4948	1.14.14.1	Unspecific monooxygenase.	NF	NF	NF	NF
4949	1.14.15.-	With a reduced iron-sulfur protein as one donor, an	NF	NF	NF	NF
4950	1.14.15.3	Alkane 1-monooxygenase.	NF	NF	NF	NF
4951	1.14.15.4	Steroid 11-beta-monooxygenase.	NF	NF	NF	NF
4952	1.14.15.5	Corticosterone 18-monooxygenase.	NF	NF	NF	NF
4953	1.14.15.6	Cholesterol monooxygenase (side-chain-cleaving).	NF	NF	NF	NF
4954	1.14.16.1	Phenylalanine 4-monooxygenase.	NF	NF	NF	NF
4955	1.14.16.2	Tyrosine 3-monooxygenase.	NF	NF	NF	NF
4956	1.14.16.4	Tryptophan 5-monooxygenase.	NF	NF	NF	NF
4957	1.14.17.1	Dopamine beta-monooxygenase.	NF	NF	NF	NF
4958	1.14.17.4	Aminocyclopropanecarboxylate oxidase.	NF	NF	NF	NF
4959	1.14.18.-	With another compound as one donor, and incorpo	NF	NF	NF	NF
4960	1.14.18.1	Monophenol monooxygenase.	NF	NF	NF	NF
4961	1.14.19.3	Linoleoyl-CoA desaturase.	NF	NF	NF	NF
4962	1.14.20.1	Deacetoxycephalosporin-C synthase.	NF	NF	NF	NF
4963	1.14.21.1	(S)-stylopine synthase.	NF	NF	NF	NF
4964	1.14.21.2	(S)-cheilanthifoline synthase.	NF	NF	NF	NF
4965	1.14.21.4	Salutaridine synthase.	NF	NF	NF	NF
4966	1.14.21.6	Lathosterol oxidase.	NF	NF	NF	NF
4967	1.14.99.-	Miscellaneous (requires further characterization).	NF	NF	NF	NF
4968	1.14.99.1	Prostaglandin-endoperoxide synthase.	NF	NF	NF	NF
4969	1.14.99.10	Steroid 21-monooxygenase.	NF	NF	NF	NF
4970	1.14.99.19	Plasmanylethanolamine desaturase.	NF	NF	NF	NF
4971	1.14.99.30	Carotene 7,8-desaturase.	NF	NF	NF	NF
4972	1.14.99.36	Beta-carotene 15,15'-monooxygenase.	NF	NF	NF	NF
4973	1.14.99.37	Taxadiene 5-alpha-hydroxylase.	NF	NF	NF	NF
4974	1.14.99.40	5,6-dimethylbenzimidazole synthase.	NF	NF	NF	NF
4975	1.14.99.7	Squalene monooxygenase.	NF	NF	NF	NF
4976	1.14.99.9	Steroid 17-alpha-monooxygenase.	NF	NF	NF	NF
4977	1.16.8.1	Cob(II)yrinic acid a,c-diamide reductase.	NF	NF	NF	NF
4978	1.17.1.2	4-hydroxy-3-methylbut-2-enyl diphosphate reducta	NF	NF	NF	FOUND
4979	1.17.1.4	Xanthine dehydrogenase.	NF	NF	NF	FOUND
4980	1.17.3.2	Xanthine oxidase.	NF	NF	NF	NF
4981	1.17.4.1	Ribonucleoside-diphosphate reductase.	FOUND	FOUND	FOUND	FOUND
4982	1.17.4.2	Ribonucleoside-triphosphate reductase.	FOUND	FOUND	FOUND	FOUND
4983	1.17.7.1	(E)-4-hydroxy-3-methylbut-2-enyl-diphosphate synt	NF	NF	NF	FOUND
4984	1.17.99.1	4-cresol dehydrogenase (hydroxylating).	NF	NF	NF	NF
4985	1.17.99.2	Ethylbenzene hydroxylase.	NF	NF	NF	NF
4986	1.17.99.3	3-alpha,7-alpha,12-alpha-trihydroxy-5-beta-cholest	NF	NF	NF	NF
4987	1.18.-.-	Acting on iron-sulfur proteins as donors.	NF	NF	NF	NF
4988	1.18.1.2	Ferredoxin--NADP(+) reductase.	NF	NF	NF	NF
4989	1.18.6.1	Nitrogenase.	NF	NF	NF	NF
4990	1.19.6.1	Nitrogenase (flavodoxin).	NF	NF	NF	NF
4991	1.2.1.-	With NAD(+) or NADP(+) as acceptor.	FOUND	FOUND	FOUND	FOUND
4992	1.2.1.10	Acetaldehyde dehydrogenase (acetylating).	NF	NF	NF	NF
4993	1.2.1.11	Aspartate-semialdehyde dehydrogenase.	FOUND	FOUND	FOUND	FOUND
4994	1.2.1.12	Glyceraldehyde-3-phosphate dehydrogenase (phos	FOUND	FOUND	FOUND	FOUND
4995	1.2.1.13	Glyceraldehyde-3-phosphate dehydrogenase (NADI	NF	NF	NF	NF
4996	1.2.1.16	Succinate-semialdehyde dehydrogenase (NAD(P)(+)	NF	NF	NF	NF
4997	1.2.1.17	Glyoxylate dehydrogenase (acylating).	NF	NF	NF	NF
4998	1.2.1.18	Malonate-semialdehyde dehydrogenase (acetylatin	NF	NF	NF	NF
4999	1.2.1.2	Formate dehydrogenase.	NF	NF	NF	FOUND

5000	1.2.1.24	Succinate-semialdehyde dehydrogenase (NAD(+)).	NF	NF	NF	NF
5001	1.2.1.27	Methylmalonate-semialdehyde dehydrogenase (acy	NF	NF	NF	FOUND
5002	1.2.1.28	Benzaldehyde dehydrogenase (NAD(+)).	NF	NF	NF	NF
5003	1.2.1.29	Aryl-aldehyde dehydrogenase.	NF	NF	NF	NF
5004	1.2.1.3	Aldehyde dehydrogenase (NAD(+)).	NF	NF	FOUND	FOUND
5005	1.2.1.31	L-aminoadipate-semialdehyde dehydrogenase.	NF	NF	NF	NF
5006	1.2.1.32	Aminomuconate-semialdehyde dehydrogenase.	NF	NF	NF	NF
5007	1.2.1.36	Retinal dehydrogenase.	NF	NF	NF	NF
5008	1.2.1.38	N-acetyl-gamma-glutamyl-phosphate reductase.	NF	NF	FOUND	FOUND
5009	1.2.1.39	Phenylacetaldehyde dehydrogenase.	NF	NF	NF	NF
5010	1.2.1.4	Aldehyde dehydrogenase (NADP(+)).	NF	NF	NF	NF
5011	1.2.1.41	Glutamate-5-semialdehyde dehydrogenase.	NF	NF	FOUND	FOUND
5012	1.2.1.43	Formate dehydrogenase (NADP(+)).	NF	NF	NF	NF
5013	1.2.1.44	Cinnamoyl-CoA reductase.	NF	NF	NF	NF
5014	1.2.1.46	Formaldehyde dehydrogenase.	NF	NF	NF	NF
5015	1.2.1.5	Aldehyde dehydrogenase (NAD(P)(+)).	NF	NF	NF	NF
5016	1.2.1.54	Gamma-guanidinobutyraldehyde dehydrogenase.	NF	NF	NF	NF
5017	1.2.1.59	Glyceraldehyde-3-phosphate dehydrogenase (NAD(	NF	NF	NF	NF
5018	1.2.1.63	6-oxohexanoate dehydrogenase.	NF	NF	NF	NF
5019	1.2.1.65	Salicylaldehyde dehydrogenase.	NF	NF	NF	NF
5020	1.2.1.7	Benzaldehyde dehydrogenase (NADP(+)).	NF	NF	NF	NF
5021	1.2.1.70	Glutamyl-tRNA reductase.	NF	NF	NF	NF
5022	1.2.1.72	Erythrose-4-phosphate dehydrogenase.	NF	NF	NF	NF
5023	1.2.1.74	Abietadienal dehydrogenase.	NF	NF	NF	NF
5024	1.2.1.9	Glyceraldehyde-3-phosphate dehydrogenase (NADP	FOUND	FOUND	FOUND	FOUND
5025	1.2.3.1	Aldehyde oxidase.	NF	NF	NF	NF
5026	1.2.3.11	Retinal oxidase.	NF	NF	NF	NF
5027	1.2.3.14	Abscisic-aldehyde oxidase.	NF	NF	NF	NF
5028	1.2.3.3	Pyruvate oxidase.	NF	NF	NF	NF
5029	1.2.3.5	Glyoxylate oxidase.	NF	NF	NF	NF
5030	1.2.3.8	Pyridoxal oxidase.	NF	NF	NF	NF
5031	1.2.4.1	Pyruvate dehydrogenase (acetyl-transferring).	FOUND	FOUND	FOUND	FOUND
5032	1.2.4.2	Oxoglutarate dehydrogenase (succinyl-transferring)	NF	NF	NF	NF
5033	1.2.4.4	3-methyl-2-oxobutanoate dehydrogenase (2-methy	NF	NF	NF	FOUND
5034	1.2.7.1	Pyruvate synthase.	NF	NF	NF	NF
5035	1.2.7.3	2-oxoglutarate synthase.	NF	NF	NF	NF
5036	1.2.7.5	Aldehyde ferredoxin oxidoreductase.	NF	NF	NF	NF
5037	1.2.7.6	Glyceraldehyde-3-phosphate dehydrogenase (ferre	NF	NF	NF	NF
5038	1.2.7.7	3-methyl-2-oxobutanoate dehydrogenase (ferredox	NF	NF	NF	NF
5039	1.2.99.5	Formylmethanofuran dehydrogenase.	NF	NF	NF	NF
5040	1.21.3.1	Isopenicillin-N synthase.	NF	NF	NF	NF
5041	1.21.3.3	Reticuline oxidase.	NF	NF	NF	NF
5042	1.3.-.-	Acting on the CH-CH group of donors.	FOUND	FOUND	FOUND	FOUND
5043	1.3.1.-	With NAD(+) or NADP(+) as acceptor.	FOUND	FOUND	FOUND	FOUND
5044	1.3.1.1	Dihydrouracil dehydrogenase (NAD(+)).	NF	NF	NF	NF
5045	1.3.1.12	Prephenate dehydrogenase.	NF	NF	NF	NF
5046	1.3.1.13	Prephenate dehydrogenase (NADP(+)).	NF	NF	FOUND	FOUND
5047	1.3.1.14	Orotate reductase (NADH).	NF	NF	NF	NF
5048	1.3.1.19	Cis-1,2-dihydrobenzene-1,2-diol dehydrogenase.	NF	NF	NF	NF
5049	1.3.1.2	Dihydropyrimidine dehydrogenase (NADP(+)).	NF	NF	NF	NF
5050	1.3.1.21	7-dehydrocholesterol reductase.	NF	NF	NF	NF
5051	1.3.1.25	1,6-dihydroxycyclohexa-2,4-diene-1-carboxylate de	NF	NF	NF	NF
5052	1.3.1.26	Dihydrodipicolinate reductase.	FOUND	FOUND	FOUND	FOUND
5053	1.3.1.29	Cis-1,2-dihydro-1,2-dihydroxynaphthalene dehydro	NF	NF	NF	NF
5054	1.3.1.3	Delta(4)-3-oxosteroid 5-beta-reductase.	NF	NF	NF	NF

5055	1.3.1.32	Maleylacetate reductase.	NF	NF	NF	NF
5056	1.3.1.33	Protochlorophyllide reductase.	NF	NF	NF	NF
5057	1.3.1.38	Trans-2-enoyl-CoA reductase (NADPH).	NF	NF	NF	NF
5058	1.3.1.42	12-oxophytodienoate reductase.	NF	NF	NF	NF
5059	1.3.1.43	Arogenate dehydrogenase.	NF	NF	NF	NF
5060	1.3.1.44	Trans-2-enoyl-CoA reductase (NAD(+)).	NF	NF	NF	NF
5061	1.3.1.49	Cis-3,4-dihydrophenanthrene-3,4-diol dehydrogena	NF	NF	NF	NF
5062	1.3.1.54	Precorrin-6A reductase.	NF	NF	FOUND	FOUND
5063	1.3.1.56	Cis-2,3-dihydrobiphenyl-2,3-diol dehydrogenase.	NF	NF	NF	NF
5064	1.3.1.63	2,4-dichlorobenzoyl-CoA reductase.	NF	NF	NF	NF
5065	1.3.1.64	Phthalate 4,5-cis-dihydrodiol dehydrogenase.	NF	NF	NF	NF
5066	1.3.1.66	Cis-dihydroethylcatechol dehydrogenase.	NF	NF	NF	NF
5067	1.3.1.67	Cis-1,2-dihydroxy-4-methylcyclohexa-3,5-diene-1-ca	NF	NF	NF	NF
5068	1.3.1.68	1,2-dihydroxy-6-methylcyclohexa-3,5-dienecarboxy	NF	NF	NF	NF
5069	1.3.1.70	Delta(14)-sterol reductase.	NF	NF	NF	NF
5070	1.3.1.71	Delta(24(24(1)))-sterol reductase.	NF	NF	NF	NF
5071	1.3.1.72	Delta(24)-sterol reductase.	NF	NF	NF	NF
5072	1.3.1.75	Divinyl chlorophyllide a 8-vinyl-reductase.	NF	NF	NF	NF
5073	1.3.1.76	Precorrin-2 dehydrogenase.	NF	NF	NF	NF
5074	1.3.1.8	Acyl-CoA dehydrogenase (NADP(+)).	NF	NF	NF	NF
5075	1.3.1.83	Geranylgeranyl diphosphate reductase.	NF	NF	NF	NF
5076	1.3.1.9	Enoyl-[acyl-carrier-protein] reductase (NADH).	NF	NF	NF	NF
5077	1.3.2.3	L-galactonolactone dehydrogenase.	NF	NF	NF	NF
5078	1.3.3.-	With oxygen as acceptor.	FOUND	FOUND	FOUND	FOUND
5079	1.3.3.1	Dihydroorotate oxidase.	NF	NF	NF	FOUND
5080	1.3.3.12	L-galactonolactone oxidase.	NF	NF	NF	NF
5081	1.3.3.3	Coproporphyrinogen oxidase.	FOUND	FOUND	FOUND	FOUND
5082	1.3.3.4	Protoporphyrinogen oxidase.	NF	NF	NF	NF
5083	1.3.3.6	Acyl-CoA oxidase.	NF	NF	NF	NF
5084	1.3.3.9	Secologanin synthase.	NF	NF	NF	NF
5085	1.3.5.1	Succinate dehydrogenase (ubiquinone).	NF	NF	NF	NF
5086	1.3.5.2	Dihydroorotate dehydrogenase.	NF	NF	FOUND	FOUND
5087	1.3.99.-	With other acceptors.	NF	NF	FOUND	FOUND
5088	1.3.99.1	Succinate dehydrogenase.	NF	NF	NF	NF
5089	1.3.99.12	2-methylacyl-CoA dehydrogenase.	NF	NF	NF	NF
5090	1.3.99.13	Long-chain-acyl-CoA dehydrogenase.	NF	NF	NF	NF
5091	1.3.99.15	Benzoyl-CoA reductase.	NF	NF	NF	NF
5092	1.3.99.2	Butyryl-CoA dehydrogenase.	NF	NF	NF	NF
5093	1.3.99.20	4-hydroxybenzoyl-CoA reductase.	NF	NF	NF	NF
5094	1.3.99.21	(R)-benzylsuccinyl-CoA dehydrogenase.	NF	NF	NF	NF
5095	1.3.99.3	Acyl-CoA dehydrogenase.	NF	NF	FOUND	FOUND
5096	1.3.99.6	3-oxo-5-beta-steroid 4-dehydrogenase.	NF	NF	NF	NF
5097	1.3.99.7	Glutaryl-CoA dehydrogenase.	NF	NF	NF	NF
5098	1.4.1.1	Alanine dehydrogenase.	NF	NF	NF	NF
5099	1.4.1.13	Glutamate synthase (NADPH).	NF	NF	NF	FOUND
5100	1.4.1.14	Glutamate synthase (NADH).	NF	NF	NF	FOUND
5101	1.4.1.18	Lysine 6-dehydrogenase.	NF	NF	NF	NF
5102	1.4.1.2	Glutamate dehydrogenase.	NF	NF	FOUND	NF
5103	1.4.1.20	Phenylalanine dehydrogenase.	NF	NF	NF	NF
5104	1.4.1.21	Aspartate dehydrogenase.	NF	NF	NF	NF
5105	1.4.1.3	Glutamate dehydrogenase (NAD(P)(+)).	NF	NF	NF	NF
5106	1.4.1.4	Glutamate dehydrogenase (NADP(+)).	NF	FOUND	NF	FOUND
5107	1.4.1.9	Leucine dehydrogenase.	NF	NF	NF	NF
5108	1.4.2.1	Glycine dehydrogenase (cytochrome).	NF	NF	NF	NF
5109	1.4.3.-	With oxygen as acceptor.	NF	NF	FOUND	FOUND

5110	1.4.3.14	L-lysine oxidase.	NF	NF	NF	NF
5111	1.4.3.16	L-aspartate oxidase.	NF	NF	NF	NF
5112	1.4.3.2	L-amino-acid oxidase.	NF	NF	NF	NF
5113	1.4.3.21	Primary-amine oxidase.	NF	NF	FOUND	FOUND
5114	1.4.3.3	D-amino-acid oxidase.	NF	NF	NF	NF
5115	1.4.3.4	Monoamine oxidase.	NF	NF	NF	NF
5116	1.4.3.5	Pyridoxal 5'-phosphate synthase.	NF	NF	FOUND	NF
5117	1.4.4.2	Glycine dehydrogenase (decarboxylating).	NF	NF	NF	NF
5118	1.4.99.4	Aralkylamine dehydrogenase.	NF	NF	NF	NF
5119	1.4.99.5	Glycine dehydrogenase (cyanide-forming).	NF	NF	NF	NF
5120	1.5.-.-	Acting on the CH-NH group of donors.	FOUND	FOUND	FOUND	FOUND
5121	1.5.1.-	With NAD(+) or NADP(+) as acceptor.	FOUND	FOUND	FOUND	FOUND
5122	1.5.1.1	Pyrroline-2-carboxylate reductase.	NF	NF	NF	NF
5123	1.5.1.10	Saccharopine dehydrogenase (NADP(+), L-glutamat	NF	NF	NF	NF
5124	1.5.1.12	1-pyrroline-5-carboxylate dehydrogenase.	NF	NF	NF	NF
5125	1.5.1.15	Methylenetetrahydrofolate dehydrogenase (NAD(+	NF	NF	NF	NF
5126	1.5.1.18	Ephedrine dehydrogenase.	NF	NF	NF	NF
5127	1.5.1.2	Pyrroline-5-carboxylate reductase.	NF	NF	FOUND	FOUND
5128	1.5.1.20	Methylenetetrahydrofolate reductase (NAD(P)H).	NF	NF	FOUND	FOUND
5129	1.5.1.21	Delta(1)-piperideine-2-carboxylate reductase.	NF	NF	NF	NF
5130	1.5.1.27	1,2-dehydroreticulinium reductase (NADPH).	NF	NF	NF	NF
5131	1.5.1.29	FMN reductase.	NF	NF	NF	FOUND
5132	1.5.1.3	Dihydrofolate reductase.	FOUND	FOUND	FOUND	FOUND
5133	1.5.1.34	6,7-dihydropteridine reductase.	NF	NF	NF	FOUND
5134	1.5.1.5	Methylenetetrahydrofolate dehydrogenase (NADP(	FOUND	NF	NF	FOUND
5135	1.5.1.7	Saccharopine dehydrogenase (NAD(+), L-lysine-form	NF	NF	FOUND	NF
5136	1.5.1.8	Saccharopine dehydrogenase (NADP(+), L-lysine-for	NF	NF	NF	NF
5137	1.5.1.9	Saccharopine dehydrogenase (NAD(+), L-glutamate-	NF	NF	NF	NF
5138	1.5.3.1	Sarcosine oxidase.	NF	NF	NF	NF
5139	1.5.3.12	Dihydrobenzophenanthridine oxidase.	NF	NF	NF	NF
5140	1.5.3.7	L-pipecolate oxidase.	NF	NF	NF	NF
5141	1.5.7.1	Methylenetetrahydrofolate reductase (ferredoxin).	NF	NF	NF	NF
5142	1.5.99.1	Sarcosine dehydrogenase.	NF	NF	NF	NF
5143	1.5.99.11	5,10-methylenetetrahydromethanopterin reductase	NF	NF	NF	NF
5144	1.5.99.2	Dimethylglycine dehydrogenase.	NF	NF	NF	NF
5145	1.5.99.3	L-pipecolate dehydrogenase.	NF	NF	NF	NF
5146	1.5.99.8	Proline dehydrogenase.	NF	NF	NF	NF
5147	1.5.99.9	Methylenetetrahydromethanopterin dehydrogenas	NF	NF	NF	NF
5148	1.6.1.1	NAD(P)(+) transhydrogenase (B-specific).	NF	NF	NF	NF
5149	1.6.1.2	NAD(P)(+) transhydrogenase (AB-specific).	NF	NF	NF	NF
5150	1.6.5.3	NADH dehydrogenase (ubiquinone).	NF	NF	NF	FOUND
5151	1.6.5.4	Monodehydroascorbate reductase (NADH).	NF	NF	NF	NF
5152	1.6.5.7	2-hydroxy-1,4-benzoquinone reductase.	NF	NF	NF	NF
5153	1.6.99.3	NADH dehydrogenase.	FOUND	NF	FOUND	FOUND
5154	1.7.3.3	Factor independent urate hydroxylase.	NF	NF	NF	NF
5155	1.8.1.12	Trypanothione-disulfide reductase.	NF	NF	NF	NF
5156	1.8.1.2	Sulfite reductase (NADPH).	NF	NF	NF	NF
5157	1.8.1.3	Hypotaurine dehydrogenase.	NF	NF	NF	NF
5158	1.8.1.4	Dihydrolipoyl dehydrogenase.	FOUND	FOUND	FOUND	FOUND
5159	1.8.4.8	Phosphoadenylyl-sulfate reductase (thioredoxin).	NF	NF	NF	NF
5160	1.8.5.1	Glutathione dehydrogenase (ascorbate).	NF	NF	NF	NF
5161	1.8.7.1	Sulfite reductase (ferredoxin).	NF	NF	NF	NF
5162	1.8.98.1	CoB--CoM heterodisulfide reductase.	NF	NF	NF	NF
5163	1.8.99.1	Sulfite reductase.	NF	NF	NF	NF
5164	1.8.99.2	Adenylyl-sulfate reductase.	NF	NF	FOUND	NF

5165	1.9.3.-	With oxygen as acceptor.	NF	NF	NF	NF
5166	1.9.3.1	Cytochrome-c oxidase.	NF	NF	NF	NF
5167	1.97.1.-	Sole sub-subclass for oxidoreductases that do not b	NF	NF	FOUND	NF
5168	1.97.1.8	Tetrachloroethene reductive dehalogenase.	NF	NF	NF	NF
5169	2.-.-.-	Transferases.	FOUND	FOUND	FOUND	FOUND
5170	2.1.1.-	Methyltransferases.	FOUND	FOUND	FOUND	FOUND
5171	2.1.1.1	Nicotinamide N-methyltransferase.	NF	NF	NF	NF
5172	2.1.1.10	Homocysteine S-methyltransferase.	NF	NF	NF	NF
5173	2.1.1.104	Caffeoyl-CoA O-methyltransferase.	NF	NF	NF	NF
5174	2.1.1.107	Uroporphyrinogen-III C-methyltransferase.	NF	NF	FOUND	FOUND
5175	2.1.1.11	Magnesium protoporphyrin IX methyltransferase.	NF	NF	NF	NF
5176	2.1.1.114	Hexaprenyldihydroxybenzoate methyltransferase.	NF	NF	NF	NF
5177	2.1.1.116	3'-hydroxy-N-methyl-(S)-coclaurine 4'-O-methyltran	NF	NF	NF	NF
5178	2.1.1.119	10-hydroxydihydrosanguinarine 10-O-methyltransfe	NF	NF	NF	NF
5179	2.1.1.120	12-hydroxydihydrochelirubine 12-O-methyltransfer	NF	NF	NF	NF
5180	2.1.1.122	(S)-tetrahydroprotoberberine N-methyltransferase.	NF	NF	NF	NF
5181	2.1.1.128	(RS)-norcoclaurine 6-O-methyltransferase.	NF	NF	NF	NF
5182	2.1.1.13	Methionine synthase.	NF	NF	FOUND	FOUND
5183	2.1.1.130	Precorrin-2 C(20)-methyltransferase.	NF	NF	NF	NF
5184	2.1.1.131	Precorrin-3B C(17)-methyltransferase.	NF	NF	NF	NF
5185	2.1.1.132	Precorrin-6Y C(5,15)-methyltransferase (decarboxyl	NF	NF	NF	NF
5186	2.1.1.133	Precorrin-4 C(11)-methyltransferase.	NF	NF	NF	NF
5187	2.1.1.14	5-methyltetrahydropteroyltriglutamate--homocyste	FOUND	FOUND	FOUND	NF
5188	2.1.1.140	(S)-coclaurine-N-methyltransferase.	NF	NF	NF	NF
5189	2.1.1.151	Cobalt-factor II C(20)-methyltransferase.	NF	NF	NF	NF
5190	2.1.1.152	Precorrin-6A synthase (deacetylating).	NF	NF	NF	NF
5191	2.1.1.158	7-methylxanthosine synthase.	NF	NF	NF	NF
5192	2.1.1.159	Theobromine synthase.	NF	NF	NF	NF
5193	2.1.1.160	Caffeine synthase.	NF	NF	NF	NF
5194	2.1.1.17	Phosphatidylethanolamine N-methyltransferase.	NF	NF	NF	NF
5195	2.1.1.2	Guanidinoacetate N-methyltransferase.	NF	NF	NF	NF
5196	2.1.1.28	Phenylethanolamine N-methyltransferase.	NF	NF	NF	NF
5197	2.1.1.37	DNA (cytosine-5-)-methyltransferase.	NF	FOUND	FOUND	FOUND
5198	2.1.1.4	Acetylserotonin O-methyltransferase.	NF	NF	NF	NF
5199	2.1.1.41	Sterol 24-C-methyltransferase.	NF	NF	NF	NF
5200	2.1.1.45	Thymidylate synthase.	FOUND	NF	FOUND	FOUND
5201	2.1.1.5	Betaine--homocysteine S-methyltransferase.	NF	NF	NF	NF
5202	2.1.1.53	Putrescine N-methyltransferase.	NF	NF	NF	NF
5203	2.1.1.6	Catechol O-methyltransferase.	NF	NF	NF	NF
5204	2.1.1.64	3-demethylubiquinone-9 3-O-methyltransferase.	NF	NF	NF	NF
5205	2.1.1.68	Caffeate O-methyltransferase.	NF	NF	NF	NF
5206	2.1.1.71	Phosphatidyl-N-methylethanolamine N-methyltrans	NF	NF	NF	NF
5207	2.1.1.86	Tetrahydromethanopterin S-methyltransferase.	NF	NF	NF	NF
5208	2.1.1.95	Tocopherol O-methyltransferase.	NF	NF	NF	NF
5209	2.1.2.1	Glycine hydroxymethyltransferase.	NF	NF	FOUND	FOUND
5210	2.1.2.10	Aminomethyltransferase.	NF	NF	NF	NF
5211	2.1.2.11	3-methyl-2-oxobutanoate hydroxymethyltransferas	NF	NF	NF	NF
5212	2.1.2.2	Phosphoribosylglycinamide formyltransferase.	NF	FOUND	FOUND	FOUND
5213	2.1.2.3	Phosphoribosylaminoimidazolecarboxamide formyl	NF	NF	NF	NF
5214	2.1.2.4	Glycine formimidoyltransferase.	NF	NF	NF	NF
5215	2.1.2.5	Glutamate formimidoyltransferase.	NF	NF	NF	NF
5216	2.1.2.7	D-alanine 2-hydroxymethyltransferase.	NF	NF	NF	NF
5217	2.1.3.2	Aspartate carbamoyltransferase.	NF	NF	FOUND	FOUND
5218	2.1.3.3	Ornithine carbamoyltransferase.	FOUND	FOUND	FOUND	FOUND
5219	2.1.4.1	Glycine amidinotransferase.	NF	NF	NF	NF

5220	2.2.1.1	Transketolase.	FOUND	FOUND	FOUND	FOUND
5221	2.2.1.2	Transaldolase.	FOUND	NF	FOUND	FOUND
5222	2.2.1.6	Acetolactate synthase.	NF	NF	FOUND	FOUND
5223	2.2.1.7	1-deoxy-D-xylulose-5-phosphate synthase.	NF	NF	FOUND	FOUND
5224	2.2.1.9	2-succinyl-5-enolpyruvyl-6-hydroxy-3-cyclohexene-1	NF	NF	NF	NF
5225	2.3.-.-	Acyltransferases.	FOUND	FOUND	FOUND	FOUND
5226	2.3.1.-	Transferring groups other than amino-acyl groups.	FOUND	FOUND	FOUND	FOUND
5227	2.3.1.1	Amino-acid N-acetyltransferase.	NF	NF	FOUND	FOUND
5228	2.3.1.101	Formylmethanofuran--tetrahydromethanopterin N-	NF	NF	NF	NF
5229	2.3.1.105	Alkylglycerophosphate 2-O-acetyltransferase.	NF	NF	NF	NF
5230	2.3.1.117	2,3,4,5-tetrahydropyridine-2,6-dicarboxylate N-succ	NF	NF	NF	NF
5231	2.3.1.12	Dihydrolipoyllysine-residue acetyltransferase.	FOUND	FOUND	FOUND	FOUND
5232	2.3.1.121	1-alkenylglycerophosphoethanolamine O-acyltransf	NF	NF	NF	NF
5233	2.3.1.129	Acyl-[acyl-carrier-protein]--UDP-N-acetylglucosamin	NF	NF	FOUND	FOUND
5234	2.3.1.15	Glycerol-3-phosphate O-acyltransferase.	NF	NF	NF	NF
5235	2.3.1.150	Salutaridinol 7-O-acetyltransferase.	NF	NF	NF	NF
5236	2.3.1.157	Glucosamine-1-phosphate N-acetyltransferase.	NF	NF	NF	NF
5237	2.3.1.16	Acetyl-CoA C-acyltransferase.	NF	NF	NF	NF
5238	2.3.1.164	Isopenicillin-N N-acyltransferase.	NF	NF	NF	NF
5239	2.3.1.166	2-alpha-hydroxytaxane 2-O-benzoyltransferase.	NF	NF	NF	NF
5240	2.3.1.167	10-deacetylbaccatin III 10-O-acetyltransferase.	NF	NF	NF	NF
5241	2.3.1.168	Dihydrolipoyllysine-residue (2-methylpropanoyl)tra	NF	NF	NF	NF
5242	2.3.1.175	Deacetylcephalosporin-C acetyltransferase.	NF	NF	NF	NF
5243	2.3.1.176	Propanoyl-CoA C-acyltransferase.	NF	NF	NF	NF
5244	2.3.1.178	Diaminobutyrate acetyltransferase.	NF	NF	NF	NF
5245	2.3.1.179	Beta-ketoacyl-acyl-carrier-protein synthase II.	NF	NF	NF	NF
5246	2.3.1.180	Beta-ketoacyl-acyl-carrier-protein synthase III.	NF	NF	NF	NF
5247	2.3.1.181	Lipoyl(octanoyl) transferase.	NF	NF	NF	NF
5248	2.3.1.19	Phosphate butyryltransferase.	NF	NF	NF	NF
5249	2.3.1.20	Diacylglycerol O-acyltransferase.	NF	NF	NF	NF
5250	2.3.1.24	Sphingosine N-acyltransferase.	NF	NF	NF	NF
5251	2.3.1.30	Serine O-acetyltransferase.	NF	NF	FOUND	FOUND
5252	2.3.1.31	Homoserine O-acetyltransferase.	NF	NF	NF	NF
5253	2.3.1.35	Glutamate N-acetyltransferase.	NF	NF	NF	NF
5254	2.3.1.37	5-aminolevulinate synthase.	NF	NF	NF	NF
5255	2.3.1.39	[Acyl-carrier-protein] S-malonyltransferase.	NF	NF	FOUND	FOUND
5256	2.3.1.41	Beta-ketoacyl-acyl-carrier-protein synthase I.	NF	NF	FOUND	FOUND
5257	2.3.1.46	Homoserine O-succinyltransferase.	NF	NF	FOUND	FOUND
5258	2.3.1.47	8-amino-7-oxononanoate synthase.	NF	NF	NF	NF
5259	2.3.1.5	Arylamine N-acetyltransferase.	NF	NF	NF	NF
5260	2.3.1.50	Serine C-palmitoyltransferase.	NF	NF	NF	NF
5261	2.3.1.51	1-acylglycerol-3-phosphate O-acyltransferase.	NF	NF	FOUND	FOUND
5262	2.3.1.54	Formate C-acetyltransferase.	FOUND	FOUND	FOUND	FOUND
5263	2.3.1.57	Diamine N-acetyltransferase.	NF	NF	NF	NF
5264	2.3.1.61	Dihydrolipoyllysine-residue succinyltransferase.	FOUND	NF	FOUND	NF
5265	2.3.1.65	Bile acid-CoA:amino acid N-acyltransferase.	NF	NF	NF	NF
5266	2.3.1.67	1-alkylglycerophosphocholine O-acetyltransferase.	NF	NF	NF	NF
5267	2.3.1.68	Glutamine N-acyltransferase.	NF	NF	NF	NF
5268	2.3.1.74	Naringenin-chalcone synthase.	NF	NF	NF	NF
5269	2.3.1.78	Heparan-alpha-glucosaminide N-acetyltransferase.	NF	NF	NF	NF
5270	2.3.1.8	Phosphate acetyltransferase.	FOUND	FOUND	FOUND	FOUND
5271	2.3.1.85	Fatty-acid synthase.	NF	NF	NF	NF
5272	2.3.1.86	Fatty-acyl-CoA synthase.	NF	NF	NF	NF
5273	2.3.1.87	Aralkylamine N-acetyltransferase.	NF	NF	NF	NF
5274	2.3.1.9	Acetyl-CoA C-acetyltransferase.	FOUND	FOUND	NF	NF

5275	2.3.2.-	Aminoacyltransferases.	NF	NF	NF	NF
5276	2.3.2.2	Gamma-glutamyltransferase.	NF	NF	NF	NF
5277	2.3.3.1	Citrate (Si)-synthase.	NF	NF	FOUND	FOUND
5278	2.3.3.10	Hydroxymethylglutaryl-CoA synthase.	FOUND	FOUND	FOUND	NF
5279	2.3.3.13	2-isopropylmalate synthase.	NF	NF	FOUND	FOUND
5280	2.3.3.14	Homocitrate synthase.	NF	NF	NF	NF
5281	2.3.3.8	ATP citrate synthase.	NF	NF	NF	NF
5282	2.3.3.9	Malate synthase.	NF	NF	NF	NF
5283	2.4.-.-	Glycosyltransferases.	FOUND	FOUND	FOUND	FOUND
5284	2.4.1.-	Hexosyltransferases.	FOUND	FOUND	FOUND	FOUND
5285	2.4.1.10	Levansucrase.	NF	NF	NF	NF
5286	2.4.1.101	Alpha-1,3-mannosyl-glycoprotein 2-beta-N-acetylgl	NF	NF	NF	NF
5287	2.4.1.102	Beta-1,3-galactosyl-O-glycosyl-glycoprotein beta-1,	NF	NF	NF	NF
5288	2.4.1.115	Anthocyanidin 3-O-glucosyltransferase.	NF	NF	NF	NF
5289	2.4.1.117	Dolichyl-phosphate beta-glucosyltransferase.	NF	NF	NF	NF
5290	2.4.1.119	Dolichyl-diphosphooligosaccharide--protein glycotr	NF	NF	NF	NF
5291	2.4.1.122	Glycoprotein-N-acetylgalactosamine 3-beta-galacto	NF	NF	NF	NF
5292	2.4.1.13	Sucrose synthase.	NF	NF	NF	NF
5293	2.4.1.130	Dolichyl-phosphate-mannose--glycolipid alpha-man	NF	NF	NF	NF
5294	2.4.1.131	Glycolipid 2-alpha-mannosyltransferase.	NF	NF	NF	NF
5295	2.4.1.132	Glycolipid 3-alpha-mannosyltransferase.	NF	NF	NF	NF
5296	2.4.1.133	Xylosylprotein 4-beta-galactosyltransferase.	NF	NF	NF	NF
5297	2.4.1.134	Galactosylxylosylprotein 3-beta-galactosyltransfera	NF	NF	NF	NF
5298	2.4.1.135	Galactosylgalactosylxylosylprotein 3-beta-glucuron	NF	NF	NF	NF
5299	2.4.1.14	Sucrose-phosphate synthase.	NF	NF	NF	NF
5300	2.4.1.141	N-acetylglucosaminyldiphosphodolichol N-acetylglu	NF	NF	NF	NF
5301	2.4.1.142	Chitobiosyldiphosphodolichol beta-mannosyltransfe	NF	NF	NF	NF
5302	2.4.1.143	Alpha-1,6-mannosyl-glycoprotein 2-beta-N-acetylgl	NF	NF	NF	NF
5303	2.4.1.144	Beta-1,4-mannosyl-glycoprotein 4-beta-N-acetylglu	NF	NF	NF	NF
5304	2.4.1.145	Alpha-1,3-mannosyl-glycoprotein 4-beta-N-acetylgl	NF	NF	NF	NF
5305	2.4.1.147	Acetylgalactosaminyl-O-glycosyl-glycoprotein beta-	NF	NF	NF	NF
5306	2.4.1.148	Acetylgalactosaminyl-O-glycosyl-glycoprotein beta-	NF	NF	NF	NF
5307	2.4.1.149	N-acetyllactosaminide beta-1,3-N-acetylglucosamin	NF	NF	NF	NF
5308	2.4.1.150	N-acetyllactosaminide beta-1,6-N-acetylglucosamin	NF	NF	NF	NF
5309	2.4.1.152	4-galactosyl-N-acetylglucosaminide 3-alpha-L-fucos	NF	NF	NF	NF
5310	2.4.1.155	Alpha-1,6-mannosyl-glycoprotein 6-beta-N-acetylgl	NF	NF	NF	NF
5311	2.4.1.157	1,2-diacylglycerol 3-glucosyltransferase.	NF	NF	NF	NF
5312	2.4.1.159	Flavonol-3-O-glucoside L-rhamnosyltransferase.	NF	NF	NF	NF
5313	2.4.1.163	Beta-galactosyl-N-acetylglucosaminylgalactosylgluc	NF	NF	NF	NF
5314	2.4.1.17	Glucuronosyltransferase.	NF	NF	NF	NF
5315	2.4.1.174	Glucuronylgalactosylproteoglycan 4-beta-N- acetylg	NF	NF	NF	NF
5316	2.4.1.175	Glucuronosyl-N-acetylgalactosaminyl-proteoglycan	NF	NF	NF	NF
5317	2.4.1.18	1,4-alpha-glucan branching enzyme.	NF	NF	NF	NF
5318	2.4.1.182	Lipid-A-disaccharide synthase.	NF	NF	FOUND	FOUND
5319	2.4.1.184	Galactolipid galactosyltransferase.	NF	NF	NF	NF
5320	2.4.1.198	Phosphatidylinositol N-acetylglucosaminyltransfera	NF	NF	NF	NF
5321	2.4.1.201	Alpha-1,6-mannosyl-glycoprotein 4-beta-N-acetylgl	NF	NF	NF	NF
5322	2.4.1.206	Lactosylceramide 1,3-N-acetyl-beta-D-glucosaminyl	NF	NF	NF	NF
5323	2.4.1.21	Starch synthase.	FOUND	FOUND	FOUND	FOUND
5324	2.4.1.22	Lactose synthase.	NF	NF	NF	NF
5325	2.4.1.223	Glucuronyl-galactosyl-proteoglycan 4-alpha-N- acet	NF	NF	NF	NF
5326	2.4.1.224	Glucuronosyl-N-acetylglucosaminyl-proteoglycan 4-	NF	NF	NF	NF
5327	2.4.1.225	N-acetylglucosaminyl-proteoglycan 4-beta-glucuron	NF	NF	NF	NF
5328	2.4.1.226	N-acetylgalactosaminyl-proteoglycan 3-beta-glucur	NF	NF	NF	NF
5329	2.4.1.227	Undecaprenyldiphospho-muramoylpentapeptide be	NF	NF	NF	NF

5330	2.4.1.228	Lactosylceramide 4-alpha-galactosyltransferase.	NF	NF	NF	NF
5331	2.4.1.232	Initiation-specific alpha-1,6-mannosyltransferase.	NF	NF	NF	NF
5332	2.4.1.241	Digalactosyldiacylglycerol synthase.	NF	NF	NF	NF
5333	2.4.1.25	4-alpha-glucanotransferase.	FOUND	FOUND	FOUND	FOUND
5334	2.4.1.37	Fucosylgalactoside 3-alpha-galactosyltransferase.	NF	NF	NF	NF
5335	2.4.1.38	Beta-N-acetylglucosaminylglycopeptide beta-1,4-ga	NF	NF	NF	NF
5336	2.4.1.40	Glycoprotein-fucosylgalactoside alpha-N-acetylgala	NF	NF	NF	NF
5337	2.4.1.41	Polypeptide N-acetylgalactosaminyltransferase.	NF	NF	NF	NF
5338	2.4.1.44	Lipopolysaccharide 3-alpha-galactosyltransferase.	NF	NF	NF	NF
5339	2.4.1.46	Monogalactosyldiacylglycerol synthase.	NF	NF	NF	NF
5340	2.4.1.47	N-acylsphingosine galactosyltransferase.	NF	NF	NF	NF
5341	2.4.1.56	Lipopolysaccharide N-acetylglucosaminyltransferase	NF	NF	NF	NF
5342	2.4.1.58	Lipopolysaccharide glucosyltransferase I.	NF	NF	NF	NF
5343	2.4.1.62	Ganglioside galactosyltransferase.	NF	NF	NF	NF
5344	2.4.1.65	3-galactosyl-N-acetylglucosaminide 4-alpha-L-fucos	NF	NF	NF	NF
5345	2.4.1.68	Glycoprotein 6-alpha-L-fucosyltransferase.	NF	NF	NF	NF
5346	2.4.1.69	Galactoside 2-alpha-L-fucosyltransferase.	NF	NF	NF	NF
5347	2.4.1.79	Globotriaosylceramide 3-beta-N-acetylgalactosamir	NF	NF	NF	NF
5348	2.4.1.8	Maltose phosphorylase.	NF	NF	NF	NF
5349	2.4.1.80	Ceramide glucosyltransferase.	NF	NF	FOUND	FOUND
5350	2.4.1.83	Dolichyl-phosphate beta-D-mannosyltransferase.	NF	NF	NF	FOUND
5351	2.4.1.88	Globoside alpha-N-acetylgalactosaminyltransferase	NF	NF	NF	NF
5352	2.4.1.91	Flavonol 3-O-glucosyltransferase.	NF	NF	NF	NF
5353	2.4.1.92	(N-acetylneuraminyl)-galactosylglucosylceramide N-	NF	NF	NF	NF
5354	2.4.2.-	Pentosyltransferases.	FOUND	FOUND	FOUND	FOUND
5355	2.4.2.1	Purine-nucleoside phosphorylase.	FOUND	FOUND	FOUND	FOUND
5356	2.4.2.10	Orotate phosphoribosyltransferase.	NF	NF	FOUND	FOUND
5357	2.4.2.11	Nicotinate phosphoribosyltransferase.	NF	NF	NF	NF
5358	2.4.2.14	Amidophosphoribosyltransferase.	NF	FOUND	FOUND	FOUND
5359	2.4.2.15	Guanosine phosphorylase.	NF	NF	NF	NF
5360	2.4.2.17	ATP phosphoribosyltransferase.	NF	NF	FOUND	FOUND
5361	2.4.2.18	Anthranilate phosphoribosyltransferase.	NF	NF	FOUND	FOUND
5362	2.4.2.19	Nicotinate-nucleotide diphosphorylase (carboxylati	FOUND	FOUND	FOUND	FOUND
5363	2.4.2.2	Pyrimidine-nucleoside phosphorylase.	FOUND	FOUND	FOUND	FOUND
5364	2.4.2.21	Nicotinate-nucleotide--dimethylbenzimidazole phos	NF	NF	FOUND	FOUND
5365	2.4.2.22	Xanthine phosphoribosyltransferase.	NF	NF	NF	FOUND
5366	2.4.2.24	1,4-beta-D-xylan synthase.	NF	NF	NF	NF
5367	2.4.2.26	Protein xylosyltransferase.	NF	NF	NF	NF
5368	2.4.2.28	S-methyl-5'-thioadenosine phosphorylase.	NF	NF	NF	NF
5369	2.4.2.3	Uridine phosphorylase.	NF	NF	NF	FOUND
5370	2.4.2.4	Thymidine phosphorylase.	FOUND	FOUND	FOUND	FOUND
5371	2.4.2.7	Adenine phosphoribosyltransferase.	FOUND	FOUND	FOUND	FOUND
5372	2.4.2.8	Hypoxanthine phosphoribosyltransferase.	FOUND	FOUND	FOUND	FOUND
5373	2.4.2.9	Uracil phosphoribosyltransferase.	FOUND	FOUND	NF	FOUND
5374	2.4.99.-	Transferring other glycosyl groups.	NF	NF	NF	NF
5375	2.4.99.1	Beta-galactoside alpha-2,6-sialyltransferase.	NF	NF	NF	NF
5376	2.4.99.10	Neolactotetraosylceramide alpha-2,3-sialyltransfera	NF	NF	NF	NF
5377	2.4.99.3	Alpha-N-acetylgalactosaminide alpha-2,6-sialyltrans	NF	NF	NF	NF
5378	2.4.99.4	Beta-galactoside alpha-2,3-sialyltransferase.	NF	NF	NF	NF
5379	2.4.99.6	N-acetyllactosaminide alpha-2,3-sialyltransferase.	NF	NF	NF	NF
5380	2.4.99.7	Alpha-N-acetylneuraminyl-2,3-beta-galactosyl-1,3-N	NF	NF	NF	NF
5381	2.4.99.8	Alpha-N-acetylneuraminate alpha-2,8-sialyltransfer	NF	NF	NF	NF
5382	2.4.99.9	Lactosylceramide alpha-2,3-sialyltransferase.	NF	NF	NF	NF
5383	2.5.1.-	Transferring alkyl or aryl groups, other than methyl	FOUND	FOUND	FOUND	FOUND
5384	2.5.1.1	Dimethylallyltranstransferase.	NF	NF	FOUND	FOUND

5385	2.5.1.10	(2E,6E)-farnesyl diphosphate synthase.	FOUND	NF	NF	NF
5386	2.5.1.15	Dihydropteroate synthase.	NF	NF	FOUND	FOUND
5387	2.5.1.16	Spermidine synthase.	NF	NF	FOUND	NF
5388	2.5.1.17	Cob(I)yrinic acid a,c-diamide adenosyltransferase.	NF	NF	FOUND	FOUND
5389	2.5.1.19	3-phosphoshikimate 1-carboxyvinyltransferase.	NF	NF	FOUND	FOUND
5390	2.5.1.21	Squalene synthase.	NF	NF	NF	NF
5391	2.5.1.22	Spermine synthase.	NF	NF	NF	NF
5392	2.5.1.26	Alkylglycerone-phosphate synthase.	NF	NF	NF	NF
5393	2.5.1.29	Farnesyltranstransferase.	NF	NF	NF	NF
5394	2.5.1.3	Thiamine-phosphate diphosphorylase.	NF	NF	NF	FOUND
5395	2.5.1.32	Phytoene synthase.	NF	NF	NF	NF
5396	2.5.1.39	4-hydroxybenzoate polyprenyltransferase.	NF	NF	NF	NF
5397	2.5.1.47	Cysteine synthase.	NF	NF	FOUND	FOUND
5398	2.5.1.48	Cystathionine gamma-synthase.	NF	NF	FOUND	NF
5399	2.5.1.49	O-acetylhomoserine aminocarboxypropyltransferas	NF	NF	NF	FOUND
5400	2.5.1.54	3-deoxy-7-phosphoheptulonate synthase.	NF	NF	FOUND	FOUND
5401	2.5.1.55	3-deoxy-8-phosphooctulonate synthase.	NF	NF	FOUND	FOUND
5402	2.5.1.56	N-acetylneuraminate synthase.	NF	NF	NF	NF
5403	2.5.1.57	N-acylneuraminate-9-phosphate synthase.	NF	NF	NF	NF
5404	2.5.1.6	Methionine adenosyltransferase.	FOUND	FOUND	FOUND	FOUND
5405	2.5.1.61	Hydroxymethylbilane synthase.	NF	NF	FOUND	FOUND
5406	2.5.1.62	Chlorophyll synthase.	NF	NF	NF	NF
5407	2.5.1.65	O-phosphoserine sulfhydrylase.	NF	NF	NF	NF
5408	2.5.1.7	UDP-N-acetylglucosamine 1-carboxyvinyltransferase	FOUND	FOUND	FOUND	FOUND
5409	2.5.1.74	1,4-dihydroxy-2-naphthoate polyprenyltransferase.	NF	NF	NF	NF
5410	2.5.1.75	tRNA dimethylallyltransferase.	FOUND	NF	NF	NF
5411	2.5.1.78	6,7-dimethyl-8-ribityllumazine synthase.	NF	NF	NF	NF
5412	2.5.1.9	Riboflavin synthase.	NF	NF	FOUND	FOUND
5413	2.6.1.-	Transaminases (aminotransferases).	FOUND	FOUND	FOUND	FOUND
5414	2.6.1.1	Aspartate transaminase.	FOUND	FOUND	FOUND	FOUND
5415	2.6.1.11	Acetylornithine transaminase.	NF	NF	FOUND	FOUND
5416	2.6.1.12	Alanine--oxo-acid transaminase.	NF	NF	NF	NF
5417	2.6.1.13	Ornithine aminotransferase.	NF	NF	NF	NF
5418	2.6.1.16	Glutamine--fructose-6-phosphate transaminase (iso	FOUND	NF	FOUND	FOUND
5419	2.6.1.17	Succinyldiaminopimelate transaminase.	NF	NF	NF	NF
5420	2.6.1.18	Beta-alanine--pyruvate transaminase.	NF	NF	NF	NF
5421	2.6.1.19	4-aminobutyrate transaminase.	NF	NF	NF	FOUND
5422	2.6.1.2	Alanine transaminase.	NF	NF	NF	NF
5423	2.6.1.21	D-amino-acid transaminase.	NF	NF	NF	NF
5424	2.6.1.27	Tryptophan transaminase.	NF	NF	NF	NF
5425	2.6.1.3	Cysteine transaminase.	NF	NF	NF	NF
5426	2.6.1.36	L-lysine 6-transaminase.	NF	NF	NF	NF
5427	2.6.1.37	2-aminoethylphosphonate--pyruvate transaminase.	NF	NF	NF	NF
5428	2.6.1.39	2-aminoadipate transaminase.	NF	NF	NF	NF
5429	2.6.1.4	Glycine transaminase.	NF	NF	NF	NF
5430	2.6.1.42	Branched-chain-amino-acid transaminase.	NF	NF	FOUND	FOUND
5431	2.6.1.44	Alanine--glyoxylate transaminase.	NF	NF	NF	NF
5432	2.6.1.45	Serine--glyoxylate transaminase.	NF	NF	NF	NF
5433	2.6.1.5	Tyrosine transaminase.	NF	NF	NF	NF
5434	2.6.1.51	Serine--pyruvate transaminase.	NF	NF	FOUND	FOUND
5435	2.6.1.52	Phosphoserine transaminase.	NF	NF	NF	NF
5436	2.6.1.55	Taurine--2-oxoglutarate transaminase.	NF	NF	NF	NF
5437	2.6.1.57	Aromatic-amino-acid transaminase.	NF	NF	NF	NF
5438	2.6.1.58	Phenylalanine(histidine) transaminase.	NF	NF	NF	NF
5439	2.6.1.6	Leucine transaminase.	NF	NF	NF	NF

5440	2.6.1.62	Adenosylmethionine--8-amino-7-oxononanoate tra	NF	NF	NF	NF
5441	2.6.1.66	Valine--pyruvate transaminase.	NF	NF	NF	NF
5442	2.6.1.7	Kynurenine--oxoglutarate transaminase.	NF	NF	NF	NF
5443	2.6.1.76	Diaminobutyrate--2-oxoglutarate transaminase.	NF	NF	NF	NF
5444	2.6.1.77	Taurine--pyruvate aminotransferase.	NF	NF	NF	NF
5445	2.6.1.84	Arginine--pyruvate transaminase.	NF	NF	NF	NF
5446	2.6.1.9	Histidinol-phosphate transaminase.	NF	NF	FOUND	FOUND
5447	2.6.99.2	Pyridoxine 5'-phosphate synthase.	NF	NF	NF	NF
5448	2.7.-.-	Transferring phosphorous-containing groups.	FOUND	FOUND	FOUND	FOUND
5449	2.7.1.-	Phosphotransferases with an alcohol group as acce	FOUND	FOUND	FOUND	FOUND
5450	2.7.1.1	Hexokinase.	NF	NF	NF	NF
5451	2.7.1.100	S-methyl-5-thioribose kinase.	NF	NF	NF	NF
5452	2.7.1.107	Diacylglycerol kinase.	FOUND	FOUND	FOUND	FOUND
5453	2.7.1.108	Dolichol kinase.	NF	NF	NF	NF
5454	2.7.1.11	6-phosphofructokinase.	FOUND	FOUND	FOUND	FOUND
5455	2.7.1.113	Deoxyguanosine kinase.	NF	NF	NF	NF
5456	2.7.1.12	Gluconokinase.	NF	NF	NF	NF
5457	2.7.1.127	Inositol-trisphosphate 3-kinase.	NF	NF	NF	NF
5458	2.7.1.13	Dehydrogluconokinase.	NF	NF	NF	NF
5459	2.7.1.130	Tetraacyldisaccharide 4'-kinase.	NF	NF	FOUND	FOUND
5460	2.7.1.137	Phosphatidylinositol 3-kinase.	NF	NF	NF	NF
5461	2.7.1.140	Inositol-tetrakisphosphate 5-kinase.	NF	NF	NF	NF
5462	2.7.1.146	ADP-specific phosphofructokinase.	NF	NF	NF	NF
5463	2.7.1.147	ADP-specific glucokinase.	NF	NF	NF	NF
5464	2.7.1.148	4-(cytidine 5'-diphospho)-2-C-methyl-D-erythritol ki	NF	NF	NF	FOUND
5465	2.7.1.154	Phosphatidylinositol-4-phosphate 3-kinase.	NF	NF	NF	NF
5466	2.7.1.156	Adenosylcobinamide kinase.	NF	NF	FOUND	FOUND
5467	2.7.1.158	Inositol-pentakisphosphate 2-kinase.	NF	NF	NF	NF
5468	2.7.1.159	Inositol-1,3,4-trisphosphate 5/6-kinase.	NF	NF	NF	NF
5469	2.7.1.16	Ribulokinase.	NF	NF	NF	NF
5470	2.7.1.161	CTP-dependent riboflavin kinase.	NF	NF	NF	NF
5471	2.7.1.17	Xylulokinase.	FOUND	NF	NF	FOUND
5472	2.7.1.19	Phosphoribulokinase.	NF	NF	NF	NF
5473	2.7.1.2	Glucokinase.	FOUND	FOUND	FOUND	FOUND
5474	2.7.1.20	Adenosine kinase.	NF	NF	NF	NF
5475	2.7.1.21	Thymidine kinase.	FOUND	FOUND	FOUND	FOUND
5476	2.7.1.23	NAD(+) kinase.	FOUND	FOUND	FOUND	FOUND
5477	2.7.1.24	Dephospho-CoA kinase.	FOUND	FOUND	FOUND	FOUND
5478	2.7.1.25	Adenylyl-sulfate kinase.	NF	NF	NF	NF
5479	2.7.1.26	Riboflavin kinase.	NF	NF	NF	NF
5480	2.7.1.29	Glycerone kinase.	FOUND	FOUND	FOUND	FOUND
5481	2.7.1.3	Ketohexokinase.	NF	NF	NF	NF
5482	2.7.1.30	Glycerol kinase.	NF	NF	FOUND	FOUND
5483	2.7.1.31	Glycerate kinase.	FOUND	NF	NF	FOUND
5484	2.7.1.32	Choline kinase.	NF	NF	NF	NF
5485	2.7.1.33	Pantothenate kinase.	NF	FOUND	FOUND	FOUND
5486	2.7.1.35	Pyridoxal kinase.	NF	NF	FOUND	NF
5487	2.7.1.36	Mevalonate kinase.	FOUND	FOUND	FOUND	NF
5488	2.7.1.39	Homoserine kinase.	NF	NF	FOUND	FOUND
5489	2.7.1.4	Fructokinase.	NF	NF	FOUND	FOUND
5490	2.7.1.40	Pyruvate kinase.	FOUND	FOUND	FOUND	FOUND
5491	2.7.1.43	Glucuronokinase.	NF	NF	NF	NF
5492	2.7.1.45	2-dehydro-3-deoxygluconokinase.	FOUND	FOUND	NF	FOUND
5493	2.7.1.46	L-arabinokinase.	NF	NF	NF	NF
5494	2.7.1.47	D-ribulokinase.	NF	NF	NF	NF

5495	2.7.1.48	Uridine kinase.	FOUND	FOUND	FOUND	FOUND
5496	2.7.1.49	Hydroxymethylpyrimidine kinase.	NF	NF	NF	NF
5497	2.7.1.50	Hydroxyethylthiazole kinase.	NF	NF	NF	FOUND
5498	2.7.1.52	Fucokinase.	NF	NF	NF	NF
5499	2.7.1.6	Galactokinase.	NF	FOUND	FOUND	FOUND
5500	2.7.1.60	N-acylmannosamine kinase.	NF	NF	NF	NF
5501	2.7.1.63	Polyphosphate--glucose phosphotransferase.	NF	NF	NF	NF
5502	2.7.1.67	1-phosphatidylinositol 4-kinase.	NF	NF	NF	NF
5503	2.7.1.68	1-phosphatidylinositol-4-phosphate 5-kinase.	NF	NF	NF	NF
5504	2.7.1.69	Protein-N(pi)-phosphohistidine--sugar phosphotran	FOUND	FOUND	FOUND	FOUND
5505	2.7.1.7	Mannokinase.	NF	NF	NF	NF
5506	2.7.1.71	Shikimate kinase.	NF	FOUND	FOUND	FOUND
5507	2.7.1.74	Deoxycytidine kinase.	NF	NF	NF	NF
5508	2.7.1.76	Deoxyadenosine kinase.	FOUND	FOUND	FOUND	FOUND
5509	2.7.1.82	Ethanolamine kinase.	NF	NF	NF	NF
5510	2.7.1.89	Thiamine kinase.	NF	NF	NF	NF
5511	2.7.1.91	Sphinganine kinase.	NF	NF	NF	NF
5512	2.7.1.92	5-dehydro-2-deoxygluconokinase.	NF	NF	NF	FOUND
5513	2.7.1.94	Acylglycerol kinase.	NF	NF	NF	NF
5514	2.7.2.-	Phosphotransferases with a carboxyl group as accep	FOUND	FOUND	FOUND	FOUND
5515	2.7.2.1	Acetate kinase.	FOUND	FOUND	FOUND	FOUND
5516	2.7.2.11	Glutamate 5-kinase.	NF	NF	FOUND	FOUND
5517	2.7.2.12	Acetate kinase (diphosphate).	NF	NF	NF	NF
5518	2.7.2.3	Phosphoglycerate kinase.	FOUND	FOUND	FOUND	FOUND
5519	2.7.2.4	Aspartate kinase.	FOUND	FOUND	FOUND	FOUND
5520	2.7.2.7	Butyrate kinase.	NF	NF	NF	NF
5521	2.7.2.8	Acetylglutamate kinase.	NF	NF	FOUND	FOUND
5522	2.7.3.2	Creatine kinase.	NF	NF	NF	NF
5523	2.7.4.11	(Deoxy)adenylate kinase.	NF	NF	NF	NF
5524	2.7.4.14	Cytidylate kinase.	FOUND	FOUND	FOUND	FOUND
5525	2.7.4.15	Thiamine-diphosphate kinase.	NF	NF	NF	NF
5526	2.7.4.16	Thiamine-phosphate kinase.	NF	NF	NF	NF
5527	2.7.4.2	Phosphomevalonate kinase.	FOUND	FOUND	FOUND	NF
5528	2.7.4.22	UMP kinase.	NF	NF	NF	NF
5529	2.7.4.3	Adenylate kinase.	FOUND	FOUND	FOUND	FOUND
5530	2.7.4.6	Nucleoside-diphosphate kinase.	NF	NF	NF	FOUND
5531	2.7.4.7	Phosphomethylpyrimidine kinase.	NF	NF	FOUND	FOUND
5532	2.7.4.8	Guanylate kinase.	FOUND	FOUND	FOUND	FOUND
5533	2.7.4.9	dTMP kinase.	FOUND	FOUND	FOUND	FOUND
5534	2.7.6.1	Ribose-phosphate diphosphokinase.	FOUND	FOUND	FOUND	FOUND
5535	2.7.6.2	Thiamine diphosphokinase.	FOUND	FOUND	FOUND	FOUND
5536	2.7.6.3	2-amino-4-hydroxy-6-hydroxymethyldihydropteridi	NF	NF	FOUND	FOUND
5537	2.7.7.-	Nucleotidyltransferases.	FOUND	FOUND	FOUND	FOUND
5538	2.7.7.1	Nicotinamide-nucleotide adenylyltransferase.	FOUND	FOUND	FOUND	NF
5539	2.7.7.10	UTP--hexose-1-phosphate uridylyltransferase.	NF	NF	NF	NF
5540	2.7.7.12	UDP-glucose--hexose-1-phosphate uridylyltransfera	FOUND	FOUND	FOUND	FOUND
5541	2.7.7.13	Mannose-1-phosphate guanylyltransferase.	FOUND	NF	FOUND	FOUND
5542	2.7.7.14	Ethanolamine-phosphate cytidylyltransferase.	NF	NF	NF	NF
5543	2.7.7.15	Choline-phosphate cytidylyltransferase.	NF	NF	NF	NF
5544	2.7.7.18	Nicotinate-nucleotide adenylyltransferase.	NF	NF	NF	FOUND
5545	2.7.7.2	FAD synthetase.	FOUND	FOUND	FOUND	FOUND
5546	2.7.7.22	Mannose-1-phosphate guanylyltransferase (GDP).	FOUND	NF	FOUND	FOUND
5547	2.7.7.23	UDP-N-acetylglucosamine diphosphorylase.	FOUND	FOUND	FOUND	FOUND
5548	2.7.7.24	Glucose-1-phosphate thymidylyltransferase.	NF	NF	FOUND	FOUND
5549	2.7.7.27	Glucose-1-phosphate adenylyltransferase.	FOUND	FOUND	FOUND	FOUND

5550	2.7.7.3	Pantetheine-phosphate adenylyltransferase.	FOUND	FOUND	FOUND	FOUND
5551	2.7.7.30	Fucose-1-phosphate guanylyltransferase.	NF	NF	NF	NF
5552	2.7.7.33	Glucose-1-phosphate cytidylyltransferase.	NF	NF	NF	NF
5553	2.7.7.34	Glucose-1-phosphate guanylyltransferase.	NF	NF	NF	NF
5554	2.7.7.37	Aldose-1-phosphate nucleotidyltransferase.	NF	NF	NF	NF
5555	2.7.7.38	3-deoxy-manno-octulosonate cytidylyltransferase.	NF	NF	FOUND	FOUND
5556	2.7.7.4	Sulfate adenylyltransferase.	NF	NF	FOUND	NF
5557	2.7.7.41	Phosphatidate cytidylyltransferase.	FOUND	FOUND	FOUND	FOUND
5558	2.7.7.43	N-acylneuraminate cytidylyltransferase.	NF	NF	NF	NF
5559	2.7.7.44	Glucuronate-1-phosphate uridylyltransferase.	NF	NF	NF	NF
5560	2.7.7.48	RNA-directed RNA polymerase.	NF	NF	NF	NF
5561	2.7.7.5	Sulfate adenylyltransferase (ADP).	NF	NF	NF	NF
5562	2.7.7.6	DNA-directed RNA polymerase.	FOUND	FOUND	FOUND	FOUND
5563	2.7.7.60	2-C-methyl-D-erythritol 4-phosphate cytidylyltransf	NF	NF	NF	NF
5564	2.7.7.62	Adenosylcobinamide-phosphate guanylyltransferas	NF	NF	FOUND	FOUND
5565	2.7.7.63	Lipoate--protein ligase.	NF	NF	NF	NF
5566	2.7.7.69	GDP-L-galactose phosphorylase.	NF	NF	NF	NF
5567	2.7.7.7	DNA-directed DNA polymerase.	FOUND	FOUND	FOUND	FOUND
5568	2.7.7.9	UTP--glucose-1-phosphate uridylyltransferase.	FOUND	FOUND	FOUND	FOUND
5569	2.7.8.-	Transferases for other substituted phosphate group	FOUND	FOUND	FOUND	FOUND
5570	2.7.8.1	Ethanolaminephosphotransferase.	NF	NF	NF	NF
5571	2.7.8.11	CDP-diacylglycerol--inositol 3-phosphatidyltransfera	NF	NF	NF	NF
5572	2.7.8.13	Phospho-N-acetylmuramoyl-pentapeptide-transfera	FOUND	FOUND	FOUND	FOUND
5573	2.7.8.15	UDP-N-acetylglucosamine--dolichyl-phosphate N-ac	NF	NF	NF	NF
5574	2.7.8.2	Diacylglycerol cholinephosphotransferase.	NF	NF	NF	NF
5575	2.7.8.20	Phosphatidylglycerol--membrane-oligosaccharide gl	NF	NF	NF	NF
5576	2.7.8.22	1-alkenyl-2-acylglycerol choline phosphotransferase	NF	NF	NF	NF
5577	2.7.8.26	Adenosylcobinamide-GDP ribazoletransferase.	NF	NF	NF	NF
5578	2.7.8.27	Sphingomyelin synthase.	NF	NF	NF	NF
5579	2.7.8.3	Ceramide cholinephosphotransferase.	NF	NF	NF	NF
5580	2.7.8.5	CDP-diacylglycerol--glycerol-3-phosphate 3-phospha	FOUND	FOUND	FOUND	FOUND
5581	2.7.8.8	CDP-diacylglycerol--serine O-phosphatidyltransferas	NF	NF	NF	NF
5582	2.7.9.1	Pyruvate, phosphate dikinase.	NF	NF	NF	NF
5583	2.7.9.3	Selenide, water dikinase.	NF	NF	NF	FOUND
5584	2.8.1.2	3-mercaptopyruvate sulfurtransferase.	NF	NF	NF	NF
5585	2.8.1.6	Biotin synthase.	NF	NF	FOUND	NF
5586	2.8.1.8	Lipoyl synthase.	NF	NF	NF	NF
5587	2.8.2.11	Galactosylceramide sulfotransferase.	NF	NF	NF	NF
5588	2.8.3.1	Propionate CoA-transferase.	NF	NF	NF	NF
5589	2.8.3.11	Citramalate CoA-transferase.	NF	NF	NF	NF
5590	2.8.3.15	Succinyl-CoA:(R)-benzylsuccinate CoA-transferase.	NF	NF	NF	NF
5591	2.8.3.2	Oxalate CoA-transferase.	NF	NF	NF	NF
5592	2.8.3.6	3-oxoadipate CoA-transferase.	NF	NF	NF	NF
5593	2.8.3.8	Acetate CoA-transferase.	NF	NF	NF	NF
5594	2.8.4.1	Coenzyme-B sulfoethylthiotransferase.	NF	NF	NF	NF
5595	3.-.-.-	Hydrolases.	FOUND	FOUND	FOUND	FOUND
5596	3.1.1.1	Carboxylesterase.	NF	NF	NF	FOUND
5597	3.1.1.11	Pectinesterase.	NF	NF	NF	NF
5598	3.1.1.14	Chlorophyllase.	NF	NF	NF	FOUND
5599	3.1.1.15	L-arabinonolactonase.	NF	NF	NF	NF
5600	3.1.1.17	Gluconolactonase.	NF	NF	NF	NF
5601	3.1.1.19	Uronolactonase.	NF	NF	NF	NF
5602	3.1.1.2	Arylesterase.	NF	NF	NF	NF
5603	3.1.1.23	Acylglycerol lipase.	NF	NF	NF	NF
5604	3.1.1.24	3-oxoadipate enol-lactonase.	NF	NF	NF	NF

5605	3.1.1.25	1,4-lactonase.	NF	NF	NF	NF
5606	3.1.1.3	Triacylglycerol lipase.	NF	NF	FOUND	NF
5607	3.1.1.31	6-phosphogluconolactonase.	NF	NF	NF	NF
5608	3.1.1.32	Phospholipase A(1).	NF	NF	NF	NF
5609	3.1.1.4	Phospholipase A(2).	NF	NF	NF	NF
5610	3.1.1.45	Carboxymethylenebutenolidase.	NF	NF	NF	NF
5611	3.1.1.47	1-alkyl-2-acetylglycerophosphocholine esterase.	NF	NF	NF	NF
5612	3.1.2.-	Thiolester hydrolases.	NF	FOUND	NF	NF
5613	3.1.2.10	Formyl-CoA hydrolase.	NF	NF	NF	NF
5614	3.1.2.14	Oleoyl-[acyl-carrier-protein] hydrolase.	NF	NF	NF	NF
5615	3.1.2.22	Palmitoyl-protein hydrolase.	NF	NF	NF	NF
5616	3.1.2.23	4-hydroxybenzoyl-CoA thioesterase.	NF	NF	NF	NF
5617	3.1.2.4	3-hydroxyisobutyryl-CoA hydrolase.	NF	NF	NF	NF
5618	3.1.3.-	Phosphoric monoester hydrolases.	FOUND	FOUND	FOUND	FOUND
5619	3.1.3.1	Alkaline phosphatase.	FOUND	FOUND	FOUND	FOUND
5620	3.1.3.11	Fructose-bisphosphatase.	NF	NF	NF	FOUND
5621	3.1.3.15	Histidinol-phosphatase.	NF	NF	FOUND	FOUND
5622	3.1.3.18	Phosphoglycolate phosphatase.	FOUND	FOUND	FOUND	FOUND
5623	3.1.3.21	Glycerol-1-phosphatase.	NF	NF	NF	NF
5624	3.1.3.24	Sucrose-phosphate phosphatase.	NF	NF	NF	NF
5625	3.1.3.25	Inositol-phosphate phosphatase.	NF	NF	NF	NF
5626	3.1.3.27	Phosphatidylglycerophosphatase.	NF	NF	NF	NF
5627	3.1.3.29	N-acylneuraminate-9-phosphatase.	NF	NF	NF	NF
5628	3.1.3.3	Phosphoserine phosphatase.	NF	NF	NF	NF
5629	3.1.3.36	Phosphoinositide 5-phosphatase.	NF	NF	NF	NF
5630	3.1.3.37	Sedoheptulose-bisphosphatase.	NF	NF	NF	NF
5631	3.1.3.4	Phosphatidate phosphatase.	NF	NF	NF	NF
5632	3.1.3.45	3-deoxy-manno-octulosonate-8-phosphatase.	NF	NF	FOUND	NF
5633	3.1.3.5	5'-nucleotidase.	FOUND	FOUND	FOUND	FOUND
5634	3.1.3.51	Dolichyl-phosphatase.	NF	NF	NF	NF
5635	3.1.3.56	Inositol-polyphosphate 5-phosphatase.	NF	NF	NF	NF
5636	3.1.3.57	Inositol-1,4-bisphosphate 1-phosphatase.	NF	NF	NF	NF
5637	3.1.3.59	Alkylacetylglycerophosphatase.	NF	NF	NF	NF
5638	3.1.3.64	Phosphatidylinositol-3-phosphatase.	NF	NF	NF	NF
5639	3.1.3.66	Phosphatidylinositol-3,4-bisphosphate 4-phosphata	NF	NF	NF	NF
5640	3.1.3.7	3'(2'),5'-bisphosphate nucleotidase.	NF	NF	NF	NF
5641	3.1.3.73	Alpha-ribazole phosphatase.	NF	NF	NF	FOUND
5642	3.1.3.74	Pyridoxal phosphatase.	NF	NF	NF	NF
5643	3.1.3.75	Phosphoethanolamine/phosphocholine phosphatas	NF	NF	NF	NF
5644	3.1.3.77	Acireductone synthase.	NF	NF	NF	NF
5645	3.1.3.9	Glucose-6-phosphatase.	NF	NF	NF	NF
5646	3.1.4.11	Phosphoinositide phospholipase C.	NF	NF	NF	NF
5647	3.1.4.12	Sphingomyelin phosphodiesterase.	NF	NF	NF	NF
5648	3.1.4.3	Phospholipase C.	NF	NF	NF	NF
5649	3.1.4.4	Phospholipase D.	NF	NF	NF	NF
5650	3.1.4.48	Dolichylphosphate-glucose phosphodiesterase.	NF	NF	NF	NF
5651	3.1.6.12	N-acetylgalactosamine-4-sulfatase.	NF	NF	NF	NF
5652	3.1.6.13	Iduronate-2-sulfatase.	NF	NF	NF	NF
5653	3.1.6.14	N-acetylglucosamine-6-sulfatase.	NF	FOUND	NF	NF
5654	3.1.6.18	Glucuronate-2-sulfatase.	NF	NF	NF	NF
5655	3.1.6.4	N-acetylgalactosamine-6-sulfatase.	NF	NF	NF	NF
5656	3.1.7.3	Monoterpenyl-diphosphatase.	NF	NF	NF	NF
5657	3.10.1.1	N-sulfoglucosamine sulfohydrolase.	NF	NF	NF	NF
5658	3.11.1.1	Phosphonoacetaldehyde hydrolase.	NF	NF	NF	FOUND
5659	3.2.-.-	Glycosylases.	FOUND	FOUND	FOUND	FOUND

5660	3.2.1.-	Glycosidases, i.e. enzymes hydrolyzing O- and S-glyc	FOUND	FOUND	FOUND	FOUND
5661	3.2.1.1	Alpha-amylase.	NF	FOUND	NF	NF
5662	3.2.1.10	Oligo-1,6-glucosidase.	NF	NF	FOUND	NF
5663	3.2.1.103	Keratan-sulfate endo-1,4-beta-galactosidase.	NF	NF	NF	NF
5664	3.2.1.105	3-alpha-(S)-strictosidine beta-glucosidase.	NF	NF	NF	NF
5665	3.2.1.106	Mannosyl-oligosaccharide glucosidase.	NF	NF	NF	FOUND
5666	3.2.1.108	Lactase.	NF	NF	NF	NF
5667	3.2.1.113	Mannosyl-oligosaccharide 1,2-alpha-mannosidase.	NF	NF	NF	NF
5668	3.2.1.114	Mannosyl-oligosaccharide 1,3-1,6-alpha-mannosida	NF	NF	NF	NF
5669	3.2.1.15	Polygalacturonase.	NF	NF	NF	FOUND
5670	3.2.1.2	Beta-amylase.	NF	NF	NF	NF
5671	3.2.1.20	Alpha-glucosidase.	NF	NF	NF	NF
5672	3.2.1.23	Beta-galactosidase.	NF	NF	NF	NF
5673	3.2.1.26	Beta-fructofuranosidase.	NF	NF	NF	NF
5674	3.2.1.3	Glucan 1,4-alpha-glucosidase.	NF	NF	NF	NF
5675	3.2.1.31	Beta-glucuronidase.	NF	NF	NF	NF
5676	3.2.1.35	Hyaluronoglucosaminidase.	NF	NF	NF	NF
5677	3.2.1.37	Xylan 1,4-beta-xylosidase.	NF	NF	NF	NF
5678	3.2.1.45	Glucosylceramidase.	NF	NF	NF	NF
5679	3.2.1.46	Galactosylceramidase.	NF	NF	NF	NF
5680	3.2.1.48	Sucrose alpha-glucosidase.	NF	NF	NF	NF
5681	3.2.1.50	Alpha-N-acetylglucosaminidase.	NF	NF	NF	NF
5682	3.2.1.52	Beta-N-acetylhexosaminidase.	NF	NF	NF	NF
5683	3.2.1.67	Galacturan 1,4-alpha-galacturonidase.	NF	NF	NF	NF
5684	3.2.1.76	L-iduronidase.	NF	NF	NF	NF
5685	3.2.1.84	Glucan 1,3-alpha-glucosidase.	NF	NF	NF	NF
5686	3.2.2.1	Purine nucleosidase.	NF	FOUND	FOUND	FOUND
5687	3.2.2.12	Inosinate nucleosidase.	NF	NF	NF	NF
5688	3.2.2.16	Methylthioadenosine nucleosidase.	NF	NF	NF	NF
5689	3.2.2.25	N-methyl nucleosidase.	NF	NF	NF	NF
5690	3.2.2.5	NAD(+) nucleosidase.	NF	NF	NF	NF
5691	3.2.2.8	Ribosylpyrimidine nucleosidase.	NF	NF	NF	NF
5692	3.2.2.9	Adenosylhomocysteine nucleosidase.	NF	FOUND	FOUND	FOUND
5693	3.3.1.1	Adenosylhomocysteinase.	NF	NF	NF	NF
5694	3.3.2.10	Soluble epoxide hydrolase.	NF	NF	NF	NF
5695	3.3.2.6	Leukotriene-A(4) hydrolase.	NF	NF	NF	NF
5696	3.4.-.-	Acting on peptide bonds (peptide hydrolases).	FOUND	FOUND	FOUND	FOUND
5697	3.4.11.1	Leucyl aminopeptidase.	FOUND	FOUND	NF	NF
5698	3.4.11.2	Membrane alanyl aminopeptidase.	NF	NF	NF	NF
5699	3.4.11.23	PepB aminopeptidase.	NF	NF	NF	NF
5700	3.4.13.20	Beta-Ala-His dipeptidase.	NF	NF	NF	NF
5701	3.4.13.3	Xaa-His dipeptidase.	NF	NF	NF	NF
5702	3.4.16.4	Serine-type D-Ala-D-Ala carboxypeptidase.	FOUND	FOUND	FOUND	FOUND
5703	3.5.1.-	In linear amides.	FOUND	FOUND	FOUND	FOUND
5704	3.5.1.1	Asparaginase.	NF	NF	NF	NF
5705	3.5.1.102	2-amino-5-formylamino-6-ribosylaminopyrimidin-4(	NF	NF	NF	NF
5706	3.5.1.12	Biotinidase.	NF	NF	NF	NF
5707	3.5.1.14	Aminoacylase.	FOUND	FOUND	FOUND	FOUND
5708	3.5.1.16	Acetylornithine deacetylase.	NF	NF	NF	NF
5709	3.5.1.18	Succinyl-diaminopimelate desuccinylase.	NF	NF	NF	FOUND
5710	3.5.1.19	Nicotinamidase.	NF	NF	NF	FOUND
5711	3.5.1.2	Glutaminase.	NF	NF	NF	NF
5712	3.5.1.22	Pantothenase.	NF	NF	NF	NF
5713	3.5.1.23	Ceramidase.	NF	NF	NF	NF
5714	3.5.1.24	Choloylglycine hydrolase.	NF	NF	NF	NF

5715	3.5.1.35	D-glutaminase.	NF	NF	NF	NF
5716	3.5.1.38	Glutamin-(asparagin-)ase.	NF	NF	NF	NF
5717	3.5.1.5	Urease.	NF	NF	NF	NF
5718	3.5.1.53	N-carbamoylputrescine amidase.	NF	NF	NF	NF
5719	3.5.1.54	Allophanate hydrolase.	NF	NF	NF	NF
5720	3.5.1.59	N-carbamoylsarcosine amidase.	NF	NF	NF	NF
5721	3.5.1.6	Beta-ureidopropionase.	NF	NF	NF	NF
5722	3.5.1.62	Acetylputrescine deacetylase.	NF	NF	NF	NF
5723	3.5.1.63	4-acetamidobutyrate deacetylase.	NF	NF	NF	NF
5724	3.5.1.78	Glutathionylspermidine amidase.	NF	NF	NF	NF
5725	3.5.1.84	Biuret amidohydrolase.	NF	NF	NF	NF
5726	3.5.1.89	N-acetylglucosaminylphosphatidylinositol deacetyla	NF	NF	NF	NF
5727	3.5.1.9	Arylformamidase.	NF	NF	NF	NF
5728	3.5.1.90	Adenosylcobinamide hydrolase.	NF	NF	NF	NF
5729	3.5.1.94	Gamma-glutamyl-gamma-aminobutyrate hydrolase	NF	NF	NF	NF
5730	3.5.2.-	In cyclic amides.	NF	NF	FOUND	FOUND
5731	3.5.2.14	N-methylhydantoinase (ATP-hydrolyzing).	NF	NF	NF	NF
5732	3.5.2.15	Cyanuric acid amidohydrolase.	NF	NF	NF	NF
5733	3.5.2.17	Hydroxyisourate hydrolase.	NF	NF	NF	NF
5734	3.5.2.2	Dihydropyrimidinase.	NF	NF	NF	FOUND
5735	3.5.2.3	Dihydroorotase.	NF	NF	FOUND	FOUND
5736	3.5.2.5	Allantoinase.	NF	NF	NF	NF
5737	3.5.2.7	Imidazolonepropionase.	NF	NF	NF	NF
5738	3.5.3.1	Arginase.	NF	NF	NF	NF
5739	3.5.3.10	D-arginase.	NF	NF	NF	NF
5740	3.5.3.11	Agmatinase.	NF	NF	FOUND	NF
5741	3.5.3.12	Agmatine deiminase.	NF	NF	NF	FOUND
5742	3.5.3.19	Ureidoglycolate hydrolase.	NF	NF	NF	NF
5743	3.5.3.3	Creatinase.	NF	NF	NF	NF
5744	3.5.3.4	Allantoicase.	NF	NF	NF	NF
5745	3.5.3.6	Arginine deiminase.	NF	NF	NF	NF
5746	3.5.3.7	Guanidinobutyrase.	NF	NF	NF	NF
5747	3.5.3.8	Formimidoylglutamase.	NF	NF	NF	NF
5748	3.5.4.1	Cytosine deaminase.	NF	NF	NF	NF
5749	3.5.4.10	IMP cyclohydrolase.	NF	FOUND	FOUND	FOUND
5750	3.5.4.12	dCMP deaminase.	FOUND	NF	NF	NF
5751	3.5.4.13	dCTP deaminase.	NF	NF	NF	NF
5752	3.5.4.16	GTP cyclohydrolase I.	NF	NF	FOUND	FOUND
5753	3.5.4.19	Phosphoribosyl-AMP cyclohydrolase.	NF	NF	FOUND	FOUND
5754	3.5.4.2	Adenine deaminase.	NF	NF	NF	FOUND
5755	3.5.4.21	Creatinine deaminase.	NF	NF	NF	NF
5756	3.5.4.25	GTP cyclohydrolase II.	NF	NF	FOUND	FOUND
5757	3.5.4.26	Diaminohydroxyphosphoribosylaminopyrimidine de	NF	NF	NF	NF
5758	3.5.4.27	Methenyltetrahydromethanopterin cyclohydrolase.	NF	NF	NF	NF
5759	3.5.4.29	GTP cyclohydrolase IIa.	NF	NF	NF	NF
5760	3.5.4.3	Guanine deaminase.	NF	NF	NF	FOUND
5761	3.5.4.4	Adenosine deaminase.	NF	FOUND	NF	NF
5762	3.5.4.5	Cytidine deaminase.	FOUND	FOUND	FOUND	FOUND
5763	3.5.4.6	AMP deaminase.	NF	NF	NF	NF
5764	3.5.4.9	Methenyltetrahydrofolate cyclohydrolase.	FOUND	FOUND	FOUND	FOUND
5765	3.5.5.1	Nitrilase.	NF	NF	NF	NF
5766	3.5.5.5	Arylacetonitrilase.	NF	NF	NF	NF
5767	3.5.99.3	Hydroxydechloroatrazine ethylaminohydrolase.	NF	NF	NF	NF
5768	3.5.99.4	N-isopropylammelide isopropylaminohydrolase.	NF	NF	NF	NF
5769	3.5.99.6	Glucosamine-6-phosphate deaminase.	FOUND	FOUND	FOUND	FOUND

5770	3.6.1.-	In phosphorous-containing anhydrides.	FOUND	FOUND	FOUND	FOUND
5771	3.6.1.15	Nucleoside-triphosphatase.	FOUND	FOUND	FOUND	FOUND
5772	3.6.1.19	Nucleoside-triphosphate diphosphatase.	NF	NF	NF	NF
5773	3.6.1.23	dUTP diphosphatase.	FOUND	FOUND	NF	FOUND
5774	3.6.1.28	Thiamine-triphosphatase.	NF	NF	NF	NF
5775	3.6.1.31	Phosphoribosyl-ATP diphosphatase.	NF	NF	NF	NF
5776	3.6.1.8	ATP diphosphatase.	NF	NF	NF	NF
5777	3.6.1.9	Nucleotide diphosphatase.	NF	NF	NF	NF
5778	3.6.2.1	Adenylylsulfatase.	NF	NF	NF	NF
5779	3.6.3.14	H(+)-transporting two-sector ATPase.	FOUND	NF	FOUND	NF
5780	3.7.1.-	In ketonic substances.	NF	NF	NF	FOUND
5781	3.7.1.2	Fumarylacetoacetase.	NF	NF	NF	FOUND
5782	3.7.1.3	Kynureninase.	NF	NF	NF	NF
5783	3.7.1.5	Acylpyruvate hydrolase.	NF	NF	NF	NF
5784	3.7.1.8	2,6-dioxo-6-phenylhexa-3-enoate hydrolase.	NF	NF	NF	NF
5785	3.7.1.9	2-hydroxymuconate-semialdehyde hydrolase.	NF	NF	NF	NF
5786	3.8.1.-	In C-halide compounds.	NF	NF	NF	NF
5787	3.8.1.2	(S)-2-haloacid dehalogenase.	NF	NF	NF	NF
5788	3.8.1.3	Haloacetate dehalogenase.	NF	NF	NF	NF
5789	3.8.1.5	Haloalkane dehalogenase.	NF	NF	NF	NF
5790	3.8.1.7	4-chlorobenzoyl-CoA dehalogenase.	NF	NF	NF	NF
5791	3.8.1.8	Atrazine chlorohydrolase.	NF	NF	NF	NF
5792	4.-.-.-	Lyases.	FOUND	FOUND	FOUND	FOUND
5793	4.1.1.-	Carboxy-lyases.	FOUND	FOUND	FOUND	FOUND
5794	4.1.1.1	Pyruvate decarboxylase.	NF	NF	NF	NF
5795	4.1.1.11	Aspartate 1-decarboxylase.	NF	NF	NF	NF
5796	4.1.1.12	Aspartate 4-decarboxylase.	NF	NF	NF	NF
5797	4.1.1.15	Glutamate decarboxylase.	NF	NF	NF	NF
5798	4.1.1.17	Ornithine decarboxylase.	NF	NF	NF	NF
5799	4.1.1.18	Lysine decarboxylase.	NF	NF	NF	NF
5800	4.1.1.19	Arginine decarboxylase.	NF	NF	FOUND	NF
5801	4.1.1.2	Oxalate decarboxylase.	NF	NF	NF	NF
5802	4.1.1.20	Diaminopimelate decarboxylase.	NF	NF	FOUND	FOUND
5803	4.1.1.21	Phosphoribosylaminoimidazole carboxylase.	NF	FOUND	FOUND	FOUND
5804	4.1.1.22	Histidine decarboxylase.	NF	NF	NF	NF
5805	4.1.1.23	Orotidine-5'-phosphate decarboxylase.	NF	NF	FOUND	FOUND
5806	4.1.1.25	Tyrosine decarboxylase.	NF	NF	NF	NF
5807	4.1.1.28	Aromatic-L-amino-acid decarboxylase.	NF	NF	NF	NF
5808	4.1.1.29	Sulfinoalanine decarboxylase.	NF	NF	NF	NF
5809	4.1.1.3	Oxaloacetate decarboxylase.	NF	NF	NF	NF
5810	4.1.1.31	Phosphoenolpyruvate carboxylase.	FOUND	FOUND	FOUND	NF
5811	4.1.1.32	Phosphoenolpyruvate carboxykinase (GTP).	NF	NF	NF	NF
5812	4.1.1.33	Diphosphomevalonate decarboxylase.	FOUND	FOUND	FOUND	NF
5813	4.1.1.34	Dehydro-L-gulonate decarboxylase.	NF	NF	NF	NF
5814	4.1.1.35	UDP-glucuronate decarboxylase.	NF	NF	NF	NF
5815	4.1.1.36	Phosphopantothenoylcysteine decarboxylase.	NF	NF	NF	NF
5816	4.1.1.37	Uroporphyrinogen decarboxylase.	NF	NF	NF	NF
5817	4.1.1.39	Ribulose-bisphosphate carboxylase.	NF	NF	NF	NF
5818	4.1.1.4	Acetoacetate decarboxylase.	NF	NF	NF	NF
5819	4.1.1.43	Phenylpyruvate decarboxylase.	NF	NF	NF	NF
5820	4.1.1.44	4-carboxymuconolactone decarboxylase.	NF	NF	NF	NF
5821	4.1.1.45	Aminocarboxymuconate-semialdehyde decarboxyla	NF	NF	NF	NF
5822	4.1.1.47	Tartronate-semialdehyde synthase.	NF	NF	NF	NF
5823	4.1.1.48	Indole-3-glycerol-phosphate synthase.	NF	NF	FOUND	FOUND
5824	4.1.1.49	Phosphoenolpyruvate carboxykinase (ATP).	NF	NF	FOUND	FOUND

5825	4.1.1.50	Adenosylmethionine decarboxylase.	NF	NF	FOUND	NF
5826	4.1.1.55	4,5-dihydroxyphthalate decarboxylase.	NF	NF	NF	NF
5827	4.1.1.65	Phosphatidylserine decarboxylase.	NF	NF	NF	FOUND
5828	4.1.1.67	UDP-galacturonate decarboxylase.	NF	NF	NF	NF
5829	4.1.1.74	Indolepyruvate decarboxylase.	NF	NF	NF	NF
5830	4.1.1.75	5-guanidino-2-oxopentanoate decarboxylase.	NF	NF	NF	NF
5831	4.1.1.77	4-oxalocrotonate decarboxylase.	NF	NF	NF	NF
5832	4.1.1.8	Oxalyl-CoA decarboxylase.	NF	NF	NF	NF
5833	4.1.1.82	Phosphonopyruvate decarboxylase.	NF	NF	NF	NF
5834	4.1.1.9	Malonyl-CoA decarboxylase.	NF	NF	NF	NF
5835	4.1.2.-	Aldehyde-lyases.	FOUND	FOUND	FOUND	FOUND
5836	4.1.2.10	Mandelonitrile lyase.	NF	NF	NF	NF
5837	4.1.2.11	Hydroxymandelonitrile lyase.	NF	NF	NF	NF
5838	4.1.2.13	Fructose-bisphosphate aldolase.	FOUND	FOUND	FOUND	FOUND
5839	4.1.2.14	2-dehydro-3-deoxy-phosphogluconate aldolase.	FOUND	FOUND	NF	FOUND
5840	4.1.2.20	2-dehydro-3-deoxyglucarate aldolase.	NF	NF	NF	NF
5841	4.1.2.25	Dihydroneopterin aldolase.	NF	NF	FOUND	NF
5842	4.1.2.27	Sphinganine-1-phosphate aldolase.	NF	NF	NF	NF
5843	4.1.2.29	5-dehydro-2-deoxyphosphogluconate aldolase.	NF	NF	NF	NF
5844	4.1.2.30	17-alpha-hydroxyprogesterone aldolase.	NF	NF	NF	NF
5845	4.1.2.45	Trans-o-hydroxybenzylidenepyruvate hydratase-ald	NF	NF	NF	NF
5846	4.1.2.46	???	NF	NF	NF	NF
5847	4.1.2.47	???	NF	NF	NF	NF
5848	4.1.2.5	Threonine aldolase.	NF	NF	FOUND	NF
5849	4.1.2.9	Phosphoketolase.	NF	NF	NF	NF
5850	4.1.3.1	Isocitrate lyase.	NF	NF	NF	NF
5851	4.1.3.25	Citramalyl-CoA lyase.	NF	NF	NF	NF
5852	4.1.3.27	Anthranilate synthase.	NF	NF	FOUND	FOUND
5853	4.1.3.36	1,4-dihydroxy-2-naphthoyl-CoA synthase.	NF	NF	NF	NF
5854	4.1.3.39	4-hydroxy-2-oxovalerate aldolase.	NF	NF	NF	NF
5855	4.1.3.4	Hydroxymethylglutaryl-CoA lyase.	NF	NF	NF	NF
5856	4.1.3.40	Chorismate lyase.	NF	NF	NF	NF
5857	4.1.99.11	Benzylsuccinate synthase.	NF	NF	NF	NF
5858	4.1.99.12	3,4-dihydroxy-2-butanone-4-phosphate synthase.	NF	NF	NF	NF
5859	4.2.1.-	Hydro-lyases.	FOUND	FOUND	FOUND	FOUND
5860	4.2.1.10	3-dehydroquinate dehydratase.	NF	NF	FOUND	FOUND
5861	4.2.1.100	Cyclohexa-1,5-dienecarbonyl-CoA hydratase.	NF	NF	NF	NF
5862	4.2.1.101	Trans-feruloyl-CoA hydratase.	NF	NF	NF	NF
5863	4.2.1.107	3-alpha,7-alpha,12-alpha-trihydroxy-5-beta-cholest-	NF	NF	NF	NF
5864	4.2.1.108	Ectoine synthase.	NF	NF	NF	NF
5865	4.2.1.109	Methylthioribulose 1-phosphate dehydratase.	NF	NF	NF	NF
5866	4.2.1.11	Phosphopyruvate hydratase.	FOUND	FOUND	FOUND	FOUND
5867	4.2.1.113	o-succinylbenzoate synthase.	NF	NF	NF	NF
5868	4.2.1.12	Phosphogluconate dehydratase.	NF	NF	NF	NF
5869	4.2.1.17	Enoyl-CoA hydratase.	NF	NF	NF	NF
5870	4.2.1.18	Methylglutaconyl-CoA hydratase.	NF	NF	NF	NF
5871	4.2.1.19	Imidazoleglycerol-phosphate dehydratase.	NF	NF	FOUND	FOUND
5872	4.2.1.2	Fumarate hydratase.	NF	NF	NF	FOUND
5873	4.2.1.20	Tryptophan synthase.	NF	NF	FOUND	FOUND
5874	4.2.1.22	Cystathionine beta-synthase.	NF	NF	NF	NF
5875	4.2.1.24	Porphobilinogen synthase.	NF	NF	FOUND	FOUND
5876	4.2.1.25	L-arabinonate dehydratase.	NF	NF	NF	NF
5877	4.2.1.3	Aconitate hydratase.	NF	NF	FOUND	NF
5878	4.2.1.33	3-isopropylmalate dehydratase.	NF	NF	FOUND	FOUND
5879	4.2.1.34	(S)-2-methylmalate dehydratase.	NF	NF	NF	NF

5880	4.2.1.36	Homoaconitate hydratase.	NF	NF	NF	NF
5881	4.2.1.39	Gluconate dehydratase.	NF	NF	NF	NF
5882	4.2.1.40	Glucarate dehydratase.	NF	NF	NF	NF
5883	4.2.1.41	5-dehydro-4-deoxyglucarate dehydratase.	NF	NF	NF	NF
5884	4.2.1.43	2-dehydro-3-deoxy-L-arabinonate dehydratase.	NF	NF	NF	NF
5885	4.2.1.44	Myo-inosose-2 dehydratase.	NF	NF	NF	FOUND
5886	4.2.1.46	dTDP-glucose 4,6-dehydratase.	NF	NF	FOUND	FOUND
5887	4.2.1.47	GDP-mannose 4,6-dehydratase.	NF	NF	NF	NF
5888	4.2.1.49	Urocanate hydratase.	NF	NF	NF	NF
5889	4.2.1.51	Prephenate dehydratase.	NF	NF	FOUND	FOUND
5890	4.2.1.52	Dihydrodipicolinate synthase.	FOUND	FOUND	FOUND	FOUND
5891	4.2.1.54	Lactoyl-CoA dehydratase.	NF	NF	NF	NF
5892	4.2.1.58	Crotonoyl-[acyl-carrier-protein] hydratase.	NF	NF	NF	NF
5893	4.2.1.60	3-hydroxydecanoyl-[acyl-carrier-protein] dehydrata	NF	NF	NF	NF
5894	4.2.1.61	3-hydroxypalmitoyl-[acyl-carrier-protein] dehydrata	NF	NF	NF	NF
5895	4.2.1.65	3-cyanoalanine hydratase.	NF	NF	NF	NF
5896	4.2.1.7	Altronate dehydratase.	NF	NF	NF	FOUND
5897	4.2.1.74	Long-chain-enoyl-CoA hydratase.	NF	NF	NF	NF
5898	4.2.1.75	Uroporphyrinogen-III synthase.	NF	NF	FOUND	FOUND
5899	4.2.1.78	(S)-norcoclaurine synthase.	NF	NF	NF	NF
5900	4.2.1.8	Mannonate dehydratase.	FOUND	FOUND	NF	FOUND
5901	4.2.1.80	2-oxopent-4-enoate hydratase.	NF	NF	NF	NF
5902	4.2.1.9	Dihydroxy-acid dehydratase.	NF	NF	FOUND	FOUND
5903	4.2.1.91	Arogenate dehydratase.	NF	NF	NF	NF
5904	4.2.1.92	Hydroperoxide dehydratase.	NF	NF	NF	NF
5905	4.2.3.-	Acting on phosphates.	NF	NF	FOUND	FOUND
5906	4.2.3.1	Threonine synthase.	NF	NF	FOUND	FOUND
5907	4.2.3.11	Sabinene-hydrate synthase.	NF	NF	NF	NF
5908	4.2.3.12	6-pyruvoyltetrahydropterin synthase.	NF	NF	NF	NF
5909	4.2.3.13	(+)-delta-cadinene synthase.	NF	NF	NF	NF
5910	4.2.3.14	Pinene synthase.	NF	NF	NF	NF
5911	4.2.3.16	(4S)-limonene synthase.	NF	NF	NF	NF
5912	4.2.3.17	Taxadiene synthase.	NF	NF	NF	NF
5913	4.2.3.18	Abietadiene synthase.	NF	NF	NF	NF
5914	4.2.3.19	Ent-kaurene synthase.	NF	NF	NF	NF
5915	4.2.3.20	(R)-limonene synthase.	NF	NF	NF	NF
5916	4.2.3.22	Germacradienol synthase.	NF	NF	NF	NF
5917	4.2.3.38	Alpha-bisabolene synthase.	NF	NF	NF	NF
5918	4.2.3.4	3-dehydroquinate synthase.	NF	NF	FOUND	FOUND
5919	4.2.3.5	Chorismate synthase.	NF	NF	FOUND	FOUND
5920	4.2.3.8	Casbene synthase.	NF	NF	NF	NF
5921	4.2.3.9	Aristolochene synthase.	NF	NF	NF	NF
5922	4.2.99.20	2-succinyl-6-hydroxy-2,4-cyclohexadiene-1-carboxy	NF	NF	NF	NF
5923	4.3.1.1	Aspartate ammonia-lyase.	NF	NF	NF	NF
5924	4.3.1.17	L-serine ammonia-lyase.	FOUND	NF	NF	FOUND
5925	4.3.1.19	Threonine ammonia-lyase.	FOUND	FOUND	FOUND	FOUND
5926	4.3.1.2	Methylaspartate ammonia-lyase.	NF	NF	NF	NF
5927	4.3.1.24	Phenylalanine ammonia-lyase.	NF	NF	NF	NF
5928	4.3.1.25	Phenylalanine/tyrosine ammonia-lyase.	NF	NF	NF	NF
5929	4.3.1.3	Histidine ammonia-lyase.	NF	NF	NF	NF
5930	4.3.1.4	Formimidoyltetrahydrofolate cyclodeaminase.	NF	NF	NF	NF
5931	4.3.2.1	Argininosuccinate lyase.	NF	NF	FOUND	FOUND
5932	4.3.2.2	Adenylosuccinate lyase.	FOUND	FOUND	FOUND	FOUND
5933	4.3.3.2	Strictosidine synthase.	NF	NF	NF	NF
5934	4.3.3.3	Deacetylisoipecoside synthase.	NF	NF	NF	NF

5935	4.4.1.1	Cystathionine gamma-lyase.	NF	NF	FOUND	NF
5936	4.4.1.10	Cysteine lyase.	NF	NF	NF	NF
5937	4.4.1.14	1-aminocyclopropane-1-carboxylate synthase.	NF	NF	NF	NF
5938	4.4.1.16	Selenocysteine lyase.	NF	NF	NF	NF
5939	4.4.1.20	Leukotriene-C(4) synthase.	NF	NF	NF	NF
5940	4.4.1.8	Cystathionine beta-lyase.	FOUND	NF	FOUND	NF
5941	4.4.1.9	L-3-cyanoalanine synthase.	NF	NF	NF	NF
5942	4.5.1.-	Carbon-halide lyases.	NF	NF	NF	NF
5943	4.5.1.1	DDT-dehydrochlorinase.	NF	NF	NF	NF
5944	4.6.1.12	2-C-methyl-D-erythritol 2,4-cyclodiphosphate synth	NF	NF	NF	FOUND
5945	4.99.1.1	Ferrochelatase.	NF	NF	NF	NF
5946	4.99.1.3	Sirohydrochlorin cobaltochelatase.	NF	NF	NF	NF
5947	4.99.1.4	Sirohydrochlorin ferrochelatase.	NF	NF	NF	NF
5948	4.99.1.5	Aliphatic aldoxime dehydratase.	NF	NF	NF	NF
5949	5.-.-.-	Isomerases.	FOUND	FOUND	FOUND	FOUND
5950	5.1.1.1	Alanine racemase.	FOUND	FOUND	FOUND	FOUND
5951	5.1.1.10	Amino-acid racemase.	NF	NF	NF	NF
5952	5.1.1.17	Isopenicillin-N epimerase.	NF	NF	NF	NF
5953	5.1.1.3	Glutamate racemase.	FOUND	FOUND	FOUND	FOUND
5954	5.1.1.4	Proline racemase.	NF	NF	NF	NF
5955	5.1.1.7	Diaminopimelate epimerase.	FOUND	FOUND	FOUND	FOUND
5956	5.1.1.9	Arginine racemase.	NF	NF	NF	NF
5957	5.1.2.-	Acting on hydroxy acids and derivatives.	NF	NF	NF	NF
5958	5.1.3.1	Ribulose-phosphate 3-epimerase.	FOUND	FOUND	FOUND	FOUND
5959	5.1.3.12	UDP-glucuronate 5'-epimerase.	NF	NF	NF	NF
5960	5.1.3.13	dTDP-4-dehydrorhamnose 3,5-epimerase.	NF	NF	FOUND	FOUND
5961	5.1.3.14	UDP-N-acetylglucosamine 2-epimerase.	NF	NF	NF	NF
5962	5.1.3.15	Glucose-6-phosphate 1-epimerase.	NF	NF	NF	NF
5963	5.1.3.17	Heparosan-N-sulfate-glucuronate 5-epimerase.	NF	NF	NF	NF
5964	5.1.3.18	GDP-mannose 3,5-epimerase.	NF	NF	NF	NF
5965	5.1.3.2	UDP-glucose 4-epimerase.	NF	FOUND	FOUND	FOUND
5966	5.1.3.20	ADP-glyceromanno-heptose 6-epimerase.	NF	NF	NF	FOUND
5967	5.1.3.4	L-ribulose-5-phosphate 4-epimerase.	FOUND	FOUND	NF	FOUND
5968	5.1.3.5	UDP-arabinose 4-epimerase.	NF	NF	NF	NF
5969	5.1.3.6	UDP-glucuronate 4-epimerase.	NF	NF	NF	NF
5970	5.1.3.7	UDP-N-acetylglucosamine 4-epimerase.	NF	NF	NF	NF
5971	5.1.99.1	Methylmalonyl-CoA epimerase.	NF	NF	NF	NF
5972	5.1.99.4	Alpha-methylacyl-CoA racemase.	NF	NF	NF	NF
5973	5.2.1.10	2-chloro-4-carboxymethylenebut-2-en-1,4-olide iso	NF	NF	NF	NF
5974	5.2.1.2	Maleylacetoacetate isomerase.	NF	NF	NF	NF
5975	5.2.1.3	Retinal isomerase.	NF	NF	NF	NF
5976	5.2.1.4	Maleylpyruvate isomerase.	NF	NF	NF	NF
5977	5.3.1.-	Interconverting aldoses and ketoses, and related co	FOUND	FOUND	FOUND	FOUND
5978	5.3.1.1	Triose-phosphate isomerase.	FOUND	FOUND	FOUND	FOUND
5979	5.3.1.12	Glucuronate isomerase.	FOUND	FOUND	NF	FOUND
5980	5.3.1.16	1-(5-phosphoribosyl)-5- ((5-phosphoribosylamino)m	NF	NF	FOUND	FOUND
5981	5.3.1.22	Hydroxypyruvate isomerase.	NF	NF	NF	NF
5982	5.3.1.23	S-methyl-5-thioribose-1-phosphate isomerase.	NF	NF	NF	NF
5983	5.3.1.24	Phosphoribosylanthranilate isomerase.	NF	NF	FOUND	FOUND
5984	5.3.1.4	L-arabinose isomerase.	NF	NF	NF	NF
5985	5.3.1.5	Xylose isomerase.	FOUND	NF	NF	FOUND
5986	5.3.1.6	Ribose-5-phosphate isomerase.	FOUND	FOUND	FOUND	FOUND
5987	5.3.1.7	Mannose isomerase.	NF	NF	NF	NF
5988	5.3.1.8	Mannose-6-phosphate isomerase.	FOUND	FOUND	FOUND	FOUND
5989	5.3.1.9	Glucose-6-phosphate isomerase.	FOUND	FOUND	FOUND	FOUND

5990	5.3.2.-	Interconverting keto- and enol- groups.	NF	NF	NF	NF
5991	5.3.3.1	Steroid Delta-isomerase.	NF	NF	NF	NF
5992	5.3.3.12	L-dopachrome isomerase.	NF	NF	NF	NF
5993	5.3.3.2	Isopentenyl-diphosphate Delta-isomerase.	FOUND	FOUND	FOUND	FOUND
5994	5.3.3.4	Muconolactone Delta-isomerase.	NF	NF	NF	NF
5995	5.3.3.5	Cholestenol Delta-isomerase.	NF	NF	NF	NF
5996	5.3.99.2	Prostaglandin-D synthase.	NF	NF	NF	NF
5997	5.3.99.3	Prostaglandin-E synthase.	NF	NF	NF	NF
5998	5.3.99.4	Prostaglandin-I synthase.	NF	NF	NF	NF
5999	5.3.99.5	Thromboxane-A synthase.	NF	NF	NF	NF
6000	5.3.99.6	Allene-oxide cyclase.	NF	NF	NF	NF
6001	5.3.99.7	Styrene-oxide isomerase.	NF	NF	NF	NF
6002	5.4.1.2	Precorrin-8X methylmutase.	NF	NF	FOUND	FOUND
6003	5.4.2.1	Phosphoglycerate mutase.	FOUND	FOUND	FOUND	FOUND
6004	5.4.2.10	Phosphoglucosamine mutase.	FOUND	FOUND	FOUND	FOUND
6005	5.4.2.2	Phosphoglucomutase.	FOUND	FOUND	FOUND	FOUND
6006	5.4.2.8	Phosphomannomutase.	NF	NF	NF	FOUND
6007	5.4.2.9	Phosphoenolpyruvate mutase.	NF	NF	NF	NF
6008	5.4.3.8	Glutamate-1-semialdehyde 2,1-aminomutase.	NF	NF	FOUND	NF
6009	5.4.4.2	Isochorismate synthase.	NF	NF	NF	NF
6010	5.4.99.1	Methylaspartate mutase.	NF	NF	NF	NF
6011	5.4.99.18	5-(carboxyamino)imidazole ribonucleotide mutase.	NF	NF	NF	NF
6012	5.4.99.2	Methylmalonyl-CoA mutase.	NF	NF	NF	NF
6013	5.4.99.5	Chorismate mutase.	NF	NF	FOUND	FOUND
6014	5.4.99.7	Lanosterol synthase.	NF	NF	NF	NF
6015	5.4.99.8	Cycloartenol synthase.	NF	NF	NF	NF
6016	5.5.1.1	Muconate cycloisomerase.	NF	NF	NF	NF
6017	5.5.1.12	Copalyl diphosphate synthase.	NF	NF	NF	NF
6018	5.5.1.13	Ent-copalyl diphosphate synthase.	NF	NF	NF	NF
6019	5.5.1.2	3-carboxy-cis,cis-muconate cycloisomerase.	NF	NF	NF	NF
6020	5.5.1.4	Inositol-3-phosphate synthase.	NF	NF	NF	NF
6021	5.5.1.6	Chalcone isomerase.	NF	NF	NF	NF
6022	5.5.1.7	Chloromuconate cycloisomerase.	NF	NF	NF	NF
6023	5.5.1.8	Bornyl diphosphate synthase.	NF	NF	NF	NF
6024	5.5.1.9	Cycloeucalenol cycloisomerase.	NF	NF	NF	NF
6025	5.99.1.4	2-hydroxychromene-2-carboxylate isomerase.	NF	NF	NF	NF
6026	6.-.-.-	Ligases.	FOUND	FOUND	FOUND	FOUND
6027	6.1.1.17	Glutamate--tRNA ligase.	FOUND	FOUND	FOUND	FOUND
6028	6.1.1.18	Glutamine--tRNA ligase.	NF	NF	NF	NF
6029	6.1.1.24	Glutamate--tRNA(Gln) ligase.	NF	NF	NF	NF
6030	6.2.1.-	Acid--thiol ligases.	NF	NF	NF	NF
6031	6.2.1.1	Acetate--CoA ligase.	NF	NF	NF	NF
6032	6.2.1.11	Biotin--CoA ligase.	NF	NF	NF	NF
6033	6.2.1.12	4-coumarate--CoA ligase.	NF	NF	NF	NF
6034	6.2.1.13	Acetate--CoA ligase (ADP-forming).	NF	NF	NF	NF
6035	6.2.1.14	6-carboxyhexanoate--CoA ligase.	NF	NF	NF	NF
6036	6.2.1.17	Propionate--CoA ligase.	NF	NF	NF	NF
6037	6.2.1.2	Butyrate--CoA ligase.	NF	NF	NF	NF
6038	6.2.1.25	Benzoate--CoA ligase.	NF	NF	NF	NF
6039	6.2.1.26	o-succinylbenzoate--CoA ligase.	NF	NF	NF	NF
6040	6.2.1.27	4-hydroxybenzoate--CoA ligase.	NF	NF	NF	NF
6041	6.2.1.3	Long-chain-fatty-acid--CoA ligase.	NF	NF	NF	NF
6042	6.2.1.33	4-chlorobenzoate--CoA ligase.	NF	NF	NF	NF
6043	6.2.1.4	Succinate--CoA ligase (GDP-forming).	NF	NF	NF	NF
6044	6.2.1.5	Succinate--CoA ligase (ADP-forming).	NF	NF	NF	NF

6045	6.2.1.7	Cholate--CoA ligase.	NF	NF	NF	NF
6046	6.2.1.8	Oxalate--CoA ligase.	NF	NF	NF	NF
6047	6.3.1.-	Acid--ammonia (or amide) ligases (amide synthases)	FOUND	FOUND	FOUND	FOUND
6048	6.3.1.1	Aspartate--ammonia ligase.	FOUND	FOUND	FOUND	FOUND
6049	6.3.1.10	Adenosylcobinamide-phosphate synthase.	NF	NF	NF	NF
6050	6.3.1.11	Glutamate--putrescine ligase.	NF	NF	NF	NF
6051	6.3.1.2	Glutamate--ammonia ligase.	NF	FOUND	FOUND	FOUND
6052	6.3.1.5	NAD(+) synthase.	NF	NF	NF	FOUND
6053	6.3.1.8	Glutathionylspermidine synthase.	NF	NF	NF	NF
6054	6.3.1.9	Trypanothione synthase.	NF	NF	NF	NF
6055	6.3.2.1	Pantoate--beta-alanine ligase.	NF	NF	NF	NF
6056	6.3.2.10	UDP-N-acetylmuramoyl-tripeptide--D-alanyl-D-alani	NF	NF	NF	NF
6057	6.3.2.12	Dihydrofolate synthase.	NF	NF	NF	NF
6058	6.3.2.17	Tetrahydrofolate synthase.	FOUND	FOUND	FOUND	FOUND
6059	6.3.2.2	Glutamate--cysteine ligase.	NF	NF	NF	NF
6060	6.3.2.26	N-(5-amino-5-carboxypentanoyl)-L-cysteinyl-D-valin	NF	NF	NF	NF
6061	6.3.2.3	Glutathione synthase.	NF	NF	NF	NF
6062	6.3.2.4	D-alanine--D-alanine ligase.	FOUND	FOUND	FOUND	FOUND
6063	6.3.2.5	Phosphopantothenate--cysteine ligase.	FOUND	FOUND	FOUND	FOUND
6064	6.3.2.6	Phosphoribosylaminoimidazolesuccinocarboxamide	NF	FOUND	FOUND	FOUND
6065	6.3.2.7	UDP-N-acetylmuramoyl-L-alanyl-D-glutamate--L-lysi	NF	NF	NF	NF
6066	6.3.2.8	UDP-N-acetylmuramate--L-alanine ligase.	FOUND	FOUND	FOUND	FOUND
6067	6.3.2.9	UDP-N-acetylmuramoyl-L-alanine--D-glutamate liga	FOUND	FOUND	FOUND	FOUND
6068	6.3.3.1	Phosphoribosylformylglycinamidine cyclo-ligase.	NF	FOUND	FOUND	FOUND
6069	6.3.3.2	5-formyltetrahydrofolate cyclo-ligase.	NF	NF	FOUND	FOUND
6070	6.3.3.3	Dethiobiotin synthase.	FOUND	FOUND	FOUND	FOUND
6071	6.3.4.-	Other carbon--nitrogen ligases.	FOUND	FOUND	FOUND	FOUND
6072	6.3.4.1	GMP synthase.	NF	NF	NF	NF
6073	6.3.4.10	Biotin--[propionyl-CoA-carboxylase (ATP-hydrolyzin	NF	NF	NF	NF
6074	6.3.4.11	Biotin--[methylcrotonoyl-CoA-carboxylase] ligase.	NF	NF	NF	NF
6075	6.3.4.13	Phosphoribosylamine--glycine ligase.	NF	FOUND	FOUND	FOUND
6076	6.3.4.14	Biotin carboxylase.	NF	NF	FOUND	FOUND
6077	6.3.4.15	Biotin--[acetyl-CoA-carboxylase] ligase.	NF	NF	FOUND	FOUND
6078	6.3.4.16	Carbamoyl-phosphate synthase (ammonia).	NF	NF	NF	NF
6079	6.3.4.18	5-(carboxyamino)imidazole ribonucleotide synthase	NF	NF	NF	NF
6080	6.3.4.2	CTP synthase.	NF	FOUND	FOUND	FOUND
6081	6.3.4.3	Formate--tetrahydrofolate ligase.	FOUND	FOUND	FOUND	FOUND
6082	6.3.4.4	Adenylosuccinate synthase.	FOUND	FOUND	FOUND	FOUND
6083	6.3.4.5	Argininosuccinate synthase.	NF	NF	FOUND	FOUND
6084	6.3.4.6	Urea carboxylase.	NF	NF	NF	NF
6085	6.3.4.9	Biotin--[methylmalonyl-CoA-carboxytransferase] lig	NF	NF	NF	NF
6086	6.3.5.1	NAD(+) synthase (glutamine-hydrolyzing).	NF	NF	FOUND	FOUND
6087	6.3.5.10	Adenosylcobyric acid synthase (glutamine-hydrolyzi	NF	NF	NF	NF
6088	6.3.5.2	GMP synthase (glutamine-hydrolyzing).	FOUND	FOUND	FOUND	FOUND
6089	6.3.5.3	Phosphoribosylformylglycinamidine synthase.	NF	FOUND	FOUND	FOUND
6090	6.3.5.4	Asparagine synthase (glutamine-hydrolyzing).	NF	NF	NF	FOUND
6091	6.3.5.5	Carbamoyl-phosphate synthase (glutamine-hydroly:	NF	NF	FOUND	FOUND
6092	6.3.5.7	Glutaminyl-tRNA synthase (glutamine-hydrolyzing).	NF	NF	NF	NF
6093	6.3.5.9	Hydrogenobyrinic acid a,c-diamide synthase (glutan	NF	NF	NF	NF
6094	6.4.1.1	Pyruvate carboxylase.	NF	NF	NF	NF
6095	6.4.1.2	Acetyl-CoA carboxylase.	NF	NF	FOUND	FOUND
6096	6.4.1.3	Propionyl-CoA carboxylase.	NF	NF	NF	NF
6097	6.4.1.4	Methylcrotonoyl-CoA carboxylase.	NF	NF	NF	NF
6098	6.6.1.1	Magnesium chelatase.	NF	NF	FOUND	FOUND
6099	6.6.1.2	Cobaltochelatase.	NF	NF	NF	NF

6100	>Biosynthesis of secondary metabolites 01110					
6101	1.-.-.-	Oxidoreductases.	FOUND	FOUND	FOUND	FOUND
6102	1.1.-.-	Acting on the CH-OH group of donors.	FOUND	FOUND	FOUND	FOUND
6103	1.1.1.-	With NAD(+) or NADP(+) as acceptor.	FOUND	FOUND	FOUND	FOUND
6104	1.1.1.1	Alcohol dehydrogenase.	FOUND	FOUND	FOUND	FOUND
6105	1.1.1.122	D-threo-aldose 1-dehydrogenase.	NF	NF	NF	NF
6106	1.1.1.133	dTDP-4-dehydrorhamnose reductase.	NF	NF	FOUND	FOUND
6107	1.1.1.144	Perillyl-alcohol dehydrogenase.	NF	NF	NF	NF
6108	1.1.1.169	2-dehydropantoate 2-reductase.	FOUND	FOUND	NF	NF
6109	1.1.1.178	3-hydroxy-2-methylbutyryl-CoA dehydrogenase.	NF	NF	NF	NF
6110	1.1.1.18	Inositol 2-dehydrogenase.	NF	NF	NF	NF
6111	1.1.1.183	Geraniol dehydrogenase.	NF	NF	NF	NF
6112	1.1.1.195	Cinnamyl-alcohol dehydrogenase.	NF	NF	NF	NF
6113	1.1.1.2	Alcohol dehydrogenase (NADP(+)).	NF	NF	FOUND	NF
6114	1.1.1.205	IMP dehydrogenase.	FOUND	FOUND	FOUND	FOUND
6115	1.1.1.206	Tropinone reductase I.	NF	NF	NF	NF
6116	1.1.1.207	(-)-menthol dehydrogenase.	NF	NF	NF	NF
6117	1.1.1.218	Morphine 6-dehydrogenase.	NF	NF	NF	NF
6118	1.1.1.219	Dihydrokaempferol 4-reductase.	NF	NF	NF	NF
6119	1.1.1.22	UDP-glucose 6-dehydrogenase.	NF	NF	NF	FOUND
6120	1.1.1.223	Isopiperitenol dehydrogenase.	NF	NF	NF	NF
6121	1.1.1.23	Histidinol dehydrogenase.	NF	NF	FOUND	FOUND
6122	1.1.1.237	Hydroxyphenylpyruvate reductase.	NF	NF	NF	NF
6123	1.1.1.246	Pterocarpin synthase.	NF	NF	NF	NF
6124	1.1.1.247	Codeinone reductase (NADPH).	NF	NF	NF	NF
6125	1.1.1.248	Salutaridine reductase (NADPH).	NF	NF	NF	NF
6126	1.1.1.25	Shikimate dehydrogenase.	FOUND	FOUND	FOUND	FOUND
6127	1.1.1.267	1-deoxy-D-xylulose-5-phosphate reductoisomerase.	NF	NF	NF	NF
6128	1.1.1.27	L-lactate dehydrogenase.	FOUND	FOUND	NF	FOUND
6129	1.1.1.273	Vellosimine dehydrogenase.	NF	NF	NF	NF
6130	1.1.1.282	Quinate/shikimate dehydrogenase.	NF	NF	NF	NF
6131	1.1.1.288	Xanthoxin dehydrogenase.	NF	NF	NF	NF
6132	1.1.1.294	Chlorophyll(ide) b reductase.	NF	NF	NF	NF
6133	1.1.1.295	Momilactone-A synthase.	NF	NF	NF	NF
6134	1.1.1.297	Limonene-1,2-diol dehydrogenase.	NF	NF	NF	NF
6135	1.1.1.3	Homoserine dehydrogenase.	NF	NF	FOUND	FOUND
6136	1.1.1.34	Hydroxymethylglutaryl-CoA reductase (NADPH).	FOUND	FOUND	FOUND	FOUND
6137	1.1.1.35	3-hydroxyacyl-CoA dehydrogenase.	NF	NF	NF	NF
6138	1.1.1.37	Malate dehydrogenase.	NF	NF	NF	NF
6139	1.1.1.41	Isocitrate dehydrogenase (NAD(+)).	NF	NF	FOUND	FOUND
6140	1.1.1.42	Isocitrate dehydrogenase (NADP(+)).	NF	NF	FOUND	FOUND
6141	1.1.1.44	Phosphogluconate dehydrogenase (decarboxylating	NF	NF	NF	NF
6142	1.1.1.49	Glucose-6-phosphate dehydrogenase.	NF	NF	NF	NF
6143	1.1.1.85	3-isopropylmalate dehydrogenase.	NF	NF	FOUND	FOUND
6144	1.1.1.86	Ketol-acid reductoisomerase.	NF	NF	FOUND	FOUND
6145	1.1.1.88	Hydroxymethylglutaryl-CoA reductase.	NF	NF	NF	NF
6146	1.1.2.7	Methanol dehydrogenase (cytochrome c).	NF	NF	NF	NF
6147	1.1.2.8	Alcohol dehydrogenase (cytochrome c).	NF	NF	NF	NF
6148	1.1.3.-	With oxygen as acceptor.	NF	NF	NF	FOUND
6149	1.1.3.16	Ecdysone oxidase.	NF	NF	NF	NF
6150	1.1.5.2	Quinoprotein glucose dehydrogenase.	NF	NF	NF	NF
6151	1.1.5.8	Quinate dehydrogenase (quinone).	NF	NF	NF	NF
6152	1.1.99.10	Glucose dehydrogenase (acceptor).	NF	NF	NF	NF
6153	1.10.3.-	With oxygen as acceptor.	NF	NF	NF	NF
6154	1.10.3.1	Catechol oxidase.	NF	NF	NF	NF

6155	1.10.99.3	Violaxanthin de-epoxidase.	NF	NF	NF	NF
6156	1.11.1.7	Peroxidase.	FOUND	FOUND	FOUND	FOUND
6157	1.13.11.51	9-cis-epoxycarotenoid dioxygenase.	NF	NF	NF	NF
6158	1.13.12.-	With incorporation of one atom of oxygen.	NF	NF	FOUND	NF
6159	1.13.12.14	Chlorophyllide-a oxygenase.	NF	NF	NF	NF
6160	1.14.-.-	Acting on paired donors, with incorporation or redu	NF	NF	NF	NF
6161	1.14.11.11	Hyoscyamine (6S)-dioxygenase.	NF	NF	NF	NF
6162	1.14.11.12	Gibberellin-44 dioxygenase.	NF	NF	NF	NF
6163	1.14.11.14	6-beta-hydroxyhyoscyamine epoxidase.	NF	NF	NF	NF
6164	1.14.11.15	Gibberellin 3-beta-dioxygenase.	NF	NF	NF	NF
6165	1.14.11.19	Leucocyanidin oxygenase.	NF	NF	NF	NF
6166	1.14.11.21	Clavaminate synthase.	NF	NF	NF	NF
6167	1.14.11.22	Flavone synthase.	NF	NF	NF	NF
6168	1.14.11.23	Flavonol synthase.	NF	NF	NF	NF
6169	1.14.11.26	Deacetoxycephalosporin-C hydroxylase.	NF	NF	NF	NF
6170	1.14.11.9	Flavanone 3-dioxygenase.	NF	NF	NF	NF
6171	1.14.12.20	Pheophorbide a oxygenase.	NF	NF	NF	NF
6172	1.14.13.-	With NADH or NADPH as one donor, and incorporat	NF	NF	NF	NF
6173	1.14.13.105	Monocyclic monoterpene ketone monooxygenase.	NF	NF	NF	NF
6174	1.14.13.107	Limonene 1,2-monooxygenase.	NF	NF	NF	NF
6175	1.14.13.108	Abietadiene hydroxylase.	NF	NF	NF	NF
6176	1.14.13.109	Abietadienol hydroxylase.	NF	NF	NF	NF
6177	1.14.13.11	Trans-cinnamate 4-monooxygenase.	NF	NF	NF	NF
6178	1.14.13.110	Geranylgeraniol 18-hydroxylase.	NF	NF	NF	NF
6179	1.14.13.112	3-epi-6-deoxocathasterone 23-monooxygenase.	NF	NF	NF	NF
6180	1.14.13.14	Trans-cinnamate 2-monooxygenase.	NF	NF	NF	NF
6181	1.14.13.21	Flavonoid 3'-monooxygenase.	NF	NF	NF	NF
6182	1.14.13.36	5-O-(4-coumaroyl)-D-quinate 3'-monooxygenase.	NF	NF	NF	NF
6183	1.14.13.37	Methyltetrahydroprotoberberine 14-monooxygena	NF	NF	NF	NF
6184	1.14.13.38	Anhydrotetracycline monooxygenase.	NF	NF	NF	NF
6185	1.14.13.39	Nitric-oxide synthase.	NF	NF	NF	NF
6186	1.14.13.41	Tyrosine N-monooxygenase.	NF	NF	NF	NF
6187	1.14.13.47	(S)-limonene 3-monooxygenase.	NF	NF	NF	NF
6188	1.14.13.49	(S)-limonene 7-monooxygenase.	NF	NF	NF	NF
6189	1.14.13.52	Isoflavone 3'-hydroxylase.	NF	NF	NF	NF
6190	1.14.13.55	Protopine 6-monooxygenase.	NF	NF	NF	NF
6191	1.14.13.56	Dihydrosanguinarine 10-monooxygenase.	NF	NF	NF	NF
6192	1.14.13.57	Dihydrochelirubine 12-monooxygenase.	NF	NF	NF	NF
6193	1.14.13.68	4-hydroxyphenylacetaldehyde oxime monooxygena	NF	NF	NF	NF
6194	1.14.13.70	Sterol 14-demethylase.	NF	NF	NF	NF
6195	1.14.13.71	N-methylcoclaurine 3'-monooxygenase.	NF	NF	NF	NF
6196	1.14.13.74	7-deoxyloganin 7-hydroxylase.	NF	NF	NF	NF
6197	1.14.13.75	Vinorine hydroxylase.	NF	NF	NF	NF
6198	1.14.13.77	Taxane 13-alpha-hydroxylase.	NF	NF	NF	NF
6199	1.14.13.78	Ent-kaurene oxidase.	NF	NF	NF	NF
6200	1.14.13.79	Ent-kaurenoic acid oxidase.	NF	NF	NF	NF
6201	1.14.13.81	Magnesium-protoporphyrin IX monomethyl ester (c	NF	NF	NF	NF
6202	1.14.13.86	2-hydroxyisoflavanone synthase.	NF	NF	NF	NF
6203	1.14.13.88	Flavonoid 3',5'-hydroxylase.	NF	NF	NF	NF
6204	1.14.13.89	Isoflavone 2'-hydroxylase.	NF	NF	NF	NF
6205	1.14.13.90	Zeaxanthin epoxidase.	NF	NF	NF	NF
6206	1.14.13.91	Deoxysarpagine hydroxylase.	NF	NF	NF	NF
6207	1.14.14.-	With reduced flavin or flavoprotein as one donor, a	NF	NF	NF	NF
6208	1.14.17.4	Aminocyclopropanecarboxylate oxidase.	NF	NF	NF	NF
6209	1.14.18.-	With another compound as one donor, and incorpo	NF	NF	NF	NF

6210	1.14.18.1	Monophenol monooxygenase.	NF	NF	NF	NF
6211	1.14.20.1	Deacetoxycephalosporin-C synthase.	NF	NF	NF	NF
6212	1.14.21.-	With NADH or NADPH as one donor, and the other	NF	NF	NF	NF
6213	1.14.21.1	(S)-stylopine synthase.	NF	NF	NF	NF
6214	1.14.21.2	(S)-cheilanthifoline synthase.	NF	NF	NF	NF
6215	1.14.21.4	Salutaridine synthase.	NF	NF	NF	NF
6216	1.14.21.5	(S)-canadine synthase.	NF	NF	NF	NF
6217	1.14.21.6	Lathosterol oxidase.	NF	NF	NF	NF
6218	1.14.99.-	Miscellaneous (requires further characterization).	NF	NF	NF	NF
6219	1.14.99.22	Ecdysone 20-monooxygenase.	NF	NF	NF	NF
6220	1.14.99.30	Carotene 7,8-desaturase.	NF	NF	NF	NF
6221	1.14.99.37	Taxadiene 5-alpha-hydroxylase.	NF	NF	NF	NF
6222	1.14.99.7	Squalene monooxygenase.	NF	NF	NF	NF
6223	1.17.1.2	4-hydroxy-3-methylbut-2-enyl diphosphate reducta	NF	NF	NF	FOUND
6224	1.17.1.3	Leucoanthocyanidin reductase.	NF	NF	NF	NF
6225	1.17.3.2	Xanthine oxidase.	NF	NF	NF	NF
6226	1.17.7.1	(E)-4-hydroxy-3-methylbut-2-enyl-diphosphate synt	NF	NF	NF	FOUND
6227	1.18.-.-	Acting on iron-sulfur proteins as donors.	NF	NF	NF	NF
6228	1.2.-.-	Acting on the aldehyde or oxo group of donors.	FOUND	FOUND	FOUND	FOUND
6229	1.2.1.-	With NAD(+) or NADP(+) as acceptor.	FOUND	FOUND	FOUND	FOUND
6230	1.2.1.11	Aspartate-semialdehyde dehydrogenase.	FOUND	FOUND	FOUND	FOUND
6231	1.2.1.12	Glyceraldehyde-3-phosphate dehydrogenase (phos	FOUND	FOUND	FOUND	FOUND
6232	1.2.1.3	Aldehyde dehydrogenase (NAD(+)).	NF	NF	FOUND	FOUND
6233	1.2.1.31	L-aminoadipate-semialdehyde dehydrogenase.	NF	NF	NF	NF
6234	1.2.1.38	N-acetyl-gamma-glutamyl-phosphate reductase.	NF	NF	FOUND	FOUND
6235	1.2.1.44	Cinnamoyl-CoA reductase.	NF	NF	NF	NF
6236	1.2.1.59	Glyceraldehyde-3-phosphate dehydrogenase (NAD(	NF	NF	NF	NF
6237	1.2.1.70	Glutamyl-tRNA reductase.	NF	NF	NF	NF
6238	1.2.1.74	Abietadienal dehydrogenase.	NF	NF	NF	NF
6239	1.2.3.14	Abscisic-aldehyde oxidase.	NF	NF	NF	NF
6240	1.2.4.1	Pyruvate dehydrogenase (acetyl-transferring).	FOUND	FOUND	FOUND	FOUND
6241	1.2.4.2	Oxoglutarate dehydrogenase (succinyl-transferring)	NF	NF	NF	NF
6242	1.2.4.4	3-methyl-2-oxobutanoate dehydrogenase (2-methy	NF	NF	NF	FOUND
6243	1.21.3.1	Isopenicillin-N synthase.	NF	NF	NF	NF
6244	1.21.3.2	Columbamine oxidase.	NF	NF	NF	NF
6245	1.21.3.3	Reticuline oxidase.	NF	NF	NF	NF
6246	1.3.1.12	Prephenate dehydrogenase.	NF	NF	NF	NF
6247	1.3.1.13	Prephenate dehydrogenase (NADP(+)).	NF	NF	FOUND	FOUND
6248	1.3.1.21	7-dehydrocholesterol reductase.	NF	NF	NF	NF
6249	1.3.1.26	Dihydrodipicolinate reductase.	FOUND	FOUND	FOUND	FOUND
6250	1.3.1.28	2,3-dihydro-2,3-dihydroxybenzoate dehydrogenase	NF	NF	NF	NF
6251	1.3.1.33	Protochlorophyllide reductase.	NF	NF	NF	NF
6252	1.3.1.36	Geissoschizine dehydrogenase.	NF	NF	NF	NF
6253	1.3.1.45	2'-hydroxyisoflavone reductase.	NF	NF	NF	NF
6254	1.3.1.70	Delta(14)-sterol reductase.	NF	NF	NF	NF
6255	1.3.1.72	Delta(24)-sterol reductase.	NF	NF	NF	NF
6256	1.3.1.73	1,2-dihydrovomilenine reductase.	NF	NF	NF	NF
6257	1.3.1.75	Divinyl chlorophyllide a 8-vinyl-reductase.	NF	NF	NF	NF
6258	1.3.1.76	Precorrin-2 dehydrogenase.	NF	NF	NF	NF
6259	1.3.1.77	Anthocyanidin reductase.	NF	NF	NF	NF
6260	1.3.1.80	Red chlorophyll catabolite reductase.	NF	NF	NF	NF
6261	1.3.1.82	(-)-isopiperitenone reductase.	NF	NF	NF	NF
6262	1.3.1.83	Geranylgeranyl diphosphate reductase.	NF	NF	NF	NF
6263	1.3.2.3	L-galactonolactone dehydrogenase.	NF	NF	NF	NF
6264	1.3.3.-	With oxygen as acceptor.	FOUND	FOUND	FOUND	FOUND

6265	1.3.3.12	L-galactonolactone oxidase.	NF	NF	NF	NF
6266	1.3.3.3	Coproporphyrinogen oxidase.	FOUND	FOUND	FOUND	FOUND
6267	1.3.3.4	Protoporphyrinogen oxidase.	NF	NF	NF	NF
6268	1.3.3.8	Tetrahydroberberine oxidase.	NF	NF	NF	NF
6269	1.3.3.9	Secologanin synthase.	NF	NF	NF	NF
6270	1.3.5.1	Succinate dehydrogenase (ubiquinone).	NF	NF	NF	NF
6271	1.3.99.-	With other acceptors.	NF	NF	FOUND	FOUND
6272	1.3.99.1	Succinate dehydrogenase.	NF	NF	NF	NF
6273	1.3.99.12	2-methylacyl-CoA dehydrogenase.	NF	NF	NF	NF
6274	1.3.99.2	Butyryl-CoA dehydrogenase.	NF	NF	NF	NF
6275	1.3.99.22	Coproporphyrinogen dehydrogenase.	NF	NF	NF	NF
6276	1.3.99.3	Acyl-CoA dehydrogenase.	NF	NF	FOUND	FOUND
6277	1.4.1.13	Glutamate synthase (NADPH).	NF	NF	NF	FOUND
6278	1.4.1.14	Glutamate synthase (NADH).	NF	NF	NF	FOUND
6279	1.4.1.18	Lysine 6-dehydrogenase.	NF	NF	NF	NF
6280	1.4.1.20	Phenylalanine dehydrogenase.	NF	NF	NF	NF
6281	1.4.1.9	Leucine dehydrogenase.	NF	NF	NF	NF
6282	1.4.3.21	Primary-amine oxidase.	NF	NF	FOUND	FOUND
6283	1.4.3.4	Monoamine oxidase.	NF	NF	NF	NF
6284	1.4.99.4	Aralkylamine dehydrogenase.	NF	NF	NF	NF
6285	1.5.-.-	Acting on the CH-NH group of donors.	FOUND	FOUND	FOUND	FOUND
6286	1.5.1.10	Saccharopine dehydrogenase (NADP(+), L-glutamat	NF	NF	NF	NF
6287	1.5.1.2	Pyrroline-5-carboxylate reductase.	NF	NF	FOUND	FOUND
6288	1.5.1.27	1,2-dehydroreticulinium reductase (NADPH).	NF	NF	NF	NF
6289	1.5.1.32	Vomilenine reductase.	NF	NF	NF	NF
6290	1.5.1.7	Saccharopine dehydrogenase (NAD(+), L-lysine-form	NF	NF	FOUND	NF
6291	1.5.1.8	Saccharopine dehydrogenase (NADP(+), L-lysine-for	NF	NF	NF	NF
6292	1.5.1.9	Saccharopine dehydrogenase (NAD(+), L-glutamate-	NF	NF	NF	NF
6293	1.5.3.12	Dihydrobenzophenanthridine oxidase.	NF	NF	NF	NF
6294	1.5.99.8	Proline dehydrogenase.	NF	NF	NF	NF
6295	1.8.1.4	Dihydrolipoyl dehydrogenase.	FOUND	FOUND	FOUND	FOUND
6296	2.-.-.-	Transferases.	FOUND	FOUND	FOUND	FOUND
6297	2.1.1.-	Methyltransferases.	FOUND	FOUND	FOUND	FOUND
6298	2.1.1.10	Homocysteine S-methyltransferase.	NF	NF	NF	NF
6299	2.1.1.101	Macrocin O-methyltransferase.	NF	NF	NF	NF
6300	2.1.1.102	Demethylmacrocin O-methyltransferase.	NF	NF	NF	NF
6301	2.1.1.104	Caffeoyl-CoA O-methyltransferase.	NF	NF	NF	NF
6302	2.1.1.107	Uroporphyrinogen-III C-methyltransferase.	NF	NF	FOUND	FOUND
6303	2.1.1.11	Magnesium protoporphyrin IX methyltransferase.	NF	NF	NF	NF
6304	2.1.1.111	Anthranilate N-methyltransferase.	NF	NF	NF	NF
6305	2.1.1.114	Hexaprenyldihydroxybenzoate methyltransferase.	NF	NF	NF	NF
6306	2.1.1.115	(RS)-1-benzyl-1,2,3,4-tetrahydroisoquinoline N-met	NF	NF	NF	NF
6307	2.1.1.116	3'-hydroxy-N-methyl-(S)-coclaurine 4'-O-methyltran	NF	NF	NF	NF
6308	2.1.1.117	(S)-scoulerine 9-O-methyltransferase.	NF	NF	NF	NF
6309	2.1.1.119	10-hydroxydihydrosanguinarine 10-O-methyltransfe	NF	NF	NF	NF
6310	2.1.1.120	12-hydroxydihydrochelirubine 12-O-methyltransfer	NF	NF	NF	NF
6311	2.1.1.122	(S)-tetrahydroprotoberberine N-methyltransferase.	NF	NF	NF	NF
6312	2.1.1.128	(RS)-norcoclaurine 6-O-methyltransferase.	NF	NF	NF	NF
6313	2.1.1.13	Methionine synthase.	NF	NF	FOUND	FOUND
6314	2.1.1.14	5-methyltetrahydropteroyltriglutamate--homocyste	FOUND	FOUND	FOUND	NF
6315	2.1.1.140	(S)-coclaurine-N-methyltransferase.	NF	NF	NF	NF
6316	2.1.1.143	24-methylenesterol C-methyltransferase.	NF	NF	NF	NF
6317	2.1.1.158	7-methylxanthosine synthase.	NF	NF	NF	NF
6318	2.1.1.160	Caffeine synthase.	NF	NF	NF	NF
6319	2.1.1.38	O-demethylpuromycin O-methyltransferase.	NF	NF	NF	NF

6320	2.1.1.41	Sterol 24-C-methyltransferase.	NF	NF	NF	NF
6321	2.1.1.46	Isoflavone 4'-O-methyltransferase.	NF	NF	NF	NF
6322	2.1.1.53	Putrescine N-methyltransferase.	NF	NF	NF	NF
6323	2.1.1.64	3-demethylubiquinone-9 3-O-methyltransferase.	NF	NF	NF	NF
6324	2.1.1.68	Caffeate O-methyltransferase.	NF	NF	NF	NF
6325	2.1.1.95	Tocopherol O-methyltransferase.	NF	NF	NF	NF
6326	2.1.2.1	Glycine hydroxymethyltransferase.	NF	NF	FOUND	FOUND
6327	2.1.2.11	3-methyl-2-oxobutanoate hydroxymethyltransferas	NF	NF	NF	NF
6328	2.1.2.2	Phosphoribosylglycinamide formyltransferase.	NF	FOUND	FOUND	FOUND
6329	2.1.2.3	Phosphoribosylaminoimidazolecarboxamide formyl	NF	NF	NF	NF
6330	2.1.3.-	Carboxyl- and carbamoyltransferases.	FOUND	FOUND	FOUND	FOUND
6331	2.1.3.3	Ornithine carbamoyltransferase.	FOUND	FOUND	FOUND	FOUND
6332	2.1.4.2	Scyllo-inosamine-4-phosphate amidinotransferase.	NF	NF	NF	NF
6333	2.2.1.1	Transketolase.	FOUND	FOUND	FOUND	FOUND
6334	2.2.1.2	Transaldolase.	FOUND	NF	FOUND	FOUND
6335	2.2.1.6	Acetolactate synthase.	NF	NF	FOUND	FOUND
6336	2.2.1.7	1-deoxy-D-xylulose-5-phosphate synthase.	NF	NF	FOUND	FOUND
6337	2.2.1.9	2-succinyl-5-enolpyruvyl-6-hydroxy-3-cyclohexene-1	NF	NF	NF	NF
6338	2.3.-.-	Acyltransferases.	FOUND	FOUND	FOUND	FOUND
6339	2.3.1.-	Transferring groups other than amino-acyl groups.	FOUND	FOUND	FOUND	FOUND
6340	2.3.1.1	Amino-acid N-acetyltransferase.	NF	NF	FOUND	FOUND
6341	2.3.1.12	Dihydrolipoyllysine-residue acetyltransferase.	FOUND	FOUND	FOUND	FOUND
6342	2.3.1.133	Shikimate O-hydroxycinnamoyltransferase.	NF	NF	NF	NF
6343	2.3.1.146	Pinosylvin synthase.	NF	NF	NF	NF
6344	2.3.1.150	Salutaridinol 7-O-acetyltransferase.	NF	NF	NF	NF
6345	2.3.1.159	Acridone synthase.	NF	NF	NF	NF
6346	2.3.1.16	Acetyl-CoA C-acyltransferase.	NF	NF	NF	NF
6347	2.3.1.160	Vinorine synthase.	NF	NF	NF	NF
6348	2.3.1.164	Isopenicillin-N N-acyltransferase.	NF	NF	NF	NF
6349	2.3.1.166	2-alpha-hydroxytaxane 2-O-benzoyltransferase.	NF	NF	NF	NF
6350	2.3.1.167	10-deacetylbaccatin III 10-O-acetyltransferase.	NF	NF	NF	NF
6351	2.3.1.168	Dihydrolipoyllysine-residue (2-methylpropanoyl)tra	NF	NF	NF	NF
6352	2.3.1.170	6'-deoxychalcone synthase.	NF	NF	NF	NF
6353	2.3.1.175	Deacetylcephalosporin-C acetyltransferase.	NF	NF	NF	NF
6354	2.3.1.35	Glutamate N-acetyltransferase.	NF	NF	NF	NF
6355	2.3.1.46	Homoserine O-succinyltransferase.	NF	NF	FOUND	FOUND
6356	2.3.1.5	Arylamine N-acetyltransferase.	NF	NF	NF	NF
6357	2.3.1.61	Dihydrolipoyllysine-residue succinyltransferase.	FOUND	NF	FOUND	NF
6358	2.3.1.74	Naringenin-chalcone synthase.	NF	NF	NF	NF
6359	2.3.1.9	Acetyl-CoA C-acetyltransferase.	FOUND	FOUND	NF	NF
6360	2.3.1.94	6-deoxyerythronolide-B synthase.	NF	NF	NF	NF
6361	2.3.1.95	Trihydroxystilbene synthase.	NF	NF	NF	NF
6362	2.3.2.-	Aminoacyltransferases.	NF	NF	NF	NF
6363	2.3.3.-	Acyl groups converted into alkyl on transfer.	FOUND	FOUND	FOUND	FOUND
6364	2.3.3.1	Citrate (Si)-synthase.	NF	NF	FOUND	FOUND
6365	2.3.3.10	Hydroxymethylglutaryl-CoA synthase.	FOUND	FOUND	FOUND	NF
6366	2.3.3.13	2-isopropylmalate synthase.	NF	NF	FOUND	FOUND
6367	2.3.3.8	ATP citrate synthase.	NF	NF	NF	NF
6368	2.4.1.-	Hexosyltransferases.	FOUND	FOUND	FOUND	FOUND
6369	2.4.1.114	2-coumarate O-beta-glucosyltransferase.	NF	NF	NF	NF
6370	2.4.1.115	Anthocyanidin 3-O-glucosyltransferase.	NF	NF	NF	NF
6371	2.4.1.159	Flavonol-3-O-glucoside L-rhamnosyltransferase.	NF	NF	NF	NF
6372	2.4.1.178	Hydroxymandelonitrile glucosyltransferase.	NF	NF	NF	NF
6373	2.4.1.195	N-hydroxythioamide S-beta-glucosyltransferase.	NF	NF	NF	NF
6374	2.4.1.219	Vomilenine glucosyltransferase.	NF	NF	NF	NF

6375	2.4.1.63	Linamarin synthase.	NF	NF	NF	NF
6376	2.4.1.85	Cyanohydrin beta-glucosyltransferase.	NF	NF	NF	NF
6377	2.4.1.91	Flavonol 3-O-glucosyltransferase.	NF	NF	NF	NF
6378	2.4.2.-	Pentosyltransferases.	FOUND	FOUND	FOUND	FOUND
6379	2.4.2.1	Purine-nucleoside phosphorylase.	FOUND	FOUND	FOUND	FOUND
6380	2.4.2.14	Amidophosphoribosyltransferase.	NF	FOUND	FOUND	FOUND
6381	2.4.2.17	ATP phosphoribosyltransferase.	NF	NF	FOUND	FOUND
6382	2.4.2.18	Anthranilate phosphoribosyltransferase.	NF	NF	FOUND	FOUND
6383	2.4.2.22	Xanthine phosphoribosyltransferase.	NF	NF	NF	FOUND
6384	2.4.2.27	dTDP-dihydrostreptose--streptidine-6-phosphate di	NF	NF	NF	NF
6385	2.4.2.8	Hypoxanthine phosphoribosyltransferase.	FOUND	FOUND	FOUND	FOUND
6386	2.5.1.-	Transferring alkyl or aryl groups, other than methyl	FOUND	FOUND	FOUND	FOUND
6387	2.5.1.1	Dimethylallyltranstransferase.	NF	NF	FOUND	FOUND
6388	2.5.1.10	(2E,6E)-farnesyl diphosphate synthase.	FOUND	NF	NF	NF
6389	2.5.1.19	3-phosphoshikimate 1-carboxyvinyltransferase.	NF	NF	FOUND	FOUND
6390	2.5.1.21	Squalene synthase.	NF	NF	NF	NF
6391	2.5.1.24	Discadenine synthase.	NF	NF	NF	NF
6392	2.5.1.29	Farnesyltranstransferase.	NF	NF	NF	NF
6393	2.5.1.30	Heptaprenyl diphosphate synthase.	NF	NF	NF	NF
6394	2.5.1.31	Di-trans,poly-cis-undecaprenyl-diphosphate synthas	FOUND	FOUND	FOUND	FOUND
6395	2.5.1.32	Phytoene synthase.	NF	NF	NF	NF
6396	2.5.1.39	4-hydroxybenzoate polyprenyltransferase.	NF	NF	NF	NF
6397	2.5.1.44	Homospermidine synthase.	NF	NF	NF	NF
6398	2.5.1.48	Cystathionine gamma-synthase.	NF	NF	FOUND	NF
6399	2.5.1.54	3-deoxy-7-phosphoheptulonate synthase.	NF	NF	FOUND	FOUND
6400	2.5.1.6	Methionine adenosyltransferase.	FOUND	FOUND	FOUND	FOUND
6401	2.5.1.61	Hydroxymethylbilane synthase.	NF	NF	FOUND	FOUND
6402	2.5.1.62	Chlorophyll synthase.	NF	NF	NF	NF
6403	2.5.1.66	N(2)-(2-carboxyethyl)arginine synthase.	NF	NF	NF	NF
6404	2.5.1.74	1,4-dihydroxy-2-naphthoate polyprenyltransferase.	NF	NF	NF	NF
6405	2.5.1.75	tRNA dimethylallyltransferase.	FOUND	NF	NF	NF
6406	2.5.1.82	Hexaprenyl diphosphate synthase (geranylgeranyl-d	NF	NF	NF	NF
6407	2.5.1.83	Hexaprenyl-diphosphate synthase ((2E,6E)-farnesyl-	NF	NF	NF	NF
6408	2.5.1.84	All-trans-nonaprenyl-diphosphate synthase (gerany	NF	NF	NF	NF
6409	2.5.1.85	All-trans-nonaprenyl-diphosphate synthase (gerany	NF	NF	NF	NF
6410	2.6.1.-	Transaminases (aminotransferases).	FOUND	FOUND	FOUND	FOUND
6411	2.6.1.1	Aspartate transaminase.	FOUND	FOUND	FOUND	FOUND
6412	2.6.1.11	Acetylornithine transaminase.	NF	NF	FOUND	FOUND
6413	2.6.1.13	Ornithine aminotransferase.	NF	NF	NF	NF
6414	2.6.1.42	Branched-chain-amino-acid transaminase.	NF	NF	FOUND	FOUND
6415	2.6.1.45	Serine--glyoxylate transaminase.	NF	NF	NF	NF
6416	2.6.1.5	Tyrosine transaminase.	NF	NF	NF	NF
6417	2.6.1.50	Glutamine--scyllo-inositol transaminase.	NF	NF	NF	NF
6418	2.6.1.56	1D-1-guanidino-3-amino-1,3-dideoxy-scyllo-inositol	NF	NF	NF	NF
6419	2.6.1.57	Aromatic-amino-acid transaminase.	NF	NF	NF	NF
6420	2.6.1.58	Phenylalanine(histidine) transaminase.	NF	NF	NF	NF
6421	2.6.1.6	Leucine transaminase.	NF	NF	NF	NF
6422	2.6.1.66	Valine--pyruvate transaminase.	NF	NF	NF	NF
6423	2.6.1.83	LL-diaminopimelate aminotransferase.	NF	NF	NF	NF
6424	2.6.1.9	Histidinol-phosphate transaminase.	NF	NF	FOUND	FOUND
6425	2.7.1.1	Hexokinase.	NF	NF	NF	NF
6426	2.7.1.11	6-phosphofructokinase.	FOUND	FOUND	FOUND	FOUND
6427	2.7.1.12	Gluconokinase.	NF	NF	NF	NF
6428	2.7.1.146	ADP-specific phosphofructokinase.	NF	NF	NF	NF
6429	2.7.1.147	ADP-specific glucokinase.	NF	NF	NF	NF

6430	2.7.1.148	4-(cytidine 5'-diphospho)-2-C-methyl-D-erythritol ki	NF	NF	NF	FOUND
6431	2.7.1.2	Glucokinase.	FOUND	FOUND	FOUND	FOUND
6432	2.7.1.36	Mevalonate kinase.	FOUND	FOUND	FOUND	NF
6433	2.7.1.40	Pyruvate kinase.	FOUND	FOUND	FOUND	FOUND
6434	2.7.1.63	Polyphosphate--glucose phosphotransferase.	NF	NF	NF	NF
6435	2.7.1.65	Scyllo-inosamine 4-kinase.	NF	NF	NF	NF
6436	2.7.1.71	Shikimate kinase.	NF	FOUND	FOUND	FOUND
6437	2.7.2.3	Phosphoglycerate kinase.	FOUND	FOUND	FOUND	FOUND
6438	2.7.2.4	Aspartate kinase.	FOUND	FOUND	FOUND	FOUND
6439	2.7.2.8	Acetylglutamate kinase.	NF	NF	FOUND	FOUND
6440	2.7.4.2	Phosphomevalonate kinase.	FOUND	FOUND	FOUND	NF
6441	2.7.4.3	Adenylate kinase.	FOUND	FOUND	FOUND	FOUND
6442	2.7.4.6	Nucleoside-diphosphate kinase.	NF	NF	NF	FOUND
6443	2.7.6.1	Ribose-phosphate diphosphokinase.	FOUND	FOUND	FOUND	FOUND
6444	2.7.7.13	Mannose-1-phosphate guanylyltransferase.	FOUND	NF	FOUND	FOUND
6445	2.7.7.22	Mannose-1-phosphate guanylyltransferase (GDP).	FOUND	NF	FOUND	FOUND
6446	2.7.7.24	Glucose-1-phosphate thymidylyltransferase.	NF	NF	FOUND	FOUND
6447	2.7.7.60	2-C-methyl-D-erythritol 4-phosphate cytidylyltransf	NF	NF	NF	NF
6448	2.7.7.69	GDP-L-galactose phosphorylase.	NF	NF	NF	NF
6449	2.7.7.9	UTP--glucose-1-phosphate uridylyltransferase.	FOUND	FOUND	FOUND	FOUND
6450	2.8.2.-	Sulfotransferases.	NF	NF	NF	NF
6451	2.8.2.24	Desulfoglucosinolate sulfotransferase.	NF	NF	NF	NF
6452	2.8.3.-	CoA-transferases.	NF	NF	NF	NF
6453	3.1.1.1	Carboxylesterase.	NF	NF	NF	FOUND
6454	3.1.1.10	Tropinesterase.	NF	NF	NF	NF
6455	3.1.1.14	Chlorophyllase.	NF	NF	NF	FOUND
6456	3.1.1.17	Gluconolactonase.	NF	NF	NF	NF
6457	3.1.1.31	6-phosphogluconolactonase.	NF	NF	NF	NF
6458	3.1.1.59	Juvenile-hormone esterase.	NF	NF	NF	NF
6459	3.1.1.78	Polyneuridine-aldehyde esterase.	NF	NF	NF	NF
6460	3.1.1.80	Acetylajmaline esterase.	NF	NF	NF	NF
6461	3.1.1.82	Pheophorbidase.	NF	NF	NF	NF
6462	3.1.2.-	Thiolester hydrolases.	NF	FOUND	NF	NF
6463	3.1.2.23	4-hydroxybenzoyl-CoA thioesterase.	NF	NF	NF	NF
6464	3.1.3.-	Phosphoric monoester hydrolases.	FOUND	FOUND	FOUND	FOUND
6465	3.1.3.11	Fructose-bisphosphatase.	NF	NF	NF	FOUND
6466	3.1.3.15	Histidinol-phosphatase.	NF	NF	FOUND	FOUND
6467	3.1.3.25	Inositol-phosphate phosphatase.	NF	NF	NF	NF
6468	3.1.3.39	Streptomycin-6-phosphatase.	NF	NF	NF	NF
6469	3.1.3.40	Guanidinodeoxy-scyllo-inositol-4-phosphatase.	NF	NF	NF	NF
6470	3.1.3.5	5'-nucleotidase.	FOUND	FOUND	FOUND	FOUND
6471	3.1.7.-	Diphosphoric monoester hydrolases.	NF	NF	NF	NF
6472	3.1.7.3	Monoterpenyl-diphosphatase.	NF	NF	NF	NF
6473	3.1.7.4	Sclareol cyclase.	NF	NF	NF	NF
6474	3.1.7.5	Geranylgeranyl diphosphate diphosphatase.	NF	NF	NF	NF
6475	3.2.1.105	3-alpha-(S)-strictosidine beta-glucosidase.	NF	NF	NF	NF
6476	3.2.1.21	Beta-glucosidase.	NF	NF	NF	NF
6477	3.2.2.25	N-methyl nucleosidase.	NF	NF	NF	NF
6478	3.3.2.1	Isochorismatase.	NF	NF	NF	FOUND
6479	3.3.2.8	Limonene-1,2-epoxide hydrolase.	NF	NF	NF	NF
6480	3.5.1.-	In linear amides.	FOUND	FOUND	FOUND	FOUND
6481	3.5.1.1	Asparaginase.	NF	NF	NF	NF
6482	3.5.1.11	Penicillin amidase.	NF	NF	NF	NF
6483	3.5.1.14	Aminoacylase.	FOUND	FOUND	FOUND	FOUND
6484	3.5.1.16	Acetylornithine deacetylase.	NF	NF	NF	NF

6485	3.5.1.22	Pantothenase.	NF	NF	NF	NF
6486	3.5.1.38	Glutamin-(asparagin-)ase.	NF	NF	NF	NF
6487	3.5.2.6	Beta-lactamase.	NF	NF	NF	FOUND
6488	3.5.3.1	Arginase.	NF	NF	NF	NF
6489	3.5.3.22	Proclavaminate amidinohydrolase.	NF	NF	NF	NF
6490	3.5.3.6	Arginine deiminase.	NF	NF	NF	NF
6491	3.5.4.10	IMP cyclohydrolase.	NF	FOUND	FOUND	FOUND
6492	3.5.4.19	Phosphoribosyl-AMP cyclohydrolase.	NF	NF	FOUND	FOUND
6493	3.5.4.6	AMP deaminase.	NF	NF	NF	NF
6494	3.6.1.31	Phosphoribosyl-ATP diphosphatase.	NF	NF	NF	NF
6495	3.7.1.-	In ketonic substances.	NF	NF	NF	FOUND
6496	4.-.-.-	Lyases.	FOUND	FOUND	FOUND	FOUND
6497	4.1.1.-	Carboxy-lyases.	FOUND	FOUND	FOUND	FOUND
6498	4.1.1.1	Pyruvate decarboxylase.	NF	NF	NF	NF
6499	4.1.1.11	Aspartate 1-decarboxylase.	NF	NF	NF	NF
6500	4.1.1.15	Glutamate decarboxylase.	NF	NF	NF	NF
6501	4.1.1.17	Ornithine decarboxylase.	NF	NF	NF	NF
6502	4.1.1.18	Lysine decarboxylase.	NF	NF	NF	NF
6503	4.1.1.20	Diaminopimelate decarboxylase.	NF	NF	FOUND	FOUND
6504	4.1.1.21	Phosphoribosylaminoimidazole carboxylase.	NF	FOUND	FOUND	FOUND
6505	4.1.1.22	Histidine decarboxylase.	NF	NF	NF	NF
6506	4.1.1.25	Tyrosine decarboxylase.	NF	NF	NF	NF
6507	4.1.1.28	Aromatic-L-amino-acid decarboxylase.	NF	NF	NF	NF
6508	4.1.1.32	Phosphoenolpyruvate carboxykinase (GTP).	NF	NF	NF	NF
6509	4.1.1.33	Diphosphomevalonate decarboxylase.	FOUND	FOUND	FOUND	NF
6510	4.1.1.37	Uroporphyrinogen decarboxylase.	NF	NF	NF	NF
6511	4.1.1.48	Indole-3-glycerol-phosphate synthase.	NF	NF	FOUND	FOUND
6512	4.1.1.49	Phosphoenolpyruvate carboxykinase (ATP).	NF	NF	FOUND	FOUND
6513	4.1.1.80	4-hydroxyphenylpyruvate decarboxylase.	NF	NF	NF	NF
6514	4.1.1.82	Phosphonopyruvate decarboxylase.	NF	NF	NF	NF
6515	4.1.2.13	Fructose-bisphosphate aldolase.	FOUND	FOUND	FOUND	FOUND
6516	4.1.2.41	Vanillin synthase.	NF	NF	NF	NF
6517	4.1.2.5	Threonine aldolase.	NF	NF	FOUND	NF
6518	4.1.2.8	Indole-3-glycerol-phosphate lyase.	NF	NF	NF	NF
6519	4.1.3.-	Oxo-acid-lyases.	FOUND	FOUND	FOUND	FOUND
6520	4.1.3.26	3-hydroxy-3-isohexenylglutaryl-CoA lyase.	NF	NF	NF	NF
6521	4.1.3.27	Anthranilate synthase.	NF	NF	FOUND	FOUND
6522	4.1.3.36	1,4-dihydroxy-2-naphthoyl-CoA synthase.	NF	NF	NF	NF
6523	4.1.3.4	Hydroxymethylglutaryl-CoA lyase.	NF	NF	NF	NF
6524	4.1.3.40	Chorismate lyase.	NF	NF	NF	NF
6525	4.1.3.6	Citrate (pro-3S)-lyase.	NF	NF	NF	NF
6526	4.2.1.-	Hydro-lyases.	FOUND	FOUND	FOUND	FOUND
6527	4.2.1.10	3-dehydroquinate dehydratase.	NF	NF	FOUND	FOUND
6528	4.2.1.101	Trans-feruloyl-CoA hydratase.	NF	NF	NF	NF
6529	4.2.1.105	2-hydroxyisoflavanone dehydratase.	NF	NF	NF	NF
6530	4.2.1.11	Phosphopyruvate hydratase.	FOUND	FOUND	FOUND	FOUND
6531	4.2.1.113	o-succinylbenzoate synthase.	NF	NF	NF	NF
6532	4.2.1.17	Enoyl-CoA hydratase.	NF	NF	NF	NF
6533	4.2.1.19	Imidazoleglycerol-phosphate dehydratase.	NF	NF	FOUND	FOUND
6534	4.2.1.2	Fumarate hydratase.	NF	NF	NF	FOUND
6535	4.2.1.20	Tryptophan synthase.	NF	NF	FOUND	FOUND
6536	4.2.1.24	Porphobilinogen synthase.	NF	NF	FOUND	FOUND
6537	4.2.1.3	Aconitate hydratase.	NF	NF	FOUND	NF
6538	4.2.1.33	3-isopropylmalate dehydratase.	NF	NF	FOUND	FOUND
6539	4.2.1.46	dTDP-glucose 4,6-dehydratase.	NF	NF	FOUND	FOUND

6540	4.2.1.51	Prephenate dehydratase.	NF	NF	FOUND	FOUND
6541	4.2.1.52	Dihydrodipicolinate synthase.	FOUND	FOUND	FOUND	FOUND
6542	4.2.1.57	Isohexenylglutaconyl-CoA hydratase.	NF	NF	NF	NF
6543	4.2.1.75	Uroporphyrinogen-III synthase.	NF	NF	FOUND	FOUND
6544	4.2.1.78	(S)-norcoclaurine synthase.	NF	NF	NF	NF
6545	4.2.1.9	Dihydroxy-acid dehydratase.	NF	NF	FOUND	FOUND
6546	4.2.1.91	Arogenate dehydratase.	NF	NF	NF	NF
6547	4.2.3.-	Acting on phosphates.	NF	NF	FOUND	FOUND
6548	4.2.3.10	(-)-endo-fenchol synthase.	NF	NF	NF	NF
6549	4.2.3.11	Sabinene-hydrate synthase.	NF	NF	NF	NF
6550	4.2.3.13	(+)-delta-cadinene synthase.	NF	NF	NF	NF
6551	4.2.3.14	Pinene synthase.	NF	NF	NF	NF
6552	4.2.3.15	Myrcene synthase.	NF	NF	NF	NF
6553	4.2.3.16	(4S)-limonene synthase.	NF	NF	NF	NF
6554	4.2.3.17	Taxadiene synthase.	NF	NF	NF	NF
6555	4.2.3.18	Abietadiene synthase.	NF	NF	NF	NF
6556	4.2.3.19	Ent-kaurene synthase.	NF	NF	NF	NF
6557	4.2.3.20	(R)-limonene synthase.	NF	NF	NF	NF
6558	4.2.3.21	Vetispiradiene synthase.	NF	NF	NF	NF
6559	4.2.3.22	Germacradienol synthase.	NF	NF	NF	NF
6560	4.2.3.23	Germacrene-A synthase.	NF	NF	NF	NF
6561	4.2.3.26	R-linalool synthase.	NF	NF	NF	NF
6562	4.2.3.27	Isoprene synthase.	NF	NF	NF	NF
6563	4.2.3.28	Ent-cassa-12,15-diene synthase.	NF	NF	NF	NF
6564	4.2.3.29	Ent-sandaracopimaradiene synthase.	NF	NF	NF	NF
6565	4.2.3.30	Ent-pimara-8(14),15-diene synthase.	NF	NF	NF	NF
6566	4.2.3.32	Levopimaradiene synthase.	NF	NF	NF	NF
6567	4.2.3.33	Stemar-13-ene synthase.	NF	NF	NF	NF
6568	4.2.3.34	Stemod-13(17)-ene synthase.	NF	NF	NF	NF
6569	4.2.3.35	Syn-pimara-7,15-diene synthase.	NF	NF	NF	NF
6570	4.2.3.38	Alpha-bisabolene synthase.	NF	NF	NF	NF
6571	4.2.3.4	3-dehydroquinate synthase.	NF	NF	FOUND	FOUND
6572	4.2.3.42	Aphidicolan-16-beta-ol synthase.	NF	NF	NF	NF
6573	4.2.3.43	Fusicocca-2,10(14)-diene synthase.	NF	NF	NF	NF
6574	4.2.3.44	Isopimara-7,15-diene synthase.	NF	NF	NF	NF
6575	4.2.3.5	Chorismate synthase.	NF	NF	FOUND	FOUND
6576	4.2.3.8	Casbene synthase.	NF	NF	NF	NF
6577	4.2.3.9	Aristolochene synthase.	NF	NF	NF	NF
6578	4.2.99.20	2-succinyl-6-hydroxy-2,4-cyclohexadiene-1-carboxy	NF	NF	NF	NF
6579	4.3.1.12	Ornithine cyclodeaminase.	NF	NF	NF	NF
6580	4.3.1.17	L-serine ammonia-lyase.	FOUND	NF	NF	FOUND
6581	4.3.1.19	Threonine ammonia-lyase.	FOUND	FOUND	FOUND	FOUND
6582	4.3.1.24	Phenylalanine ammonia-lyase.	NF	NF	NF	NF
6583	4.3.1.25	Phenylalanine/tyrosine ammonia-lyase.	NF	NF	NF	NF
6584	4.3.2.1	Argininosuccinate lyase.	NF	NF	FOUND	FOUND
6585	4.3.2.2	Adenylosuccinate lyase.	FOUND	FOUND	FOUND	FOUND
6586	4.3.3.2	Strictosidine synthase.	NF	NF	NF	NF
6587	4.3.3.3	Deacetylisoipecoside synthase.	NF	NF	NF	NF
6588	4.4.1.-	Carbon-sulfur lyases.	FOUND	NF	FOUND	FOUND
6589	4.4.1.14	1-aminocyclopropane-1-carboxylate synthase.	NF	NF	NF	NF
6590	4.4.1.8	Cystathionine beta-lyase.	FOUND	NF	FOUND	NF
6591	4.6.1.12	2-C-methyl-D-erythritol 2,4-cyclodiphosphate synth	NF	NF	NF	FOUND
6592	4.99.1.1	Ferrochelatase.	NF	NF	NF	NF
6593	4.99.1.4	Sirohydrochlorin ferrochelatase.	NF	NF	NF	NF
6594	4.99.1.5	Aliphatic aldoxime dehydratase.	NF	NF	NF	NF

6595	5.1.1.17	Isopenicillin-N epimerase.	NF	NF	NF	NF
6596	5.1.1.7	Diaminopimelate epimerase.	FOUND	FOUND	FOUND	FOUND
6597	5.1.3.1	Ribulose-phosphate 3-epimerase.	FOUND	FOUND	FOUND	FOUND
6598	5.1.3.13	dTDP-4-dehydrorhamnose 3,5-epimerase.	NF	NF	FOUND	FOUND
6599	5.1.3.15	Glucose-6-phosphate 1-epimerase.	NF	NF	NF	NF
6600	5.1.3.18	GDP-mannose 3,5-epimerase.	NF	NF	NF	NF
6601	5.1.3.3	Aldose 1-epimerase.	NF	FOUND	NF	FOUND
6602	5.2.1.-	Cis-trans Isomerases.	FOUND	FOUND	FOUND	FOUND
6603	5.3.1.-	Interconverting aldoses and ketoses, and related co	FOUND	FOUND	FOUND	FOUND
6604	5.3.1.1	Triose-phosphate isomerase.	FOUND	FOUND	FOUND	FOUND
6605	5.3.1.16	1-(5-phosphoribosyl)-5- ((5-phosphoribosylamino)m	NF	NF	FOUND	FOUND
6606	5.3.1.24	Phosphoribosylanthranilate isomerase.	NF	NF	FOUND	FOUND
6607	5.3.1.6	Ribose-5-phosphate isomerase.	FOUND	FOUND	FOUND	FOUND
6608	5.3.1.8	Mannose-6-phosphate isomerase.	FOUND	FOUND	FOUND	FOUND
6609	5.3.1.9	Glucose-6-phosphate isomerase.	FOUND	FOUND	FOUND	FOUND
6610	5.3.3.2	Isopentenyl-diphosphate Delta-isomerase.	FOUND	FOUND	FOUND	FOUND
6611	5.3.3.5	Cholestenol Delta-isomerase.	NF	NF	NF	NF
6612	5.3.99.9	Neoxanthin synthase.	NF	NF	NF	NF
6613	5.4.2.1	Phosphoglycerate mutase.	FOUND	FOUND	FOUND	FOUND
6614	5.4.2.2	Phosphoglucomutase.	FOUND	FOUND	FOUND	FOUND
6615	5.4.2.5	Phosphoglucomutase (glucose-cofactor).	NF	NF	NF	NF
6616	5.4.2.8	Phosphomannomutase.	NF	NF	NF	FOUND
6617	5.4.2.9	Phosphoenolpyruvate mutase.	NF	NF	NF	NF
6618	5.4.3.8	Glutamate-1-semialdehyde 2,1-aminomutase.	NF	NF	FOUND	NF
6619	5.4.4.-	Transferring hydroxy groups.	NF	NF	NF	NF
6620	5.4.4.2	Isochorismate synthase.	NF	NF	NF	NF
6621	5.4.99.17	Squalene--hopene cyclase.	NF	NF	NF	NF
6622	5.4.99.18	5-(carboxyamino)imidazole ribonucleotide mutase.	NF	NF	NF	NF
6623	5.4.99.3	2-acetolactate mutase.	NF	NF	NF	NF
6624	5.4.99.5	Chorismate mutase.	NF	NF	FOUND	FOUND
6625	5.4.99.7	Lanosterol synthase.	NF	NF	NF	NF
6626	5.4.99.8	Cycloartenol synthase.	NF	NF	NF	NF
6627	5.5.1.12	Copalyl diphosphate synthase.	NF	NF	NF	NF
6628	5.5.1.13	Ent-copalyl diphosphate synthase.	NF	NF	NF	NF
6629	5.5.1.14	Syn-copalyl-diphosphate synthase.	NF	NF	NF	NF
6630	5.5.1.4	Inositol-3-phosphate synthase.	NF	NF	NF	NF
6631	5.5.1.6	Chalcone isomerase.	NF	NF	NF	NF
6632	5.5.1.8	Bornyl diphosphate synthase.	NF	NF	NF	NF
6633	5.5.1.9	Cycloeucalenol cycloisomerase.	NF	NF	NF	NF
6634	6.1.1.17	Glutamate--tRNA ligase.	FOUND	FOUND	FOUND	FOUND
6635	6.2.1.-	Acid--thiol ligases.	NF	NF	NF	NF
6636	6.2.1.1	Acetate--CoA ligase.	NF	NF	NF	NF
6637	6.2.1.12	4-coumarate--CoA ligase.	NF	NF	NF	NF
6638	6.2.1.26	o-succinylbenzoate--CoA ligase.	NF	NF	NF	NF
6639	6.2.1.32	Anthranilate--CoA ligase.	NF	NF	NF	NF
6640	6.2.1.4	Succinate--CoA ligase (GDP-forming).	NF	NF	NF	NF
6641	6.2.1.5	Succinate--CoA ligase (ADP-forming).	NF	NF	NF	NF
6642	6.3.1.1	Aspartate--ammonia ligase.	FOUND	FOUND	FOUND	FOUND
6643	6.3.2.1	Pantoate--beta-alanine ligase.	NF	NF	NF	NF
6644	6.3.2.26	N-(5-amino-5-carboxypentanoyl)-L-cysteinyl-D-valin	NF	NF	NF	NF
6645	6.3.2.6	Phosphoribosylaminoimidazolesuccinocarboxamide	NF	FOUND	FOUND	FOUND
6646	6.3.3.1	Phosphoribosylformylglycinamidine cyclo-ligase.	NF	FOUND	FOUND	FOUND
6647	6.3.3.4	(Carboxyethyl)arginine beta-lactam-synthase.	NF	NF	NF	NF
6648	6.3.4.-	Other carbon--nitrogen ligases.	FOUND	FOUND	FOUND	FOUND
6649	6.3.4.13	Phosphoribosylamine--glycine ligase.	NF	FOUND	FOUND	FOUND

6650	6.3.4.18	5-(carboxyamino)imidazole ribonucleotide synthase	NF	NF	NF	NF
6651	6.3.4.5	Argininosuccinate synthase.	NF	NF	FOUND	FOUND
6652	6.3.5.-	Carbon--nitrogen ligases with glutamine as amido-N	FOUND	FOUND	FOUND	FOUND
6653	6.3.5.3	Phosphoribosylformylglycinamidine synthase.	NF	FOUND	FOUND	FOUND
6654	6.3.5.4	Asparagine synthase (glutamine-hydrolyzing).	NF	NF	NF	FOUND
6655	6.4.1.2	Acetyl-CoA carboxylase.	NF	NF	FOUND	FOUND
6656	6.4.1.5	Geranoyl-CoA carboxylase.	NF	NF	NF	NF
6657	6.6.1.1	Magnesium chelatase.	NF	NF	FOUND	FOUND
6658	>Microbial metabolism in diverse environments 01120					
6659	1.-.-.-	Oxidoreductases.	FOUND	FOUND	FOUND	FOUND
6660	1.1.-.-	Acting on the CH-OH group of donors.	FOUND	FOUND	FOUND	FOUND
6661	1.1.1.-	With NAD(+) or NADP(+) as acceptor.	FOUND	FOUND	FOUND	FOUND
6662	1.1.1.1	Alcohol dehydrogenase.	FOUND	FOUND	FOUND	FOUND
6663	1.1.1.154	Ureidoglycolate dehydrogenase.	NF	NF	NF	NF
6664	1.1.1.157	3-hydroxybutyryl-CoA dehydrogenase.	NF	NF	NF	NF
6665	1.1.1.174	Cyclohexane-1,2-diol dehydrogenase.	NF	NF	NF	NF
6666	1.1.1.2	Alcohol dehydrogenase (NADP(+)).	NF	NF	FOUND	NF
6667	1.1.1.215	Gluconate 2-dehydrogenase.	NF	NF	NF	NF
6668	1.1.1.244	Methanol dehydrogenase.	NF	NF	NF	NF
6669	1.1.1.245	Cyclohexanol dehydrogenase.	NF	NF	NF	NF
6670	1.1.1.256	Fluoren-9-ol dehydrogenase.	NF	NF	NF	NF
6671	1.1.1.257	4-(hydroxymethyl)benzenesulfonate dehydrogenase	NF	NF	NF	NF
6672	1.1.1.258	6-hydroxyhexanoate dehydrogenase.	NF	NF	NF	NF
6673	1.1.1.259	3-hydroxypimeloyl-CoA dehydrogenase.	NF	NF	NF	NF
6674	1.1.1.26	Glyoxylate reductase.	NF	NF	NF	NF
6675	1.1.1.27	L-lactate dehydrogenase.	FOUND	FOUND	NF	FOUND
6676	1.1.1.272	(R)-2-hydroxyacid dehydrogenase.	NF	NF	NF	NF
6677	1.1.1.284	S-(hydroxymethyl)glutathione dehydrogenase.	NF	NF	NF	NF
6678	1.1.1.29	Glycerate dehydrogenase.	NF	NF	NF	NF
6679	1.1.1.298	3-hydroxypropionate dehydrogenase (NADP(+)).	NF	NF	NF	NF
6680	1.1.1.3	Homoserine dehydrogenase.	NF	NF	FOUND	FOUND
6681	1.1.1.35	3-hydroxyacyl-CoA dehydrogenase.	NF	NF	NF	NF
6682	1.1.1.36	Acetoacetyl-CoA reductase.	NF	NF	NF	NF
6683	1.1.1.37	Malate dehydrogenase.	NF	NF	NF	NF
6684	1.1.1.39	Malate dehydrogenase (decarboxylating).	NF	NF	NF	NF
6685	1.1.1.40	Malate dehydrogenase (oxaloacetate-decarboxylati	NF	NF	FOUND	NF
6686	1.1.1.41	Isocitrate dehydrogenase (NAD(+)).	NF	NF	FOUND	FOUND
6687	1.1.1.42	Isocitrate dehydrogenase (NADP(+)).	NF	NF	FOUND	FOUND
6688	1.1.1.43	Phosphogluconate 2-dehydrogenase.	NF	NF	NF	NF
6689	1.1.1.44	Phosphogluconate dehydrogenase (decarboxylating	NF	NF	NF	NF
6690	1.1.1.47	Glucose 1-dehydrogenase.	NF	NF	NF	NF
6691	1.1.1.49	Glucose-6-phosphate dehydrogenase.	NF	NF	NF	NF
6692	1.1.1.77	Lactaldehyde reductase.	NF	NF	FOUND	NF
6693	1.1.1.79	Glyoxylate reductase (NADP(+)).	NF	NF	NF	NF
6694	1.1.1.82	Malate dehydrogenase (NADP(+)).	NF	NF	NF	NF
6695	1.1.1.90	Aryl-alcohol dehydrogenase.	NF	NF	NF	NF
6696	1.1.1.95	Phosphoglycerate dehydrogenase.	FOUND	NF	FOUND	FOUND
6697	1.1.1.97	3-hydroxybenzyl-alcohol dehydrogenase.	NF	NF	NF	NF
6698	1.1.2.7	Methanol dehydrogenase (cytochrome c).	NF	NF	NF	NF
6699	1.1.2.8	Alcohol dehydrogenase (cytochrome c).	NF	NF	NF	NF
6700	1.1.3.13	Alcohol oxidase.	NF	NF	NF	NF
6701	1.1.3.15	(S)-2-hydroxy-acid oxidase.	NF	NF	NF	FOUND
6702	1.1.3.19	4-hydroxymandelate oxidase.	NF	NF	NF	NF
6703	1.1.3.38	Vanillyl-alcohol oxidase.	NF	NF	NF	NF
6704	1.1.3.4	Glucose oxidase.	NF	NF	NF	NF

6705	1.1.3.5	Hexose oxidase.	NF	NF	NF	NF
6706	1.1.99.14	Glycolate dehydrogenase.	NF	NF	NF	NF
6707	1.1.99.3	Gluconate 2-dehydrogenase (acceptor).	NF	NF	NF	NF
6708	1.1.99.31	(S)-mandelate dehydrogenase.	NF	NF	NF	NF
6709	1.11.1.6	Catalase.	NF	NF	NF	NF
6710	1.11.1.7	Peroxidase.	FOUND	FOUND	FOUND	FOUND
6711	1.12.98.1	Coenzyme F420 hydrogenase.	NF	NF	NF	NF
6712	1.12.98.2	5,10-methenyltetrahydromethanopterin hydrogena	NF	NF	NF	NF
6713	1.12.99.6	Hydrogenase (acceptor).	NF	NF	NF	NF
6714	1.13.-.-	Acting on single donors with incorporation of mole	NF	NF	FOUND	FOUND
6715	1.13.11.-	With incorporation of two atoms of oxygen.	NF	NF	NF	NF
6716	1.13.11.1	Catechol 1,2-dioxygenase.	NF	NF	NF	NF
6717	1.13.11.14	2,3-dihydroxybenzoate 3,4-dioxygenase.	NF	NF	NF	NF
6718	1.13.11.15	3,4-dihydroxyphenylacetate 2,3-dioxygenase.	NF	NF	NF	NF
6719	1.13.11.16	3-carboxyethylcatechol 2,3-dioxygenase.	NF	NF	NF	NF
6720	1.13.11.2	Catechol 2,3-dioxygenase.	NF	NF	NF	NF
6721	1.13.11.3	Protocatechuate 3,4-dioxygenase.	NF	NF	NF	NF
6722	1.13.11.37	Hydroxyquinol 1,2-dioxygenase.	NF	NF	NF	NF
6723	1.13.11.38	1-hydroxy-2-naphthoate 1,2-dioxygenase.	NF	NF	NF	NF
6724	1.13.11.39	Biphenyl-2,3-diol 1,2-dioxygenase.	NF	NF	NF	NF
6725	1.13.11.4	Gentisate 1,2-dioxygenase.	NF	NF	NF	NF
6726	1.13.11.41	2,4'-dihydroxyacetophenone dioxygenase.	NF	NF	NF	NF
6727	1.13.11.5	Homogentisate 1,2-dioxygenase.	NF	NF	NF	NF
6728	1.13.11.56	1,2-dihydroxynaphthalene dioxygenase.	NF	NF	NF	NF
6729	1.13.11.8	Protocatechuate 4,5-dioxygenase.	NF	NF	NF	NF
6730	1.13.12.-	With incorporation of one atom of oxygen.	NF	NF	FOUND	NF
6731	1.14.-.-	Acting on paired donors, with incorporation or redu	NF	NF	NF	NF
6732	1.14.11.-	With 2-oxoglutarate as one donor, and incorporatio	NF	NF	NF	NF
6733	1.14.12.-	With NADH or NADPH as one donor, and incorporat	NF	NF	NF	NF
6734	1.14.12.1	Anthranilate 1,2-dioxygenase (deaminating, decarb	NF	NF	NF	NF
6735	1.14.12.10	Benzoate 1,2-dioxygenase.	NF	NF	NF	NF
6736	1.14.12.11	Toluene dioxygenase.	NF	NF	NF	NF
6737	1.14.12.12	Naphthalene 1,2-dioxygenase.	NF	NF	NF	NF
6738	1.14.12.13	2-chlorobenzoate 1,2-dioxygenase.	NF	NF	NF	NF
6739	1.14.12.14	2-aminobenzenesulfonate 2,3-dioxygenase.	NF	NF	NF	NF
6740	1.14.12.15	Terephthalate 1,2-dioxygenase.	NF	NF	NF	NF
6741	1.14.12.18	Biphenyl 2,3-dioxygenase.	NF	NF	NF	NF
6742	1.14.12.19	3-phenylpropanoate dioxygenase.	NF	NF	NF	NF
6743	1.14.12.3	Benzene 1,2-dioxygenase.	NF	NF	NF	NF
6744	1.14.12.7	Phthalate 4,5-dioxygenase.	NF	NF	NF	NF
6745	1.14.12.8	4-sulfobenzoate 3,4-dioxygenase.	NF	NF	NF	NF
6746	1.14.12.9	4-chlorophenylacetate 3,4-dioxygenase.	NF	NF	NF	NF
6747	1.14.13.-	With NADH or NADPH as one donor, and incorporat	NF	NF	NF	NF
6748	1.14.13.1	Salicylate 1-monooxygenase.	NF	NF	NF	NF
6749	1.14.13.10	2,6-dihydroxypyridine 3-monooxygenase.	NF	NF	NF	NF
6750	1.14.13.12	Benzoate 4-monooxygenase.	NF	NF	NF	NF
6751	1.14.13.14	Trans-cinnamate 2-monooxygenase.	NF	NF	NF	NF
6752	1.14.13.18	4-hydroxyphenylacetate 1-monooxygenase.	NF	NF	NF	NF
6753	1.14.13.2	4-hydroxybenzoate 3-monooxygenase.	NF	NF	NF	NF
6754	1.14.13.20	2,4-dichlorophenol 6-monooxygenase.	NF	NF	NF	NF
6755	1.14.13.22	Cyclohexanone monooxygenase.	NF	NF	NF	NF
6756	1.14.13.23	3-hydroxybenzoate 4-monooxygenase.	NF	NF	NF	NF
6757	1.14.13.24	3-hydroxybenzoate 6-monooxygenase.	NF	NF	NF	NF
6758	1.14.13.25	Methane monooxygenase.	NF	NF	NF	NF
6759	1.14.13.27	4-aminobenzoate 1-monooxygenase.	NF	NF	NF	NF

6760	1.14.13.29	4-nitrophenol 2-monooxygenase.	NF	NF	NF	NF
6761	1.14.13.3	4-hydroxyphenylacetate 3-monooxygenase.	NF	NF	NF	NF
6762	1.14.13.33	4-hydroxybenzoate 3-monooxygenase (NAD(P)H).	NF	NF	NF	NF
6763	1.14.13.4	Melilotate 3-monooxygenase.	NF	NF	NF	NF
6764	1.14.13.40	Anthraniloyl-CoA monooxygenase.	NF	NF	NF	NF
6765	1.14.13.50	Pentachlorophenol monooxygenase.	NF	NF	NF	NF
6766	1.14.13.59	L-lysine 6-monooxygenase (NADPH).	NF	NF	NF	NF
6767	1.14.13.63	3-hydroxyphenylacetate 6-hydroxylase.	NF	NF	NF	NF
6768	1.14.13.64	4-hydroxybenzoate 1-hydroxylase.	NF	NF	NF	NF
6769	1.14.13.69	Alkene monooxygenase.	NF	NF	NF	NF
6770	1.14.13.7	Phenol 2-monooxygenase.	NF	NF	NF	NF
6771	1.14.13.8	Flavin-containing monooxygenase.	NF	NF	NF	NF
6772	1.14.13.82	Vanillate monooxygenase.	NF	NF	NF	NF
6773	1.14.13.84	4-hydroxyacetophenone monooxygenase.	NF	NF	NF	NF
6774	1.14.14.1	Unspecific monooxygenase.	NF	NF	NF	NF
6775	1.14.15.-	With a reduced iron-sulfur protein as one donor, an	NF	NF	NF	NF
6776	1.14.16.6	Mandelate 4-monooxygenase.	NF	NF	NF	NF
6777	1.14.18.-	With another compound as one donor, and incorpo	NF	NF	NF	NF
6778	1.14.99.-	Miscellaneous (requires further characterization).	NF	NF	NF	NF
6779	1.14.99.23	3-hydroxybenzoate 2-monooxygenase.	NF	NF	NF	NF
6780	1.17.1.4	Xanthine dehydrogenase.	NF	NF	NF	FOUND
6781	1.17.5.-	With a quinone or similar compound as acceptor.	NF	NF	NF	NF
6782	1.17.99.1	4-cresol dehydrogenase (hydroxylating).	NF	NF	NF	NF
6783	1.17.99.2	Ethylbenzene hydroxylase.	NF	NF	NF	NF
6784	1.18.6.1	Nitrogenase.	NF	NF	NF	NF
6785	1.2.-.-	Acting on the aldehyde or oxo group of donors.	FOUND	FOUND	FOUND	FOUND
6786	1.2.1.-	With NAD(+) or NADP(+) as acceptor.	FOUND	FOUND	FOUND	FOUND
6787	1.2.1.10	Acetaldehyde dehydrogenase (acetylating).	NF	NF	NF	NF
6788	1.2.1.11	Aspartate-semialdehyde dehydrogenase.	FOUND	FOUND	FOUND	FOUND
6789	1.2.1.12	Glyceraldehyde-3-phosphate dehydrogenase (phos	FOUND	FOUND	FOUND	FOUND
6790	1.2.1.16	Succinate-semialdehyde dehydrogenase (NAD(P)(+)	NF	NF	NF	NF
6791	1.2.1.2	Formate dehydrogenase.	NF	NF	NF	FOUND
6792	1.2.1.21	Glycolaldehyde dehydrogenase.	NF	NF	NF	NF
6793	1.2.1.28	Benzaldehyde dehydrogenase (NAD(+)).	NF	NF	NF	NF
6794	1.2.1.29	Aryl-aldehyde dehydrogenase.	NF	NF	NF	NF
6795	1.2.1.3	Aldehyde dehydrogenase (NAD(+)).	NF	NF	FOUND	FOUND
6796	1.2.1.32	Aminomuconate-semialdehyde dehydrogenase.	NF	NF	NF	NF
6797	1.2.1.39	Phenylacetaldehyde dehydrogenase.	NF	NF	NF	NF
6798	1.2.1.4	Aldehyde dehydrogenase (NADP(+)).	NF	NF	NF	NF
6799	1.2.1.43	Formate dehydrogenase (NADP(+)).	NF	NF	NF	NF
6800	1.2.1.45	4-carboxy-2-hydroxymuconate-6-semialdehyde deh	NF	NF	NF	NF
6801	1.2.1.46	Formaldehyde dehydrogenase.	NF	NF	NF	NF
6802	1.2.1.5	Aldehyde dehydrogenase (NAD(P)(+)).	NF	NF	NF	NF
6803	1.2.1.59	Glyceraldehyde-3-phosphate dehydrogenase (NAD(	NF	NF	NF	NF
6804	1.2.1.60	5-carboxymethyl-2-hydroxymuconic-semialdehyde	NF	NF	NF	NF
6805	1.2.1.61	4-hydroxymuconic-semialdehyde dehydrogenase.	NF	NF	NF	NF
6806	1.2.1.62	4-formylbenzenesulfonate dehydrogenase.	NF	NF	NF	NF
6807	1.2.1.63	6-oxohexanoate dehydrogenase.	NF	NF	NF	NF
6808	1.2.1.64	4-hydroxybenzaldehyde dehydrogenase.	NF	NF	NF	NF
6809	1.2.1.65	Salicylaldehyde dehydrogenase.	NF	NF	NF	NF
6810	1.2.1.67	Vanillin dehydrogenase.	NF	NF	NF	NF
6811	1.2.1.7	Benzaldehyde dehydrogenase (NADP(+)).	NF	NF	NF	NF
6812	1.2.1.75	Malonyl CoA reductase (malonate semialdehyde-fo	NF	NF	NF	NF
6813	1.2.1.76	Succinate-semialdehyde dehydrogenase (acetylatin	NF	NF	NF	NF
6814	1.2.1.9	Glyceraldehyde-3-phosphate dehydrogenase (NADP	FOUND	FOUND	FOUND	FOUND

6815	1.2.3.-	With oxygen as acceptor.	NF	NF	NF	NF
6816	1.2.3.1	Aldehyde oxidase.	NF	NF	NF	NF
6817	1.2.4.1	Pyruvate dehydrogenase (acetyl-transferring).	FOUND	FOUND	FOUND	FOUND
6818	1.2.4.2	Oxoglutarate dehydrogenase (succinyl-transferring)	NF	NF	NF	NF
6819	1.2.7.1	Pyruvate synthase.	NF	NF	NF	NF
6820	1.2.7.3	2-oxoglutarate synthase.	NF	NF	NF	NF
6821	1.2.7.4	Carbon-monoxide dehydrogenase (ferredoxin).	NF	NF	NF	NF
6822	1.2.7.5	Aldehyde ferredoxin oxidoreductase.	NF	NF	NF	NF
6823	1.2.7.6	Glyceraldehyde-3-phosphate dehydrogenase (ferre	NF	NF	NF	NF
6824	1.2.99.2	Carbon-monoxide dehydrogenase (acceptor).	NF	NF	NF	NF
6825	1.2.99.5	Formylmethanofuran dehydrogenase.	NF	NF	NF	NF
6826	1.3.-.-	Acting on the CH-CH group of donors.	FOUND	FOUND	FOUND	FOUND
6827	1.3.1.-	With NAD(+) or NADP(+) as acceptor.	FOUND	FOUND	FOUND	FOUND
6828	1.3.1.11	2-coumarate reductase.	NF	NF	NF	NF
6829	1.3.1.19	Cis-1,2-dihydrobenzene-1,2-diol dehydrogenase.	NF	NF	NF	NF
6830	1.3.1.25	1,6-dihydroxycyclohexa-2,4-diene-1-carboxylate de	NF	NF	NF	NF
6831	1.3.1.26	Dihydrodipicolinate reductase.	FOUND	FOUND	FOUND	FOUND
6832	1.3.1.29	Cis-1,2-dihydro-1,2-dihydroxynaphthalene dehydro	NF	NF	NF	NF
6833	1.3.1.31	2-enoate reductase.	NF	NF	NF	NF
6834	1.3.1.32	Maleylacetate reductase.	NF	NF	NF	NF
6835	1.3.1.49	Cis-3,4-dihydrophenanthrene-3,4-diol dehydrogena	NF	NF	NF	NF
6836	1.3.1.53	(3S,4R)-3,4-dihydroxycyclohexa-1,5-diene-1,4-dicar	NF	NF	NF	NF
6837	1.3.1.56	Cis-2,3-dihydrobiphenyl-2,3-diol dehydrogenase.	NF	NF	NF	NF
6838	1.3.1.57	Phloroglucinol reductase.	NF	NF	NF	NF
6839	1.3.1.58	2,3-dihydroxy-2,3-dihydro-p-cumate dehydrogenas	NF	NF	NF	NF
6840	1.3.1.62	Pimeloyl-CoA dehydrogenase.	NF	NF	NF	NF
6841	1.3.1.63	2,4-dichlorobenzoyl-CoA reductase.	NF	NF	NF	NF
6842	1.3.1.64	Phthalate 4,5-cis-dihydrodiol dehydrogenase.	NF	NF	NF	NF
6843	1.3.1.66	Cis-dihydroethylcatechol dehydrogenase.	NF	NF	NF	NF
6844	1.3.1.67	Cis-1,2-dihydroxy-4-methylcyclohexa-3,5-diene-1-ca	NF	NF	NF	NF
6845	1.3.1.68	1,2-dihydroxy-6-methylcyclohexa-3,5-dienecarboxy	NF	NF	NF	NF
6846	1.3.1.84	Acrylyl-CoA reductase (NADPH).	NF	NF	NF	NF
6847	1.3.99.-	With other acceptors.	NF	NF	FOUND	FOUND
6848	1.3.99.1	Succinate dehydrogenase.	NF	NF	NF	NF
6849	1.3.99.15	Benzoyl-CoA reductase.	NF	NF	NF	NF
6850	1.3.99.20	4-hydroxybenzoyl-CoA reductase.	NF	NF	NF	NF
6851	1.3.99.21	(R)-benzylsuccinyl-CoA dehydrogenase.	NF	NF	NF	NF
6852	1.3.99.7	Glutaryl-CoA dehydrogenase.	NF	NF	NF	NF
6853	1.4.-.-	Acting on the CH-NH(2) group of donors.	NF	FOUND	FOUND	FOUND
6854	1.4.1.13	Glutamate synthase (NADPH).	NF	NF	NF	FOUND
6855	1.4.1.14	Glutamate synthase (NADH).	NF	NF	NF	FOUND
6856	1.4.3.12	Cyclohexylamine oxidase.	NF	NF	NF	NF
6857	1.4.99.3	Amine dehydrogenase.	NF	NF	NF	NF
6858	1.5.1.-	With NAD(+) or NADP(+) as acceptor.	FOUND	FOUND	FOUND	FOUND
6859	1.5.1.20	Methylenetetrahydrofolate reductase (NAD(P)H).	NF	NF	FOUND	FOUND
6860	1.5.1.5	Methylenetetrahydrofolate dehydrogenase (NADP(	FOUND	NF	NF	FOUND
6861	1.5.3.5	(S)-6-hydroxynicotine oxidase.	NF	NF	NF	NF
6862	1.5.3.6	(R)-6-hydroxynicotine oxidase.	NF	NF	NF	NF
6863	1.5.8.1	Dimethylamine dehydrogenase.	NF	NF	NF	NF
6864	1.5.8.2	Trimethylamine dehydrogenase.	NF	NF	NF	NF
6865	1.5.99.11	5,10-methylenetetrahydromethanopterin reductase	NF	NF	NF	NF
6866	1.5.99.4	Nicotine dehydrogenase.	NF	NF	NF	NF
6867	1.5.99.5	Methylglutamate dehydrogenase.	NF	NF	NF	NF
6868	1.5.99.9	Methylenetetrahydromethanopterin dehydrogenas	NF	NF	NF	NF
6869	1.6.-.-	Acting on NADH or NADPH.	FOUND	NF	FOUND	FOUND

6870	1.6.5.-	With a quinone or similar compound as acceptor.	NF	NF	NF	FOUND
6871	1.6.5.6	p-benzoquinone reductase (NADPH).	NF	NF	NF	NF
6872	1.6.5.7	2-hydroxy-1,4-benzoquinone reductase.	NF	NF	NF	NF
6873	1.6.6.9	Trimethylamine-N-oxide reductase.	NF	NF	NF	NF
6874	1.7.1.-	With NAD(+) or NADP(+) as acceptor.	NF	NF	NF	NF
6875	1.7.1.4	Nitrite reductase (NAD(P)H).	NF	NF	NF	NF
6876	1.7.2.1	Nitrite reductase (NO-forming).	NF	NF	NF	NF
6877	1.7.3.3	Factor independent urate hydroxylase.	NF	NF	NF	NF
6878	1.7.7.1	Ferredoxin--nitrite reductase.	NF	NF	NF	NF
6879	1.7.99.4	Nitrate reductase.	NF	NF	NF	NF
6880	1.7.99.6	Nitrous-oxide reductase.	NF	NF	NF	NF
6881	1.7.99.7	Nitric-oxide reductase.	NF	NF	NF	NF
6882	1.8.1.2	Sulfite reductase (NADPH).	NF	NF	NF	NF
6883	1.8.1.4	Dihydrolipoyl dehydrogenase.	FOUND	FOUND	FOUND	FOUND
6884	1.8.2.1	Sulfite dehydrogenase.	NF	NF	NF	NF
6885	1.8.3.1	Sulfite oxidase.	NF	NF	NF	NF
6886	1.8.4.8	Phosphoadenylyl-sulfate reductase (thioredoxin).	NF	NF	NF	NF
6887	1.8.7.1	Sulfite reductase (ferredoxin).	NF	NF	NF	NF
6888	1.8.98.1	CoB--CoM heterodisulfide reductase.	NF	NF	NF	NF
6889	1.8.99.1	Sulfite reductase.	NF	NF	NF	NF
6890	1.8.99.2	Adenylyl-sulfate reductase.	NF	NF	FOUND	NF
6891	1.8.99.3	Hydrogensulfite reductase.	NF	NF	NF	NF
6892	1.97.1.-	Sole sub-subclass for oxidoreductases that do not b	NF	NF	FOUND	NF
6893	1.97.1.2	Pyrogallol hydroxytransferase.	NF	NF	NF	NF
6894	1.97.1.8	Tetrachloroethene reductive dehalogenase.	NF	NF	NF	NF
6895	2.1.-.-	Transferring one-carbon groups.	FOUND	FOUND	FOUND	FOUND
6896	2.1.1.-	Methyltransferases.	FOUND	FOUND	FOUND	FOUND
6897	2.1.1.21	Methylamine--glutamate N-methyltransferase.	NF	NF	NF	NF
6898	2.1.1.86	Tetrahydromethanopterin S-methyltransferase.	NF	NF	NF	NF
6899	2.1.1.90	Methanol--5-hydroxybenzimidazolylcobamide Co-m	NF	NF	NF	NF
6900	2.1.2.1	Glycine hydroxymethyltransferase.	NF	NF	FOUND	FOUND
6901	2.1.3.5	Oxamate carbamoyltransferase.	NF	NF	NF	NF
6902	2.2.1.1	Transketolase.	FOUND	FOUND	FOUND	FOUND
6903	2.2.1.2	Transaldolase.	FOUND	NF	FOUND	FOUND
6904	2.2.1.3	Formaldehyde transketolase.	NF	NF	NF	NF
6905	2.3.1.-	Transferring groups other than amino-acyl groups.	FOUND	FOUND	FOUND	FOUND
6906	2.3.1.101	Formylmethanofuran--tetrahydromethanopterin N-	NF	NF	NF	NF
6907	2.3.1.102	N(6)-hydroxylysine O-acetyltransferase.	NF	NF	NF	NF
6908	2.3.1.117	2,3,4,5-tetrahydropyridine-2,6-dicarboxylate N-succ	NF	NF	NF	NF
6909	2.3.1.12	Dihydrolipoyllysine-residue acetyltransferase.	FOUND	FOUND	FOUND	FOUND
6910	2.3.1.16	Acetyl-CoA C-acyltransferase.	NF	NF	NF	NF
6911	2.3.1.169	CO-methylating acetyl-CoA synthase.	NF	NF	NF	NF
6912	2.3.1.174	3-oxoadipyl-CoA thiolase.	NF	NF	NF	NF
6913	2.3.1.178	Diaminobutyrate acetyltransferase.	NF	NF	NF	NF
6914	2.3.1.30	Serine O-acetyltransferase.	NF	NF	FOUND	FOUND
6915	2.3.1.47	8-amino-7-oxononanoate synthase.	NF	NF	NF	NF
6916	2.3.1.5	Arylamine N-acetyltransferase.	NF	NF	NF	NF
6917	2.3.1.61	Dihydrolipoyllysine-residue succinyltransferase.	FOUND	NF	FOUND	NF
6918	2.3.1.8	Phosphate acetyltransferase.	FOUND	FOUND	FOUND	FOUND
6919	2.3.1.9	Acetyl-CoA C-acetyltransferase.	FOUND	FOUND	NF	NF
6920	2.3.3.1	Citrate (Si)-synthase.	NF	NF	FOUND	FOUND
6921	2.3.3.8	ATP citrate synthase.	NF	NF	NF	NF
6922	2.3.3.9	Malate synthase.	NF	NF	NF	NF
6923	2.5.1.65	O-phosphoserine sulfhydrylase.	NF	NF	NF	NF
6924	2.6.1.-	Transaminases (aminotransferases).	FOUND	FOUND	FOUND	FOUND

6925	2.6.1.1	Aspartate transaminase.	FOUND	FOUND	FOUND	FOUND
6926	2.6.1.17	Succinyldiaminopimelate transaminase.	NF	NF	NF	NF
6927	2.6.1.2	Alanine transaminase.	NF	NF	NF	NF
6928	2.6.1.37	2-aminoethylphosphonate--pyruvate transaminase.	NF	NF	NF	NF
6929	2.6.1.45	Serine--glyoxylate transaminase.	NF	NF	NF	NF
6930	2.6.1.52	Phosphoserine transaminase.	NF	NF	NF	NF
6931	2.6.1.76	Diaminobutyrate--2-oxoglutarate transaminase.	NF	NF	NF	NF
6932	2.7.1.-	Phosphotransferases with an alcohol group as accep	FOUND	FOUND	FOUND	FOUND
6933	2.7.1.1	Hexokinase.	NF	NF	NF	NF
6934	2.7.1.11	6-phosphofructokinase.	FOUND	FOUND	FOUND	FOUND
6935	2.7.1.12	Gluconokinase.	NF	NF	NF	NF
6936	2.7.1.13	Dehydrogluconokinase.	NF	NF	NF	NF
6937	2.7.1.146	ADP-specific phosphofructokinase.	NF	NF	NF	NF
6938	2.7.1.147	ADP-specific glucokinase.	NF	NF	NF	NF
6939	2.7.1.19	Phosphoribulokinase.	NF	NF	NF	NF
6940	2.7.1.2	Glucokinase.	FOUND	FOUND	FOUND	FOUND
6941	2.7.1.25	Adenylyl-sulfate kinase.	NF	NF	NF	NF
6942	2.7.1.29	Glycerone kinase.	FOUND	FOUND	FOUND	FOUND
6943	2.7.1.31	Glycerate kinase.	FOUND	NF	NF	FOUND
6944	2.7.1.39	Homoserine kinase.	NF	NF	FOUND	FOUND
6945	2.7.1.40	Pyruvate kinase.	FOUND	FOUND	FOUND	FOUND
6946	2.7.1.41	Glucose-1-phosphate phosphodismutase.	NF	NF	NF	NF
6947	2.7.1.45	2-dehydro-3-deoxygluconokinase.	FOUND	FOUND	NF	FOUND
6948	2.7.1.63	Polyphosphate--glucose phosphotransferase.	NF	NF	NF	NF
6949	2.7.1.7	Mannokinase.	NF	NF	NF	NF
6950	2.7.2.1	Acetate kinase.	FOUND	FOUND	FOUND	FOUND
6951	2.7.2.2	Carbamate kinase.	FOUND	FOUND	NF	FOUND
6952	2.7.2.3	Phosphoglycerate kinase.	FOUND	FOUND	FOUND	FOUND
6953	2.7.2.4	Aspartate kinase.	FOUND	FOUND	FOUND	FOUND
6954	2.7.6.1	Ribose-phosphate diphosphokinase.	FOUND	FOUND	FOUND	FOUND
6955	2.7.7.4	Sulfate adenylyltransferase.	NF	NF	FOUND	NF
6956	2.7.7.5	Sulfate adenylyltransferase (ADP).	NF	NF	NF	NF
6957	2.7.9.-	Phosphotransferases with paired acceptors.	NF	NF	NF	FOUND
6958	2.7.9.1	Pyruvate, phosphate dikinase.	NF	NF	NF	NF
6959	2.7.9.2	Pyruvate, water dikinase.	NF	NF	NF	FOUND
6960	2.8.2.-	Sulfotransferases.	NF	NF	NF	NF
6961	2.8.2.1	Aryl sulfotransferase.	NF	NF	NF	NF
6962	2.8.2.2	Alcohol sulfotransferase.	NF	NF	NF	NF
6963	2.8.2.3	Amine sulfotransferase.	NF	NF	NF	NF
6964	2.8.2.6	Choline sulfotransferase.	NF	NF	NF	NF
6965	2.8.3.-	CoA-transferases.	NF	NF	NF	NF
6966	2.8.3.1	Propionate CoA-transferase.	NF	NF	NF	NF
6967	2.8.3.12	Glutaconate CoA-transferase.	NF	NF	NF	NF
6968	2.8.3.15	Succinyl-CoA:(R)-benzylsuccinate CoA-transferase.	NF	NF	NF	NF
6969	2.8.3.6	3-oxoadipate CoA-transferase.	NF	NF	NF	NF
6970	2.8.3.8	Acetate CoA-transferase.	NF	NF	NF	NF
6971	2.8.4.1	Coenzyme-B sulfoethylthiotransferase.	NF	NF	NF	NF
6972	3.1.1.-	Carboxylic ester hydrolases.	FOUND	FOUND	FOUND	FOUND
6973	3.1.1.17	Gluconolactonase.	NF	NF	NF	NF
6974	3.1.1.2	Arylesterase.	NF	NF	NF	NF
6975	3.1.1.24	3-oxoadipate enol-lactonase.	NF	NF	NF	NF
6976	3.1.1.31	6-phosphogluconolactonase.	NF	NF	NF	NF
6977	3.1.1.35	Dihydrocoumarin hydrolase.	NF	NF	NF	NF
6978	3.1.1.45	Carboxymethylenebutenolidase.	NF	NF	NF	NF
6979	3.1.1.57	2-pyrone-4,6-dicarboxylate lactonase.	NF	NF	NF	NF

6980	3.1.2.-	Thiolester hydrolases.	NF	FOUND	NF	NF
6981	3.1.2.12	S-formylglutathione hydrolase.	NF	NF	NF	NF
6982	3.1.2.23	4-hydroxybenzoyl-CoA thioesterase.	NF	NF	NF	NF
6983	3.1.3.1	Alkaline phosphatase.	FOUND	FOUND	FOUND	FOUND
6984	3.1.3.10	Glucose-1-phosphatase.	NF	NF	NF	NF
6985	3.1.3.11	Fructose-bisphosphatase.	NF	NF	NF	FOUND
6986	3.1.3.2	Acid phosphatase.	FOUND	FOUND	FOUND	FOUND
6987	3.1.3.3	Phosphoserine phosphatase.	NF	NF	NF	NF
6988	3.1.3.37	Sedoheptulose-bisphosphatase.	NF	NF	NF	NF
6989	3.1.3.41	4-nitrophenylphosphatase.	NF	NF	NF	NF
6990	3.1.3.7	3'(2'),5'-bisphosphate nucleotidase.	NF	NF	NF	NF
6991	3.1.3.71	2-phosphosulfolactate phosphatase.	NF	NF	NF	NF
6992	3.1.8.1	Aryldialkylphosphatase.	NF	NF	NF	FOUND
6993	3.10.1.2	Cyclamate sulfohydrolase.	NF	NF	NF	NF
6994	3.11.1.1	Phosphonoacetaldehyde hydrolase.	NF	NF	NF	FOUND
6995	3.11.1.2	Phosphonoacetate hydrolase.	NF	NF	NF	NF
6996	3.3.2.-	Ether hydrolases.	NF	NF	NF	FOUND
6997	3.3.2.10	Soluble epoxide hydrolase.	NF	NF	NF	NF
6998	3.5.1.-	In linear amides.	FOUND	FOUND	FOUND	FOUND
6999	3.5.1.107	Maleamate amidohydrolase.	NF	NF	NF	NF
7000	3.5.1.18	Succinyl-diaminopimelate desuccinylase.	NF	NF	NF	FOUND
7001	3.5.1.2	Glutaminase.	NF	NF	NF	NF
7002	3.5.1.38	Glutamin-(asparagin-)ase.	NF	NF	NF	NF
7003	3.5.1.4	Amidase.	NF	FOUND	NF	NF
7004	3.5.1.5	Urease.	NF	NF	NF	NF
7005	3.5.1.54	Allophanate hydrolase.	NF	NF	NF	NF
7006	3.5.1.84	Biuret amidohydrolase.	NF	NF	NF	NF
7007	3.5.2.-	In cyclic amides.	NF	NF	FOUND	FOUND
7008	3.5.2.15	Cyanuric acid amidohydrolase.	NF	NF	NF	NF
7009	3.5.2.17	Hydroxyisourate hydrolase.	NF	NF	NF	NF
7010	3.5.2.5	Allantoinase.	NF	NF	NF	NF
7011	3.5.3.19	Ureidoglycolate hydrolase.	NF	NF	NF	NF
7012	3.5.3.4	Allantoicase.	NF	NF	NF	NF
7013	3.5.3.9	Allantoate deiminase.	NF	NF	NF	NF
7014	3.5.4.-	In cyclic amidines.	FOUND	FOUND	FOUND	FOUND
7015	3.5.4.27	Methenyltetrahydromethanopterin cyclohydrolase.	NF	NF	NF	NF
7016	3.5.4.9	Methenyltetrahydrofolate cyclohydrolase.	FOUND	FOUND	FOUND	FOUND
7017	3.5.5.1	Nitrilase.	NF	NF	NF	NF
7018	3.5.5.6	Bromoxynil nitrilase.	NF	NF	NF	NF
7019	3.5.5.7	Aliphatic nitrilase.	NF	NF	NF	NF
7020	3.5.99.-	In other compounds.	FOUND	FOUND	FOUND	FOUND
7021	3.5.99.3	Hydroxydechloroatrazine ethylaminohydrolase.	NF	NF	NF	NF
7022	3.5.99.4	N-isopropylammelide isopropylaminohydrolase.	NF	NF	NF	NF
7023	3.6.1.7	Acylphosphatase.	NF	NF	NF	NF
7024	3.7.1.-	In ketonic substances.	NF	NF	NF	FOUND
7025	3.7.1.11	Cyclohexane-1,2-dione hydrolase.	NF	NF	NF	NF
7026	3.7.1.2	Fumarylacetoacetase.	NF	NF	NF	FOUND
7027	3.7.1.5	Acylpyruvate hydrolase.	NF	NF	NF	NF
7028	3.7.1.8	2,6-dioxo-6-phenylhexa-3-enoate hydrolase.	NF	NF	NF	NF
7029	3.7.1.9	2-hydroxymuconate-semialdehyde hydrolase.	NF	NF	NF	NF
7030	3.8.1.-	In C-halide compounds.	NF	NF	NF	NF
7031	3.8.1.2	(S)-2-haloacid dehalogenase.	NF	NF	NF	NF
7032	3.8.1.3	Haloacetate dehalogenase.	NF	NF	NF	NF
7033	3.8.1.5	Haloalkane dehalogenase.	NF	NF	NF	NF
7034	3.8.1.7	4-chlorobenzoyl-CoA dehalogenase.	NF	NF	NF	NF

7035	3.8.1.8	Atrazine chlorohydrolase.	NF	NF	NF	NF
7036	4.1.1.-	Carboxy-lyases.	FOUND	FOUND	FOUND	FOUND
7037	4.1.1.20	Diaminopimelate decarboxylase.	NF	NF	FOUND	FOUND
7038	4.1.1.31	Phosphoenolpyruvate carboxylase.	FOUND	FOUND	FOUND	NF
7039	4.1.1.32	Phosphoenolpyruvate carboxykinase (GTP).	NF	NF	NF	NF
7040	4.1.1.39	Ribulose-bisphosphate carboxylase.	NF	NF	NF	NF
7041	4.1.1.44	4-carboxymuconolactone decarboxylase.	NF	NF	NF	NF
7042	4.1.1.46	o-pyrocatechuate decarboxylase.	NF	NF	NF	NF
7043	4.1.1.49	Phosphoenolpyruvate carboxykinase (ATP).	NF	NF	FOUND	FOUND
7044	4.1.1.55	4,5-dihydroxyphthalate decarboxylase.	NF	NF	NF	NF
7045	4.1.1.59	Gallate decarboxylase.	NF	NF	NF	NF
7046	4.1.1.61	4-hydroxybenzoate decarboxylase.	NF	NF	NF	NF
7047	4.1.1.62	Gentisate decarboxylase.	NF	NF	NF	NF
7048	4.1.1.63	Protocatechuate decarboxylase.	NF	NF	NF	NF
7049	4.1.1.68	5-oxopent-3-ene-1,2,5-tricarboxylate decarboxylase	NF	NF	NF	NF
7050	4.1.1.69	3,4-dihydroxyphthalate decarboxylase.	NF	NF	NF	NF
7051	4.1.1.7	Benzoylformate decarboxylase.	NF	NF	NF	NF
7052	4.1.1.70	Glutaconyl-CoA decarboxylase.	NF	NF	NF	NF
7053	4.1.1.77	4-oxalocrotonate decarboxylase.	NF	NF	NF	NF
7054	4.1.1.79	Sulfopyruvate decarboxylase.	NF	NF	NF	NF
7055	4.1.1.82	Phosphonopyruvate decarboxylase.	NF	NF	NF	NF
7056	4.1.1.86	Diaminobutyrate decarboxylase.	NF	NF	NF	NF
7057	4.1.2.-	Aldehyde-lyases.	FOUND	FOUND	FOUND	FOUND
7058	4.1.2.13	Fructose-bisphosphate aldolase.	FOUND	FOUND	FOUND	FOUND
7059	4.1.2.14	2-dehydro-3-deoxy-phosphogluconate aldolase.	FOUND	FOUND	NF	FOUND
7060	4.1.2.22	Fructose-6-phosphate phosphoketolase.	NF	NF	NF	NF
7061	4.1.2.32	Trimethylamine-oxide aldolase.	NF	NF	NF	NF
7062	4.1.2.34	4-(2-carboxyphenyl)-2-oxobut-3-enoate aldolase.	NF	NF	NF	NF
7063	4.1.2.43	3-hexulose-6-phosphate synthase.	NF	NF	NF	NF
7064	4.1.2.45	Trans-o-hydroxybenzylidenepyruvate hydratase-ald	NF	NF	NF	NF
7065	4.1.2.5	Threonine aldolase.	NF	NF	FOUND	NF
7066	4.1.3.-	Oxo-acid-lyases.	FOUND	FOUND	FOUND	FOUND
7067	4.1.3.1	Isocitrate lyase.	NF	NF	NF	NF
7068	4.1.3.17	4-hydroxy-4-methyl-2-oxoglutarate aldolase.	NF	NF	NF	NF
7069	4.1.3.24	Malyl-CoA lyase.	NF	NF	NF	NF
7070	4.1.3.39	4-hydroxy-2-oxovalerate aldolase.	NF	NF	NF	NF
7071	4.1.99.-	Other carbon-carbon lyases.	NF	NF	NF	FOUND
7072	4.1.99.11	Benzylsuccinate synthase.	NF	NF	NF	NF
7073	4.2.1.-	Hydro-lyases.	FOUND	FOUND	FOUND	FOUND
7074	4.2.1.100	Cyclohexa-1,5-dienecarbonyl-CoA hydratase.	NF	NF	NF	NF
7075	4.2.1.103	Cyclohexyl-isocyanide hydratase.	NF	NF	NF	NF
7076	4.2.1.108	Ectoine synthase.	NF	NF	NF	NF
7077	4.2.1.11	Phosphopyruvate hydratase.	FOUND	FOUND	FOUND	FOUND
7078	4.2.1.112	Acetylene hydratase.	NF	NF	NF	NF
7079	4.2.1.116	3-hydroxypropionyl-CoA dehydratase.	NF	NF	NF	NF
7080	4.2.1.12	Phosphogluconate dehydratase.	NF	NF	NF	NF
7081	4.2.1.120	4-hydroxybutanoyl-CoA dehydratase.	NF	NF	NF	NF
7082	4.2.1.17	Enoyl-CoA hydratase.	NF	NF	NF	NF
7083	4.2.1.2	Fumarate hydratase.	NF	NF	NF	FOUND
7084	4.2.1.3	Aconitate hydratase.	NF	NF	FOUND	NF
7085	4.2.1.39	Gluconate dehydratase.	NF	NF	NF	NF
7086	4.2.1.52	Dihydrodipicolinate synthase.	FOUND	FOUND	FOUND	FOUND
7087	4.2.1.54	Lactoyl-CoA dehydratase.	NF	NF	NF	NF
7088	4.2.1.55	3-hydroxybutyryl-CoA dehydratase.	NF	NF	NF	NF
7089	4.2.1.69	Cyanamide hydratase.	NF	NF	NF	NF

7090	4.2.1.80	2-oxopent-4-enoate hydratase.	NF	NF	NF	NF
7091	4.2.1.83	4-oxalmesaconate hydratase.	NF	NF	NF	NF
7092	4.2.1.84	Nitrile hydratase.	NF	NF	NF	NF
7093	4.2.3.1	Threonine synthase.	NF	NF	FOUND	FOUND
7094	4.3.-.-	Carbon-nitrogen lyases.	FOUND	FOUND	FOUND	FOUND
7095	4.4.1.19	Phosphosulfolactate synthase.	NF	NF	NF	NF
7096	4.4.1.22	S-(hydroxymethyl)glutathione synthase.	NF	NF	NF	NF
7097	4.5.1.-	Carbon-halide lyases.	NF	NF	NF	NF
7098	4.5.1.1	DDT-dehydrochlorinase.	NF	NF	NF	NF
7099	4.5.1.3	Dichloromethane dehalogenase.	NF	NF	NF	NF
7100	4.99.1.7	Phenylacetaldoxime dehydratase.	NF	NF	NF	NF
7101	5.1.1.7	Diaminopimelate epimerase.	FOUND	FOUND	FOUND	FOUND
7102	5.1.2.-	Acting on hydroxy acids and derivatives.	NF	NF	NF	NF
7103	5.1.2.2	Mandelate racemase.	NF	NF	NF	NF
7104	5.1.3.1	Ribulose-phosphate 3-epimerase.	FOUND	FOUND	FOUND	FOUND
7105	5.1.3.15	Glucose-6-phosphate 1-epimerase.	NF	NF	NF	NF
7106	5.1.3.3	Aldose 1-epimerase.	NF	FOUND	NF	FOUND
7107	5.1.99.1	Methylmalonyl-CoA epimerase.	NF	NF	NF	NF
7108	5.1.99.3	Allantoin racemase.	NF	NF	NF	NF
7109	5.2.1.1	Maleate isomerase.	NF	NF	NF	NF
7110	5.2.1.10	2-chloro-4-carboxymethylenebut-2-en-1,4-olide iso	NF	NF	NF	NF
7111	5.2.1.2	Maleylacetoacetate isomerase.	NF	NF	NF	NF
7112	5.2.1.4	Maleylpyruvate isomerase.	NF	NF	NF	NF
7113	5.3.1.1	Triose-phosphate isomerase.	FOUND	FOUND	FOUND	FOUND
7114	5.3.1.27	6-phospho-3-hexuloisomerase.	NF	NF	NF	NF
7115	5.3.1.6	Ribose-5-phosphate isomerase.	FOUND	FOUND	FOUND	FOUND
7116	5.3.1.9	Glucose-6-phosphate isomerase.	FOUND	FOUND	FOUND	FOUND
7117	5.3.2.-	Interconverting keto- and enol- groups.	NF	NF	NF	NF
7118	5.3.3.-	Transposing C==C bonds.	FOUND	FOUND	FOUND	FOUND
7119	5.3.3.10	5-carboxymethyl-2-hydroxymuconate Delta-isomera	NF	NF	NF	FOUND
7120	5.3.3.3	Vinylacetyl-CoA Delta-isomerase.	NF	NF	NF	NF
7121	5.3.3.4	Muconolactone Delta-isomerase.	NF	NF	NF	NF
7122	5.3.99.-	Other intramolecular oxidoreductases.	NF	NF	NF	NF
7123	5.3.99.7	Styrene-oxide isomerase.	NF	NF	NF	NF
7124	5.4.2.1	Phosphoglycerate mutase.	FOUND	FOUND	FOUND	FOUND
7125	5.4.2.2	Phosphoglucomutase.	FOUND	FOUND	FOUND	FOUND
7126	5.4.2.9	Phosphoenolpyruvate mutase.	NF	NF	NF	NF
7127	5.4.4.1	(Hydroxyamino)benzene mutase.	NF	NF	NF	NF
7128	5.4.99.14	4-carboxymethyl-4-methylbutenolide mutase.	NF	NF	NF	NF
7129	5.4.99.2	Methylmalonyl-CoA mutase.	NF	NF	NF	NF
7130	5.5.1.-	Intramolecular lyases.	NF	NF	NF	NF
7131	5.5.1.1	Muconate cycloisomerase.	NF	NF	NF	NF
7132	5.5.1.11	Dichloromuconate cycloisomerase.	NF	NF	NF	NF
7133	5.5.1.2	3-carboxy-cis,cis-muconate cycloisomerase.	NF	NF	NF	NF
7134	5.5.1.7	Chloromuconate cycloisomerase.	NF	NF	NF	NF
7135	5.99.1.4	2-hydroxychromene-2-carboxylate isomerase.	NF	NF	NF	NF
7136	6.2.1.-	Acid--thiol ligases.	NF	NF	NF	NF
7137	6.2.1.1	Acetate--CoA ligase.	NF	NF	NF	NF
7138	6.2.1.13	Acetate--CoA ligase (ADP-forming).	NF	NF	NF	NF
7139	6.2.1.25	Benzoate--CoA ligase.	NF	NF	NF	NF
7140	6.2.1.27	4-hydroxybenzoate--CoA ligase.	NF	NF	NF	NF
7141	6.2.1.32	Anthranilate--CoA ligase.	NF	NF	NF	NF
7142	6.2.1.33	4-chlorobenzoate--CoA ligase.	NF	NF	NF	NF
7143	6.2.1.36	3-hydroxypropionyl-CoA synthase.	NF	NF	NF	NF
7144	6.2.1.4	Succinate--CoA ligase (GDP-forming).	NF	NF	NF	NF

7145	6.2.1.5	Succinate--CoA ligase (ADP-forming).	NF	NF	NF	NF
7146	6.2.1.9	Malate--CoA ligase.	NF	NF	NF	NF
7147	6.3.1.2	Glutamate--ammonia ligase.	NF	FOUND	FOUND	FOUND
7148	6.3.2.27	Aerobactin synthase.	NF	NF	NF	NF
7149	6.3.4.3	Formate--tetrahydrofolate ligase.	FOUND	FOUND	FOUND	FOUND
7150	6.3.5.4	Asparagine synthase (glutamine-hydrolyzing).	NF	NF	NF	FOUND
7151	6.4.1.-	Ligases that form carbon-carbon bonds.	NF	NF	FOUND	FOUND
7152	6.4.1.1	Pyruvate carboxylase.	NF	NF	NF	NF
7153	6.4.1.2	Acetyl-CoA carboxylase.	NF	NF	FOUND	FOUND
7154	6.4.1.3	Propionyl-CoA carboxylase.	NF	NF	NF	NF
7155	>Phosphatidylinositol signaling system 04070					
7156	2.7.1.107	Diacylglycerol kinase.	FOUND	FOUND	FOUND	FOUND
7157	2.7.1.127	Inositol-trisphosphate 3-kinase.	NF	NF	NF	NF
7158	2.7.1.137	Phosphatidylinositol 3-kinase.	NF	NF	NF	NF
7159	2.7.1.140	Inositol-tetrakisphosphate 5-kinase.	NF	NF	NF	NF
7160	2.7.1.149	1-phosphatidylinositol-5-phosphate 4-kinase.	NF	NF	NF	NF
7161	2.7.1.150	1-phosphatidylinositol-3-phosphate 5-kinase.	NF	NF	NF	NF
7162	2.7.1.153	Phosphatidylinositol-4,5-bisphosphate 3-kinase.	NF	NF	NF	NF
7163	2.7.1.154	Phosphatidylinositol-4-phosphate 3-kinase.	NF	NF	NF	NF
7164	2.7.1.158	Inositol-pentakisphosphate 2-kinase.	NF	NF	NF	NF
7165	2.7.1.159	Inositol-1,3,4-trisphosphate 5/6-kinase.	NF	NF	NF	NF
7166	2.7.1.67	1-phosphatidylinositol 4-kinase.	NF	NF	NF	NF
7167	2.7.1.68	1-phosphatidylinositol-4-phosphate 5-kinase.	NF	NF	NF	NF
7168	2.7.11.13	Protein kinase C.	NF	NF	NF	NF
7169	2.7.7.41	Phosphatidate cytidylyltransferase.	FOUND	FOUND	FOUND	FOUND
7170	2.7.8.11	CDP-diacylglycerol--inositol 3-phosphatidyltransfera	NF	NF	NF	NF
7171	3.1.3.25	Inositol-phosphate phosphatase.	NF	NF	NF	NF
7172	3.1.3.36	Phosphoinositide 5-phosphatase.	NF	NF	NF	NF
7173	3.1.3.56	Inositol-polyphosphate 5-phosphatase.	NF	NF	NF	NF
7174	3.1.3.57	Inositol-1,4-bisphosphate 1-phosphatase.	NF	NF	NF	NF
7175	3.1.3.64	Phosphatidylinositol-3-phosphatase.	NF	NF	NF	NF
7176	3.1.3.66	Phosphatidylinositol-3,4-bisphosphate 4-phosphata	NF	NF	NF	NF
7177	3.1.3.67	Phosphatidylinositol-3,4,5-trisphosphate 3-phospha	NF	NF	NF	NF
7178	3.1.4.11	Phosphoinositide phospholipase C.	NF	NF	NF	NF
