# Supplementary material for: Genomic sequence analysis and characterization of Sneathia amnii sp. nov
Source: BMC Genomics. 2012 Dec 17;13(Suppl 8):S4. doi: 10.1186/1471-2164-13-S8-S4 (PMC3535699; doi:10.1186/1471-2164-13-S8-S4)
Supplement: Additional file 1 — Supplementary figure S1 - Multiple sequence alignment of 16S rDNA from S. amnii and related organisms. Alignment of 16S rDNA sequences of S. amnii and representative strains belonging to seven representants of the Fusobacteriaceae family: Sneathia, Streptobacillus, Leptotrichia, Sebaldella, Propionigenium, Ilyobacter, and Fusobacterium. [file 1471-2164-13-S8-S4-S1.pdf]

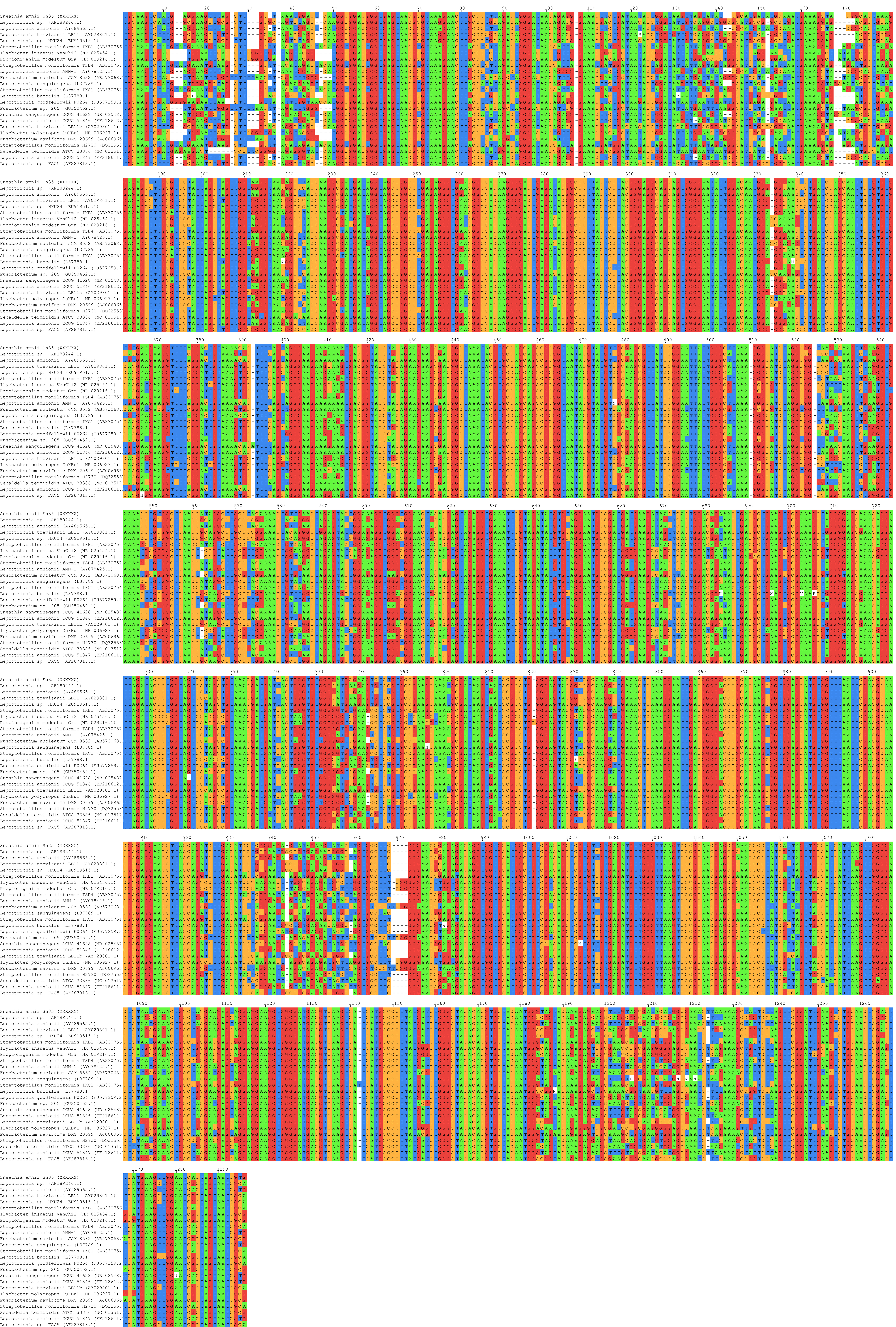
Sneathia amnii Sn35 (XXXXXX)
Leptotrichia sp. (AF189244.1)
Leptotrichia amnionii (AY489565.1)
Leptotrichia trevisanii LB11 (AY029801.1)
Leptotrichia sp. HKU24 (EU919515.1)
Streptobacillus moniliformis IKB1 (AB330756.
Ilyobacter insuetus VenChi2 (NR 025454.1)
Propionigenium modestum Gra (NR 029216.1)
Streptobacillus moniliformis TSD4 (AB330757.
Leptotrichia amnionii AMN-1 (AY078425.1)
Fusobacterium nucleatum JCM 8532 (AB573068.1
Leptotrichia sanguinegens (L37789.1)
Streptobacillus moniliformis IKC1 (AB330754.
Leptotrichia buccalis (L37788.1)
Leptotrichia goodfellowii F0264 (FJ577259.2)
Fusobacterium sp. 205 (GU350452.1)
Sneathia sanguinegens CCUG 41628 (NR 025487.
Leptotrichia amnionii CCUG 51846 (EF218612.1
Leptotrichia trevisanii LB11b (AY029801.1)
Ilyobacter polytropus CuHBu1 (NR 036927.1)
Fusobacterium naviforme DMS 20699 (AJ006965.
Streptobacillus moniliformis H2730 (DQ325537
Sebaldella termitidis ATCC 33386 (NC 013517)
Leptotrichia amnionii CCUG 51847 (EF218611.1
Leptotrichia sp. FAC5 (AF287813.1)
